# Enaminone-based carboxylic acids as novel non-classical carbonic anhydrases inhibitors: design, synthesis and *in vitro* biological assessment

**DOI:** 10.1080/14756366.2022.2114079

**Published:** 2022-08-23

**Authors:** Mahmoud F. Abo-Ashour, Hadia Almahli, Alessandro Bonardia, Amira Khalil, Tarfah Al-Warhi, Sara T. Al-Rashood, Hatem A. Abdel-Aziz, Alessio Nocentini, Claudiu T. Supuran, Wagdy M. Eldehna

**Affiliations:** a Department of Pharmaceutical Chemistry, Faculty of Pharmacy, El saleheya El Gadida University, Cambridge, Egypt; b Department of Chemistry, University of Cambridge, Cambridge, United Kingdom; c Department of NEUROFARBA, Section of Pharmaceutical and Nutraceutical Sciences, University of Florence, Firenze, Italy; d Pharmaceutical Chemistry Department, Faculty of Pharmacy, The British University in Egypt (BUE), Cairo, Egypt; e Department of Chemistry, College of Science, Princess Nourah Bint Abdulrahman University, Riyadh, Saudi Arabia; f Department of Pharmaceutical Chemistry, College of Pharmacy, King Saud University, Riyadh, Saudi Arabia; g Department of Applied Organic Chemistry, National Research Center, Dokki, Egypt; h Department of Pharmaceutical Chemistry, Faculty of Pharmacy, Kafrelsheikh University, Kafrelsheikh, Egypt

**Keywords:** Carbonic anhydrase, H-NMR, enaminone, stopped-flow assay

## Abstract

In searching for new molecular drug targets, Carbonic Anhydrases (CAs) have emerged as valuable targets in diverse diseases. CAs play critical functions in maintaining pH and CO_2_ homeostasis, metabolic pathways, and much more. So, it is becoming attractive for medicinal chemists to design novel inhibitors for this class of enzymes with improved potency and selectivity towards the different isoforms. In the present study, three sets of carboxylic acid derivatives **5a–q**, **7a–b** and **12a–c** were designed, developed and evaluated for the hCA inhibitory effects against hCA I, II, IX and XII. Compounds **5l, 5m,** and **5q** elicited the highest inhibitory activities against hCA II, IX and XII. In summary, structural rigidification, regioisomerism and structural extension, all played obvious roles in the degree of hCA inhibition. This present work could be a good starting point for the design of more non-classical selective hCA inhibitors as potential targets for several diseases.

## 1. Introduction

The Zn(II) metalloenzymes carbonic anhydrases (CA, EC 4.2.1.1) is a very significant family in humans and in most living organisms that catalyses the reversible CO_2_ hydration reaction to bicarbonate ion^1^. This fundamental reaction orchestrates several physiological processes requiring pH control and ion transport^2^. Fifteen humans (*h*) CA isoforms have been discovered so far, and they showed diverse distribution among tissues and cells.

Dysfunction of *h*CA activities results in several pathological consequences, highlighting these isozymes as promising drug targets for diverse therapeutic interventions with small molecule CA inhibitors (CAIs) ^3^. Accordingly, the pharmacological applications of several CAIs were reported for the management of different diseases including ophthalmologic problems^4^^,^^5^, human malignancies^6^, high-altitude sickness^7^, epilepsy^8^, peptic ulcers^9^, obesity^10^, and congestive heart failure^11^.

**Figure 1.**
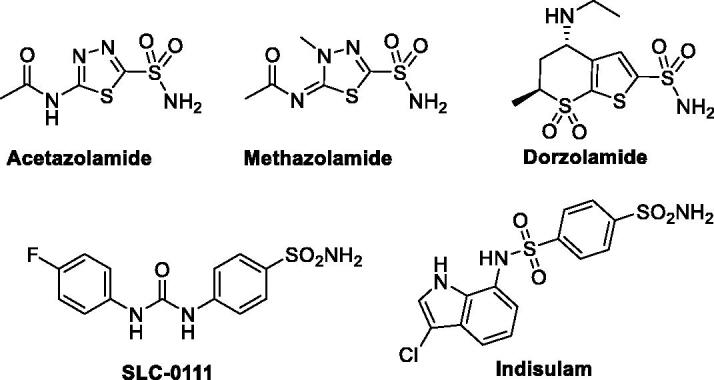
Structures of certain sulphonamide-based CAIs in clinical use and in clinical trials.

Carbonic anhydrases are classically inhibited by small molecules tethered with the primary sulphonamide-based zinc binding group (ZBG), as well as its bioisosteres such as sulfamides and sulfamates^12–14^. Although several chemotypes of CA inhibitors have been identified in the last few decades (such as coumarins, phenols, thiocarbamates, and carboxylates)^15–17^ only CA inhibitors based on sulphonamides could be used in the clinical setting for glaucoma treatment (such as acetazolamide, methazolamide and dorzolamide, Figure 1), and in clinical trials for the treatment of human malignancies (such as SLC-0111 and indisulam, Figure 1)^18^^,^^19^. Accordingly, the development of non­classical CA inhibitors has emerged as a promising approach to discovering new efficient CA inhibitors for the management of various disorders.

The carboxylic acid-bearing small molecules represent an important category of the non-classical carbonic anhydrase inhibitors^20^. Interestingly, they can inhibit the metalloenzymes *via* various modes of action. Firstly, they are able to bind directly to the catalytic zinc displacing bound water-hydroxide anion, similarly to sulphonamides. Alternatively, certain carboxylic acid derivatives can anchor to the zinc-bound water-hydroxide ion *via* a hydrogen bonding, similar to the binding mode noticed for phenol-based molecules. Lastly, carboxylates may bind within an adjacent pocket to the entrance which is located outside the active site of the carbonic anhydrase. This interaction results in a block of the proton shuttle His64 residue in its “out” conformation, leading to the inhibition of CA catalytic activity^21–23^.

**Figure 2.**
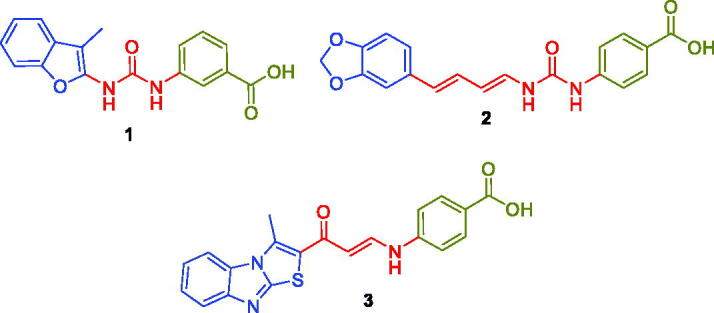
Structures for some reported carboxylic acid derivatives as non-classical CA inhibitors.

In 2020, our research team has reported novel series of benzofuran-based carboxylic acid small molecules that tethered with benzoic or hippuric acid motifs as potential CA inhibitors^24^. These acid moieties are linked to 5-bromobenzofuran or 2-methylbenzofuran tail through an ureido linker. Among these acid derivatives, *m*-benzoic acid-bearing derivative (Compound **1**, Figure 2) emerged as submicromolar *h*CA IX inhibitor (*K*_I_ = 0.79 µM), as well as potent *h*CAXII inhibitor (*K*_I_ = 2.3 µM). Moreover, we developed another ureides series of piperine-based carboxylic acid derivatives that incorporate benzoic acid moieties linked to the natural product piperine through an ureido spacer^25^. Acid derivative **2** (Figure 2) exerted moderate inhibition activities against both CA IX and XII isoforms (*K*_I_ = 16.1 µM and 14.4 µM, respectively).

**Figure 3.**
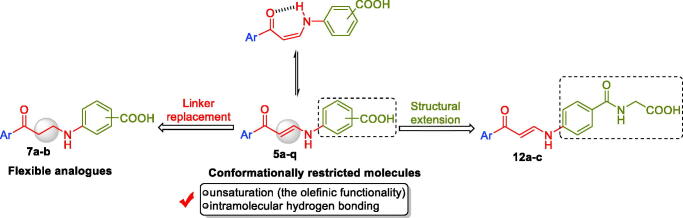
Design for herein reported carboxylic acid derivatives **5a–q**, **7a–b** and **12a–c**.

Also, in 2020 we designed and prepared a novel methylthiazolo[3,2-*a*]benzimidazole-tethered carboxylic acid derivatives as potential CA inhibitors^26^. In this work, the enaminone linker was utilised to link the ZBG benzoic acid motifs to the thiazolo[3,2-*a*]benzimidazole tail^26^. Enaminone-based carboxylic acid **3** (Figure 2) effectively inhibited *h*CA IX and XII isoforms (*K*_I_ = 0.83 µM and 2.4 µM, respectively). In the current work, we devoted our effort to developing new non-classical CA inhibitors through the design of three sets of new carboxylic acid derivatives (**5a–q**, **7a–b** and **12a–c**), Figure 3.

The first (**5a–q**) and the second (**7a–b**) sets incorporated a benzoic acid moiety that is connected to different aryl tails through an enaminone linker, for the first set, or through the more flexible saturated 3-oxo-propylamine linker, for the second set, Figure 3. The incorporation of the enaminone linker is expected to provide more rigid molecules, than the corresponding molecules that tethered with the 3-oxo-propylamine linker. The expected conformational restriction in the enaminone-bearing carboxylic acids (**5a–q**) should be attributed to the unsaturation (the olefinic functional group), as well as due to the formation of an intramolecular hydrogen bonding that resulted in a pseudo-six-membered ring. Lastly, a structural extension approach was utilised to replace the benzoic acid motif with the hippuric acid to furnish the third series **12a–c** (Figure 3). The potential ability of the target carboxylic acids (**5a–q**, **7a–b** and **12a–c**) to inhibit *h*CA I, II, IX and XII isoforms was assessed *via* the stopped-flow carbon dioxide hydrase assay.

## 2. Results and discussion

### 2.1. Chemistry

**Scheme 1.**
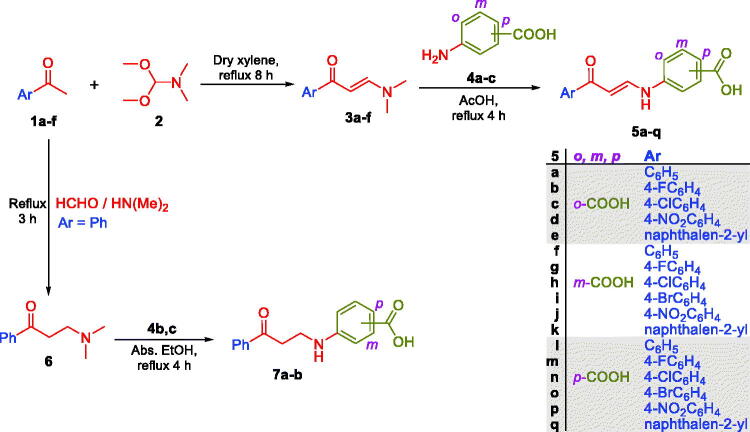
Synthesis of benzoic acids-bearing enaminones **5a–q** and 3/4-((3-oxo-3-phenylpropyl)amino)benzoic acids **7a–b.**

**Scheme 2.**
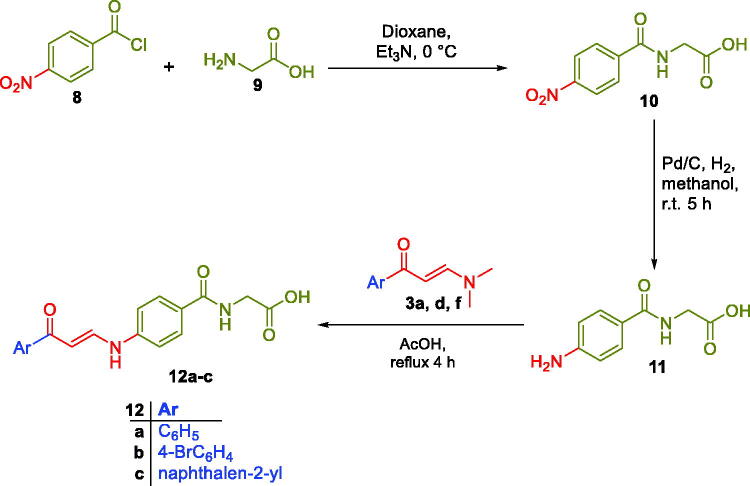
Synthesis of hippuric acids-bearing enaminones **12a–c.**

In this study, the synthesis of the new carboxylic acids-based carbonic anhydrase inhibitors **5a–q**, **7a–b** and **12a–c** is outlined in Schemes 1 and 2. In Scheme 1, six aryl methyl ketones **1a–f** were condensed with DMF-DMA to furnish the corresponding aryl enaminones intermediates **3a–f** that subsequently reacted with *ortho*, *meta* and *para* aminobenzoic acids **4a–c** in glacial acetic acid to produce the target carboxylic acids **5a–q**. On the other hand, acetophenone **1a** has been reacted with dimethyl amine and formaldehyde *via* Mannich reaction to get 3-(dimethylamino)-1-phenylpropan-1-one **6**, which subsequently reacted with *m*- and *p*- aminobenzoic acids **4b–c** in absolute ethanol to furnish targeted carboxylic acids **7a–b** with saturated linker between the ZBG and the aryl tail.

In Scheme 2, glycine **9** was acylated with 4-nitrobenzoyl chloride **8** in dry dioxan to get (4-nitrobenzoyl)glycine **10**, which was then reduced to the corresponding (4-aminobenzoyl)glycine **11**. The third set of the target carboxylic acids with the desired structural extension **12a–c** was prepared through the nucleophilic substitution reaction of (4-aminobenzoyl)glycine **11** with the previously prepared enaminones **3a,d,f** in a boiling glacial acetic acid (Scheme 2). The proposed structure for carboxylic acids enaminones **5a–q**, **7a–b** and **12a–c** were supported by the elemental and spectral data.

**Figure 4.**
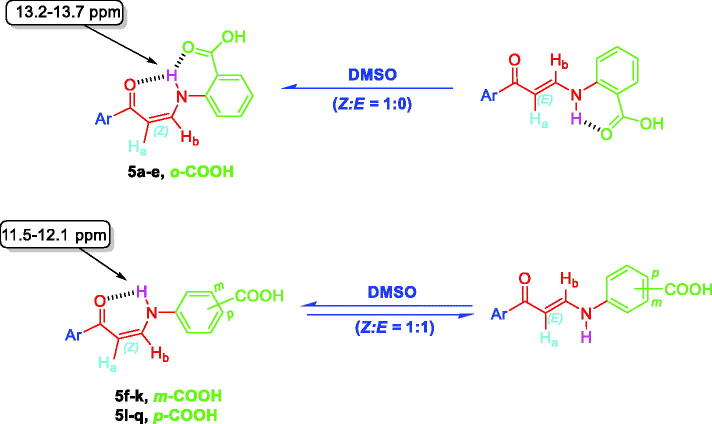
The existence of *ortho*-substituted carboxylic acids enaminones **5a–e** in *Z*-form and the existence of *meta*- and *para*-substituted carboxylic acids enaminones **5f–k** and **5l–q** in *E/Z-*forms (1:1) in DMSO.

^1^H NMR spectra of 2-substituted carboxylic acids bearing enaminones **5a–e** revealed they are existing in *Z*-form around the olefinic bond which was evidenced by up-field doublets of *Z*-H_a_ around *δ* 6.13–6.37 *ppm* with coupling constant (*J*) equals 8.0 Hz. The latter *Z*-form was assumed to be established through the formation of two intramolecular hydrogen bonds that resulted in two pseudo-six-membered rings. ^1^H NMR spectra of *ortho*-carboxylic acids bearing enaminones **5a–e** exhibited a doublet D_2_O exchangeable signal that was integrated for one proton, attributable to the NH group of the *Z-*form, at more down-field region *δ* 13.2–13.7 *ppm* when compared with those of 3- and 4-substituted carboxylic acids bearing enaminones **5f–k** and **5l–q**, respectively, at *δ* 11.5–12.1 *ppm* (Figure 4). This negative shift could be explained by the presence of the two intramolecular hydrogen bonds. The coupling constant value of these doublets is 12.0 Hz which suggests the *trans* direction of NH around HN-CH_b_ in enaminones **5a–e**.

**Figure 5.**
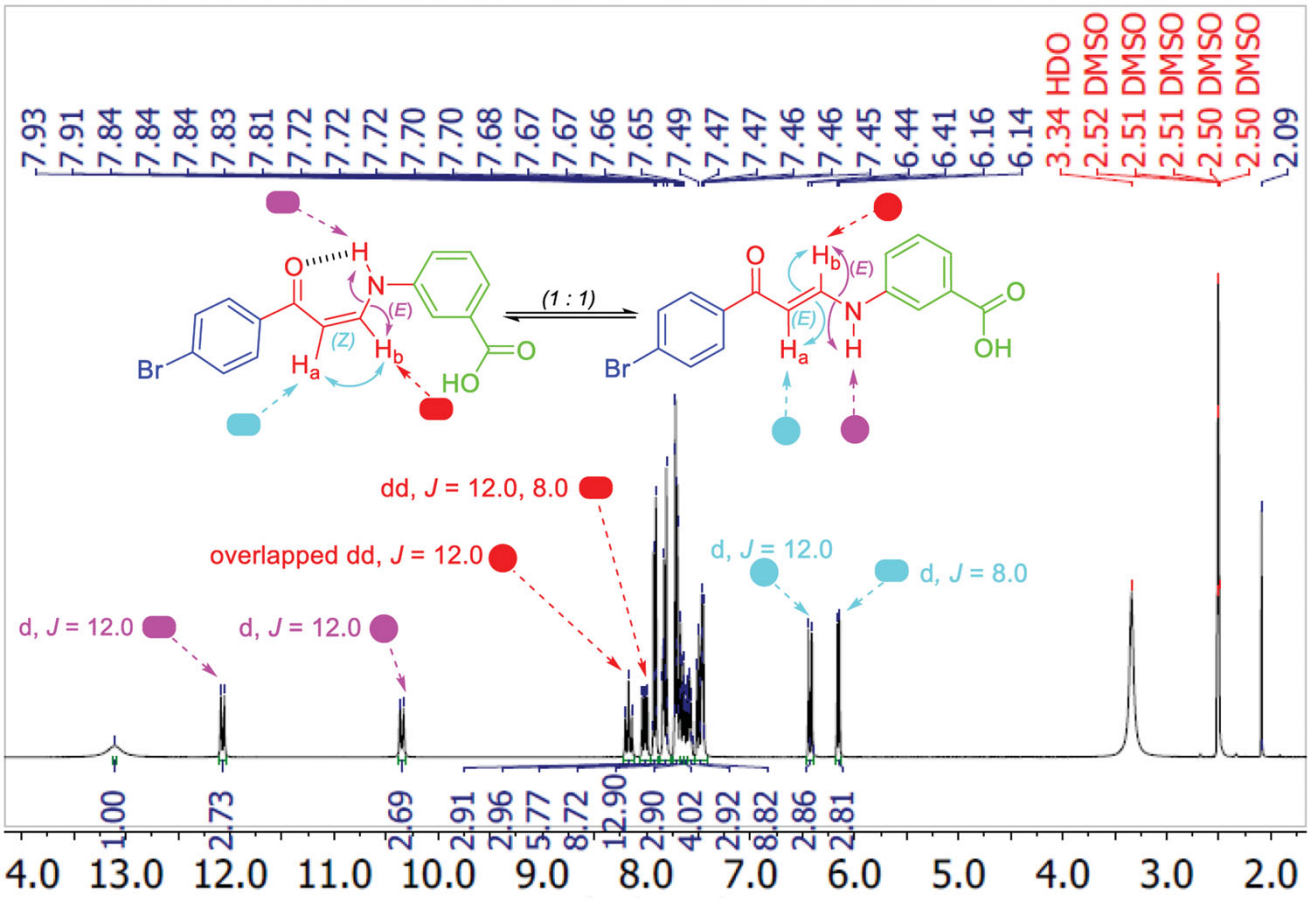
^1^H NMR of enaminone **5i** showing the presence of *Z*-form [*Z* H_a_ (d), *Z* H_b_ (dd), *E* NH (d)] and *E*-form [*E* H_a_ (d), *E* H_b_ (overlapped dd), *E* NH (d)] in DMSO as a representative example for enaminones **5f–k** and **5l–q**.

^1^H NMR spectra of 3- and 4-substituted carboxylic enaminones **5f–k** and **5l–q** disclosed their presence in *Z/E* geometric conjugation around H_a_C=CH_b_ in an equal ratio (*Z:E* = 1:1) according to the integrations of their signals. The presence of *E*-isomer was evidenced *via* the *J* constant for olefinic protons H_a_ and H_b_ (*J_Ha-Hb_* = 12.00 Hz) which was determined by the down-field doublet signal of *E*-H_a_ around *δ* 6.4–6.7 *ppm* (Figures 4 and 5). In a similar way, the presence of the *Z*-form was revealed by determining the *J* constant for the olefinic protons Ha and Hb (*J* = 8.0 Hz) that came from the up-field doublet signals of *Z*-H_a_ around *δ* 6.13–6.41 *ppm*. Furthermore, the ^1^H NMR spectra displayed 2 doublet signal sets, integrated for one proton, each belonging to the (NH) proton for the *E* and *Z* isomers around *δ* 10.28–10.59 and 12.03–12.14 *ppm* (Figures 4 and 5).

The downfield signals of NH proton could be attributed to the *Z* isomer and the up-field signals for the same group to the *E* isomer according to their integration values as explained in our previous study^27^. The coupling constants for these doublets are 12.0 Hz which suggests the presence of NH of enaminones **5f–k** and **5l–q** in *trans* direction for H_b_ around HN-CH_b_ bond in both forms *E* and *Z* (Figures 4 and 5). Moreover, *Z* (NH) appeared down-field, due to the formation of intra-molecular hydrogen bonding with the carbonyl oxygen. However, this hydrogen bond between C=O and NH groups was absolutely confirmed, in a previous study, by X-ray single crystal analysis of a sulphonamide analog for compound **5** which exhibited a *Z* configuration around H_a_C=CH_b_ with the *trans* HN-CH_b_^27^_._

**Figure 6.**
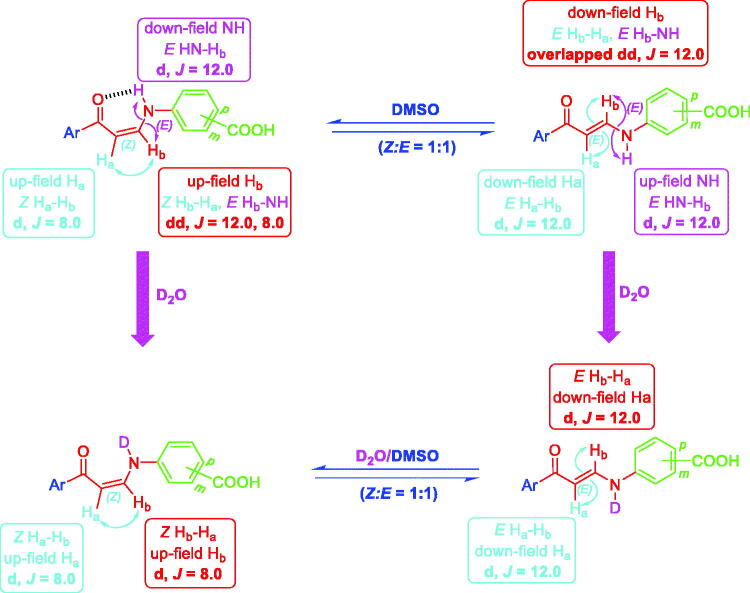
The presence of *meta*- and *para*-substituted carboxylic enaminones **5f–k** and **5l–q** in *Z/E-*forms in DMSO and in D_2_O/DMSO (^1^H NMR).

Interestingly, the ^1^H NMR of *meta*-substituted carboxylic enaminones **5g** and **5i**, as well as ^1^H NMR for *para*-substituted carboxylic enaminones **5o**, among **5f–k** and **5l–q**, revealed the appearance of down-field *Z*-H_b_ signal around *δ* 8.00 *ppm* as dd with *J* = 12.0 and 8.0 Hz due to *J_E-Hb-NH_* and *J_Z-Hb-Ha_*, respectively, whereas the up-field *E*-H_b_ signal appeared around *δ* 8.14 as overlapped dd with two *J* = 12.0 Hz (*J_E-Hb-NH_* = *J_E-Hb-Ha_* = 12.0 Hz). It is worthy to note that after the addition of D_2_O to the DMSO solution of these compounds (**5g**, **5i** and **5o**) in their NMR tube, the coupling of NH proton for H_b_ in both *Z* and *E* forms disappeared and the dd of *Z*-H_b_ was converted to doublet signal due to the coupling of Z-H_a_ only with *J* = 8.0 Hz parallel with the conversion of overlapped dd of *E*-H_b_ to doublet signal due to *E*-H_a_ coupling only with *J* = 12.0 Hz (Figure 6).

**Figure 7.**
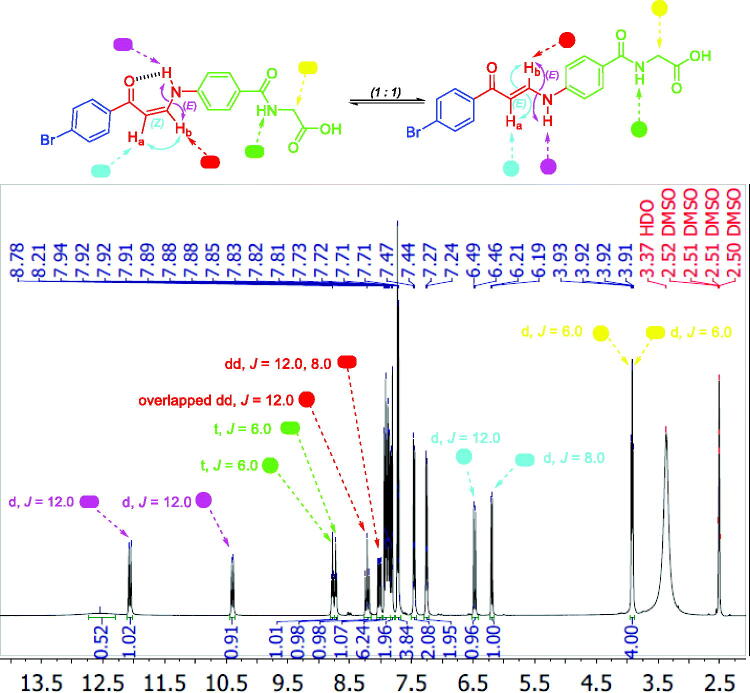
^1^H NMR of enaminone **12b** which showed the existence of *Z*- and *E*-form in DMSO as a representative example for **12a–c**.

Furthermore, the ^1^H NMR of 4-carboxamido enaminone **12b**, as a representative example for enaminones **12a–c**, showed the appearance of two sets of doublet signals, integrated for the two protons of –CH_2_- at *δ* 3.91 and *δ* 3.92 and two sets of triplet, for the proton of the amidic -NH group of at *δ* 8.74 and *δ* 8.79 beside two sets of doublet *Z/E* H_a_ and *Z/E*, H_b_, and two dd of *E NH*, Figure 7.

### 2.2. Biological evaluation

#### 2.2.1. CAS inhibition

In the current study, we have explored the inhibitory activities of the synthesised carboxylic acid derivatives **5a–q**, **7a–b** and **12a–c** against different carbonic anhydrase isoforms (I, II, IX and XII) using acetazolamide (AAZ) as a reference CA inhibitor. The resulted inhibition constants were presented in Table 1.

**Table 1.** *h*CA I, II, IX and XII inhibition results from the carboxylic acid derivatives **5a–q**, **7a–b** and **12a–c** and acetazolamide (**AAZ**) as a reference CA inhibitor. 
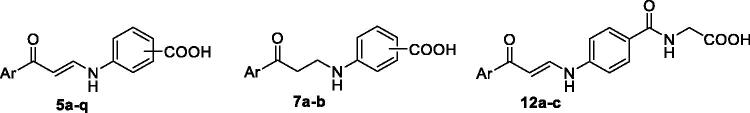

Cmpd.	COOH	Ar	K_I_ (μM)^a^			
			hCA I	hCA II	hCA IX	hCA XII
**5a**	*ortho*	-C_6_H_5_	>100	39.4	10.6	6.4
**5b**	*ortho*	−4-F-C_6_H_4_	>100	24.3	13.2	4.3
**5c**	*ortho*	−4-Cl-C_6_H_4_	>100	32.7	17.7	12.5
**5d**	*ortho*	−4-NO_2_-C_6_H_4_	>100	26.9	14.1	7.8
**5e**	*ortho*	−2-Naph	>100	49.0	19.3	15.7
**5f**	*meta*	-C_6_H_5_	>100	72.8	15.9	9.5
**5g**	*meta*	−4-F-C_6_H_4_	>100	68.2	9.6	10.3
**5h**	*meta*	−4-Cl-C_6_H_4_	>100	77.1	19.2	8.6
**5i**	*meta*	−4-Br-C_6_H_4_	>100	81.4	32.5	13.9
**5j**	*meta*	−4-NO_2_-C_6_H_4_	>100	80.7	24.7	8.9
**5k**	*meta*	−2-Naph	>100	92.3	11.4	9.7
**5l**	*para*	-C_6_H_5_	75.3	9.8	1.2	0.97
**5m**	*para*	−4-F-C_6_H_4_	68.8	8.7	0.92	1.1
**5n**	*para*	−4-Cl-C_6_H_4_	81.5	13.5	3.4	4.6
**5o**	*para*	−4-Br-C_6_H_4_	>100	15.3	5.6	6.2
**5p**	*para*	−4-NO_2_-C_6_H_4_	95.6	12.6	4.1	0.85
**5q**	*para*	−2-Naph	79.4	7.4	0.76	1.5
**7a**	*meta*	-C_6_H_5_	>100	64.3	23.7	7.4
**7b**	*para*	-C_6_H_5_	63.8	16.7	8.3	9.1
**12a**	–	-C_6_H_5_	>100	>100	>100	>100
**12b**	–	−4-Br-C_6_H_4_	>100	>100	>100	>100
**12c**	–	−2-Naph	>100	>100	>100	>100
**AAZ**	–	–	0.250	0.012	0.025	0.006

^a^Mean from 3 different assays (errors were in the range of ± 5–10% of the reported values).

The stopped-flow assay outputs disclosed that the off-target cytosolic *h*CA I exhibited the lowest inhibition in this study. Both the ortho and meta carboxylic acids bearing enaminones (**5a–e** and **5f–k**) didn’t exert any noticeable inhibitory activity towards this isoform (*K*_I_ > 100 µM), whereas the para carboxylic acids bearing enaminones **5l**, **5m**, **5n**, **5p** and **5q** exhibited weak inhibitory effect (*K*_I_ = 75.3, 68.8, 81.5, 95.6 and 79.4 µM, respectively), Table 1. In like manner, the target carboxylic acids **7a–b** with a saturated 3-oxo-propylamine linker showed the same behaviour; the meta carboxylic acid derivative **7a** emerged as inactive, whereas the para carboxylic acid derivative **7b** emerged as a weak inhibitor for the *h*CA I isoform (*K*_I_ = 63.8 µM), Table 1.

Moreover, the stopped-flow assay outputs showed that the physiologically relevant *h*CA II isoform was affected by herein reported carboxylic acids bearing enaminones **5a–q** and carboxylic acids **7a–b**. Both the ortho and meta carboxylic acids bearing enaminones (**5a–e** and **5f–k**) moderately inhibited *h*CA II isoform with inhibition constants spanning in the ranges 24.3–49.0 µM and 68.2–92.3 µM, respectively, whereas the para carboxylic acids bearing enaminones **5l–q** effectively inhibited this isoform (*K*_I_ range: 7.4–15.3 µM). In addition, carboxylic acids **7a–b** with a saturated 3-oxo-propylamine linker displayed *K*_I_ values equal to 64.3 µM and 16.7 µM, respectively. In particular, the best inhibitory activity in the CO_2_ hydrase assay was demonstrated by the para carboxylic acids bearing enaminones **5l**, **5m** and **5q** that showed *K*_I_ values in the single-digit micromolar range equal to 9.8 µM, 8.7 µM and 7.4 µM, respectively. Also, carboxylic acids **5n**, **5o**, **5p** and **7b** exhibited good activity against *h*CA II isoform with *K*_I_ = 13.5 µM, 15.3 µM, 12.6 µM and 16.7 µM, respectively.

Regarding the effect of the regioisomerism with the enaminones series **5a–q**, it was found that shifting the carboxylic acid group from the ortho position (compounds **5a–e**) to the meta position (compounds **5f–k**) resulted in decreasing activity. For example, the phenyl and naphthyl-bearing enaminones **5f** and **5k** displayed about 2-fold decreased potency (*K*_I_ = 72.8 µM and 92.3 µM, respectively), in comparison to their ortho counterparts **5a** and **5e** (*K*_I_ = 39.4 µM and 49.0 µM, respectively), Table 1. In turn, shifting the carboxylic acid functionality from the ortho position (enaminones **5a–e**) to the para position (enaminones **5l–q**) led to about 2–6.5 fold enhancement of the inhibitory activity against *h*CA II isoform. For example, the phenyl and naphthyl-bearing enaminones **5l** and **5q** emerged as more potent inhibitors (*K*_I_ = 9.8 µM and 7.4 µM, respectively) than their ortho analogues **5a** and **5e** (*K*_I_ = 39.4 µM and 49.0 µM, respectively). Similarly in the second set, the para carboxylic acid derivative **7b** displayed better activity (*K*_I_ = 16.7 µM) than its meta counterpart **7a** (*K*_I_ = 64.3 µM) against *h*CA II isoform, Table 1.

The trans-membrane *h*CA IX, the third examined isoform, was effectively inhibited by all carboxylic acids bearing enaminones **5a–q** and carboxylic acids **7a–b** evaluated here, with inhibition constants spanning in the range 0.76–32.5 µM. The best inhibitory action towards *h*CA IX isoform has been observed for enaminones **5m** and **5q** with sub-micromolar inhibition constants (0.92 µM and 0.76 µM, respectively), whereas compounds **5g**, **5l**, **5n**, **5o**, **5p** and **7b** exerted good inhibitory activity with *K*_I_s in the single-digit micromolar range equal to 9.6 µM, 1.2 µM, 3.4 µM, 5.6 µM, 4.1 µM and 8.3 µM, respectively.

As discussed above for the inhibitory activity against *h*CA II, grafting the carboxylic acid functionality at the para position (enaminones **5l–q**) resulted in a much-enhanced activity (*K*_I_ range: 0.76–5.6 µM) in comparison to the ortho (enaminones **5a–e**; *K*_I_ range: 10.6–19.3 µM) and meta (enaminones **5f–k**; *K*_I_ range: 11.4–32.5 µM) counterparts, Table 1. For example, the phenyl and naphthyl-bearing enaminones **5l** and **5q** showed about 9- and 25-fold enhanced potency (*K*_I_ = 1.2 µM and 0.76 µM, respectively) in comparison to their ortho counterparts **5a** and **5e** (*K*_I_ = 10.6 µM and 19.3 µM, respectively), and showed about 13- and 15-fold increased activity in comparison to their meta counterparts **5f** and **5k** (*K*_I_ = 15.9 µM and 11.4 µM, respectively). In like manner, the para carboxylic acid derivative **7b** exerted better *h*CA IX inhibitory activity (*K*_I_ = 8.3 µM) than its meta counterpart **7a** (*K*_I_ = 23.7 µM), Table 1.

On the other hand, examining the impact of the substitution of the phenyl tail within the most potent enaminones series (**5l–q**) revealed that only grafting the small fluorine substituent (enaminone **5m**; *K*_I_ = 0.92 µM) can enhance the activity of the unsubstituted phenyl-bearing enaminone **5l** (*K*_I_ = 0.92 µM). In turn, substituting the phenyl tail with 4-Cl, 4-Br or 4-NO_2_ substituents reduced the activity. Moreover, the replacement of the phenyl tail with a naphthyl one resulted in the most potent *h*CA IX inhibitor in this work (enaminone **5m**; *K*_I_ = 0.76 µM), Table 1. Notably, the same SAR could be observed for the ortho carboxylic acids bearing enaminones **5f–k**.

It is worthy to mention that the phenyl-bearing enaminones **5f** and **5l** exhibited better activity against *h*CA IX (*K*_I_ = 15.9 µM and 1.2 µM, respectively) than their flexible counterparts **7a–b** that bear the saturated 3-oxo-propylamine linker (*K*_I_ = 23.7 µM and 8.3 µM, respectively) which hints out that the conformational restriction for herein reported compounds is more favourable for *h*CA IX inhibitory action.

The newly reported carboxylic acids (**5a–q** and **7a–b**) efficiently inhibited the trans-membrane *h*CA XII isoform with *K*_I_ range of 0.85–15.7 µM. Uniquely, the para carboxylic acids bearing enaminones **5l** and **5p** induced inhibition in the submicromolar range, with *K*_I_s of 0.97 µM and 0.85 µM, respectively. Similarly, to the inhibition profile for both *h*CA II and IX isoforms, the para carboxylic acids bearing enaminones (**5l–q**) exerted more potent activity (*K*_I_ range: 0.85–6.2 µM) than their ortho (enaminones **5a–e**; *K*_I_ range: 4.3–15.7 µM) and meta (enaminones **5f–k**; *K*_I_ range: 8.6–13.9 µM) counterparts, Table 1. With an exception for the 4-NO_2_ substitution (enaminone **5p**; *K*_I_ = 0.85 µM), neither substitution of the phenyl tail nor its bioisosteric replacement with the naphthyl moiety enhanced the *h*CA XII inhibitory activity.

Finally, the third set of hippuric acid-bearing enaminones **12a–c** couldn’t inhibit any of the examined CA isoforms up to 100 µM, highlighting that the structural extension approach is not appropriate for the CA inhibitory activity (Table 1).

## 3. Conclusion

Three sets of our designed non-classical CA inhibitors (**5a–q**, **7a–b** and **12a–c**) were synthesised and evaluated for their CA inhibitory activity. The first (**5a–q**) and the second (**7a–b**) sets incorporated a benzoic acid moiety that is connected to different aryl tails through an enaminone linker, for the first set, or through the more flexible saturated 3-oxo-propylamine linker, for the second set. For the third set, a structural extension approach was utilised to replace the benzoic acid motif with the hippuric acid to furnish the carboxylic acid derivatives **12a–c**. The inhibitory activity for the prepared carboxylic acids was variable across the four tested CA isoforms, where for example, most compounds showed no or weak inhibition towards *h*CA I. Furthermore, compounds **5q**, **5m**, and **5l** exhibited a very similar inhibition pattern towards *h*CA II and IX with K_I_ values equal to 7.4, 8.7, 9.8 and 0.76, 0.92, 1.2 µM respectively. Regarding *h*CA XII, the para nitro-substituted derivative **5p** showed the highest inhibitory activity. Structural extension as done in the third set **12a–c**, showed no inhibitory actions towards all tested hCA isoforms, which suggested that this extension might lead to undesired interactions within the CA active site. Generally, ortho/meta carboxylic acid substituted derivatives exhibited weaker inhibitory activities against the tested *h*CAs. Future studies could reveal the desired pharmacophoric features which could enhance *h*CA inhibitory actions, as well as selectivity across different isoforms, that belong to this family of enzymes.

## 4. Experimental

### 4.1. Chemistry

#### 4.1.1. General

Stuart melting point apparatus was used for melting point measurements and were uncorrected. Schimadzu FT-IR 8400S spectrophotometer was used to record Infra-red (IR) spectra as KBr discs. NMR Spectra were recorded on a Bruker NMR spectrometer, at 400 and 100 MHz for ^1^H spectrum and ^13 ^C spectrum, respectively, and were run at 100 MHz in deuterated dimethylsulphoxide (DMSO-*d_6_*). HRMS spectra were recorded by a Bruker MicroTOF spectrometer.

#### 4.1.2. Synthesis of intermediates 3, 6 and 10

3-(Dimethylamino)-1-phenylprop-2-en-1-ones (**3a–f**^28^) 3-(dimethylamino)-1-phenylpropan-1-one (**6**^29^) and (4-aminobenzoyl)glycine (**11**^30^) were prepared according to the literature procedures.

#### 4.1.3. *Synthesis of the target carboxylic acids enaminones* 5a–q

An amount of 0.6 mmol from the appropriate enaminone intermediate **3a–f** was dissolved in 4 ml glacial acetic acid, then the required amount (0.8 g, 0.6 mmol) of *o-, m-*, *p*-aminobenzoic acid **4a–c** was added to the reaction mixture. Reflux was continued for 3 h, and then the precipitated solid was filtered off while hot, washed with hot 70% ethanol (3 × 2 ml), dried and recrystallized from DMF/methanol mixture (1:4) to yield the target enaminones **5a–q** (Supporting Information).

#### 4.1.4. Synthesis of target 3/4-((3-oxo-3-phenylpropyl)amino)benzoic acids 7a–b

To a hot solution of 3-(dimethylamino)-1-phenylpropan-1-one **6** (0.25 g, 1.41 mmol) in absolute ethanol (12 ml), an equivalent amount of *m*- or *p*-aminobenzoic acid **4b–c** (0.19 g, 1.41 mmol) was added, and the mixture was heated under reflux for 4 h. The formed solid, after cooling, was collected and washed with aq. potassium bicarbonate solution and cold methanol (2 × 3 ml), then recrystallized from isopropanol to get the 3/4-((3-oxo-3-phenylpropyl)amino)benzoic acids **7a–b**, respectively (Supporting Information).

#### 4.1.5. *Synthesis of target carboxylic acids enaminones* 12a–c

Enaminones **12a–c** were prepared *via* the same method described previously for enaminones **5a–q**, utilising (4-aminobenzoyl)glycine **11** instead of aminobenzoic acids **4a–c** (Supporting Information).

### 4.2. Biological evaluation

#### 4.2.1. Ca inhibitory assay

The target carbonic anhydrase inhibitors reported in this work were evaluated for their potential CA-catalysed CO_2_ hydration activities utilising an Applied Photophysics stopped-flow instrument as described previously^31–35^ (Supporting Materials).

## Supplementary Material

Supplemental Material
