# Supplementary material for: Enaminone-based carboxylic acids as novel non-classical carbonic anhydrases inhibitors: design, synthesis and in vitro biological assessment
Source: J Enzyme Inhib Med Chem. 2022 Aug 23;37(1):2256–64. doi: 10.1080/14756366.2022.2114079 (PMC9466612; doi:10.1080/14756366.2022.2114079)
Supplement: Supplemental Material [file IENZ_A_2114079_SM4511.pdf]

Supporting Information

Enaminone-based carboxylic acids as novel non-classical carbonic anhydrases inhibitors: Design, Synthesis and *in vitro* biological assessment

Mahmoud F. Abo-Ashour, Hadia Almahli, Alessandro Bonardia, Amira Khalil, Tarfah Al-Warhi, Sara T. Al-Rashood, Hatem A. Abdel-Aziz, Alessio Nocentini, Claudiu T. Supuran, Wagdy M. Eldehna

* Corresponding authors. E-mail addresses: wagdy2000@gmail.com (W.M. Eldehna), claudiu.supuran@unifi.it (C.T. Supuran).

Tables of Contents

**1.**	**Physical and spectral data for target carboxylic acids**	3-10
**2.**	**Carbonic Anhydrase Inhibition Assay**	11
**3.**	**NMR and HRMS Spectra**	12-96

1. Physical and spectral data for target carboxylic acids

2-((3-Oxo-3-phenylprop-1-en-1-yl)amino)benzoic acid ***5a***.

Buff crystals, m.p. 130-131 °C (yield 75%); ^{1}HNMR (DMSO-d_6) *δ ppm*: 6.14 (d, 1 H, Aroma._H, *J* = 8.4 Hz), 7.06-7.10 (m, 1 H, Aroma._H), 7.41-7.47 (m, 2 H, Aroma._H), 7.49-7.55 (m, 3H, Aroma._H), 7.60 (d, 1 H, Aroma._H, *J* = 8.4 Hz), 7.95-7.97 (m, 2 H, Aroma._H), 7.99-8.01 (m, 1 H, Aroma._H), 13.46 (d, 1 H, NH, *J* = 12.4 Hz), 13.60 (s, 1 H, OH); ^{13}C NMR (DMSO-d_6) *δ ppm*: 91.41, 95.38, 99.44, 110.32, 113.37, 114.90, 115.01, 116.78, 119.47, 121.23, 122.22, 127.65, 127.91, 128.61, 128.88, 129.01, 131.23, 131.65, 132.08, 132.22, 132.56, 133.64, 134.10, 139.42, 142.41, 143.45, 151.95, 154.67 (Aromatic carbons), 169.11, 170.15 (C=O acidic), 187.91, 188.85 (C=O); HRMS (ESI) for $C_{16}H_{14}O_3N$, Cal 268.09682, found 268.09681 $[M+H]^+$; E.A. Cal. for $C_{16}H_{13}NO_3$: C, 71.90; H, 4.90; N, 5.24; found C, 71.72; H, 4.93; N, 5.21.

2-((3-(4-Fluorophenyl)-3-oxoprop-1-en-1-yl)amino)benzoic acid ***5b***.

Yellow crystals, m.p. 230-232 °C (yield 65%); 1H NMR (DMSO-d_6) *δ ppm*: 6.19 (d, 1 H, Aroma._H, *J* = 8.4 Hz), 7.10 (d, 1 H, Aroma._H, *J* = 8.0 Hz), 7.31 (d, 2 H, Aroma._H, *J* = 8.8 Hz), 7.59-7.63 (m, 1 H, Aroma._H), 7.68 (d, 1 H, Aroma._H, *J* = 8.4 Hz), 7.90-7.99 (m, 2 H, Aroma._H), 8.04-8.07 (m, 2 H, Aroma._H), 13.18 (d, 1 H, NH, *J* = 12.4 Hz), 13.48 (s, 1 H, OH); E.A. Cal. for $C_{16}H_{12}FNO_3$: C, 67.37; H, 4.24; N, 4.91; found C, 67.58; H, 4.21; N, 4.96.

2-((3-(4-Chlorophenyl)-3-oxoprop-1-en-1-yl)amino)benzoic acid ***5c***.

Yellow crystals, m.p. 165-166 °C (yield 60%); ^{1}H NMR (DMSO-d6, 400 MHz) *δ ppm*: 6.13 (d, 1 H, Aroma._H, *J* = 8.0 Hz), 7.07 (t, 1 H, Aroma._H, *J* = 7.6 Hz), 7.41-7.52 (m, 1 H, Aroma._H), 7.53-7.56 (m, 2 H, Aroma._H), 7.63 (d, 1 H, Aroma._H, *J* = 8.4 Hz), 7.87 (m, 1 H, Aroma._H), 7.96-7.99 (m, 3H, Aroma._H), 13.43 (d, 1 H, NH, *J* = 12.4 Hz), 13.57 (s, 1 H, OH); E.A. Cal. for $C_{16}H_{12}ClNO_3$: C, 63.69; H, 4.01; N, 4.64; found C, 63.58; H, 4.07; N, 4.60.

2-((3-(4-Nitrophenyl)-3-oxoprop-1-en-1-yl)amino)benzoic acid ***5d***.

Orange crystals, m.p. 200-202 °C (yield 70%); ^{1}H NMR (DMSO-d_6) *δ ppm*: 6.16 (d, 1 H, Aroma._H, *J* = 8.0 Hz), 7.09-7.13 (m, 1 H, Aroma._H), 7.41-7.54 (m, 1 H, Aroma._H), 7.64 (d, 1 H, Aroma._H, *J* = 8.0 Hz), 7.94-8.01 (m, 2 H, Aroma._H), 8.15 (d, 2 H, Aroma._H, *J* = 8.8 Hz), 8.26 (d, 2 H, Aroma._H, *J* = 8.8 Hz), 13.67 (d, 1 H, NH, *J* = 12.8 Hz), 13.93 (s, 1 H, OH);

^{13}C NMR (DMSO-d_6) *δ ppm* : 111.76, 113.76, 114.99, 118.76, 122.26, 123.79, 126.85, 128.77, 134.69, 135.21, 138.16, 142.13, 148.02 (aromatic carbons), 162.07 (hydrazide C=O), 163.10 (isatin C=O); HRMS (ESI) for $C_{16}H_{11}O_5N_2$, Cal 311.06734, found 311.06779 $[M\text{-}H]^+$; E.A. Cal. for $C_{16}H_{12}N_2O_5$: C, 61.54; H, 3.87; N, 8.97; found C, 61.73; H, 3.90; N, 8.91.

2-((3-(Naphthalen-2-yl)-3-oxoprop-1-en-1-yl)amino)benzoic acid ***5e***.

Yellow crystals, m.p. 240-242 °C (yield 66%); ^1H NMR (DMSO-d_6) *δ ppm*: 6.37 (d, 1 H, Aroma._H, *J* = 8.5 Hz), 7.10 (t, 1 H, Aroma._H, *J* = 8.0 Hz), 7.57-7.64 (m, 3H, Aroma._H), 7.70 (d, 1 H, Aroma._H, *J* = 8.5 Hz), 7.93-8.01 (m, 4H, Aroma._H), 8.04-8.06 (m, 1 H, Aroma._H), 8.10 (d, 1 H, Aroma._H, *J* = 8.0 Hz), 8.63 (s, 1 H, Aroma._H), 13.24 (d, 1 H, NH, *J* = 12.5 Hz), 13.41 (s, 1 H, OH); E.A. Cal. for $C_{20}H_{15}NO_3$: C, 75.70; H, 4.76; N, 4.41; found C, 75.98; H, 4.74; N, 4.45.

3-((3-Oxo-3-phenylprop-1-en-1-yl)amino)benzoic acid ***5f***.

Buff crystals, m.p. 200-201 °C (yield 60%); ^1H NMR (DMSO-d_6) *δ ppm*: 6.13 (d, 0.5 H, Aroma._H, *J* = 7.6 Hz), 6.45 (d, 0.5 H, Aroma._H, *J* = 12.4 Hz), 7.27-7.29 (m, 0.5H, Aroma._H), 7.34-7.43 (m, 1.5H, Aroma._H), 7.47-7.57 (m, 3.5H, Aroma._H), 7.63 (d, 0.5 H, Aroma._H, *J* = 7.2 Hz), 7.70-7.79 (m, 1 H, Aroma._H), 7.85-7.98 (m, 2.5H, Aroma._H), 8.14-8.19 (m, 0.5H, Aroma._H), 10.36 (d, 0.5 H, NH, *J* = 8.8 Hz), 12.11 (d, 0.5 H, NH, *J* = 12.8 Hz), 13.07 (s, 1 H, OH); ^{13}C NMR (DMSO-d_6) *δ ppm* : 91.41, 93.79, 98.00, 115.08, 117.09, 118.12, 118.92, 123.67, 124.72, 127.60, 127.63, 127.69, 128.61, 128.96, 129.06, 129.68, 131.23, 131.97, 132.20, 137.96, 138.40, 139.13, 139.91, 140.15, 141.20, 144.80, 146.44, 154.67 (Aromatic carbons), 169.09, 169.15 (C=O acidic), 187.92, 189.98 (C=O); E.A. Cal. for $C_{16}H_{13}NO_3$: C, 71.90; H, 4.90; N, 5.24; found C, 71.82; H, 4.93; N, 5.18.

3-((3-(4-Fluorophenyl)-3-oxoprop-1-en-1-yl)amino)benzoic acid ***5g***.

Yellow crystals, m.p 235-237 °C (yield 70%); ^1H NMR (DMSO-d_6) *δ ppm*: 6.14 (d, 0.5 H, Aroma._H, *J* = 8.0 Hz), 6.43 (d, 0.5 H, Aroma._H, *J* = 12.4 Hz), 7.29-7.34 (m, 2 H, Aroma._H), 7.44-7.50 47 (m, 1.5H, Aroma._H), 7.56-7.67 47 (m, 2 H, Aroma._H), 7.82 (br s, 0.5H, Aroma._H), 7.93-8.00 (m, 1.5H, Aroma._H), 8.03 (dd, 1 H, Aroma._H, *J* = 12.0 and 8.0 Hz), 8.13 (overlapped dd, 0.5H, Aroma._H, *J* = 12.0 and 12.0 Hz), 10.28 (d, 0.5 H, NH, *J* = 12.8 Hz), 12.03 (d, 0.5 H, NH, *J* = 12.4 Hz), 13.09 (s, 1 H, OH); ^{13}C NMR (DMSO-d_6) *δ ppm* : 94.03,

98.29, 115.78, 115.88, 116.00, 116.10, 117.20, 120.00, 121.16, 123.54, 124.57, 130.37, 130.40, 130.49, 132.63, 132.81, 135.59, 135.62, 136.31, 136.34, 140.86, 141.75, 144.63, 146.36, 163.38, 163.54, 165.86, 166.03 (Aromatic carbons), 167.39, 167.44 (C=O acidic), 186.55, 188.69 (C=O); E.A. Cal. for $C_{16}H_{12}FNO_3$: C, 67.37; H, 4.24; N, 4.91; found C, 67.49; H, 4.21; N, 4.97.

3-((3-(4-Chlorophenyl)-3-oxoprop-1-en-1-yl)amino)benzoic acid ***5h****.*

Yellow crystals, m.p. 240-242 °C (yield 85%); ^{1}H NMR (DMSO-d_6) *δ ppm*: 6.15 (d, 0.5 H, Aroma._H, *J* = 8.0 Hz), 6.41 (d, 0.5 H, Aroma._H, *J* = 12.4 Hz), 7.45-7.51 (m, 1.5 H, Aroma._H), 7.55-7.59 (m, 2.5 H, Aroma._H), 7.61-7.67 (m, 1.5 H, Aroma._H), 7.83 (br s, 0.5H, Aroma._H), 7.88 (d, 1 H, Aroma._H, *J* = 8.8 Hz), 7.98-8.03 (m, 1.5 H, Aroma._H), 8.13-8.20 (m, 0.5 H, Aroma._H), 10.32 (d, 0.5 H, NH, *J* = 12.8 Hz), 12.05 (d, 0.5 H, NH, *J* = 12.4 Hz), 13.01 (s, 1 H, OH); ^{13}C NMR (DMSO-d_6) *δ ppm :* 94.03, 98.27, 116.86, 117.30, 120.04, 121.27, 123.64, 124.69, 125.69, 129.09, 129.17, 129.57, 129.64, 130.43, 130.52, 132.63, 132.82, 133.16, 136.86, 137.10, 137.16, 137.71, 138.45, 140.81, 141.67, 142.62, 144.94, 146.70 (Aromatic Carbons), 167.38, 167.43 (C=O acidic), 186.71, 188.68 (C=O); HRMS (ESI) for $C_{16}H_{13}O_3N^{35}Cl$, Cal 302.05894, found 302.05794 $[M+H]^+$; E.A. Cal. for $C_{16}H_{12}ClNO_3$: C, 63.69; H, 4.01; N, 4.64; found C, 63.76; H, 3.97; N, 4.49.

3-((3-(4-Bromophenyl)-3-oxoprop-1-en-1-yl)amino)benzoic acid ***5i****.*

Yellow crystals, m.p. 257-260 °C (yield 71%); ^{1}H NMR (DMSO-d_6) *δ ppm*: 6.14 (d, 0.5 H, Aroma._H, *J* = 8.0 Hz), 6.40 (d, 0.5 H, Aroma._H, *J* = 12.4 Hz), 7.45-7.47 (m, 1 H, Aroma._H), 7.48-7.51 (m, 0.5H, Aroma._H), 7.57-7.61 (m, 1 H, Aroma._H), 7.64-7.67 (m, 1 H, Aroma._H), 7.69-7.72 (m, 2 H, Aroma._H), 7.79-7.84 (m, 1.5H, Aroma._H), 7.90-7.93 (m, 1 H, Aroma._H), 7.99 (dd, 0.5H, Aroma._H, *J* = 12.0 and 8.0 Hz), 8.14 (overlapped dd, 0.5H, Aroma._H, *J* = 12.0 and 12.0 Hz), 10.33 (d, 0.5 H, NH, *J* = 12.8 Hz), 12.05 (d, 0.5 H, NH, *J* = 12.4 Hz), 13.01 (s, 1 H, OH); ^{13}C NMR (DMSO-d_6) *δ ppm:* 93.99, 98.24, 115.94, 116.84, 117.30, 120.06, 121.28, 123.66, 124.71, 125.87, 126.21, 129.74, 129.82, 130.43, 130.52, 132.04, 132.12, 132.65, 132.84, 136.13, 136.74, 138.05, 138.80, 140.80, 141.66, 142.37, 144.97, 146.74 (Aromatic Carbons), 167.38, 168.44 (C=O acidic), 186.86, 188.81 (C=O); E.A. Cal. for $C_{16}H_{12}BrNO_3$: C, 55.51; H, 3.49; N, 4.05; found C, 55.41; H, 3.53; N, 4.00.

3-((3-(4-Nitrophenyl)-3-oxoprop-1-en-1-yl)amino)benzoic acid ***5j****.*

Yellow crystals, m.p. 270-271 °C (yield 70%); ^{1}H NMR (DMSO-d_6) *δ ppm*: 6.22 (d, 0.5 H, Aroma._H, *J* = 8.0 Hz), 6.43 (d, 0.5 H, Aroma._H, *J* = 12.4 Hz), 7.45-7.53 (m, 1.5H, Aroma._H), 7.58-7.62 (m, 0.5H, Aroma._H), 7.65-7.70 (m, 1.5H, Aroma._H), 7.88 (br s, 0.5H, Aroma._H), 8.07-8.14 (m, 1.5H, Aroma._H), 8.19-8.25 (m, 1.5H, Aroma._H), 8.32-8.35 (m, 2 H, Aroma._H), 10.51 (d, 0.5 H, NH, *J* = 12.8 Hz), 12.14 (d, 0.5 H, NH, *J* = 12.8 Hz), 13.10 (s, 1 H, OH); HRMS (ESI) for $C_{16}H_{11}O_5N_2$, Cal 311.06734, found 311.06783 $[M-H]^+$; E.A. Cal. for $C_{16}H_{12}N_2O_5$: C, 61.54; H, 3.87; N, 8.97; found C, 61.70; H, 3.91; N, 8.92.

3-((3-(Naphthalen-2-yl)-3-oxoprop-1-en-1-yl)amino)benzoic acid ***5k***.

Yellow powder, m.p. 250-251 °C (yield 80%); ^{1}H NMR (DMSO-d_6) *δ ppm* 6.34 (d, 0.5 H, Aroma._H, *J* = 8.5 Hz), 6.62 (d, 0.5 H, Aroma._H, *J* = 12.5 Hz), 7.45-7.50 (m, 1.5H, Aroma._H), 7.59-7.64 (m, 3.5H, Aroma._H), 7.69 (s, 0.5H, Aroma._H), 7.84 (s, 0.5H, Aroma._H), 7.95-8.06 (m, 3.5H, Aroma._H), 8.10 (d, 0.5 H, Aroma._H, *J* = 8.0 Hz), 8.18-8.23 (m, 0.5H, Aroma._H), 8.48 (s, 0.5H, Aroma._H), 8.62 (s, 0.5H, Aroma._H), 10.32 (d, 0.5H, NH, *J* = 13.0 Hz), 12.13 (d, 0.5H, NH, *J* = 12.0 Hz), 13.11 (s, 1 H, OH); E.A. Cal. for $C_{20}H_{15}NO_3$: C, 75.70; H, 4.76; N, 4.41; found C, 75.79; H, 4.73; N, 4.38.

4-((3-Oxo-3-phenylprop-1-en-1-yl)amino)benzoic acid ***5l***.

Yellow crystals, m.p. 278-280 °C (yield 77%); ^{1}H NMR (DMSO-d_6) *δ ppm*: 6.22 (d, 0.5 H, Aroma._H, *J* = 8.0 Hz), 6.53 (d, 0.5 H, Aroma._H, *J* = 12.4 Hz), 7.23 (d, 1 H, Aroma._H, *J* = 8.8 Hz), 7.42 (d, 1 H, Aroma._H, *J* = 8.8 Hz), 7.49 (t, 2 H, Aroma._H, *J* = 8.8 Hz), 7.57-7.59 (m, 1 H, Aroma._H), 7.88-7.93 (m, 3H, Aroma._H), 7.96-8.01 (m, 1.5H, Aroma._H), 8.16 (t, 0.5H, Aroma._H, *J* = 12.8 Hz), 10.41 (d, 0.5H, NH, *J* = 12.8 Hz), 12.05 (d, 0.5H, NH, *J* = 12.0 Hz), 12.73 (s, 1 H, OH); ^{13}C NMR (DMSO-d_6) *δ ppm:* 95.18, 99.93, 115.29, 116.20, 124.36, 125.52, 127.77, 127.80, 128.47, 129.06, 129.13, 131.62, 131.79, 132.04, 132.28, 132.54, 138.80, 139.57, 140.58, 142.53, 143.68, 144.37, 145.32, 145.39 (Aromatic carbons), 167.29, 167.39 (C=O acidic), 188.10, 190.47 (C=O); HRMS (ESI) for $C_{16}H_{14}O_3N$, Cal 268.09682, found 268.09680 $[M+H]^+$; E.A. Cal. for $C_{16}H_{13}NO_3$: C, 71.90; H, 4.90; N, 5.24; found C, 71.81; H, 4.93; N, 5.20.

4-((3-(4-Fluorophenyl)-3-oxoprop-1-en-1-yl)amino)benzoic acid ***5m***.

Yellow crystals, m.p. 285-287 °C (yield 70%); ^{1}H NMR (DMSO-d_6) δ *ppm*: 6.21 (d, 0.5 H, Aroma._H, *J* = 8.4 Hz), 6.49 (d, 0.5 H, Aroma._H, *J* = 12.8 Hz), 7.23 (d, 1 H, Aroma._H, *J* = 8.8 Hz), 7.31 (t, 2 H, Aroma._H, *J* = 8.8 Hz), 7.43 (d, 1 H, Aroma._H, *J* = 8.8 Hz), 7.88-7.92 (m, 2 H, Aroma._H), 7.94-8.01(m, 1.5H, Aroma._H), 8.05-8.09 (m, 1 H, Aroma._H), 8.15-8.22 (m, 0.5H, Aroma._H), 10.42 (d, 0.5H, NH, *J* = 12.8 Hz), 12.01 (d, 0.5H, NH, *J* = 12.4 Hz), 12.73 (s, 1 H, OH); ^{13}C NMR (DMSO-d_6) δ *ppm* : 94.95, 99.60, 115.35, 115.87, 115.96, 116.08, 116.18, 116.25, 124.43, 125.57, 130.48, 130.52, 130.57, 130.61, 131.60, 131.77, 135.42, 136.13, 136.16, 143.88, 144.32, 145.33, 145.51, 163.46, 163.66, 165.94 (Aromatic carbons), 167.28, 167.37 (C=O acidic), 186.66, 189.01 (C=O); HRMS (ESI) for $C_{16}H_{13}O_3NF$, Cal 286.08849, found 286.08745 [M+H]$^+$; E.A. Cal. for $C_{16}H_{12}FNO_3$: C, 67.37; H, 4.24; N, 4.91; found C, 67.49; H, 4.21; N, 4.96.

4-((3-(4-Chlorophenyl)-3-oxoprop-1-en-1-yl)amino)benzoic acid ***5n***.

Yellow crystals, m.p. over 300 °C (yield 67%); ^{1}H NMR (DMSO-d_6) δ *ppm*: 6.22 (d, 0.5 H, Aroma._H, *J* = 8.0 Hz), 6.48 (d, 0.5 H, Aroma._H, *J* = 12.4 Hz), 7.24 (d, 1 H, Aroma._H, *J* = 8.8 Hz), 7.44 (d, 1 H, Aroma._H, *J* = 8.8 Hz), 7.56 (d, 2 H, Aroma._H, *J* = 8.4 Hz), 7.88-7.93 (m, 3H, Aroma._H), 7.99-8.04 (m, 1.5H, Aroma._H), 8.16-8.23 (m, 0.5H, Aroma._H), 10.46 (d, 0.5H, NH, *J* = 12.8 Hz), 12.03 (d, 0.5H, NH, *J* = 12.4 Hz), 12.75 (s, 1 H, OH); ^{13}C NMR (DMSO-d_6) δ *ppm*: 94.94, 99.57, 115.44, 116.36, 124.54, 125.69, 129.15, 129.22, 129.68, 129.71, 131.60, 131.77, 134.43, 136.84, 137.03, 137.39, 137.49, 137.57, 138.25, 138.48, 138.82, 139.27, 144.21, 144.25, 145.24, 145.85, 147.14, 149.42 (Aromatic carbons), 167.27, 167.36 (C=O acidic), 186.86, 189.03 (C=O); E.A. Cal. for $C_{16}H_{12}ClNO_3$: C, 63.69; H, 4.01; N, 4.64; found C, 63.88; H, 4.04; N, 4.67.

4-((3-(4-Bromophenyl)-3-oxoprop-1-en-1-yl)amino)benzoic acid ***5o***.

Yellow crystals, m.p. over 300 °C (yield 72%); ^{1}H NMR (DMSO-d_6) δ *ppm*: 6.21 (d, 0.5 H, Aroma._H, *J* = 8.0 Hz), 6.47 (d, 0.5 H, Aroma._H, *J* = 12.4 Hz), 7.24 (d, 1 H, Aroma._H, *J* = 8.8 Hz), 7.44 (d, 1 H, Aroma._H, *J* = 8.8 Hz), 7.71 (d, 2 H, Aroma._H, *J* = 8.0 Hz), 7.81 (d, 1 H, Aroma._H, *J* = 8.8 Hz), 7.88-7.94 (m, 3H, Aroma._H), 8.00 (dd, 0.5H, Aroma._H, *J* = 12.0 Hz, 8.0 Hz), 8.17 (overlapped dd, 0.5H, Aroma._H, *J* = 12.0 and 12.0 Hz), 10.46 (d, 0.5H, NH, *J* = 12.8 Hz), 12.03 (d, 0.5H, NH, *J* = 12.4 Hz), 12.74 (s, 1 H, OH); ^{13}C NMR (DMSO-d_6) δ *ppm* : 94.90, 95.67, 99.55, 104.13, 115.45, 116.37, 124.54, 125.69, 126.07, 126.48, 129.84, 129.88,

130.69, 131.60, 131.77, 131.82, 132.10, 132.17, 132.32, 133.57, 134.11, 137.84, 138.60, 144.23, 144.25, 145.24, 145.53, 145.88 (Aromatic Carbons), 167.26, 167.35 (C=O acidic), 187.01, 189.17 (C=O); HRMS (ESI) for $C_{16}H_{13}O_3N^{79}Br$, Cal 346.00843, found 346.00742 $[M+H]^+$; E.A. Cal. for $C_{16}H_{12}BrNO_3$: C, 55.51; H, 3.49; N, 4.05; found C, 55.34; H, 3.51; N, 4.09.

4-((3-(4-Nitrophenyl)-3-oxoprop-1-en-1-yl)amino)benzoic acid ***5p****.*

Yellow crystals, m.p. over 300 °C (yield 56%); ^{1}H NMR (DMSO-d_6) δ *ppm*: 6.28 (d, 0.33 H, Aroma._H, J = 8.0 Hz), 6.49 (d, 0.66 H, Aroma._H, J = 12.4 Hz), 7.27 (d, 1 H, Aroma._H, J = 8.8 Hz), 7.48 (d, 1 H, Aroma._H, J = 8.8 Hz), 7.89-7.94 (m, 2 H, Aroma._H), 8.07-8.13 (m, 1.66 H, Aroma._H), 8.20-8.27 (m, 1.33 H, Aroma._H), 8.32-8.35 (m, 2 H, Aroma._H), 10.59 (d, 0.66 H, NH, J = 12.8 Hz), 12.01 (d, 0.66 H, NH, J = 12.4 Hz), 12.72 (s, 1 H, OH); ^{13}C NMR (DMSO-d_6) δ *ppm*: 95.30, 99.92, 115.75, 116.73, 124.30, 124.99, 129.11, 131.58, 131.74, 143.97, 144.97, 145.30, 146.96, 149.52 (Aromatic carbons), 167.31 (C=O acidic), 186.68 (C=O); E.A. Cal. for $C_{16}H_{12}N_2O_5$: C, 61.54; H, 3.87; N, 8.97; found C, 61.70; H, 3.90; N, 8.99.

4-((3-(Naphthalen-2-yl)-3-oxoprop-1-en-1-yl)amino)benzoic acid ***5q****.*

Yellow crystals, m.p. over 300 °C (yield 75%); ^{1}H NMR (DMSO-d_6) δ *ppm*: 6.41 (d, 0.5 H, Aroma._H, J = 8.5 Hz), 6.69 (d, 0.5 H, Aroma._H, J = 12.5 Hz), 7.24 (d, 1 H, Aroma._H, J = 9.0 Hz), 7.44 (d, 1 H, Aroma._H, J = 8.5 Hz), 7.59-7.63 (m, 2 H, Aroma._H), 7.88-7.92 (m, 2 H, Aroma._H), 7.97-8.08 (m, 3.5H, Aroma._H), 8.10 (d, 1 H, Aroma._H, J = 8.5 Hz), 8.20-8.25 (m, 0.5H, Aroma._H), 8.49 (s, 0.5H, Aroma._H), 8.65 (s, 0.5H, Aroma._H), 10.44 (d, 0.5H, NH, J = 13.0 Hz), 12.10 (d, 0.5H, NH, J = 12.5 Hz), 12.71 (s, 1 H, OH); E.A. Cal. for $C_{20}H_{15}NO_3$: C, 75.70; H, 4.76; N, 4.41; found C, 75.54; H, 4.79; N, 4.44.

3-((3-Oxo-3-phenylpropyl)amino)benzoic acid ***7a****.*

White crystals, m.p. 218-219 °C (yield 70%); ^{1}H NMR (DMSO-d_6) δ *ppm*: 3.30 (t, 2 H, C=O-C$\underline{H}_2$-, J = 6.4 Hz), 3.41 (d, 2 H, NH-C$\underline{H}_2$-, J = 6.8 Hz), 5.92 (s, 1 H, NH), 6.80 (d, 1 H, Aroma._H, J = 8.8 Hz), 7.13-7.21 (m, 3H, Aroma._H), 7.51 (t, 2 H, Aroma._H, J = 8.0 Hz), 7.62 (t, 1 H, Aroma._H, J = 8.8 Hz), 7.97 (d, 2 H, Aroma._H, J = 8.4 Hz), 12.66 (s, 1 H, OH); ^{13}C NMR (DMSO-d_6) δ *ppm*: 37.99 (NH-$\underline{C}H_2$), 38.51 (C=O$\underline{C}H_2$), 112.94, 116.63, 117.16, 128.38, 129.20, 129.51, 131.93, 133.69, 137.14, 149.20 (Aromatic carbons), 168.37 (C=O

acidic), 199.16 (C=O); HRMS (ESI) for $C_{16}H_{16}O_3N$, Cal 270.11247, found 270.11248 $[M+H]^+$; E.A. Cal. for $C_{16}H_{15}NO_3$: C, 71.36; H, 5.61; N, 5.20; found C, 71.26; H, 5.64; N, 5.25.

4-((3-Oxo-3-phenylpropyl)amino)benzoic acid ***7b***.

White crystals, m.p. 178-180 °C (yield 70%); ^{1}H NMR (DMSO-d_6) *δ ppm*: 3.32 (t, 2 H, C=O-C<u>H</u>$_2$-, *J* = 6.4 Hz), 3.45 (t, 2 H, NH-C<u>H</u>$_2$-, *J* = 6.0 Hz), 6.46-6.49 (m, 1 H, Aroma._H), 6.59 (d, 2 H, Aroma._H, *J* = 8.8 Hz), 7.52 (t, 2 H, Aroma._H, *J* = 7.6 Hz), 7.63-7.69 (m, 3H, 2 H, Aroma._H, 1 H, NH), 7.97 (d, 2 H, Aroma._H, *J* = 7.2 Hz), 12.00 (s, 1 H, OH); ^{13}C NMR (DMSO-d_6) *δ ppm*: 37.86 (NH-<u>C</u>H$_2$), 37.97 (C=O<u>C</u>H$_2$), 111.28, 117.41, 128.38, 129.20, 131.67, 133.71, 137.09, 152.85 (Aromatic carbons), 167.99 (C=O acidic), 198.96 (C=O); HRMS (ESI) for $C_{16}H_{16}O_3N$, Cal 270.11247, found 270.11241 $[M+H]^+$; E.A. Cal. for $C_{16}H_{15}NO_3$: C, 71.36; H, 5.61; N, 5.20; found C, 71.25; H, 5.65; N, 5.24.

4-((3-Oxo-3-phenylprop-1-en-1-yl)amino)benzoylglycine ***12a***.

Yellow crystals, m.p. over 300 °C (yield 70%); ^{1}H NMR (DMSO-d_6) *δ ppm*: 3.91 (t, 2 H, NH-C<u>H</u>$_2$-, *J* = 6.0 Hz), 6.20 (d, 0.5 H, Aroma._H, *J* = 8.0 Hz), 6.51 (d, 0.5 H, Aroma._H, *J* = 12.4 Hz), 7.23 (d, 1 H, Aroma._H, *J* = 8.8 Hz), 7.43 (d, 1 H, Aroma._H, *J* = 8.8 Hz), 7.49-7.53 (m, 2 H, Aroma._H), 7.55-7.59 (m, 1 H, Aroma._H), 7.86-7.91 (m, 3H, Aroma._H), 7.96-8.01 (m, 1.5H, Aroma._H), 8.17 (overlapped dd, 0.5H, Aroma._H, *J* = 12.4 and 12.8 Hz), 8.71, 8.76 (t, 1 H, amid NH, *J* = 6.0 Hz), 10.32 (d, 0.5H, NH, *J* = 12.8 Hz), 12.06 (d, 0.5H, NH, *J* = 12.4 Hz), 12.58 (s, 1 H, OH); ^{13}C NMR (DMSO-d_6) *δ ppm*: 41.67 (NH-<u>C</u>H$_2$), 94.81, 99.47, 115.28, 116.11, 120.94, 120.97, 125.12, 127.72, 127.76, 128.74, 129.04, 129.12, 129.19, 129.49, 129.59, 130.52, 131.64, 132.19, 132.45, 134.96, 138.92, 139.71, 142.55, 143.17, 144.03, 144.17, 144.58, 145.58, 166.23, 166.34 (C=O amide), 171.92, 171.96 (C=O acidic), 188.06, 190.37 (C=O); HRMS (ESI) for $C_{18}H_{17}O_4N_2$, Cal 325.11883, found 325.11832 $[M+H]^+$; E.A. Cal. for $C_{18}H_{16}N_2O_4$: C, 66.66; H, 4.97; N, 8.64; found C, 66.87; H, 4.95; N, 8.58.

4-((3-(4-Bromophenyl)-3-oxoprop-1-en-1-yl)amino)benzoylglycine ***12b***.

Yellow crystals, m.p. over 300 °C (yield 70%); ^{1}H NMR (DMSO-d_6) *δ ppm*: 3.90 (t, 2 H, NH-C<u>H</u>$_2$-, *J* = 5.6 Hz), 6.18 (d, 0.5 H, Aroma._H, *J* = 8.0 Hz), 6.45 (d, 0.5 H, Aroma._H, *J* = 12.4 Hz), 7.24 (d, 1 H, Aroma._H, *J* = 8.8 Hz), 7.44 (d, 1 H, Aroma._H, *J* = 8.8 Hz), 7.70 (d, 2 H, Aroma._H, *J* = 8.4 Hz), 7.81 (d, 1 H, Aroma._H, *J* = 8.8 Hz), 7.85-7.93 (m, 3H, Aroma._H),

7.99-8.04 (m, 0.5H, Aroma._H), 8.18 (overlapped dd, 0.5H, Aroma._H, *J* =12.8 and 12.8 Hz), 8.70, 8.76 (t, 1 H, amid NH, *J* = 6.0 Hz), 10.38 (d, 0.5H, NH, *J* = 13.2 Hz), 12.04 (d, 0.5H, NH, *J* = 12.4 Hz), 12.54 (s, 1 H, OH); ^{13}C NMR (DMSO-d_6) *δ ppm*: 41.67 (NH-$\underline{C}H_2$), 94.52, 99.07, 115.43, 116.27, 125.97, 126.36, 127.90, 128.92, 129.47, 129.57, 129.80, 129.84, 130.31, 132.07, 132.14, 132.49, 134.90, 135.68, 137.38, 137.95, 138.74, 140.60, 143.03, 143.38, 144.01, 144.57, 146.13 (Aromatic carbons), 166.19, 166.29 (C=O amide), 171.89, 171.93 (C=O acidic), 186.94, 189.05 (C=O); HRMS (ESI) for $C_{18}H_{16}O_4N_2{}^{79}Br$, Cal 403.02934, found 403.02878 $[M+H]^+$; E.A. Cal. for $C_{18}H_{15}BrN_2O_4$: C, 53.62; H, 3.75; N, 6.95; found C, 53.73; H, 3.77; N, 6.90.

4-((3-(Naphthalen-2-yl)-3-oxoprop-1-en-1-yl)amino)benzoylglycine ***12c***.

Yellow crystals, m.p over 300 °C (yield 70%); ^{1}H NMR (DMSO-d_6) *δ ppm*: 3.89 (t, 2 H, NH-C$\underline{H_2}$-, *J* = 7.0 Hz), 6.39 (d, 0.5 H, Aroma._H, *J* = 7.5 Hz), 6.67 (d, 0.5 H, Aroma._H, *J* = 12.5 Hz), 7.24 (d, 1 H, Aroma._H, *J* = 8.0 Hz), 7.45 (d, 1 H, Aroma._H, *J* = 9.0 Hz), 7.53-7.64 (m, 2 H, Aroma._H), 7.84-7.89 (m, 2 H, Aroma._H), 7.96-8.06 (m, 3H, Aroma._H), 8.10 (d, 1 H, Aroma._H, *J* = 7.5 Hz), 8.22-8.27 (m, 0.5H, Aroma._H), 8.49 (s, 0.5H, Aroma._H), 8.65 (s, 0.5H, Aroma._H), 8.70 (t, 0.5H, Aroma._H, *J* = 6.5 Hz), 8.76 (t, 1 H, amid NH, *J* = 6.0 Hz), 10.37 (d, 0.5H, NH, *J* = 13.5 Hz), 12.12 (d, 0.5H, NH, *J* = 12.5 Hz), 12.58 (s, 1 H, OH); E.A. Cal. for $C_{22}H_{18}N_2O_4$: C, 70.58; H, 4.85; N, 7.48; found C, 70.67; H, 4.82; N, 7.43.

2. Carbonic anhydrase inhibition assay

The carbonic anhydrase catalyzed CO_2 hydration actions for the carboxylic acid derivatives (**5a-q, 7a-b and 12a-c**) were assayed utilizing an instrument of Applied Photophysics stopped-flow [3], as described previously. The enzymes are recombinant proteins prepared in our lab. Phenol red (at a concentration of 0.2 mM) has been used as indicator, working at the absorbance maximum of 557 nm, with 20 mM Hepes (pH 7.5) as buffer, and 20 mM Na_2SO_4 (for maintaining constant the ionic strength), following the initial rates of the CA-catalyzed CO_2 hydration reaction for a period of 10-100 s. The CO_2 concentrations ranged from 1.7 to 17 mM for the determination of the kinetic parameters and inhibition constants. For each inhibitor at least six traces of the initial 5-10% of the reaction have been used for determining the initial velocity. The uncatalyzed rates were determined in the same manner and subtracted from the total observed rates. Stock solutions of inhibitor (0.1 mM) were prepared in distilled-deionized water and dilutions up to 0.01 nM were done thereafter with the assay buffer. Inhibitor and enzyme solutions were preincubated together for 15 min at room temperature prior to assay, in order to allow for the formation of the E-I complex. The inhibition constants were obtained by non-linear least-squares methods using PRISM 3 and the Cheng-Prusoff equation, and represent the mean from at least three different determinations.

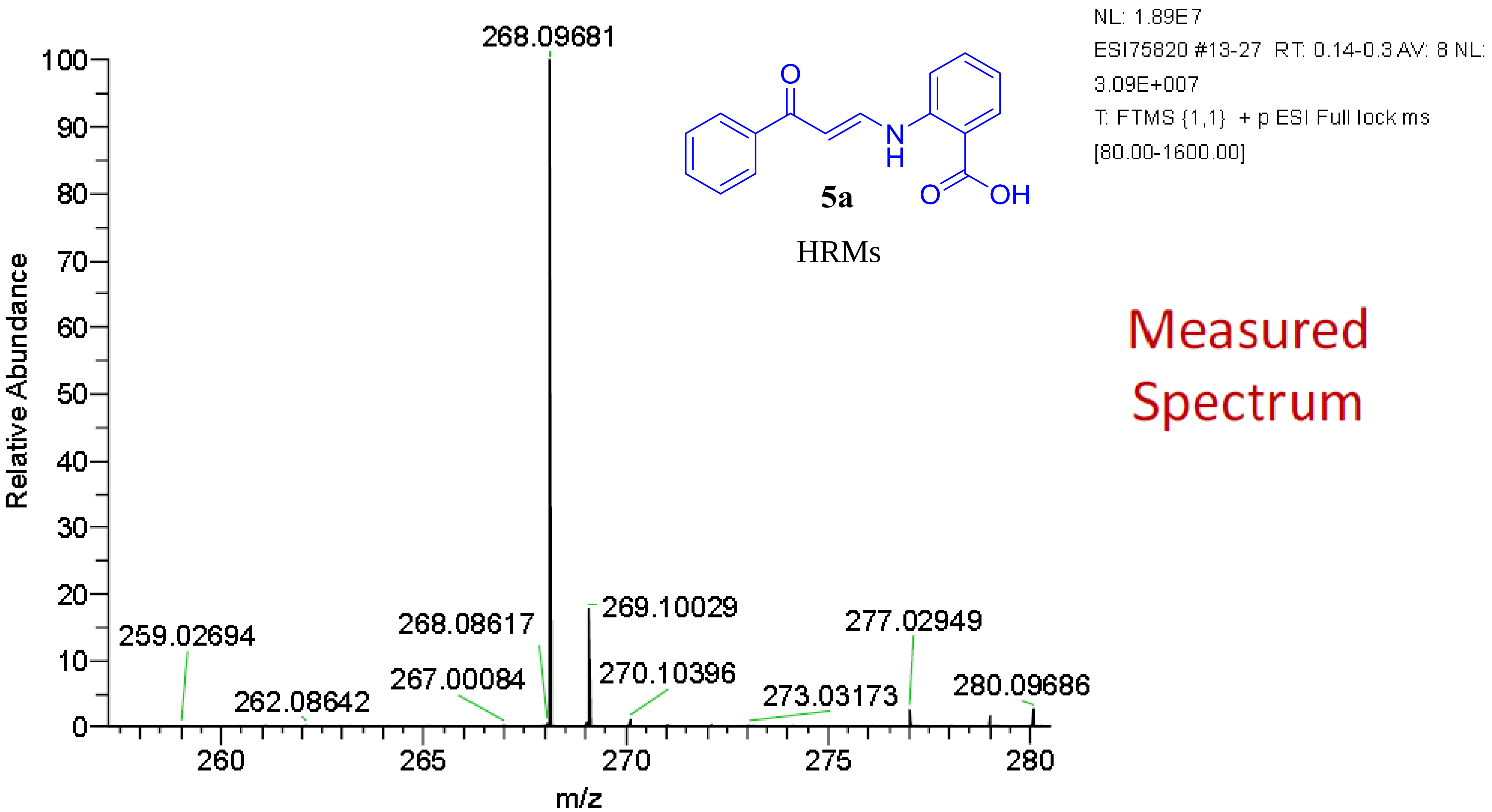
NL: 1.89E7
ESI75820 #13-27 RT: 0.14-0.3 AV: 8 NL: 3.09E+007
T: FTMS {1,1} + p ESI Full lock ms [80.00-1600.00]
5a
HRMs
Measured Spectrum
Relative Abundance
m/z
268.09681
269.10029
268.08617
259.02694
262.08642
267.00084
270.10396
273.03173
277.02949
280.09686

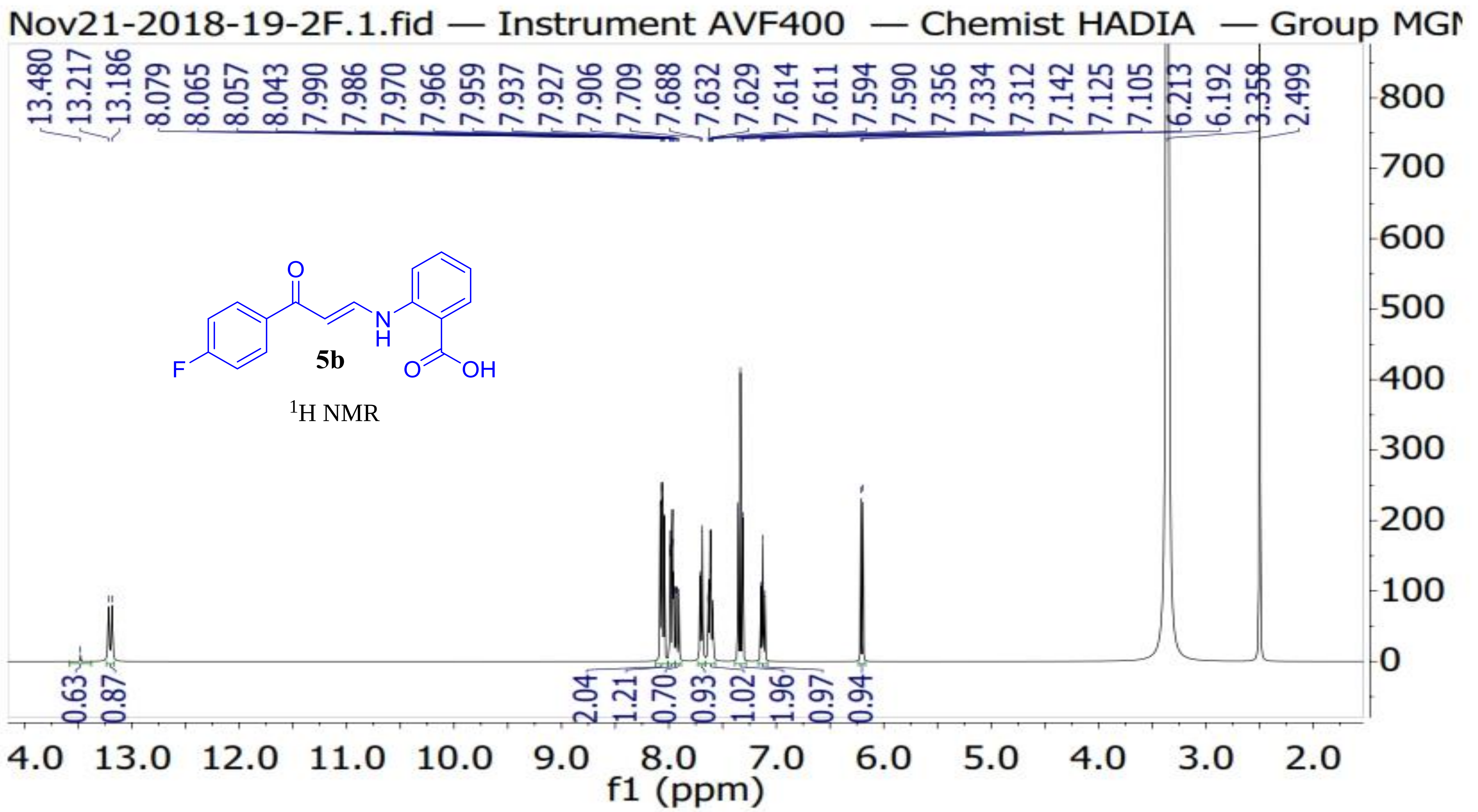
Nov21-2018-19-2F.1.fid — Instrument AVF400 — Chemist HADIA — Group MGN
13.480
13.217
13.186
8.079
8.065
8.057
8.043
7.990
7.986
7.970
7.966
7.959
7.937
7.927
7.906
7.709
7.688
7.632
7.629
7.614
7.611
7.594
7.590
7.356
7.334
7.312
7.142
7.125
7.105
6.213
6.192
3.358
2.499
O
N
H
F
5b
O
OH
1H NMR
800
700
600
500
400
300
200
100
0
0.63
0.87
2.04
1.21
0.70
0.93
1.02
1.96
0.97
0.94
4.0
13.0
12.0
11.0
10.0
9.0
8.0
7.0
6.0
5.0
4.0
3.0
2.0
f1 (ppm)

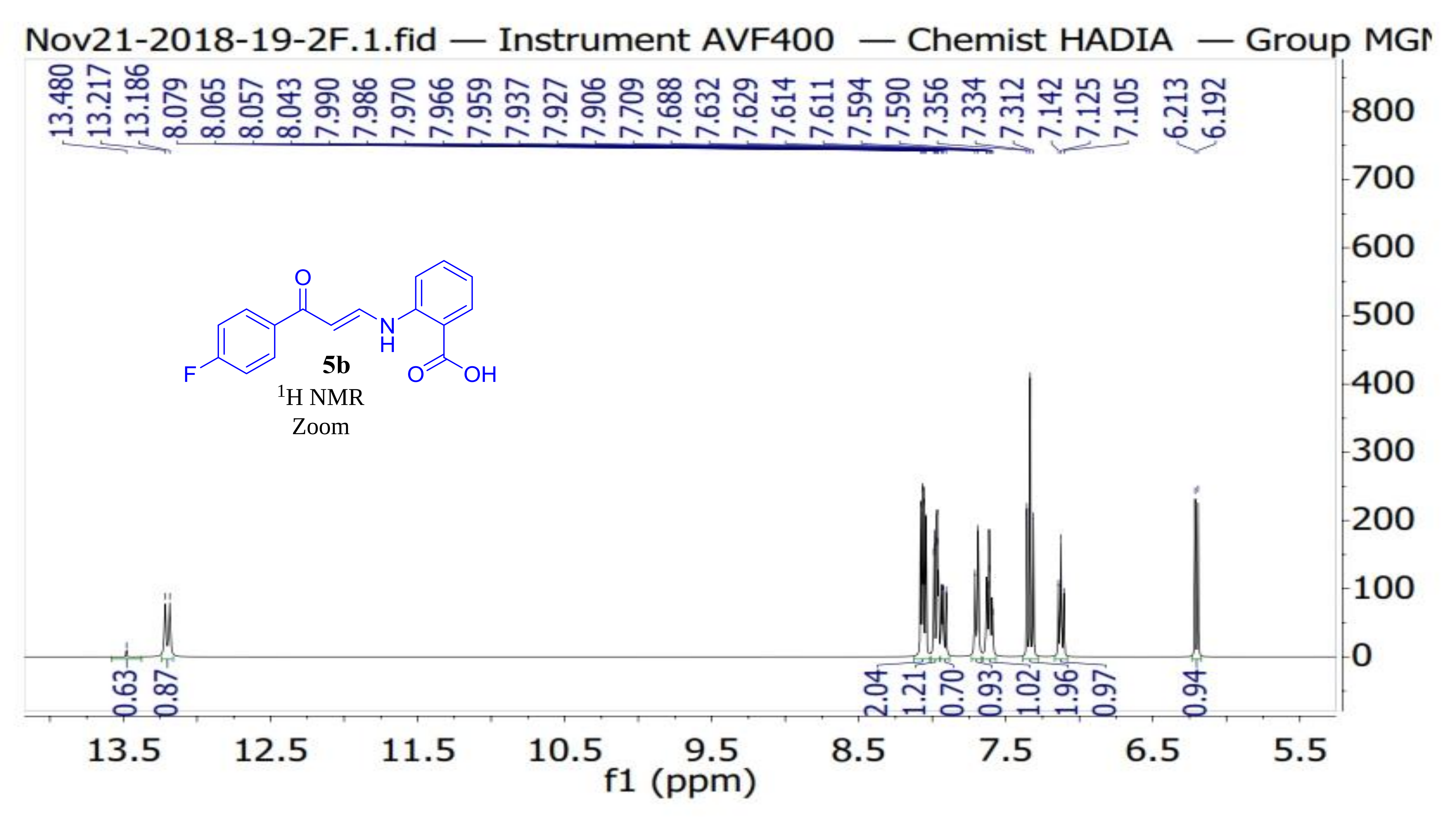

Nov21-2018-19-2F.1.fid — Instrument AVF400 — Chemist HADIA — Group MGN
13.480
13.217
13.186
8.079
8.065
8.057
8.043
7.990
7.986
7.970
7.966
7.959
7.937
7.927
7.906
7.709
7.688
7.632
7.629
7.614
7.611
7.594
7.590
7.356
7.334
7.312
7.142
7.125
7.105
6.213
6.192
O
N
H
O
OH
F
5b
1H NMR
Zoom
800
700
600
500
400
300
200
100
0
0.63
0.87
2.04
1.21
0.70
0.93
1.02
1.96
0.97
0.94
13.5
12.5
11.5
10.5
9.5
8.5
7.5
6.5
5.5
f1 (ppm)

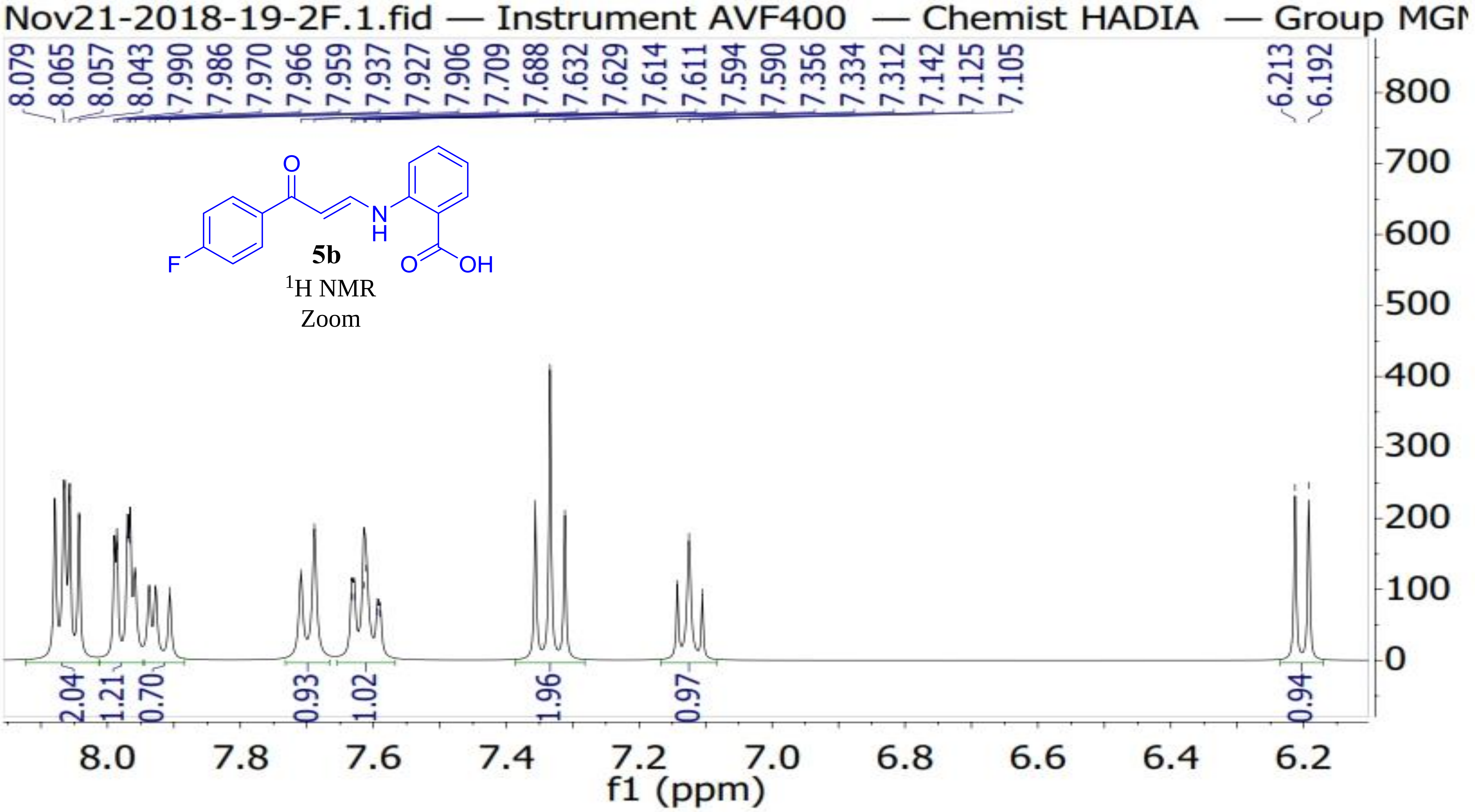

Nov21-2018-19-2F.1.fid — Instrument AVF400 — Chemist HADIA — Group MG
8.079 8.065 8.057 8.043 7.990 7.986 7.970 7.966 7.959 7.937 7.927 7.906 7.709 7.688 7.632 7.629 7.614 7.611 7.594 7.590 7.356 7.334 7.312 7.142 7.125 7.105
6.213 6.192
O
N
H
F
O
OH
5b
1H NMR
Zoom
2.04 1.21 0.70 0.93 1.02 1.96 0.97 0.94
8.0 7.8 7.6 7.4 7.2 7.0 6.8 6.6 6.4 6.2
f1 (ppm)
800 700 600 500 400 300 200 100 0

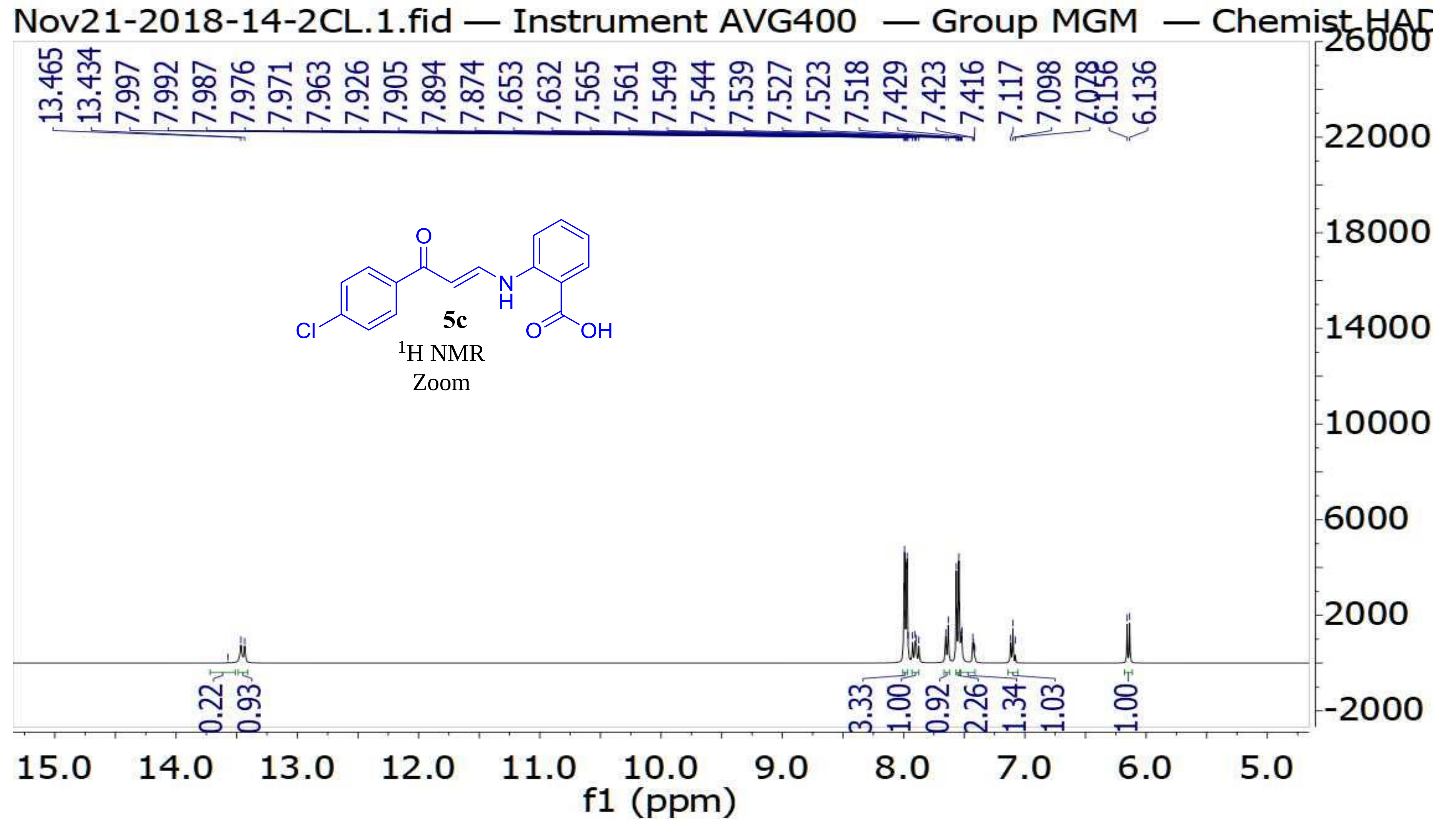

Nov21-2018-14-2CL.1.fid — Instrument AVG400 — Group MGM — Chemist HAD
13.465
13.434
7.997
7.992
7.987
7.976
7.971
7.963
7.926
7.905
7.894
7.874
7.653
7.632
7.565
7.561
7.549
7.544
7.539
7.527
7.523
7.518
7.429
7.423
7.416
7.117
7.098
7.078
6.156
6.136
O
N
H
Cl
O
OH
5c
¹H NMR
Zoom
0.22
0.93
3.33
1.00
0.92
2.26
1.34
1.03
1.00
26000
22000
18000
14000
10000
6000
2000
-2000
15.0
14.0
13.0
12.0
11.0
10.0
9.0
8.0
7.0
6.0
5.0
f1 (ppm)

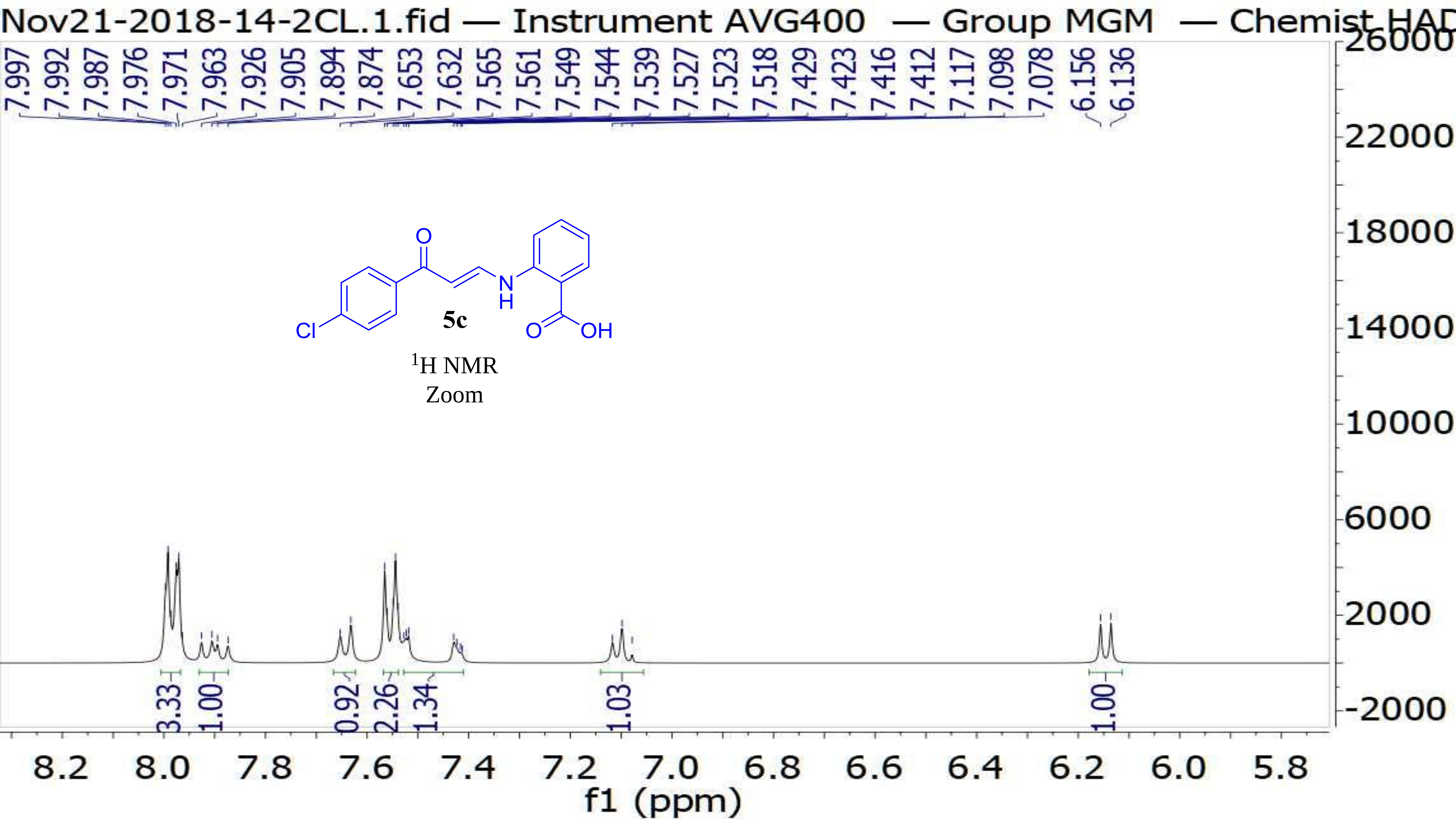
Nov21-2018-14-2CL.1.fid — Instrument AVG400 — Group MGM — Chemist HAD
7.997 7.992 7.987 7.976 7.971 7.963 7.926 7.905 7.894 7.874 7.653 7.632 7.565 7.561 7.549 7.544 7.539 7.527 7.523 7.518 7.429 7.423 7.416 7.412 7.117 7.098 7.078 6.156 6.136
5c
Cl
O
N
H
O
OH
1H NMR
Zoom
3.33 1.00 0.92 2.26 1.34 1.03 1.00
8.2 8.0 7.8 7.6 7.4 7.2 7.0 6.8 6.6 6.4 6.2 6.0 5.8
f1 (ppm)
26000 22000 18000 14000 10000 6000 2000 -2000

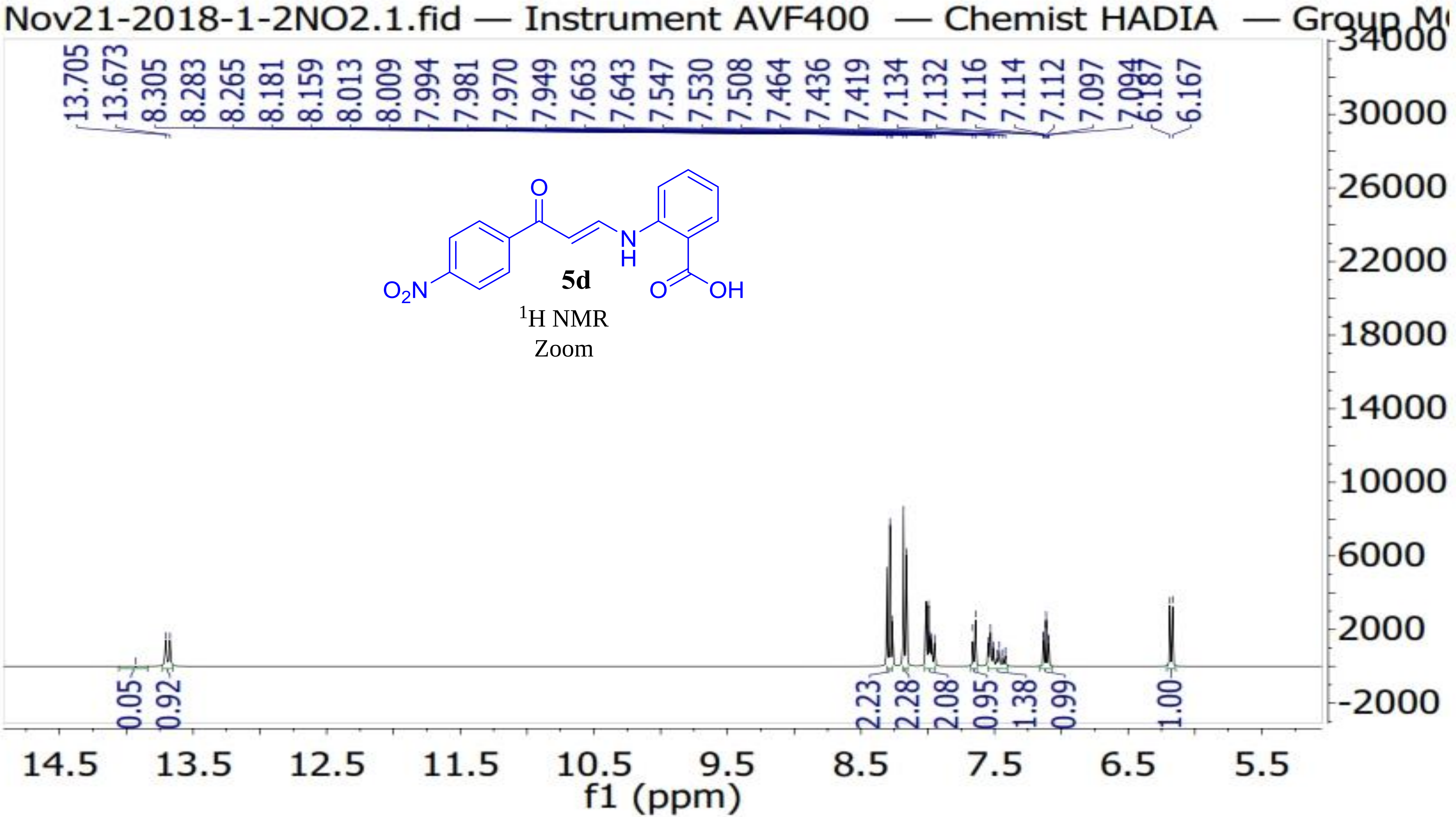
Nov21-2018-1-2NO2.1.fid — Instrument AVF400 — Chemist HADIA — Group
13.705
13.673
8.305
8.283
8.265
8.181
8.159
8.013
8.009
7.994
7.981
7.970
7.949
7.663
7.643
7.547
7.530
7.508
7.464
7.436
7.419
7.134
7.132
7.116
7.114
7.112
7.097
7.094
6.187
6.167
O
N
H
O2N
5d
1H NMR
Zoom
O
OH
34000
30000
26000
22000
18000
14000
10000
6000
2000
-2000
0.05
0.92
2.23
2.28
2.08
0.95
1.38
0.99
1.00
14.5
13.5
12.5
11.5
10.5
9.5
8.5
7.5
6.5
5.5
f1 (ppm)

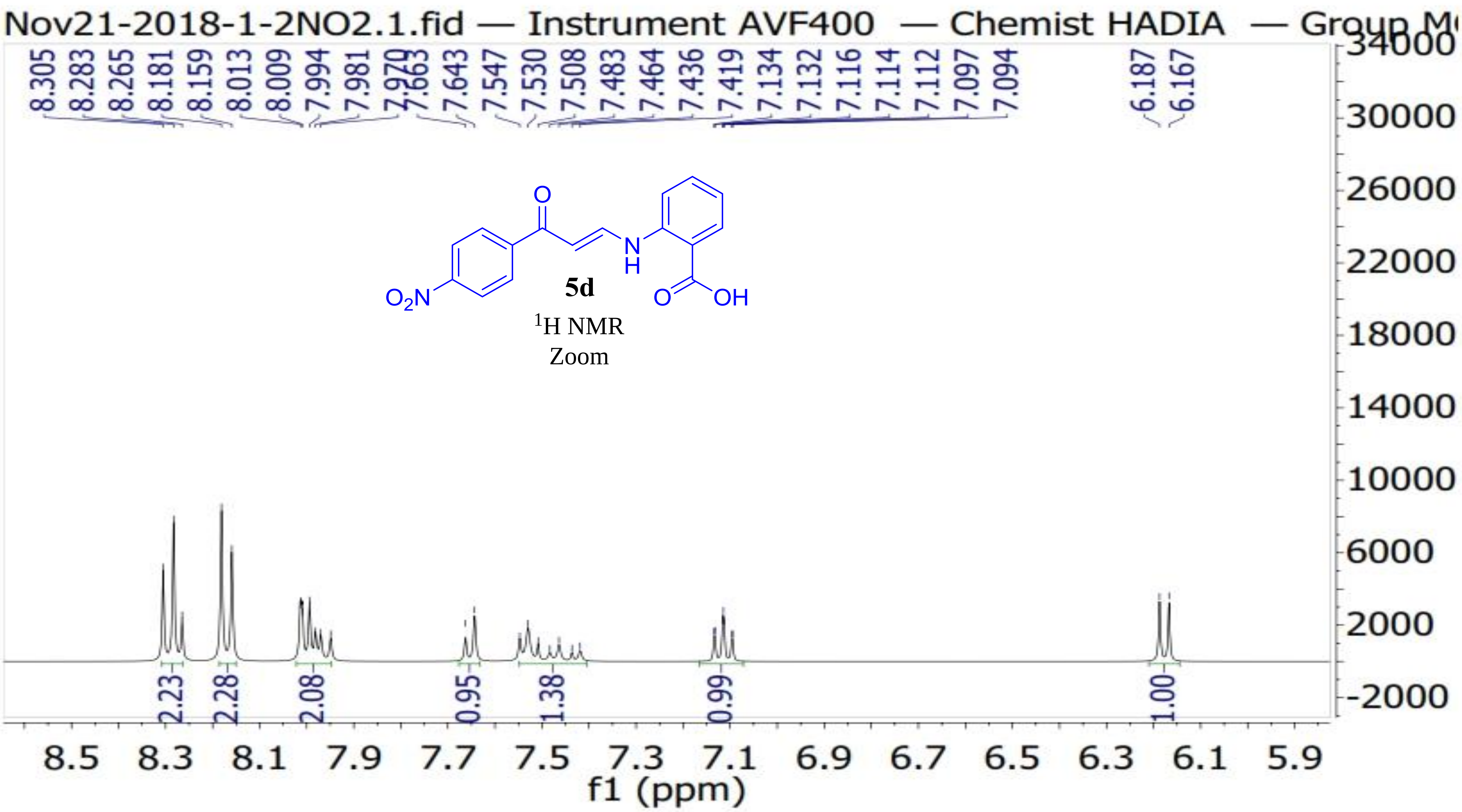
Nov21-2018-1-2NO2.1.fid — Instrument AVF400 — Chemist HADIA — Group M
8.305 8.283 8.265 8.181 8.159 8.013 8.009 7.994 7.981 7.970 7.663 7.643 7.547 7.530 7.508 7.483 7.464 7.436 7.419 7.134 7.132 7.116 7.114 7.112 7.097 7.094 6.187 6.167
O
N
H
O2N
5d
O
OH
1H NMR
Zoom
2.23 2.28 2.08 0.95 1.38 0.99 1.00
f1 (ppm)

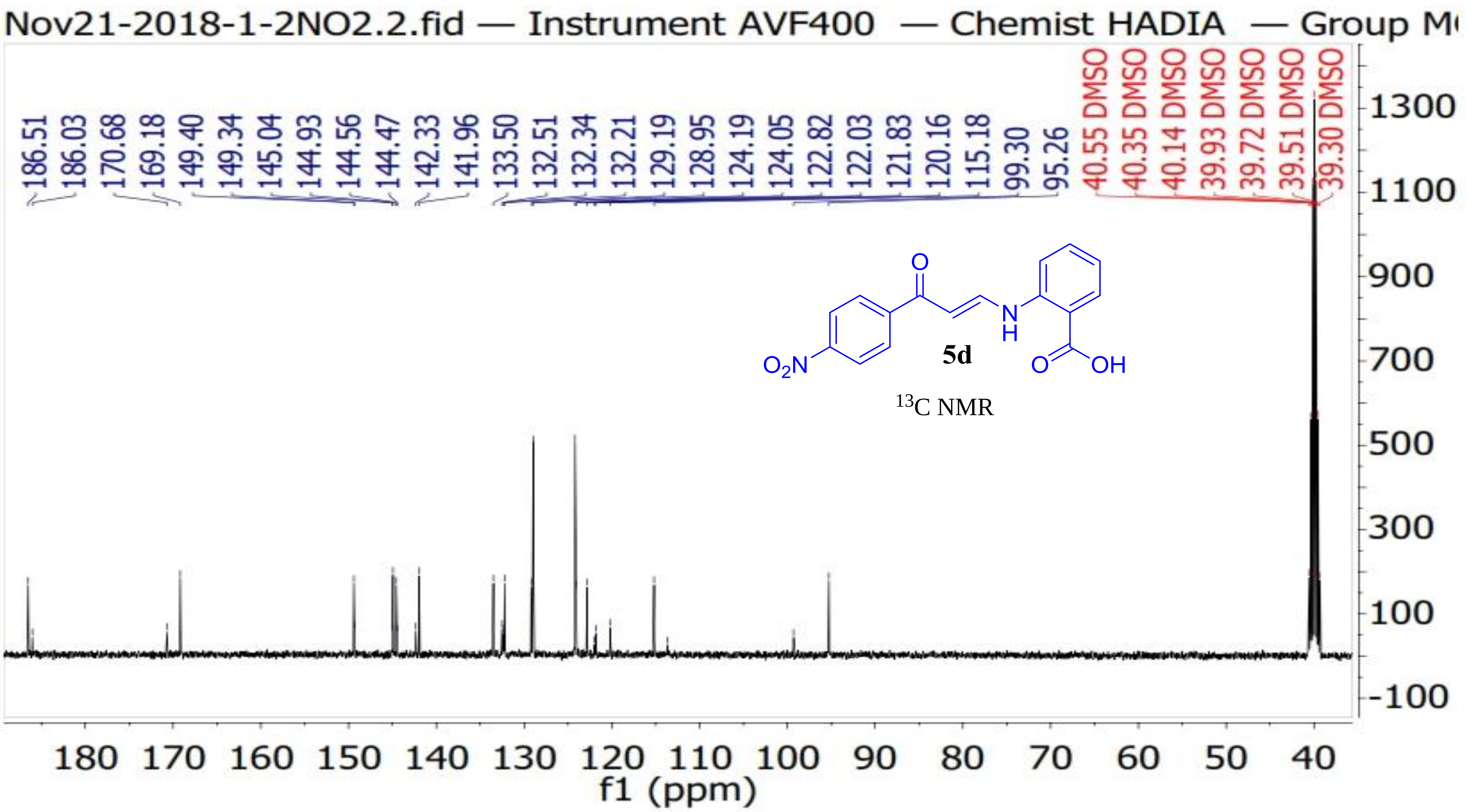
Nov21-2018-1-2NO2.2.fid — Instrument AVF400 — Chemist HADIA — Group M
186.51
186.03
170.68
169.18
149.40
149.34
145.04
144.93
144.56
144.47
142.33
141.96
133.50
132.51
132.34
132.21
129.19
128.95
124.19
124.05
122.82
122.03
121.83
120.16
115.18
99.30
95.26
40.55 DMSO
40.35 DMSO
40.14 DMSO
39.93 DMSO
39.72 DMSO
39.51 DMSO
39.30 DMSO
O
N
H
O_2N
5d
O
OH
^{13}C NMR
1300
1100
900
700
500
300
100
-100
180
170
160
150
140
130
120
110
100
90
80
70
60
50
40
f1 (ppm)

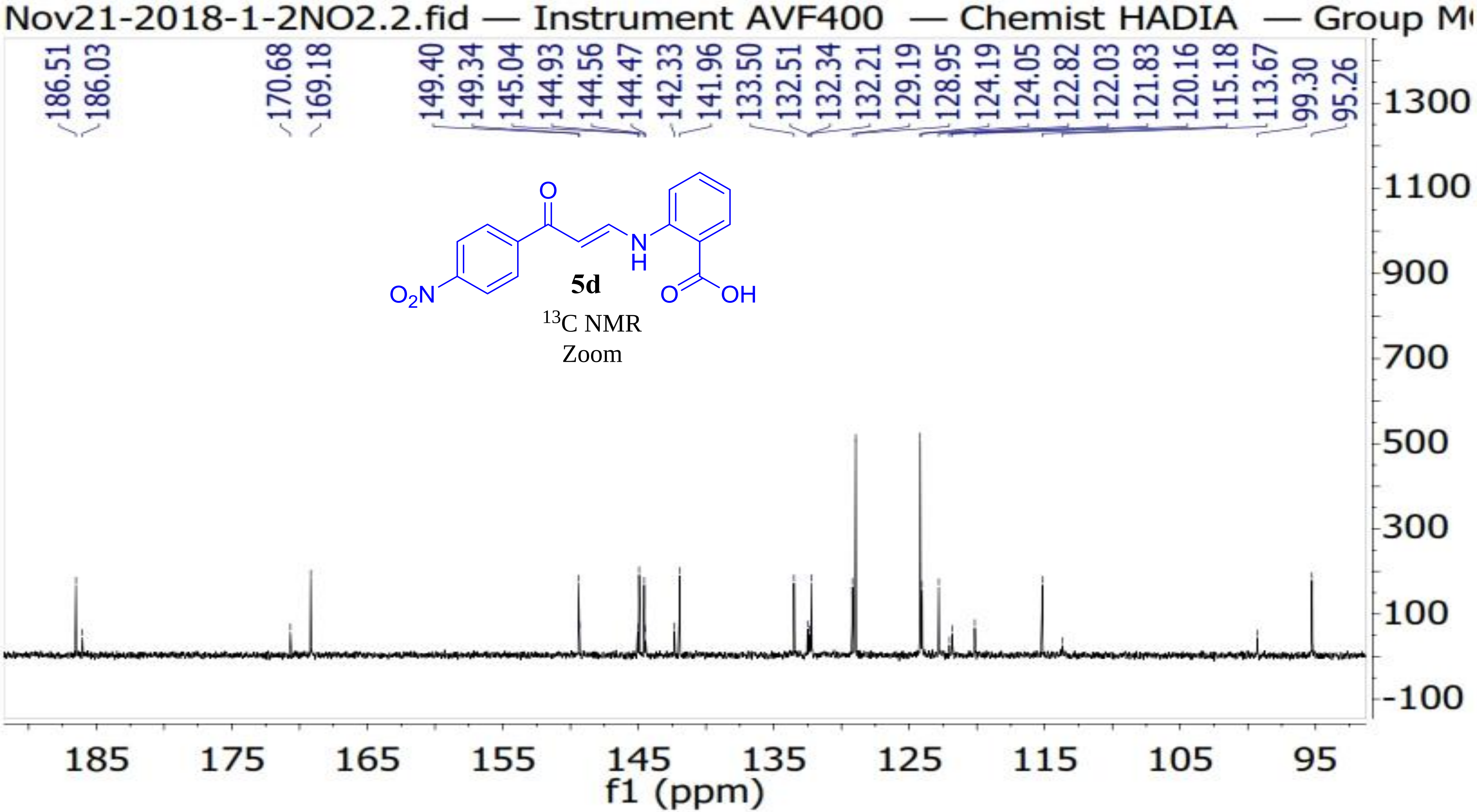
Nov21-2018-1-2NO2.2.fid — Instrument AVF400 — Chemist HADIA — Group M
186.51
186.03
170.68
169.18
149.40
149.34
145.04
144.93
144.56
144.47
142.33
141.96
133.50
132.51
132.34
132.21
129.19
128.95
124.19
124.05
122.82
122.03
121.83
120.16
115.18
113.67
99.30
95.26
O
O_2N
N
H
O
OH
5d
^{13}C NMR
Zoom
1300
1100
900
700
500
300
100
-100
185
175
165
155
145
135
125
115
105
95
f1 (ppm)

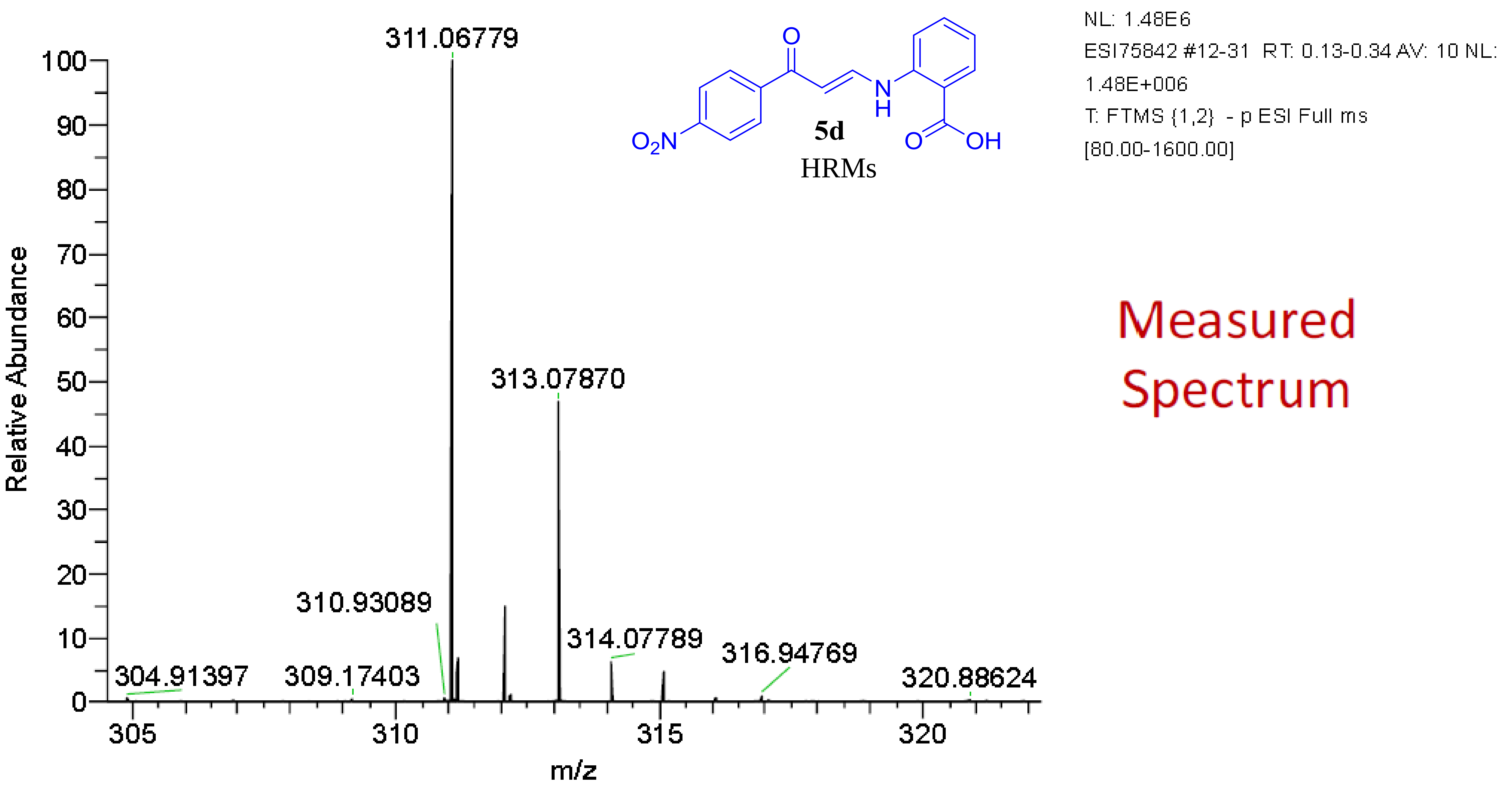

311.06779
313.07870
310.93089
314.07789
316.94769
304.91397
309.17403
320.88624
Relative Abundance
m/z
100
90
80
70
60
50
40
30
20
10
0
305
310
315
320
O
N
H
O₂N
O
OH
5d
HRMs
NL: 1.48E6
ESI75842 #12-31 RT: 0.13-0.34 AV: 10 NL: 1.48E+006
T: FTMS {1,2} - p ESI Full ms [80.00-1600.00]
Measured Spectrum

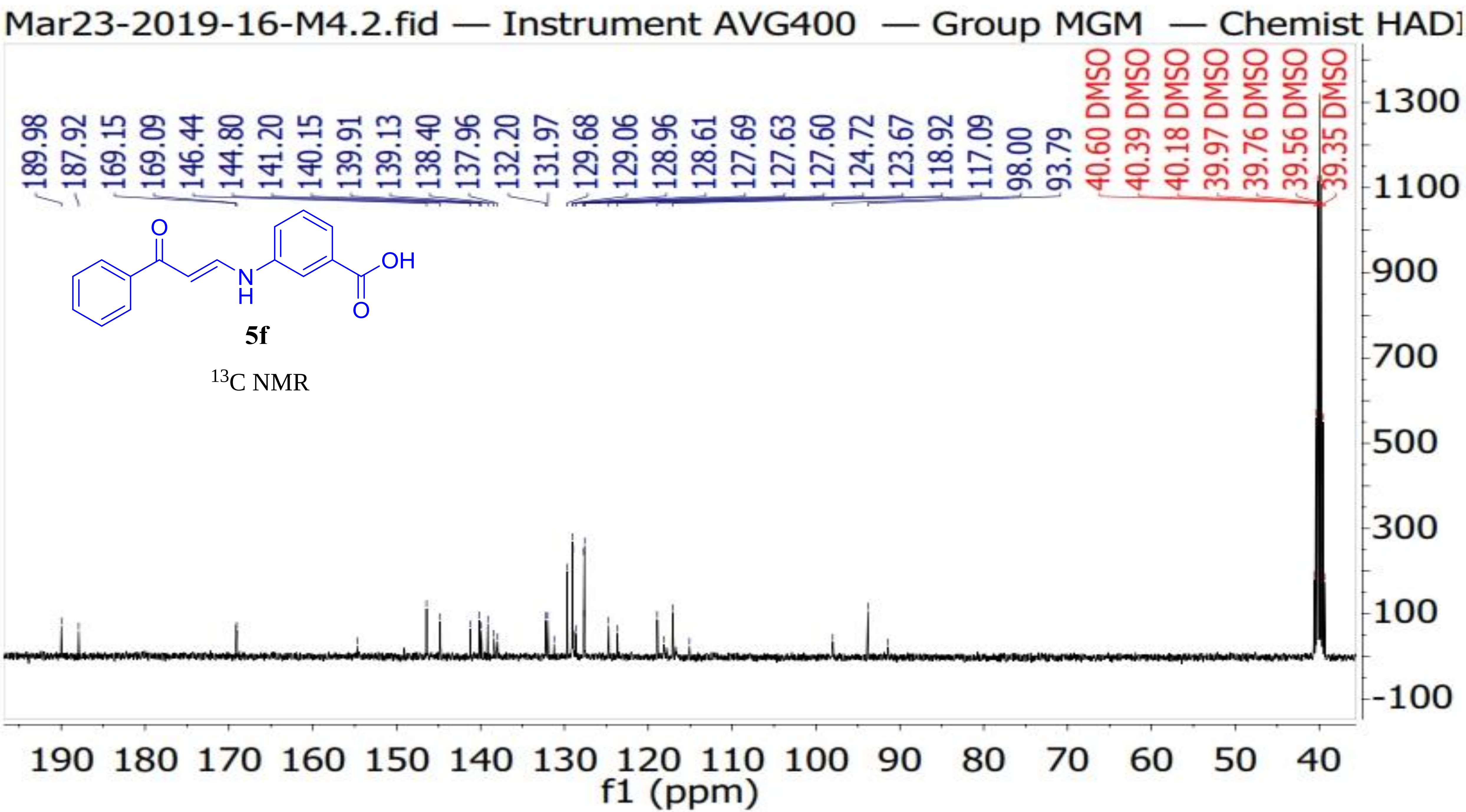
Mar23-2019-16-M4.2.fid — Instrument AVG400 — Group MGM — Chemist HADI
189.98
187.92
169.15
169.09
146.44
144.80
141.20
140.15
139.91
139.13
138.40
137.96
132.20
131.97
129.68
129.06
128.96
128.61
127.69
127.63
127.60
124.72
123.67
118.92
117.09
98.00
93.79
40.60 DMSO
40.39 DMSO
40.18 DMSO
39.97 DMSO
39.76 DMSO
39.56 DMSO
39.35 DMSO
O
OH
N
H
O
5f
^{13}C NMR
f1 (ppm)

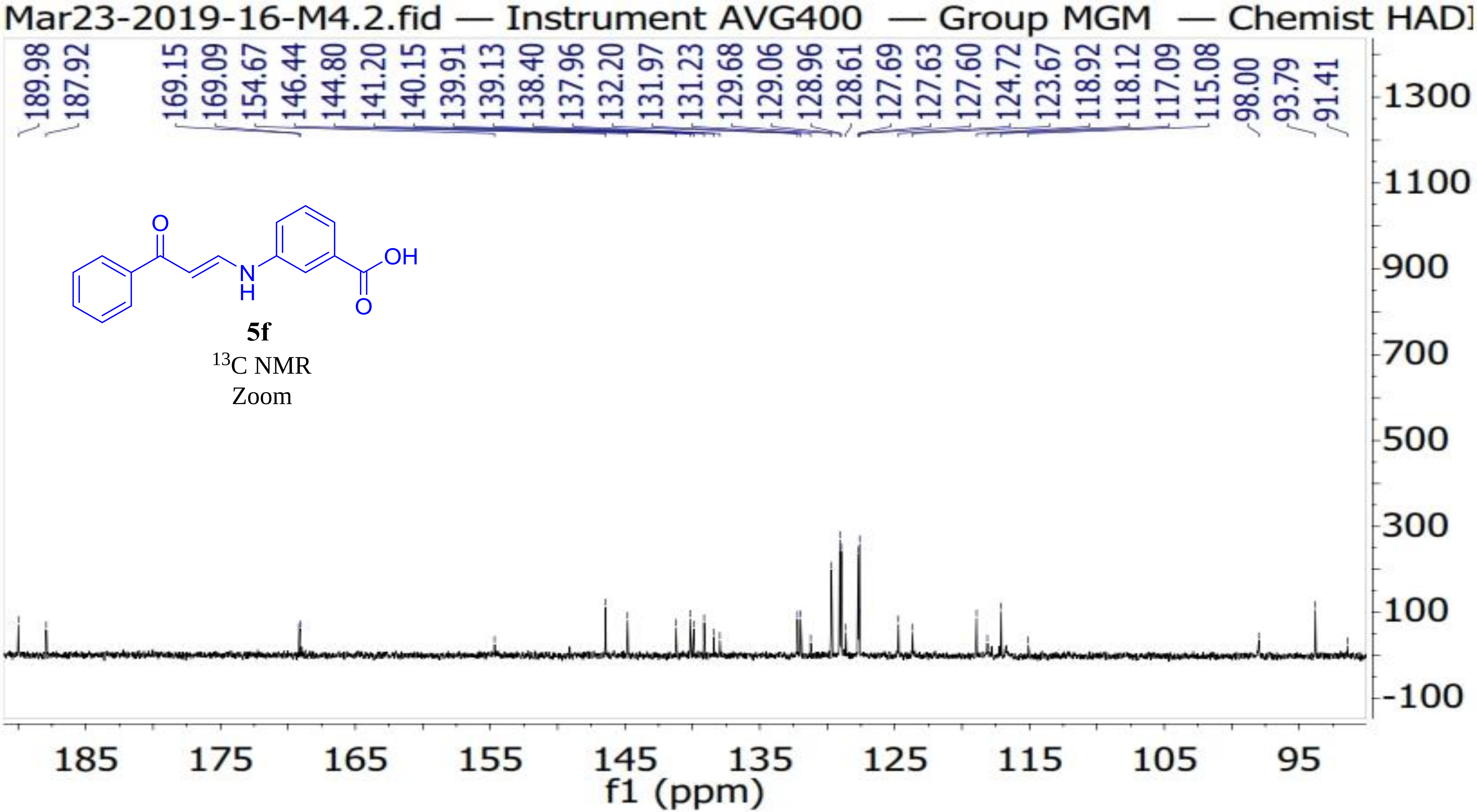
Mar23-2019-16-M4.2.fid — Instrument AVG400 — Group MGM — Chemist HADI
189.98
187.92
169.15
169.09
154.67
146.44
144.80
141.20
140.15
139.91
139.13
138.40
137.96
132.20
131.97
131.23
129.68
129.06
128.96
128.61
127.69
127.63
127.60
124.72
123.67
118.92
118.12
117.09
115.08
98.00
93.79
91.41
O
OH
N
H
O
5f
13C NMR
Zoom
1300
1100
900
700
500
300
100
-100
185
175
165
155
145
135
125
115
105
95
f1 (ppm)

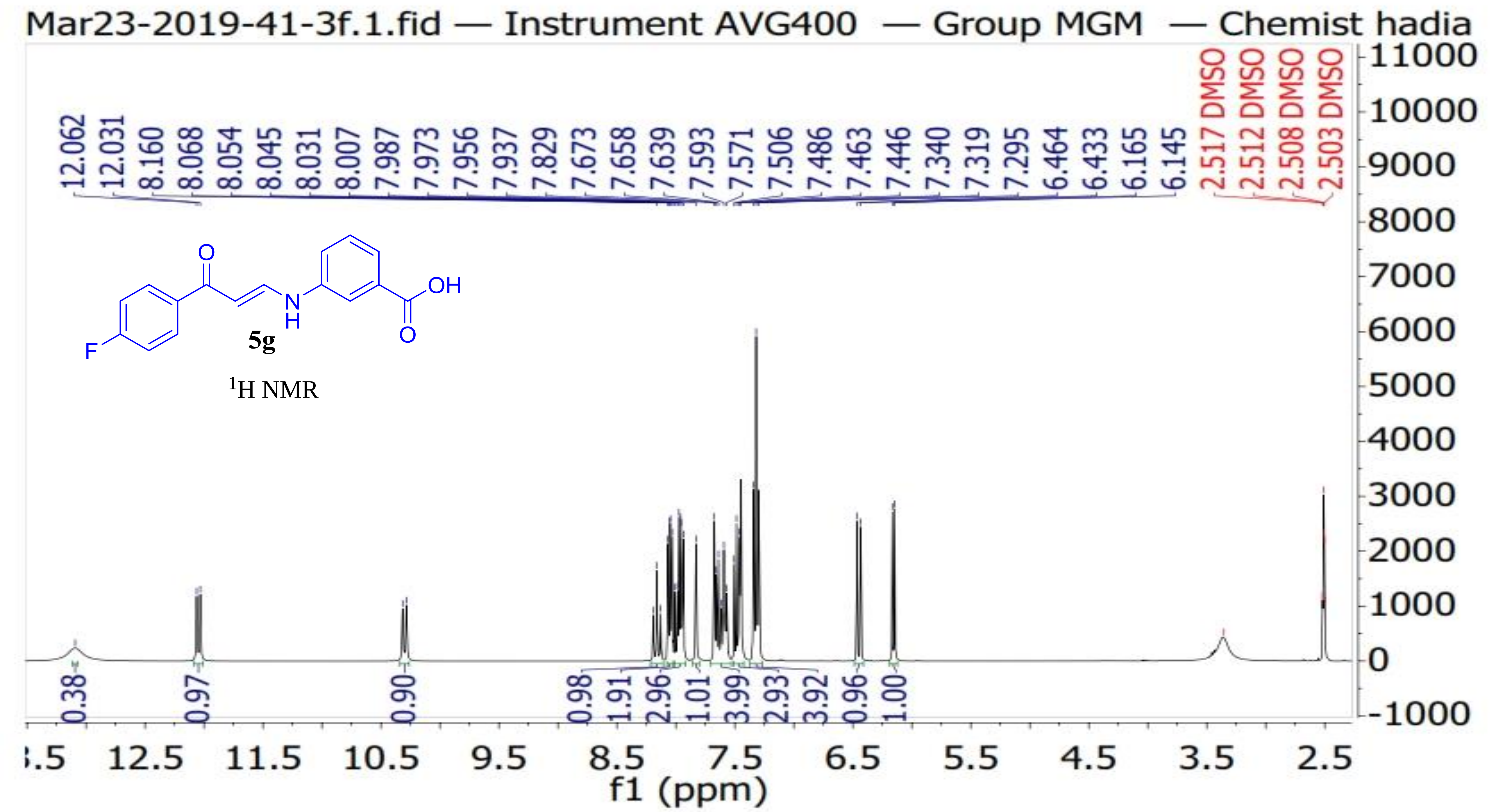
Mar23-2019-41-3f.1.fid — Instrument AVG400 — Group MGM — Chemist hadia
12.062 12.031 8.160 8.068 8.054 8.045 8.031 8.007 7.987 7.973 7.956 7.937 7.829 7.673 7.658 7.639 7.593 7.571 7.506 7.486 7.463 7.446 7.340 7.319 7.295 6.464 6.433 6.165 6.145
2.517 DMSO 2.512 DMSO 2.508 DMSO 2.503 DMSO
O
OH
N
H
O
F
5g
^{1}H NMR
0.38 0.97 0.90 0.98 1.91 2.96 1.01 3.99 2.93 3.92 0.96 1.00
f1 (ppm)

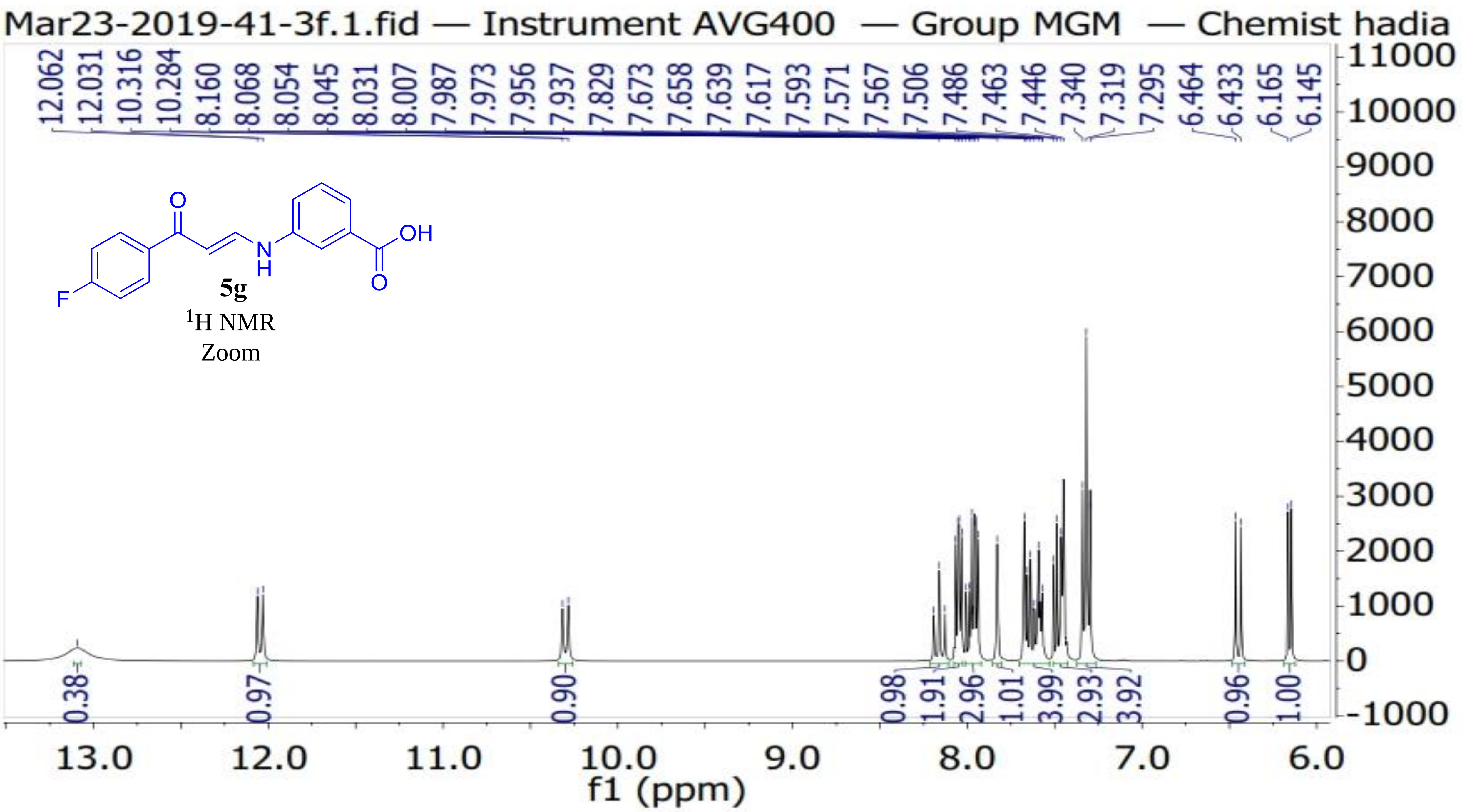

Mar23-2019-41-3f.1.fid — Instrument AVG400 — Group MGM — Chemist hadia
12.062
12.031
10.316
10.284
8.160
8.068
8.054
8.045
8.031
8.007
7.987
7.973
7.956
7.937
7.829
7.673
7.658
7.639
7.617
7.593
7.571
7.567
7.506
7.486
7.463
7.446
7.340
7.319
7.295
6.464
6.433
6.165
6.145
O
OH
N
H
O
F
5g
1H NMR
Zoom
0.38
0.97
0.90
0.98
1.91
2.96
1.01
3.99
2.93
3.92
0.96
1.00
11000
10000
9000
8000
7000
6000
5000
4000
3000
2000
1000
0
-1000
13.0
12.0
11.0
10.0
9.0
8.0
7.0
6.0
f1 (ppm)

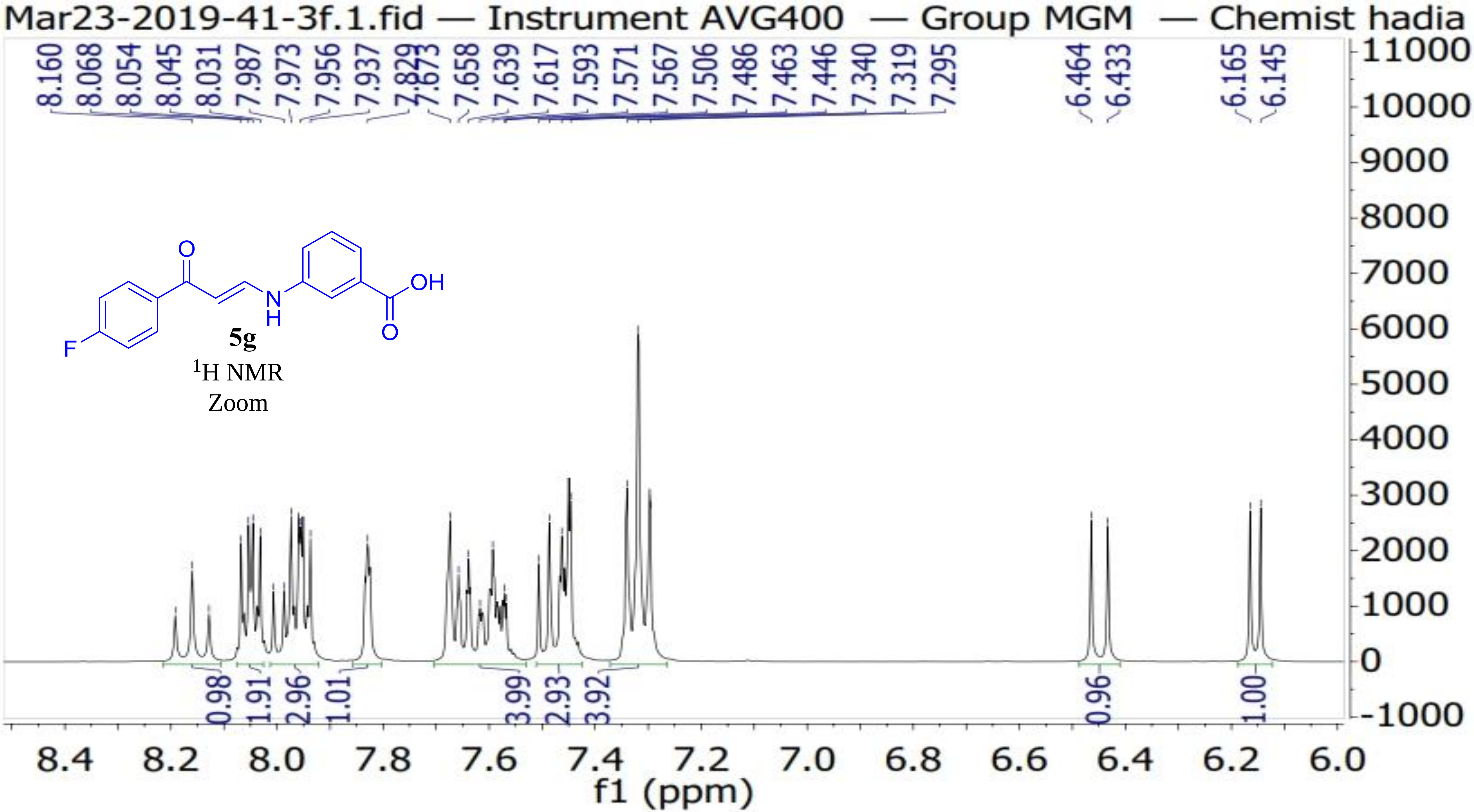

Mar23-2019-41-3f.1.fid — Instrument AVG400 — Group MGM — Chemist hadia
8.160 8.068 8.054 8.045 8.031 7.987 7.973 7.956 7.937 7.829 7.673 7.658 7.639 7.617 7.593 7.571 7.567 7.506 7.486 7.463 7.446 7.340 7.319 7.295 6.464 6.433 6.165 6.145
O
OH
N
H
O
F
5g
1H NMR
Zoom
0.98 1.91 2.96 1.01 3.99 2.93 3.92 0.96 1.00
11000 10000 9000 8000 7000 6000 5000 4000 3000 2000 1000 0 -1000
8.4 8.2 8.0 7.8 7.6 7.4 7.2 7.0 6.8 6.6 6.4 6.2 6.0
f1 (ppm)

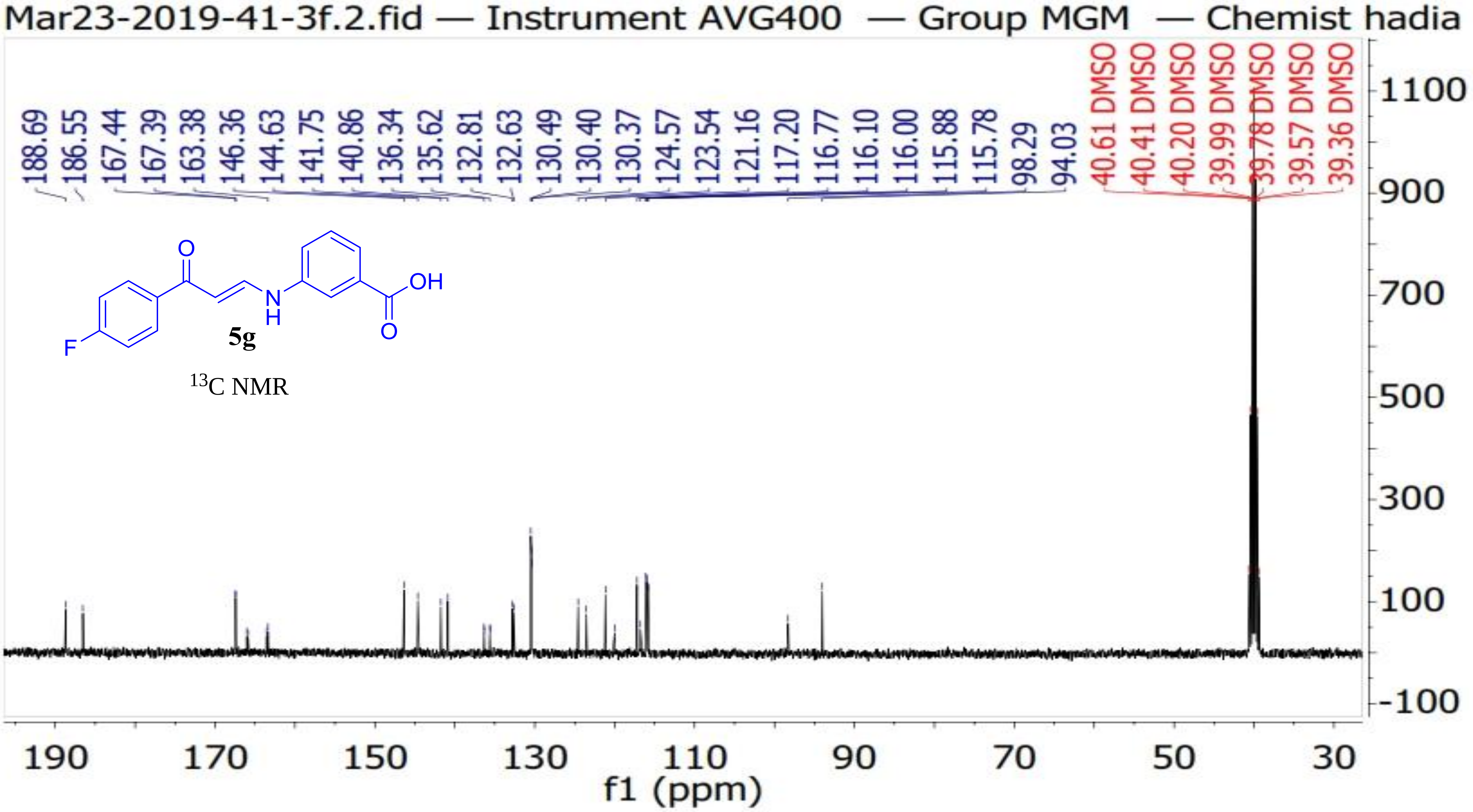
Mar23-2019-41-3f.2.fid — Instrument AVG400 — Group MGM — Chemist hadia
188.69
186.55
167.44
167.39
163.38
146.36
144.63
141.75
140.86
136.34
135.62
132.81
132.63
130.49
130.40
130.37
124.57
123.54
121.16
117.20
116.77
116.10
116.00
115.88
115.78
98.29
94.03
40.61 DMSO
40.41 DMSO
40.20 DMSO
39.99 DMSO
39.78 DMSO
39.57 DMSO
39.36 DMSO
O
OH
N
H
O
F
5g
^{13}C NMR
f1 (ppm)

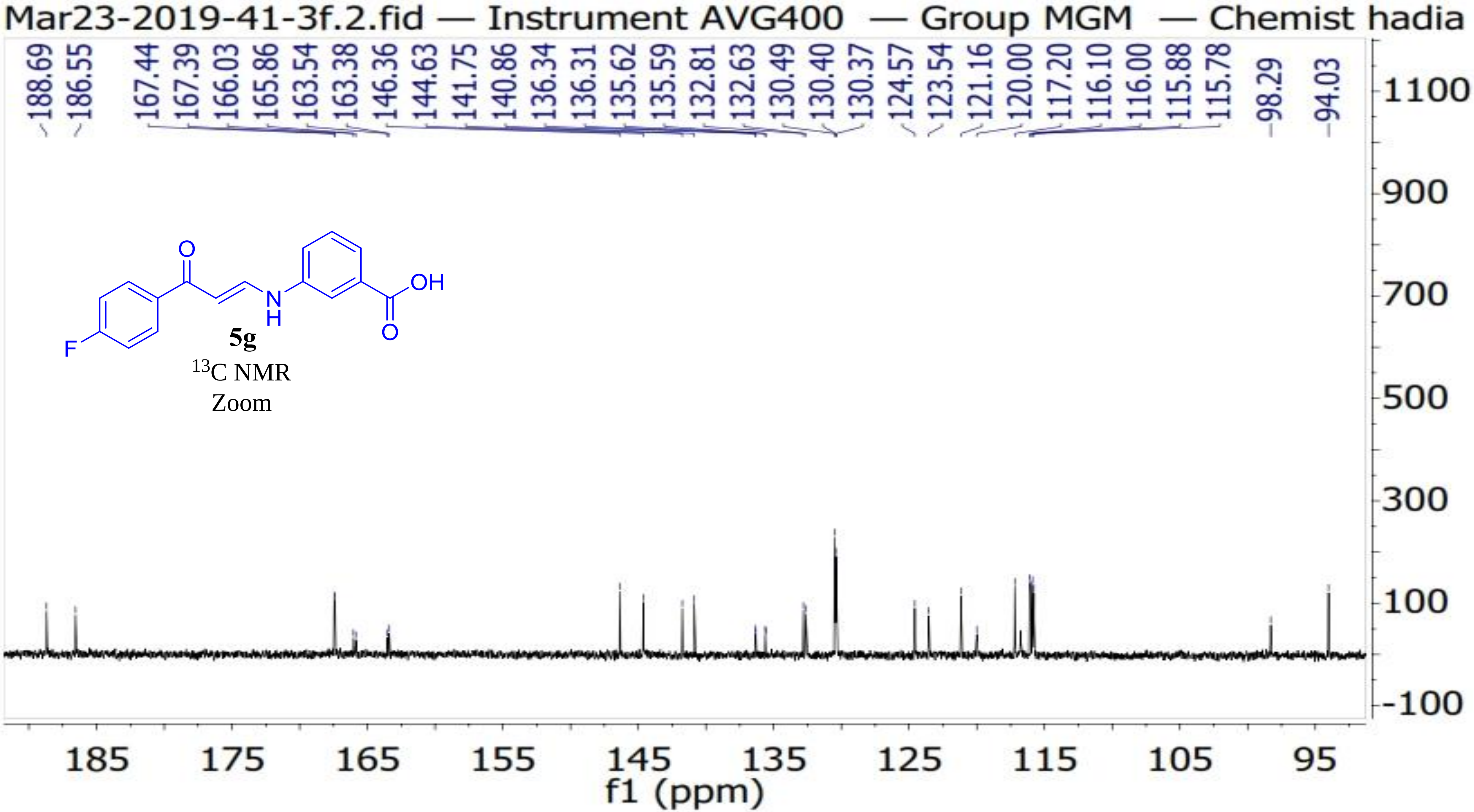
Mar23-2019-41-3f.2.fid — Instrument AVG400 — Group MGM — Chemist hadia
188.69
186.55
167.44
167.39
166.03
165.86
163.54
163.38
146.36
144.63
141.75
140.86
136.34
136.31
135.62
135.59
132.81
132.63
130.49
130.40
130.37
124.57
123.54
121.16
120.00
117.20
116.10
116.00
115.88
115.78
98.29
94.03
O
OH
N
H
O
F
5g
13C NMR
Zoom
1100
900
700
500
300
100
-100
185
175
165
155
145
135
125
115
105
95
f1 (ppm)

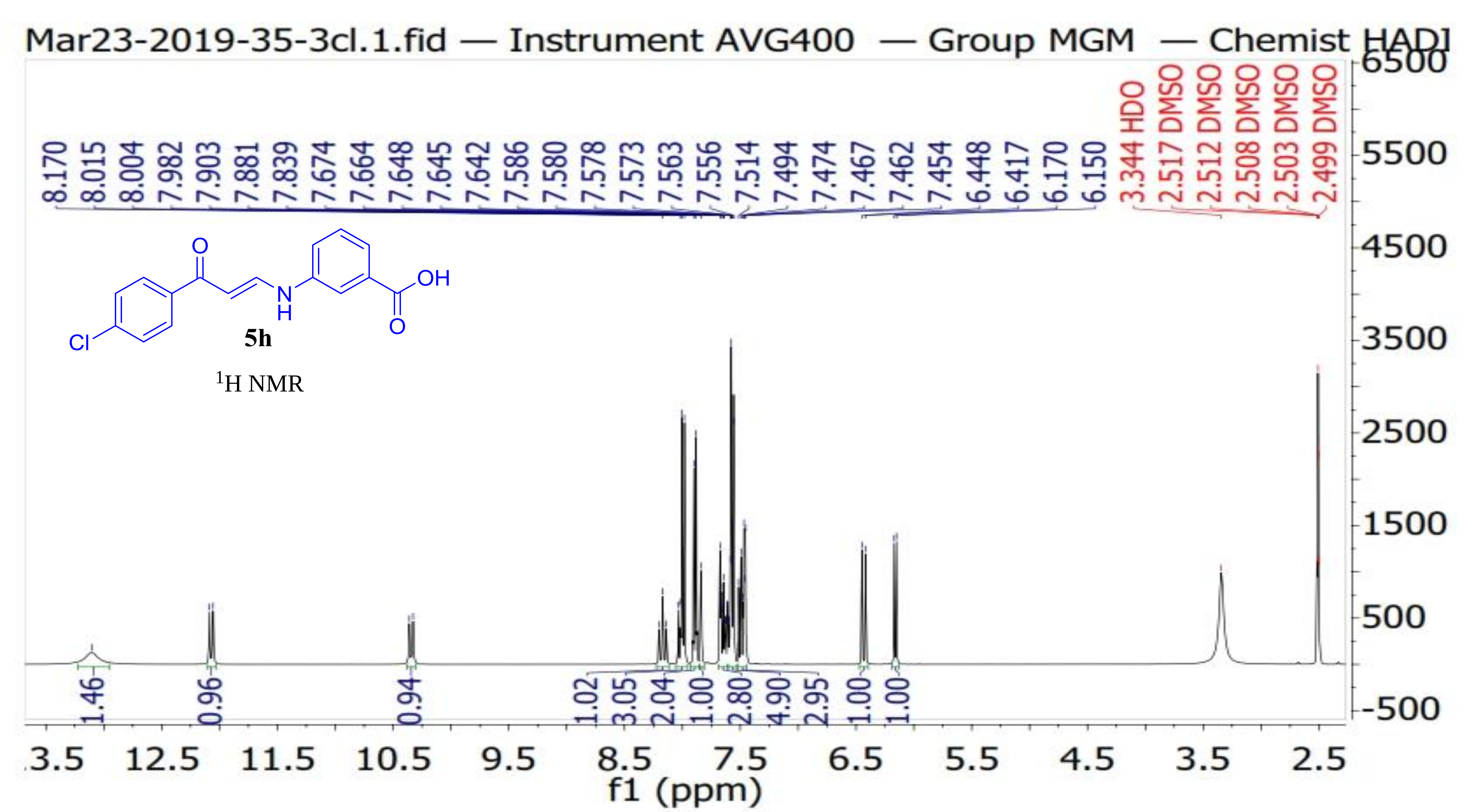
Mar23-2019-35-3cl.1.fid — Instrument AVG400 — Group MGM — Chemist HADI
8.170 8.015 8.004 7.982 7.903 7.881 7.839 7.674 7.664 7.648 7.645 7.642 7.586 7.580 7.578 7.573 7.563 7.556 7.514 7.494 7.474 7.467 7.462 7.454 6.448 6.417 6.170 6.150
3.344 HDO
2.517 DMSO
2.512 DMSO
2.508 DMSO
2.503 DMSO
2.499 DMSO
O
OH
N
H
O
Cl
5h
^{1}H NMR
1.46 0.96 0.94 1.02 3.05 2.04 1.00 2.80 4.90 2.95 1.00 1.00
f1 (ppm)

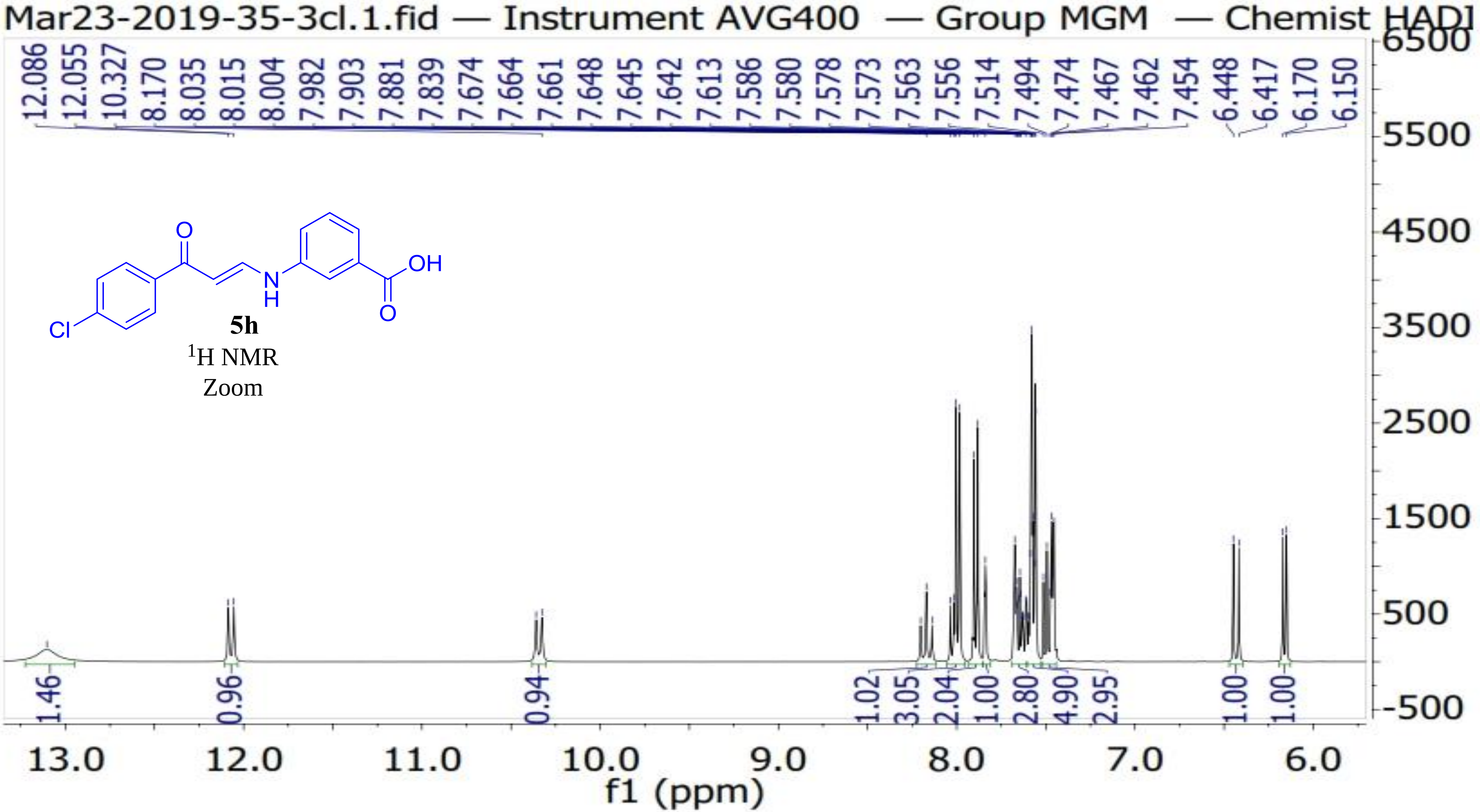
Mar23-2019-35-3cl.1.fid — Instrument AVG400 — Group MGM — Chemist HADI
12.086 12.055 10.327 8.170 8.035 8.015 8.004 7.982 7.903 7.881 7.839 7.674 7.664 7.661 7.648 7.645 7.642 7.613 7.586 7.580 7.578 7.573 7.563 7.556 7.514 7.494 7.474 7.467 7.462 7.454 6.448 6.417 6.170 6.150
O
OH
N
H
O
Cl
5h
1H NMR
Zoom
1.46 0.96 0.94 1.02 3.05 2.04 1.00 2.80 4.90 2.95 1.00 1.00
6500 5500 4500 3500 2500 1500 500 -500
13.0 12.0 11.0 10.0 9.0 8.0 7.0 6.0
f1 (ppm)

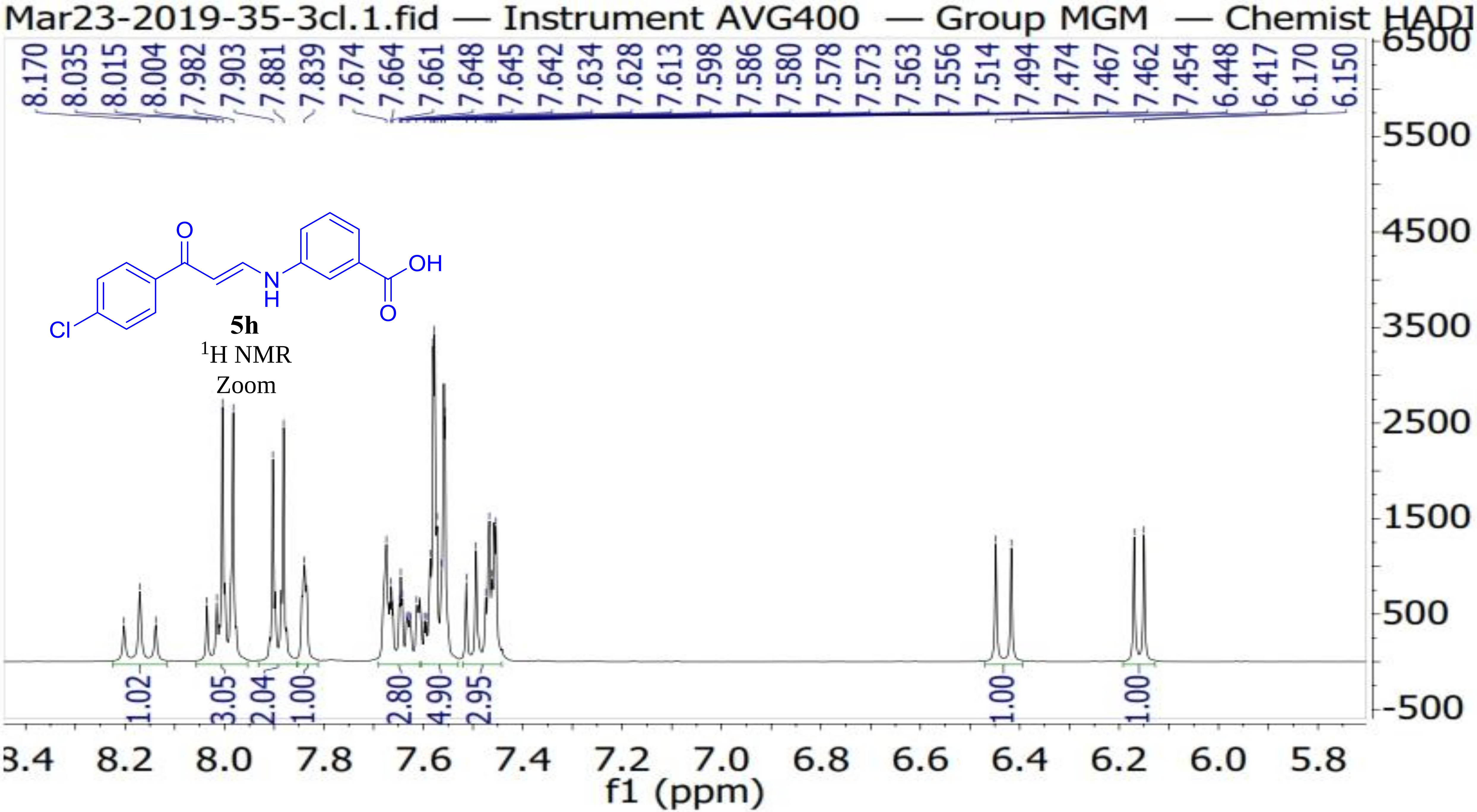

Mar23-2019-35-3cl.1.fid — Instrument AVG400 — Group MGM — Chemist HADI
8.170 8.035 8.015 8.004 7.982 7.903 7.881 7.839 7.674 7.664 7.661 7.648 7.645 7.642 7.634 7.628 7.613 7.598 7.586 7.580 7.578 7.573 7.563 7.556 7.514 7.494 7.474 7.467 7.462 7.454 6.448 6.417 6.170 6.150
O
OH
N
H
O
Cl
5h
1H NMR
Zoom
1.02 3.05 2.04 1.00 2.80 4.90 2.95 1.00 1.00
8.4 8.2 8.0 7.8 7.6 7.4 7.2 7.0 6.8 6.6 6.4 6.2 6.0 5.8
f1 (ppm)
6500 5500 4500 3500 2500 1500 500 -500

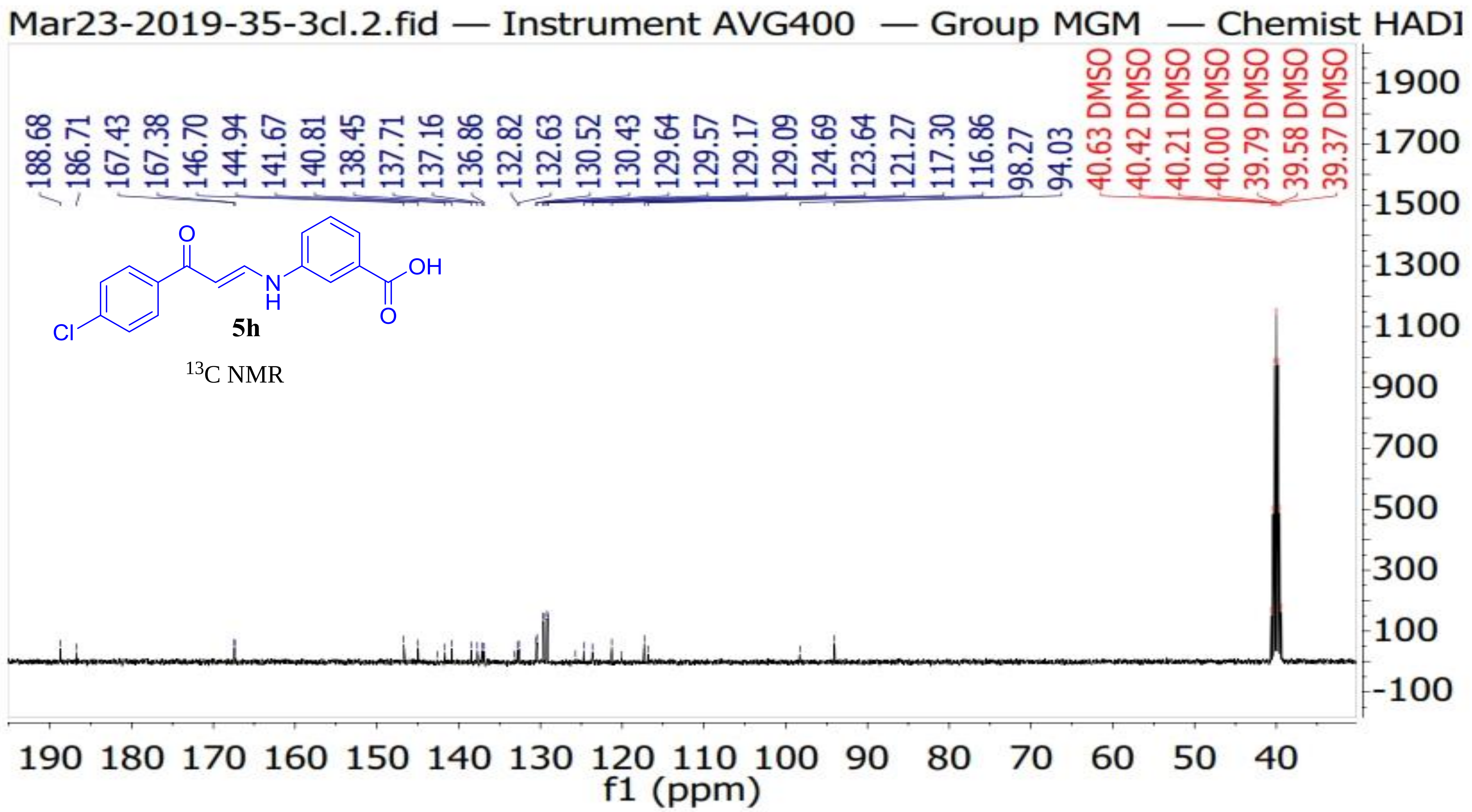

Mar23-2019-35-3cl.2.fid — Instrument AVG400 — Group MGM — Chemist HADI
188.68
186.71
167.43
167.38
146.70
144.94
141.67
140.81
138.45
137.71
137.16
136.86
132.82
132.63
130.52
130.43
129.64
129.57
129.17
129.09
124.69
123.64
121.27
117.30
116.86
98.27
94.03
40.63 DMSO
40.42 DMSO
40.21 DMSO
40.00 DMSO
39.79 DMSO
39.58 DMSO
39.37 DMSO
5h
^{13}C NMR
f1 (ppm)

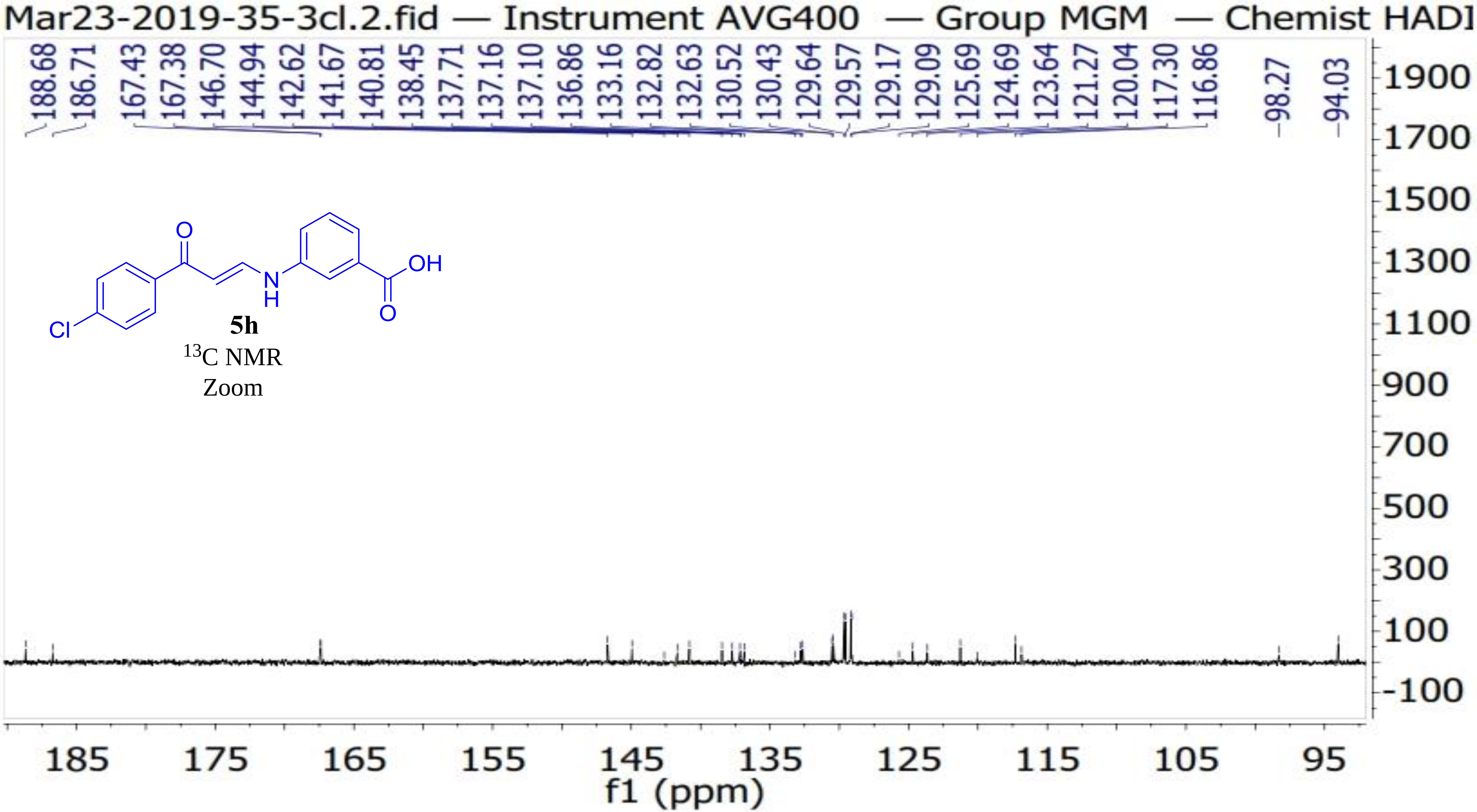
Mar23-2019-35-3cl.2.fid — Instrument AVG400 — Group MGM — Chemist HADI
188.68
186.71
167.43
167.38
146.70
144.94
142.62
141.67
140.81
138.45
137.71
137.16
137.10
136.86
133.16
132.82
132.63
130.52
130.43
129.64
129.57
129.17
129.09
125.69
124.69
123.64
121.27
120.04
117.30
116.86
98.27
94.03
O
OH
N
H
O
Cl
5h
13C NMR
Zoom
1900
1700
1500
1300
1100
900
700
500
300
100
-100
185
175
165
155
145
135
125
115
105
95
f1 (ppm)

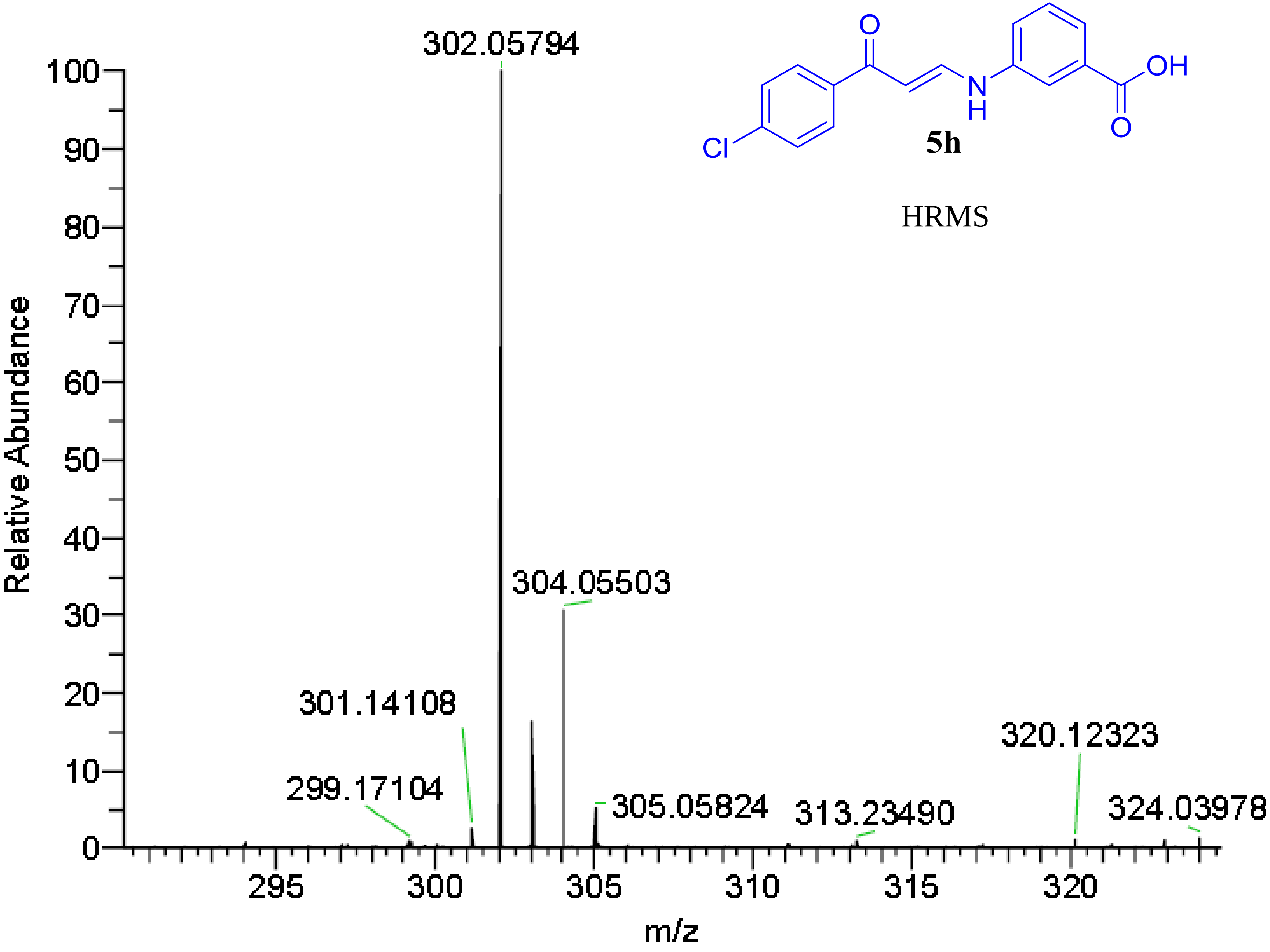

5h

HRMS

Measured Spectrum

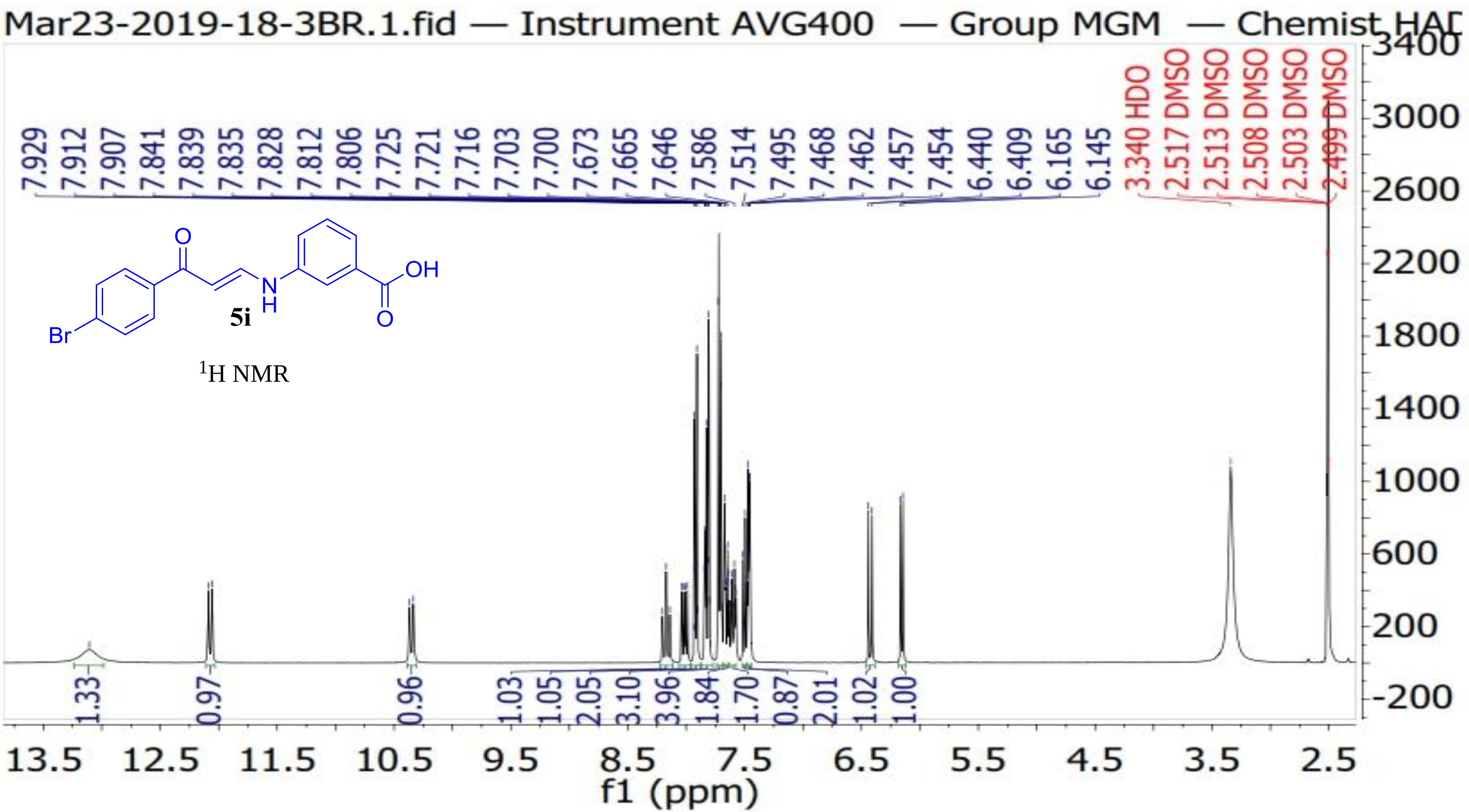

Mar23-2019-18-3BR.1.fid — Instrument AVG400 — Group MGM — Chemist HAC
7.929
7.912
7.907
7.841
7.839
7.835
7.828
7.812
7.806
7.725
7.721
7.716
7.703
7.700
7.673
7.665
7.646
7.586
7.514
7.495
7.468
7.462
7.457
7.454
6.440
6.409
6.165
6.145
3.340 HDO
2.517 DMSO
2.513 DMSO
2.508 DMSO
2.503 DMSO
2.499 DMSO
O
OH
N
H
O
Br
5i
1H NMR
1.33
0.97
0.96
1.03
1.05
2.05
3.10
3.96
1.84
1.70
0.87
2.01
1.02
1.00
13.5
12.5
11.5
10.5
9.5
8.5
7.5
6.5
5.5
4.5
3.5
2.5
f1 (ppm)
3400
3000
2600
2200
1800
1400
1000
600
200
-200

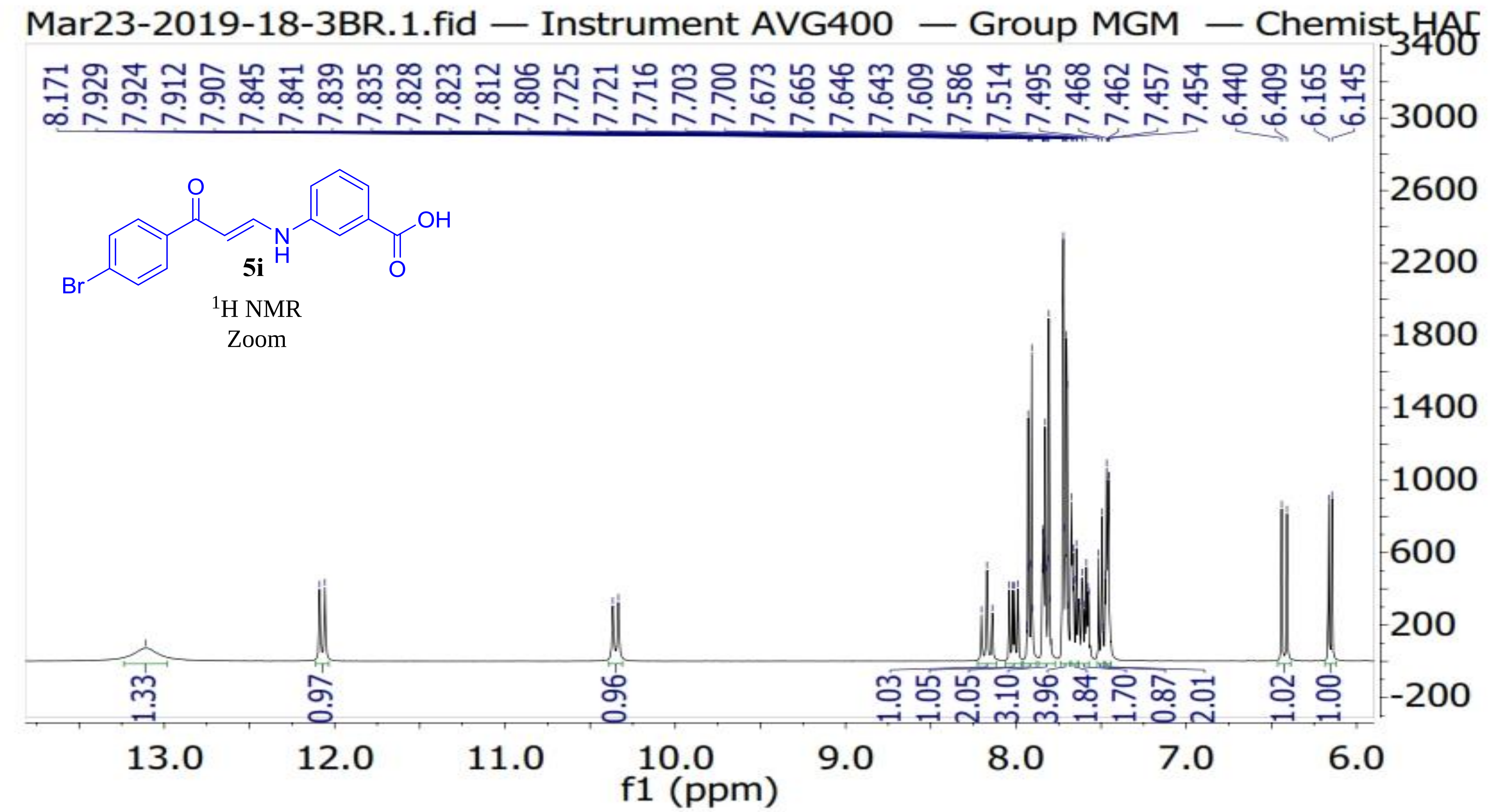

Mar23-2019-18-3BR.1.fid — Instrument AVG400 — Group MGM — Chemist HAD
8.171 7.929 7.924 7.912 7.907 7.845 7.841 7.839 7.835 7.828 7.823 7.812 7.806 7.725 7.721 7.716 7.703 7.700 7.673 7.665 7.646 7.643 7.609 7.586 7.514 7.495 7.468 7.462 7.457 7.454 6.440 6.409 6.165 6.145
O
OH
N
H
Br
O
5i
1H NMR
Zoom
1.33 0.97 0.96 1.03 1.05 2.05 3.10 3.96 1.84 1.70 0.87 2.01 1.02 1.00
13.0 12.0 11.0 10.0 9.0 8.0 7.0 6.0
f1 (ppm)
3400 3000 2600 2200 1800 1400 1000 600 200 -200

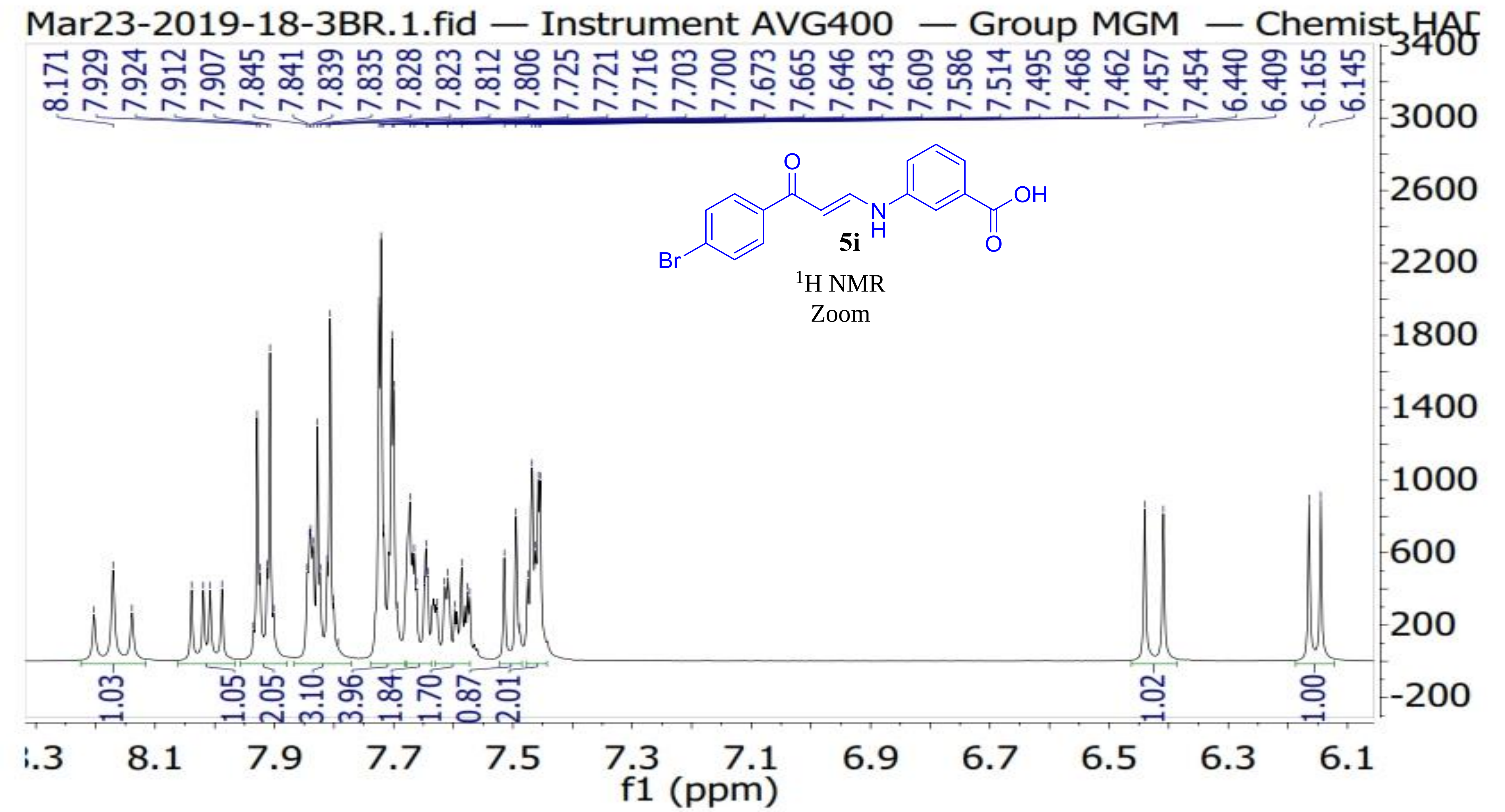
Mar23-2019-18-3BR.1.fid — Instrument AVG400 — Group MGM — Chemist HAD
8.171 7.929 7.924 7.912 7.907 7.845 7.841 7.839 7.835 7.828 7.823 7.812 7.806 7.725 7.721 7.716 7.703 7.700 7.673 7.665 7.646 7.643 7.609 7.586 7.514 7.495 7.468 7.462 7.457 7.454 6.440 6.409 6.165 6.145
Br
O
N
H
OH
O
5i
1H NMR
Zoom
1.03 1.05 2.05 3.10 3.96 1.84 1.70 0.87 2.01 1.02 1.00
3400 3000 2600 2200 1800 1400 1000 600 200 -200
8.3 8.1 7.9 7.7 7.5 7.3 7.1 6.9 6.7 6.5 6.3 6.1
f1 (ppm)

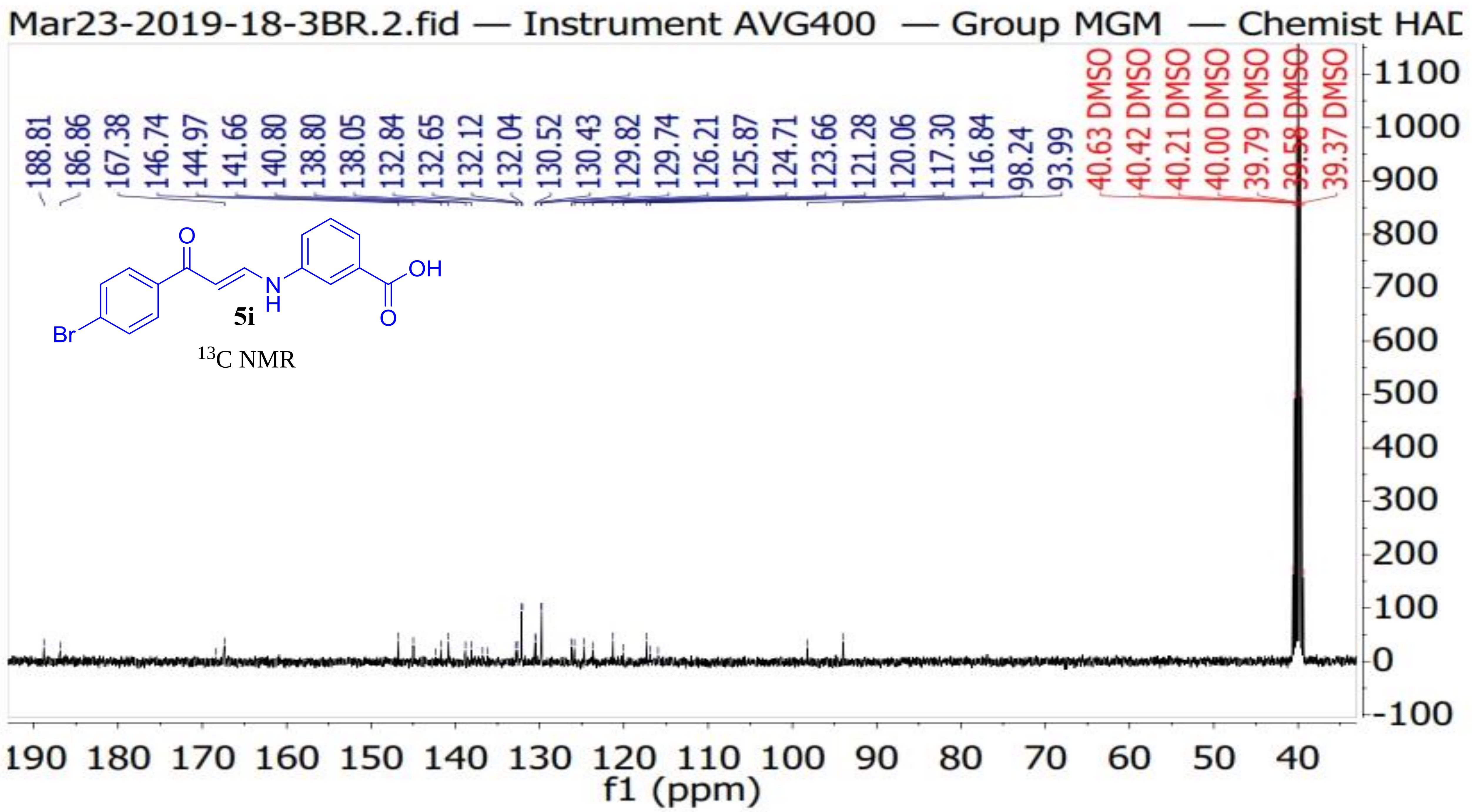

Mar23-2019-18-3BR.2.fid — Instrument AVG400 — Group MGM — Chemist HAD
188.81
186.86
167.38
146.74
144.97
141.66
140.80
138.80
138.05
132.84
132.65
132.12
132.04
130.52
130.43
129.82
129.74
126.21
125.87
124.71
123.66
121.28
120.06
117.30
116.84
98.24
93.99
40.63 DMSO
40.42 DMSO
40.21 DMSO
40.00 DMSO
39.79 DMSO
39.58 DMSO
39.37 DMSO
O
OH
N
H
Br
O
5i
^{13}C NMR
f1 (ppm)

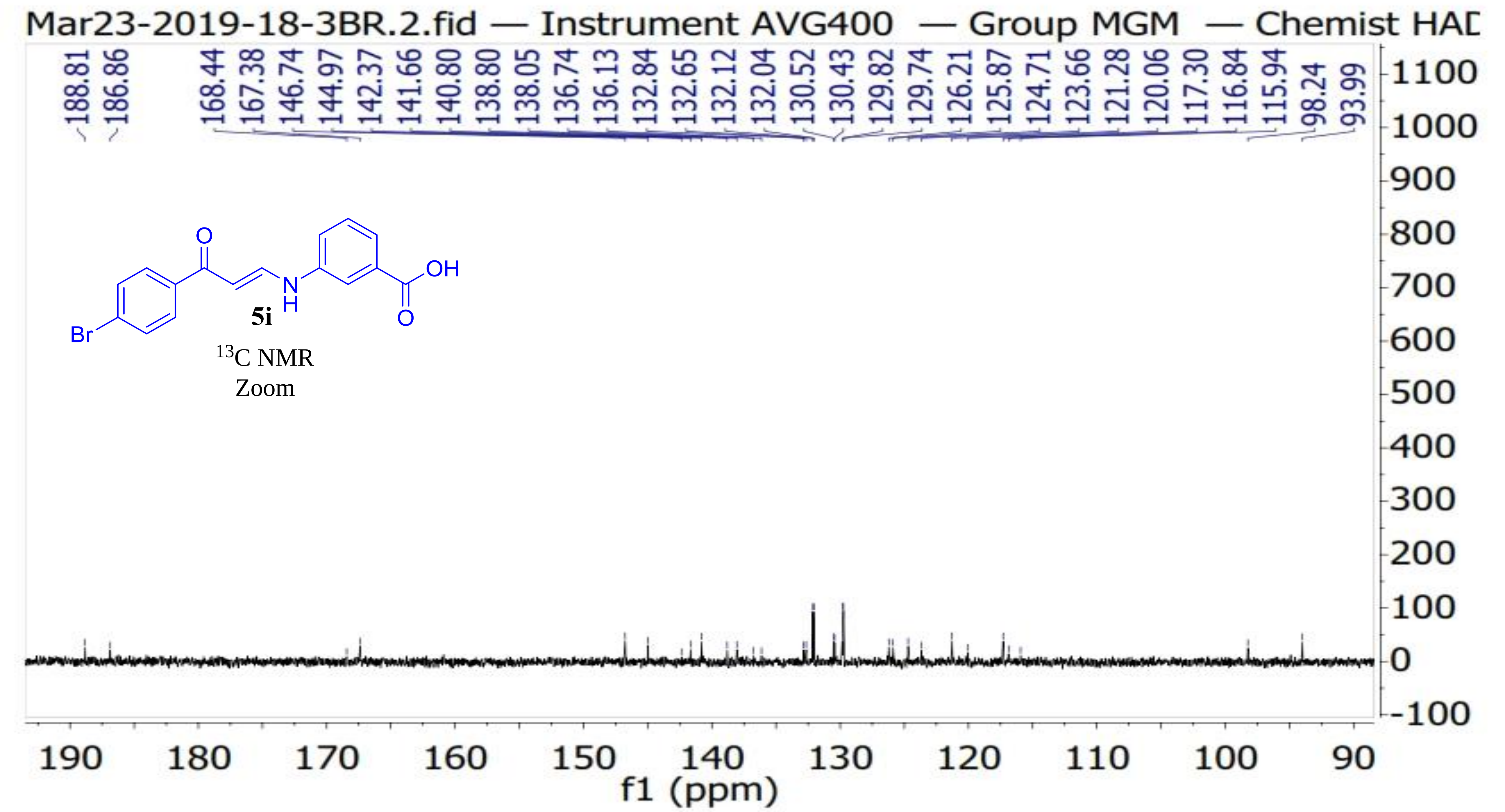

Mar23-2019-18-3BR.2.fid — Instrument AVG400 — Group MGM — Chemist HAD
188.81
186.86
168.44
167.38
146.74
144.97
142.37
141.66
140.80
138.80
138.05
136.74
136.13
132.84
132.65
132.12
132.04
130.52
130.43
129.82
129.74
126.21
125.87
124.71
123.66
121.28
120.06
117.30
116.84
115.94
98.24
93.99
O
OH
N
H
Br
O
5i
^{13}C NMR
Zoom
f1 (ppm)

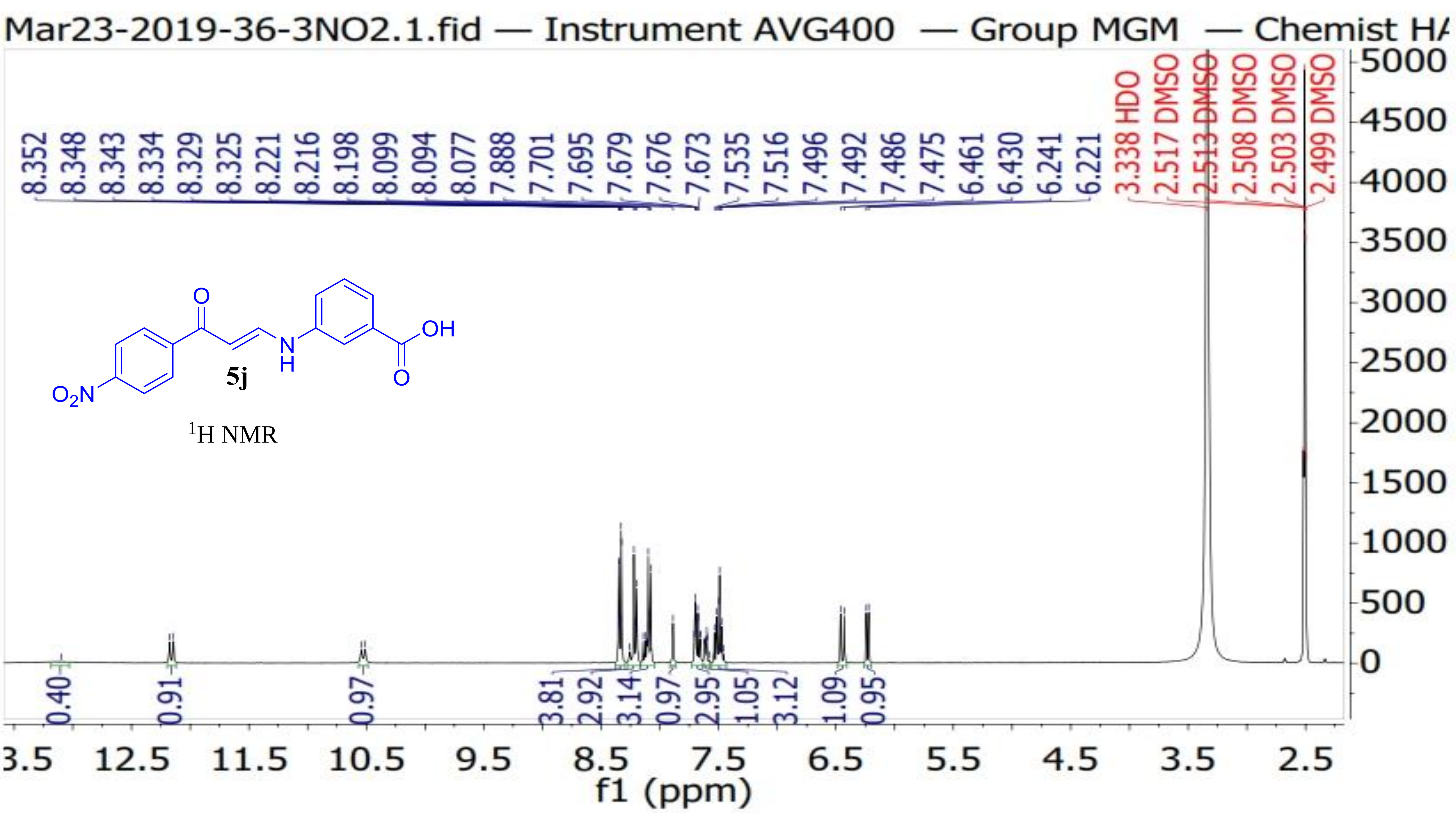

Mar23-2019-36-3NO2.1.fid — Instrument AVG400 — Group MGM — Chemist H/
8.352 8.348 8.343 8.334 8.329 8.325 8.221 8.216 8.198 8.099 8.094 8.077 7.888 7.701 7.695 7.679 7.676 7.673 7.535 7.516 7.496 7.492 7.486 7.475 6.461 6.430 6.241 6.221
3.338 HDO
2.517 DMSO
2.513 DMSO
2.508 DMSO
2.503 DMSO
2.499 DMSO
O
OH
N
H
O
O_2N
5j
^{1}H NMR
0.40 0.91 0.97 3.81 2.92 3.14 0.97 2.95 1.05 3.12 1.09 0.95
5000 4500 4000 3500 3000 2500 2000 1500 1000 500 0
3.5 12.5 11.5 10.5 9.5 8.5 7.5 6.5 5.5 4.5 3.5 2.5
f1 (ppm)

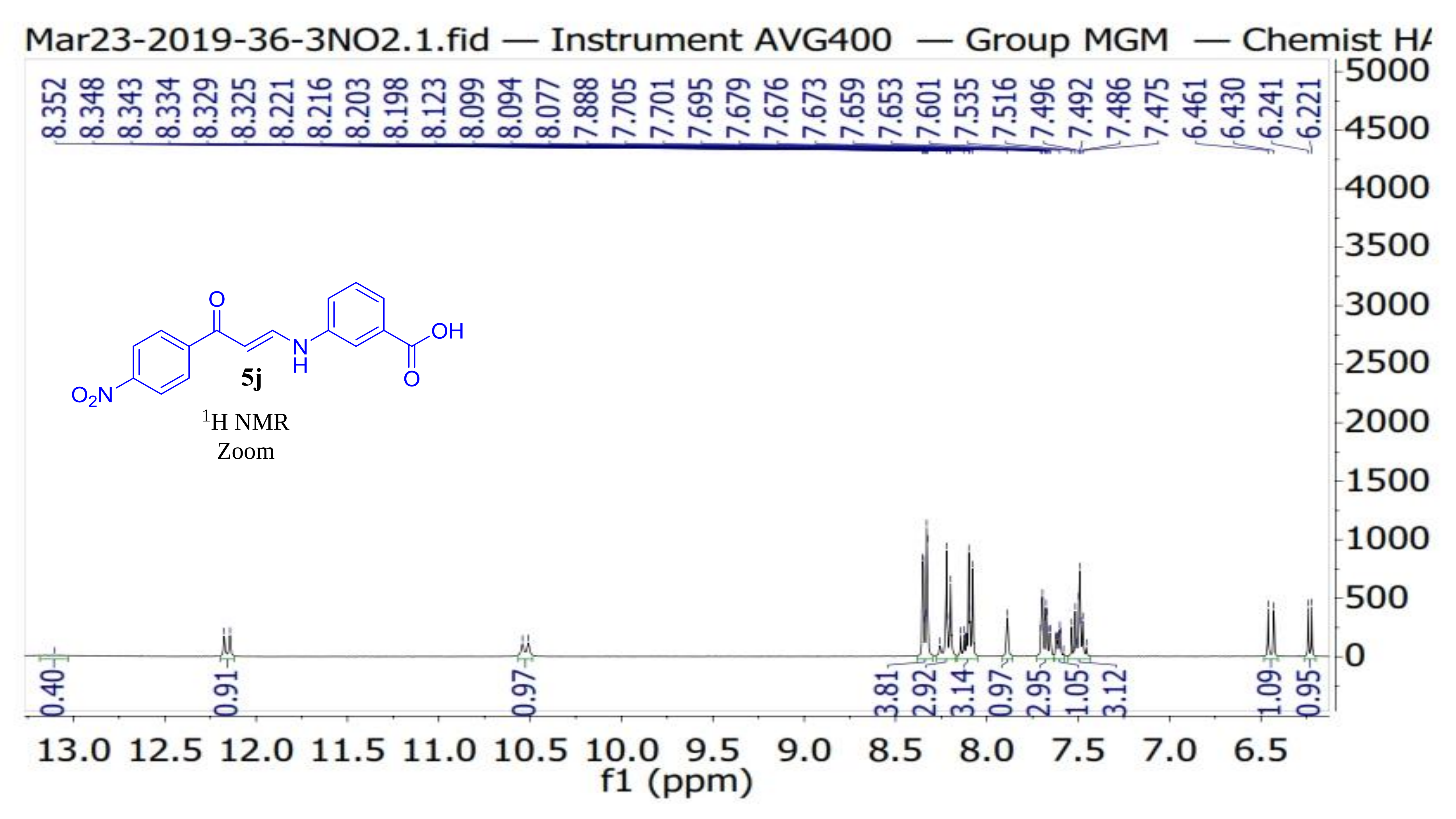

Mar23-2019-36-3NO2.1.fid — Instrument AVG400 — Group MGM — Chemist HA
8.352 8.348 8.343 8.334 8.329 8.325 8.221 8.216 8.203 8.198 8.123 8.099 8.094 8.077 7.888 7.705 7.701 7.695 7.679 7.676 7.673 7.659 7.653 7.601 7.535 7.516 7.496 7.492 7.486 7.475 6.461 6.430 6.241 6.221
O
OH
N
H
O
O_2N
5j
^{1}H NMR
Zoom
0.40 0.91 0.97 3.81 2.92 3.14 0.97 2.95 1.05 3.12 1.09 0.95
13.0 12.5 12.0 11.5 11.0 10.5 10.0 9.5 9.0 8.5 8.0 7.5 7.0 6.5
f1 (ppm)
5000 4500 4000 3500 3000 2500 2000 1500 1000 500 0

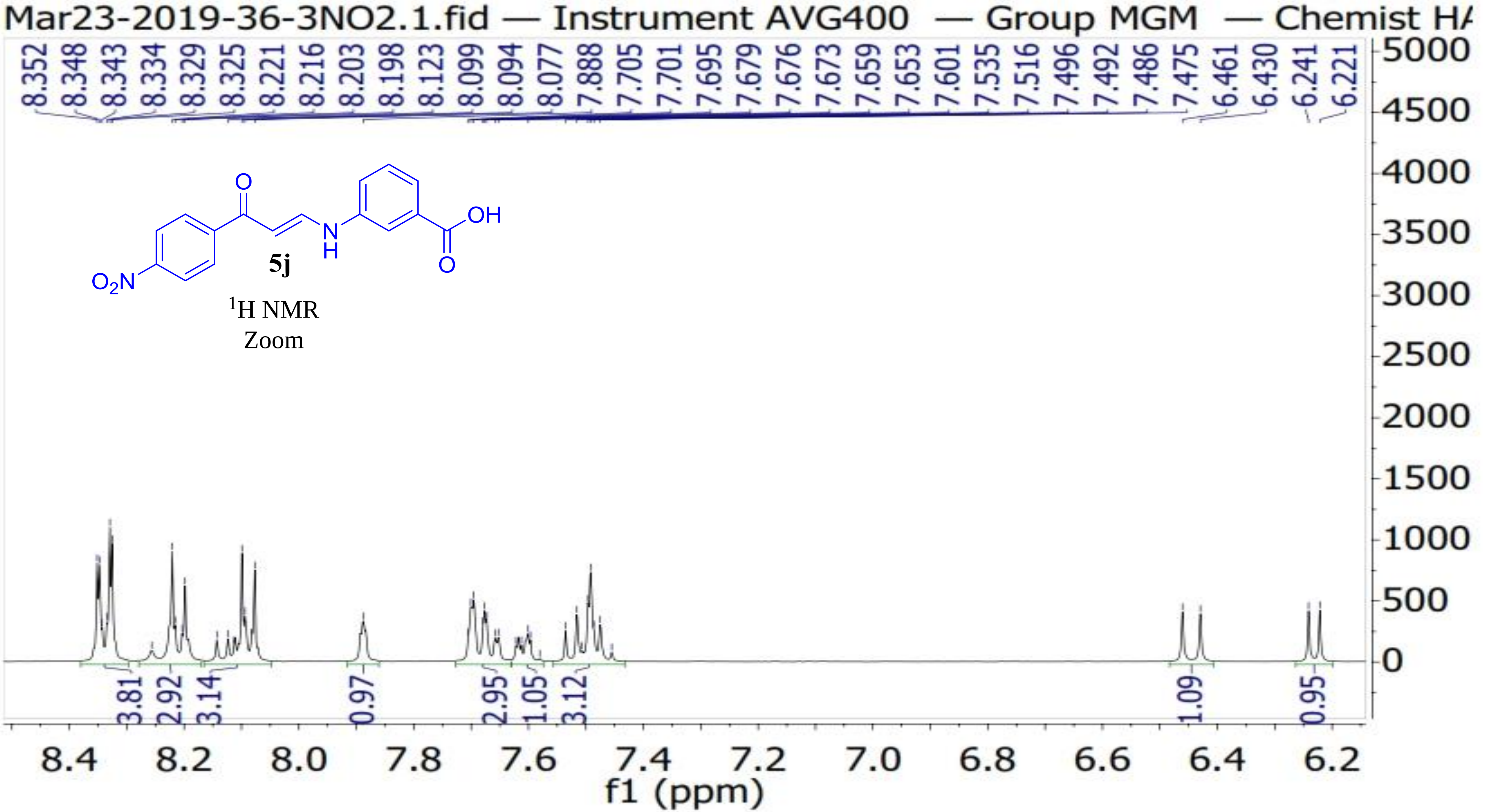
Mar23-2019-36-3NO2.1.fid — Instrument AVG400 — Group MGM — Chemist HA
8.352 8.348 8.343 8.334 8.329 8.325 8.221 8.216 8.203 8.198 8.123 8.099 8.094 8.077 7.888 7.705 7.701 7.695 7.679 7.676 7.673 7.659 7.653 7.601 7.535 7.516 7.496 7.492 7.486 7.475 6.461 6.430 6.241 6.221
O
OH
O
O2N
N
H
5j
1H NMR
Zoom
3.81 2.92 3.14 0.97 2.95 1.05 3.12 1.09 0.95
5000 4500 4000 3500 3000 2500 2000 1500 1000 500 0
8.4 8.2 8.0 7.8 7.6 7.4 7.2 7.0 6.8 6.6 6.4 6.2
f1 (ppm)

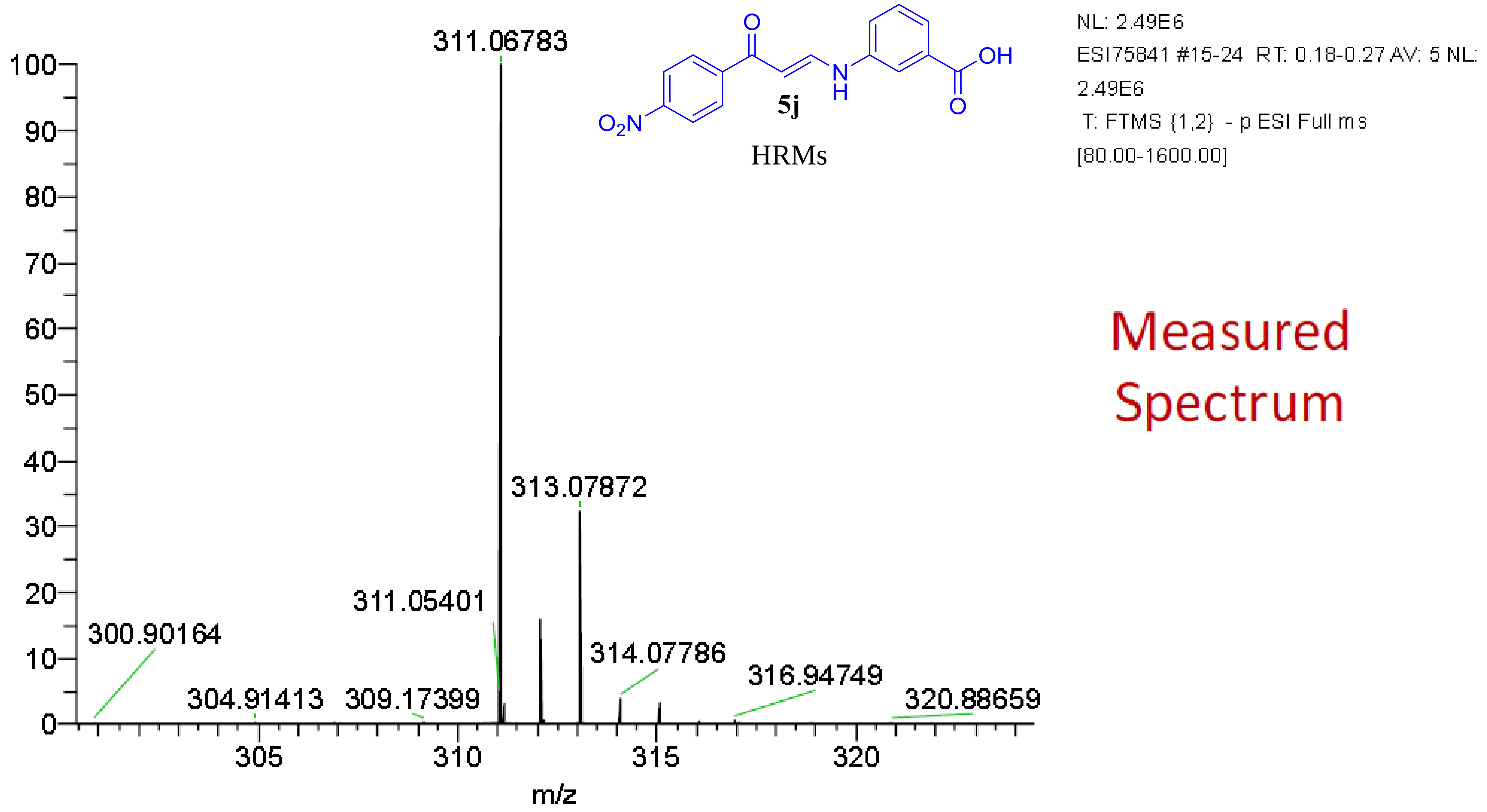

5j
HRMs
NL: 2.49E6
ESI75841 #15-24 RT: 0.18-0.27 AV: 5 NL: 2.49E6
T: FTMS {1,2} - p ESI Full ms [80.00-1600.00]
Measured Spectrum
311.06783
313.07872
311.05401
300.90164
314.07786
316.94749
304.91413
309.17399
320.88659
m/z

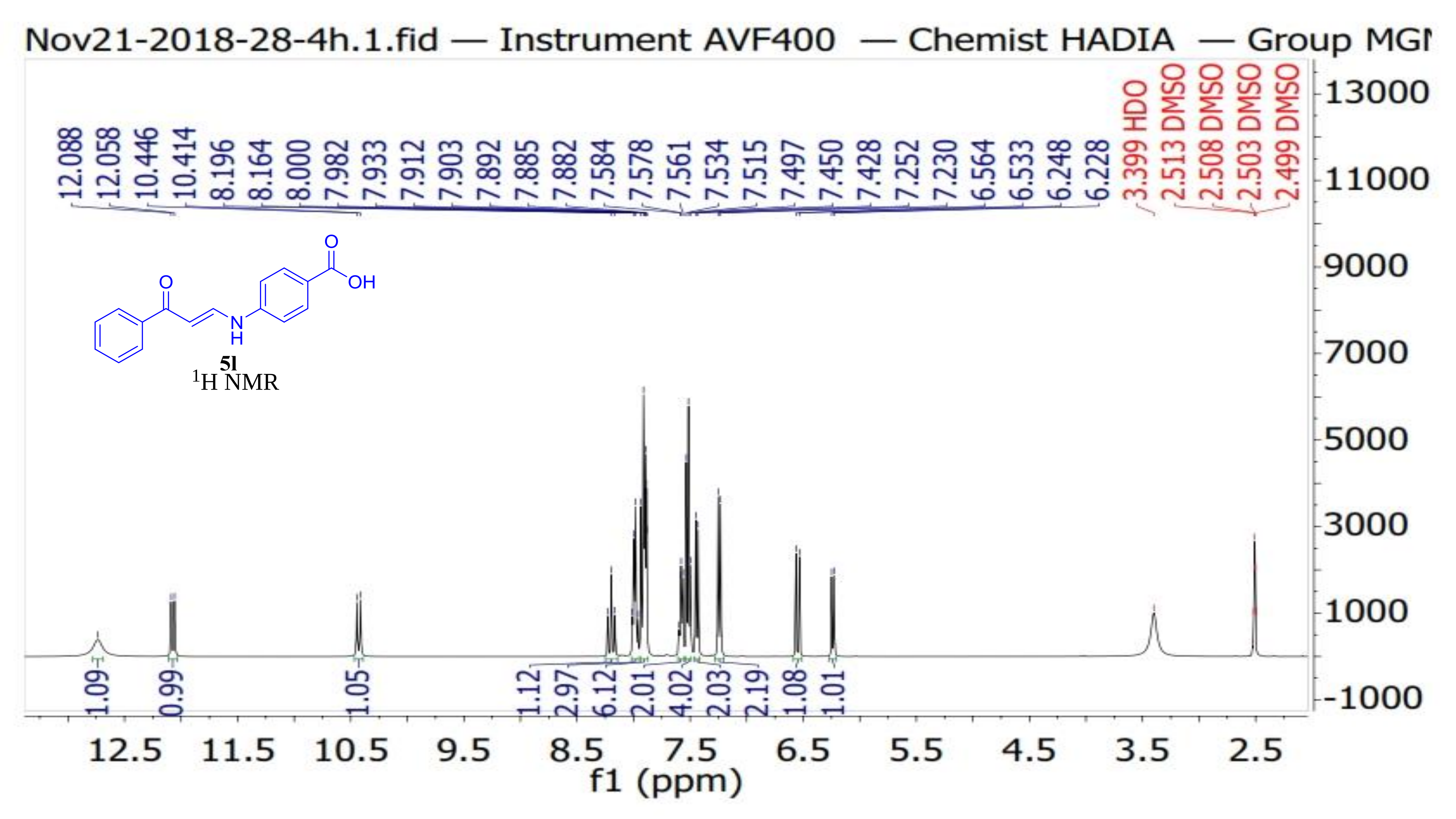
Nov21-2018-28-4h.1.fid — Instrument AVF400 — Group MGI — Chemist HADIA
12.088
12.058
10.446
10.414
8.196
8.164
8.000
7.982
7.933
7.912
7.903
7.892
7.885
7.882
7.584
7.578
7.561
7.534
7.515
7.497
7.450
7.428
7.252
7.230
6.564
6.533
6.248
6.228
3.399 HDO
2.513 DMSO
2.508 DMSO
2.503 DMSO
2.499 DMSO
O
OH
O
N
H
5l
1H NMR
1.09
0.99
1.05
1.12
2.97
6.12
2.01
4.02
2.03
2.19
1.08
1.01
12.5
11.5
10.5
9.5
8.5
7.5
6.5
5.5
4.5
3.5
2.5
f1 (ppm)
13000
11000
9000
7000
5000
3000
1000
-1000

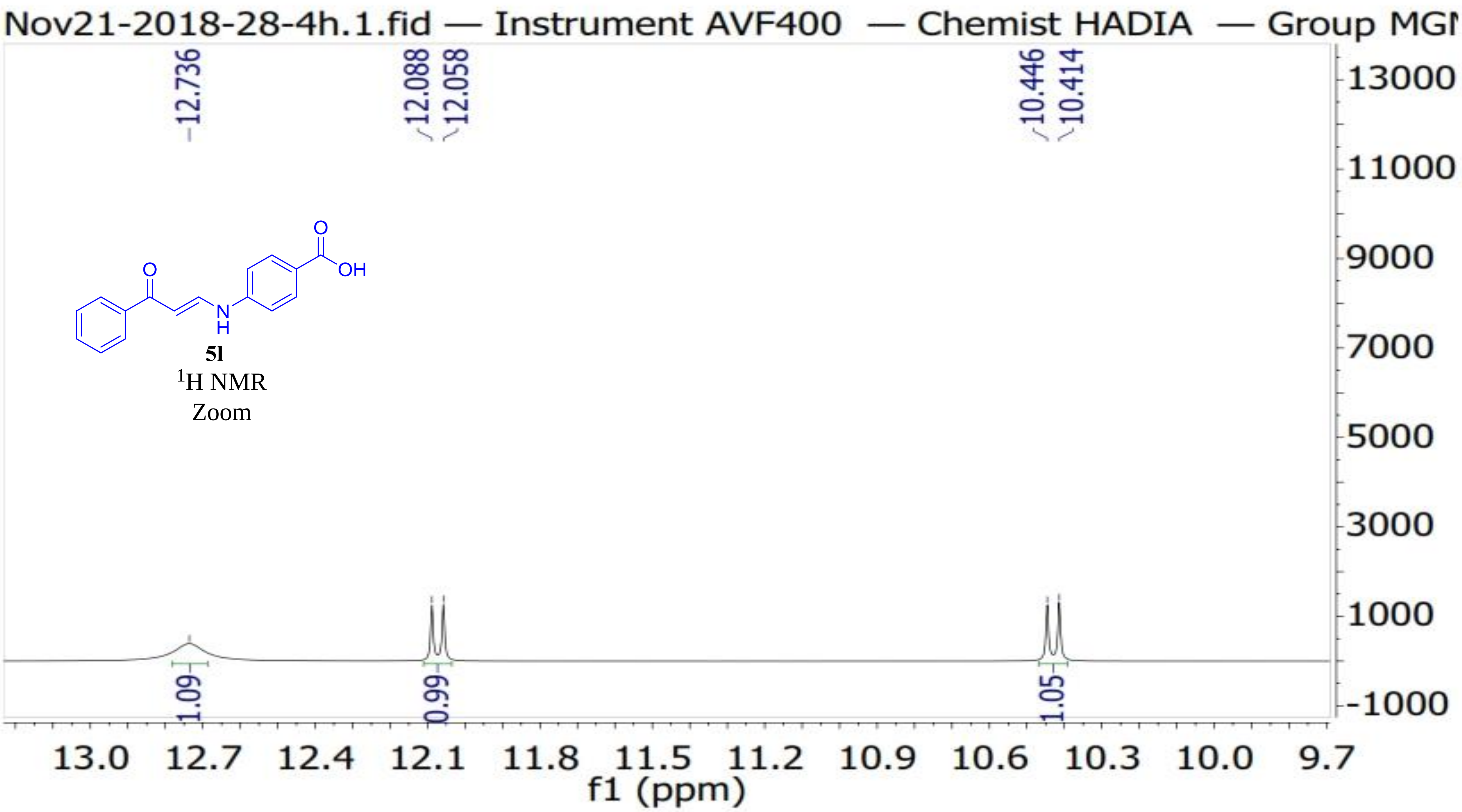

Nov21-2018-28-4h.1.fid — Instrument AVF400 — Chemist HADIA — Group MGI
12.736
12.088
12.058
10.446
10.414
O
OH
O
N
H
5l
1H NMR
Zoom
13000
11000
9000
7000
5000
3000
1000
-1000
1.09
0.99
1.05
13.0
12.7
12.4
12.1
11.8
11.5
11.2
10.9
10.6
10.3
10.0
9.7
f1 (ppm)

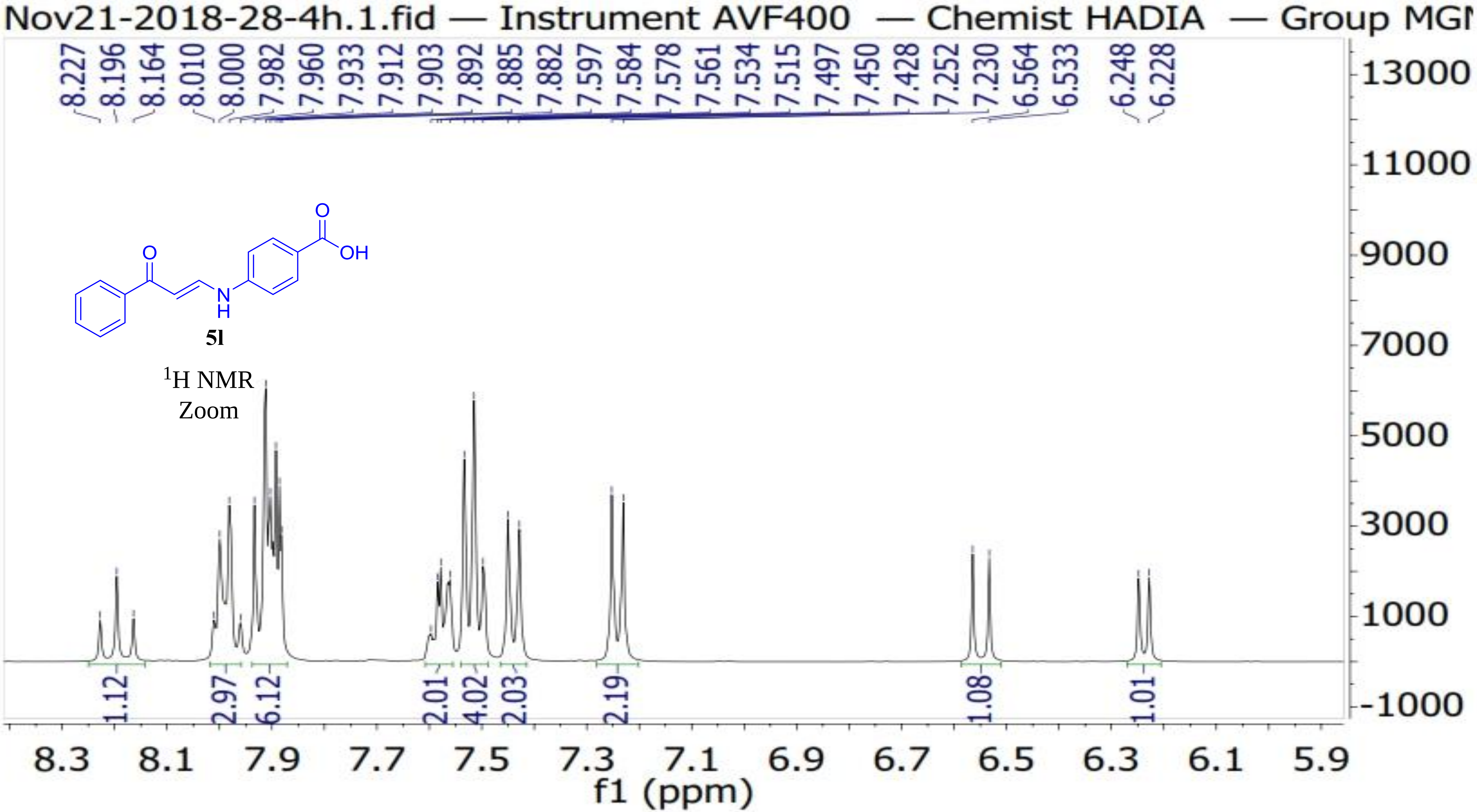

Nov21-2018-28-4h.1.fid — Instrument AVF400 — Chemist HADIA — Group MGI
8.227 8.196 8.164 8.010 8.000 7.982 7.960 7.933 7.912 7.903 7.892 7.885 7.882 7.597 7.584 7.578 7.561 7.534 7.515 7.497 7.450 7.428 7.252 7.230 6.564 6.533 6.248 6.228
O
OH
O
N
H
5l
1H NMR
Zoom
1.12 2.97 6.12 2.01 4.02 2.03 2.19 1.08 1.01
13000 11000 9000 7000 5000 3000 1000 -1000
8.3 8.1 7.9 7.7 7.5 7.3 7.1 6.9 6.7 6.5 6.3 6.1 5.9
f1 (ppm)

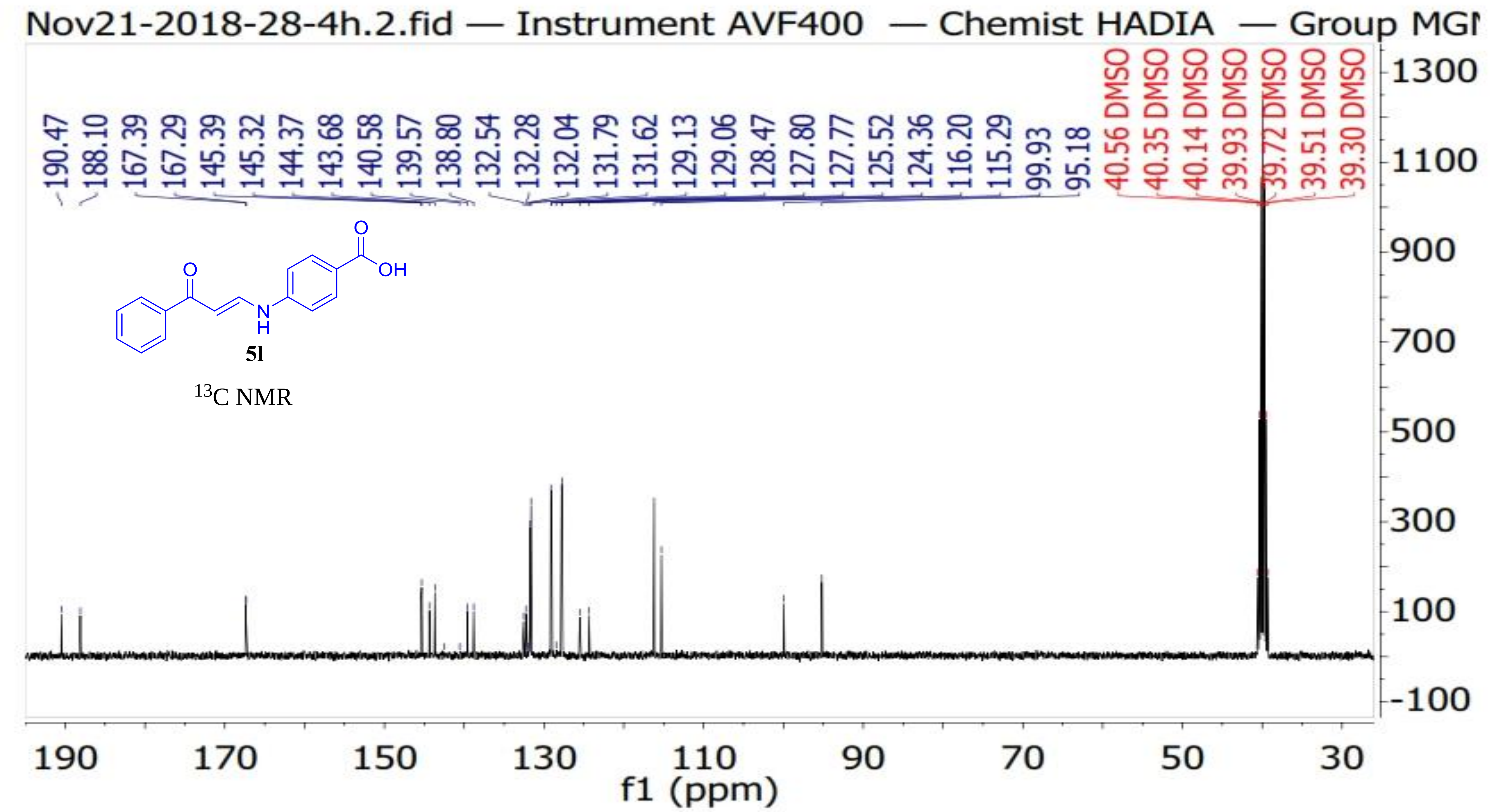
Nov21-2018-28-4h.2.fid — Instrument AVF400 — Chemist HADIA — Group MGI
190.47
188.10
167.39
167.29
145.39
145.32
144.37
143.68
140.58
139.57
138.80
132.54
132.28
132.04
131.79
131.62
129.13
129.06
128.47
127.80
127.77
125.52
124.36
116.20
115.29
99.93
95.18
40.56 DMSO
40.35 DMSO
40.14 DMSO
39.93 DMSO
39.72 DMSO
39.51 DMSO
39.30 DMSO
O
OH
O
N
H
5l
13C NMR
1300
1100
900
700
500
300
100
-100
190
170
150
130
110
90
70
50
30
f1 (ppm)

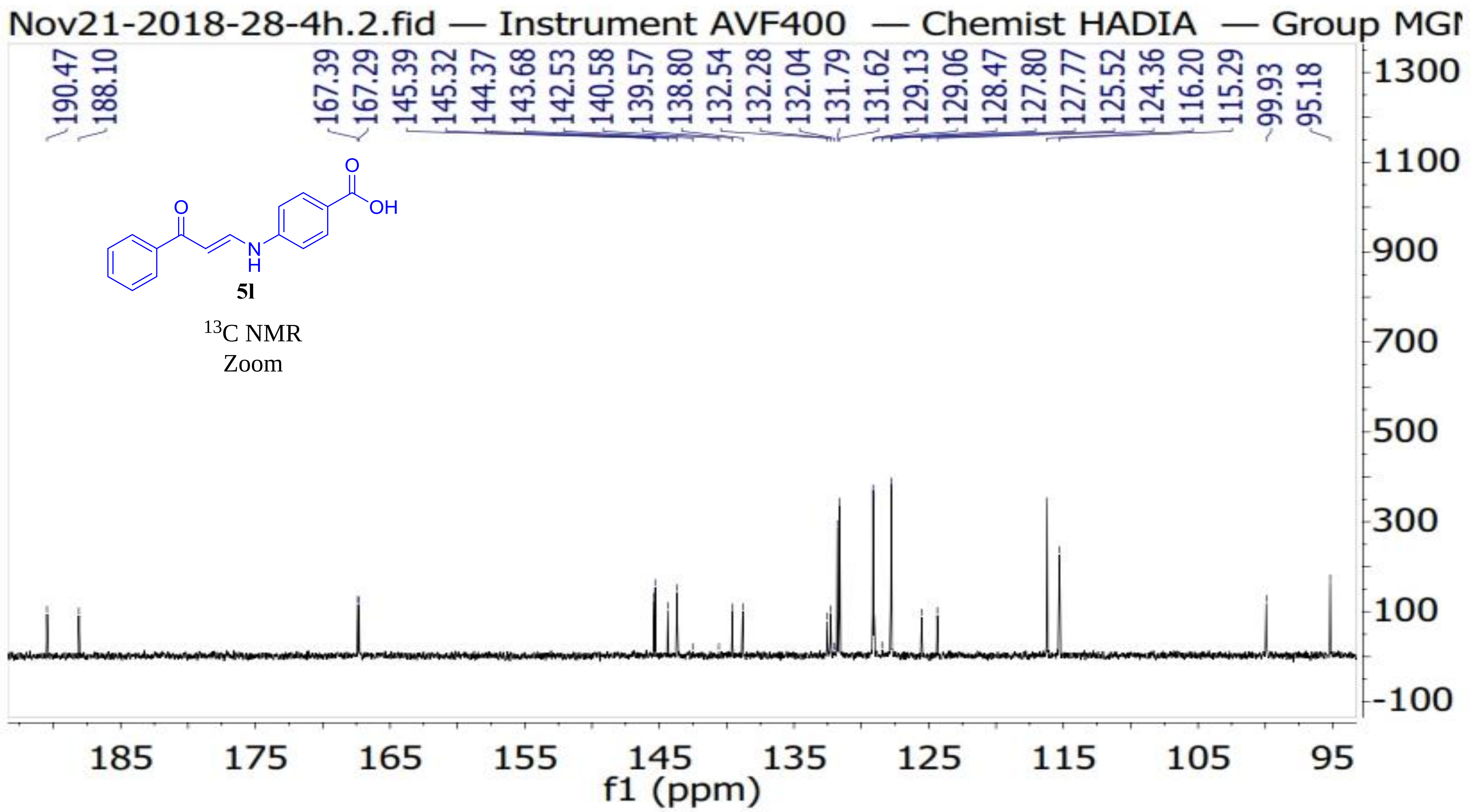

Nov21-2018-28-4h.2.fid — Instrument AVF400 — Chemist HADIA — Group MGI
190.47
188.10
167.39
167.29
145.39
145.32
144.37
143.68
142.53
140.58
139.57
138.80
132.54
132.28
132.04
131.79
131.62
129.13
129.06
128.47
127.80
127.77
125.52
124.36
116.20
115.29
99.93
95.18
O
OH
O
N
H
5l
13C NMR
Zoom
1300
1100
900
700
500
300
100
-100
185
175
165
155
145
135
125
115
105
95
f1 (ppm)

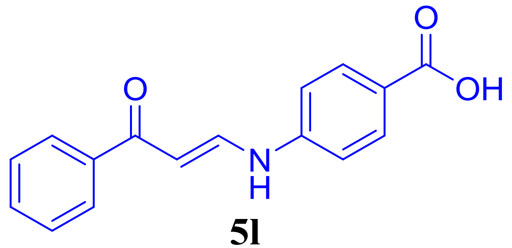

5l

HRMs

NL: 1.66E7
ESI75821 #13-28 RT: 0.15-0.3 AV: 8 NL: 3.02E+007
T: FTMS {1,1} + p ESI Full lock ms [80.00-1600.00]

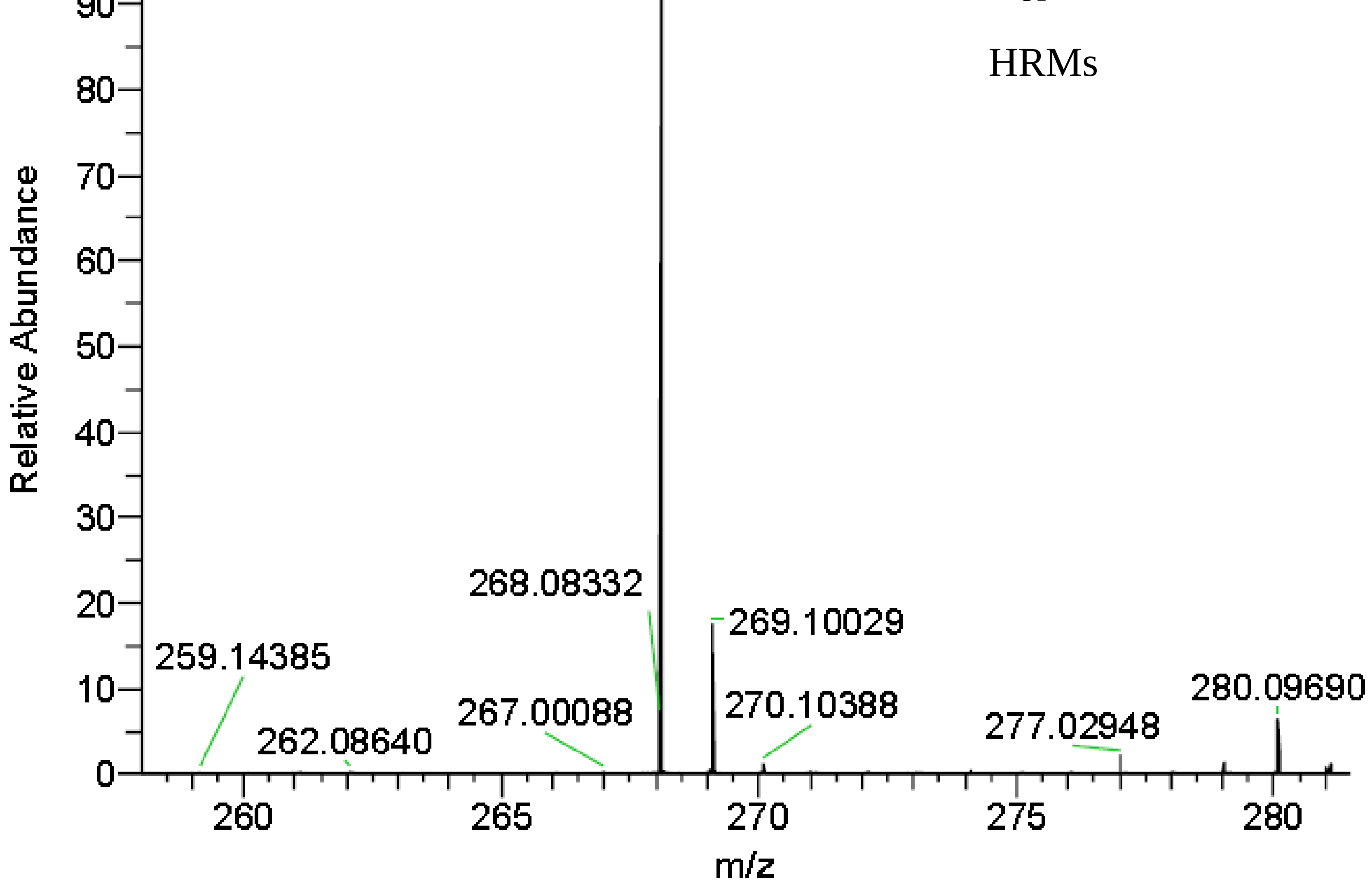

Measured Spectrum

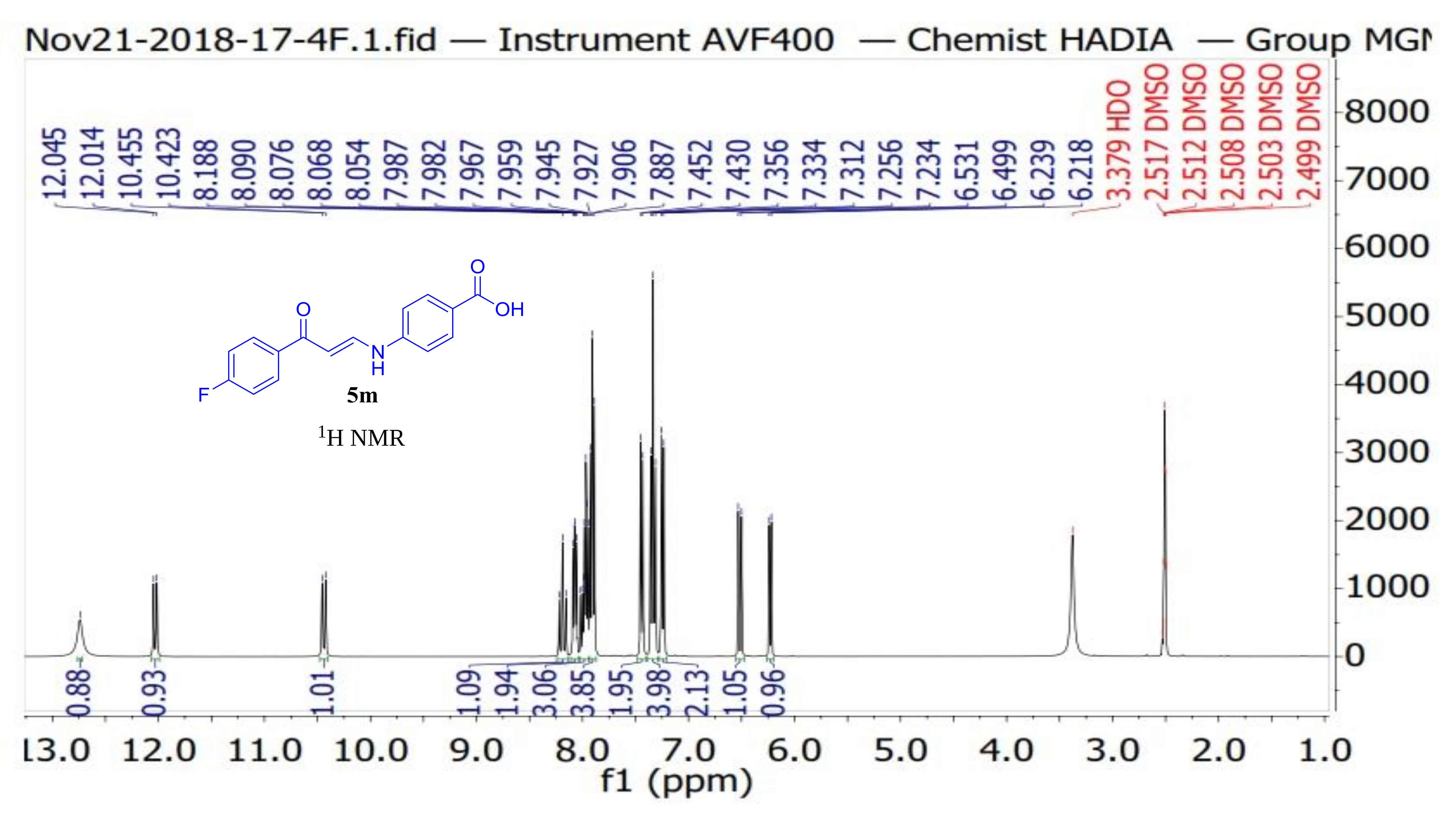

Nov21-2018-17-4F.1.fid — Instrument AVF400 — Chemist HADIA — Group MGN
12.045
12.014
10.455
10.423
8.188
8.090
8.076
8.068
8.054
7.987
7.982
7.967
7.959
7.945
7.927
7.906
7.887
7.452
7.430
7.356
7.334
7.312
7.256
7.234
6.531
6.499
6.239
6.218
3.379 HDO
2.517 DMSO
2.512 DMSO
2.508 DMSO
2.503 DMSO
2.499 DMSO
O
OH
O
N
H
F
5m
^{1}H NMR
0.88
0.93
1.01
1.09
1.94
3.06
3.85
1.95
3.98
2.13
1.05
0.96
f1 (ppm)

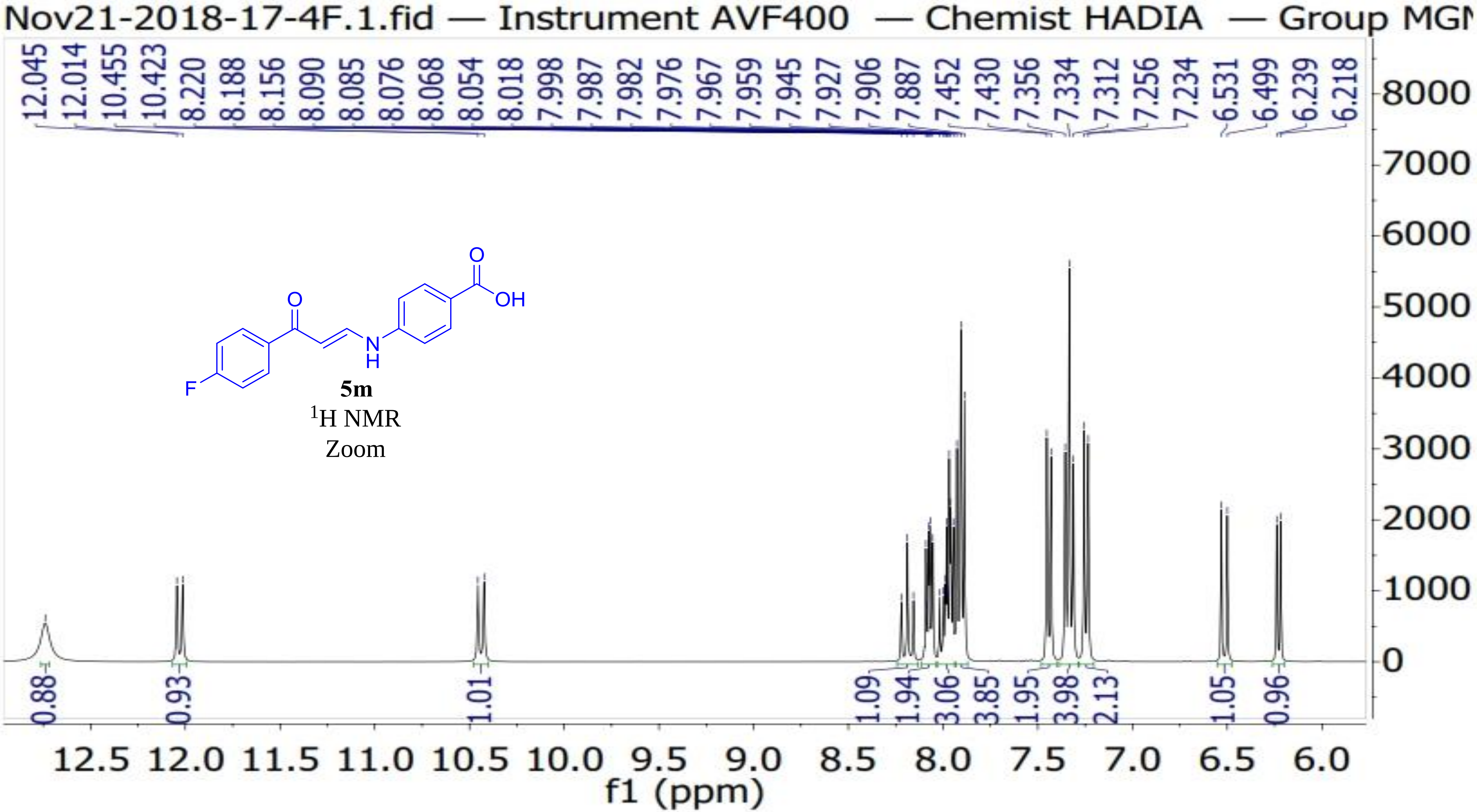
Nov21-2018-17-4F.1.fid — Instrument AVF400 — Chemist HADIA — Group MGN
12.045 12.014 10.455 10.423 8.220 8.188 8.156 8.090 8.085 8.076 8.068 8.054 8.018 7.998 7.987 7.982 7.976 7.967 7.959 7.945 7.927 7.906 7.887 7.452 7.430 7.356 7.334 7.312 7.256 7.234 6.531 6.499 6.239 6.218
O
OH
O
N
H
F
5m
1H NMR
Zoom
8000 7000 6000 5000 4000 3000 2000 1000 0
0.88 0.93 1.01 1.09 1.94 3.06 3.85 1.95 3.98 2.13 1.05 0.96
12.5 12.0 11.5 11.0 10.5 10.0 9.5 9.0 8.5 8.0 7.5 7.0 6.5 6.0
f1 (ppm)

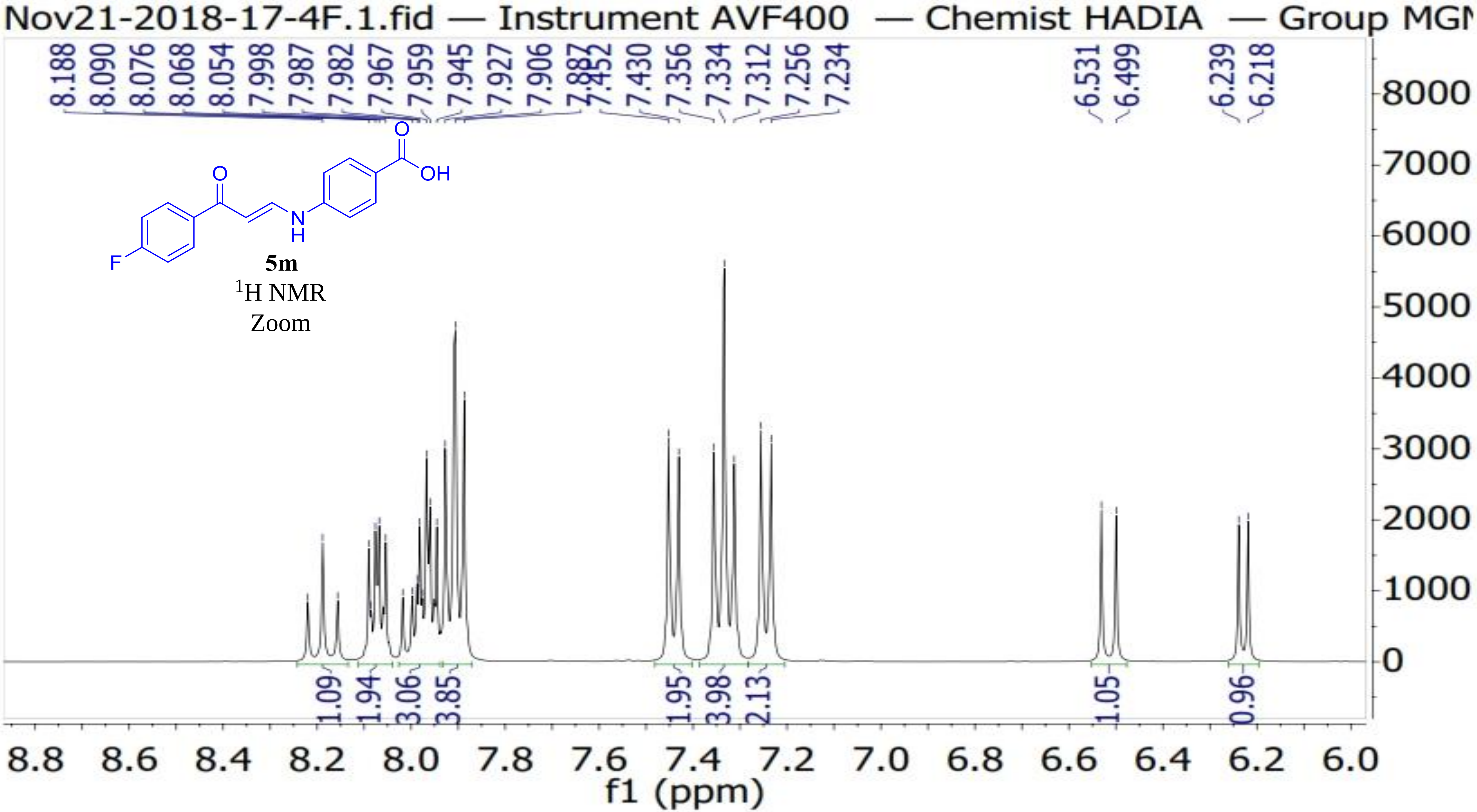

Nov21-2018-17-4F.1.fid — Instrument AVF400 — Chemist HADIA — Group MGN
8.188 8.090 8.076 8.068 8.054 7.998 7.987 7.982 7.967 7.959 7.945 7.927 7.906 7.887 7.452 7.430 7.356 7.334 7.312 7.256 7.234
6.531 6.499 6.239 6.218
5m
1H NMR
Zoom
1.09 1.94 3.06 3.85 1.95 3.98 2.13 1.05 0.96
8.8 8.6 8.4 8.2 8.0 7.8 7.6 7.4 7.2 7.0 6.8 6.6 6.4 6.2 6.0
f1 (ppm)
8000 7000 6000 5000 4000 3000 2000 1000 0

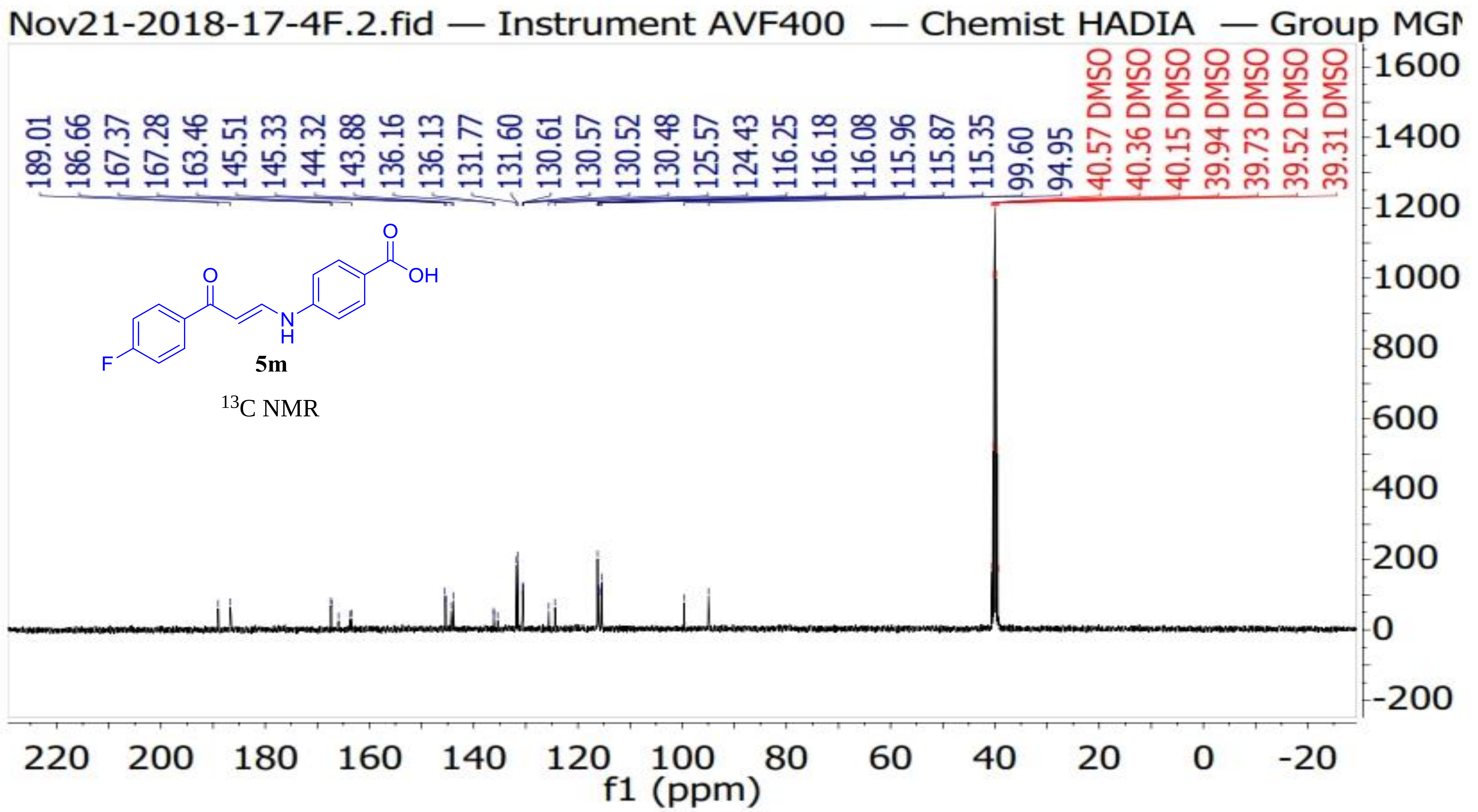
Nov21-2018-17-4F.2.fid — Instrument AVF400 — Chemist HADIA — Group MGN
189.01
186.66
167.37
167.28
163.46
145.51
145.33
144.32
143.88
136.16
136.13
131.77
131.60
130.61
130.57
130.52
130.48
125.57
124.43
116.25
116.18
116.08
115.96
115.87
115.35
99.60
94.95
40.57 DMSO
40.36 DMSO
40.15 DMSO
39.94 DMSO
39.73 DMSO
39.52 DMSO
39.31 DMSO
O
OH
O
N
H
F
5m
13C NMR
f1 (ppm)

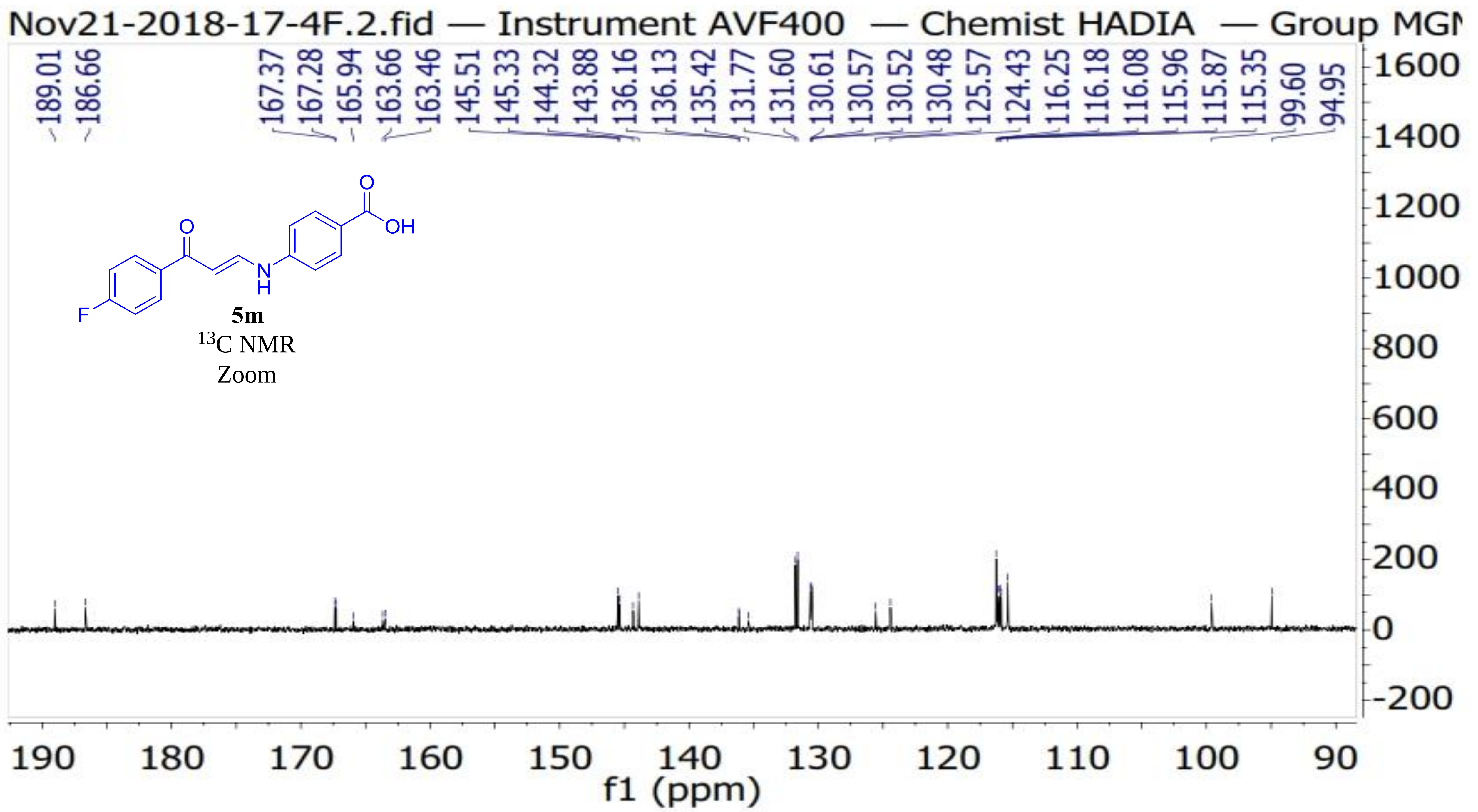
Nov21-2018-17-4F.2.fid — Instrument AVF400 — Chemist HADIA — Group MGI
189.01
186.66
167.37
167.28
165.94
163.66
163.46
145.51
145.33
144.32
143.88
136.16
136.13
135.42
131.77
131.60
130.61
130.57
130.52
130.48
125.57
124.43
116.25
116.18
116.08
115.96
115.87
115.35
99.60
94.95
O
OH
O
N
H
F
5m
13C NMR
Zoom
f1 (ppm)

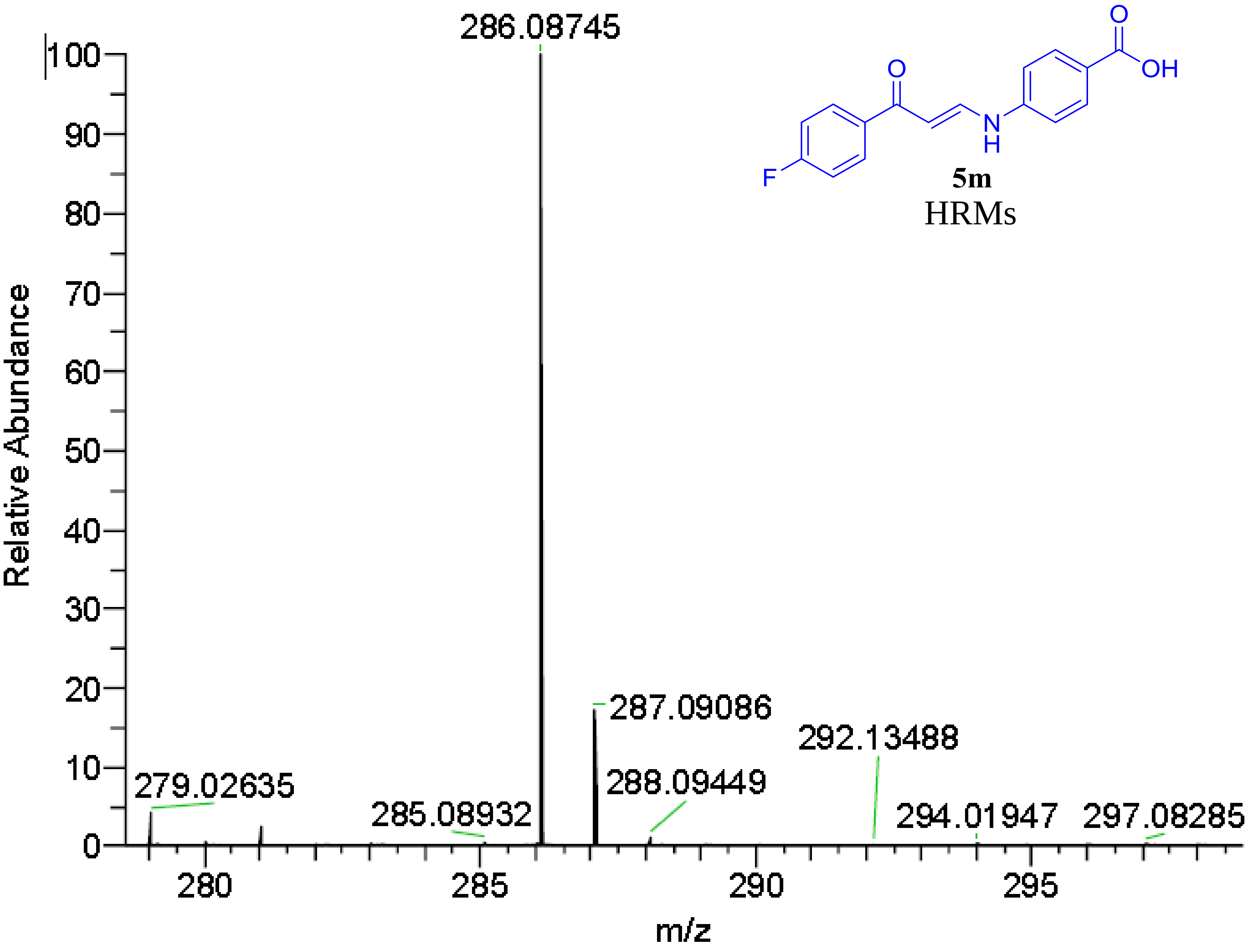

5m

HRMs

NL: 9.39E6
ESI75840 #13-28 RT: 0.14-0.3 AV: 8 NL: 3.62E+007
T: FTMS {1,1} + p ESI Full lock ms [80.00-1600.00]

Measured Spectrum

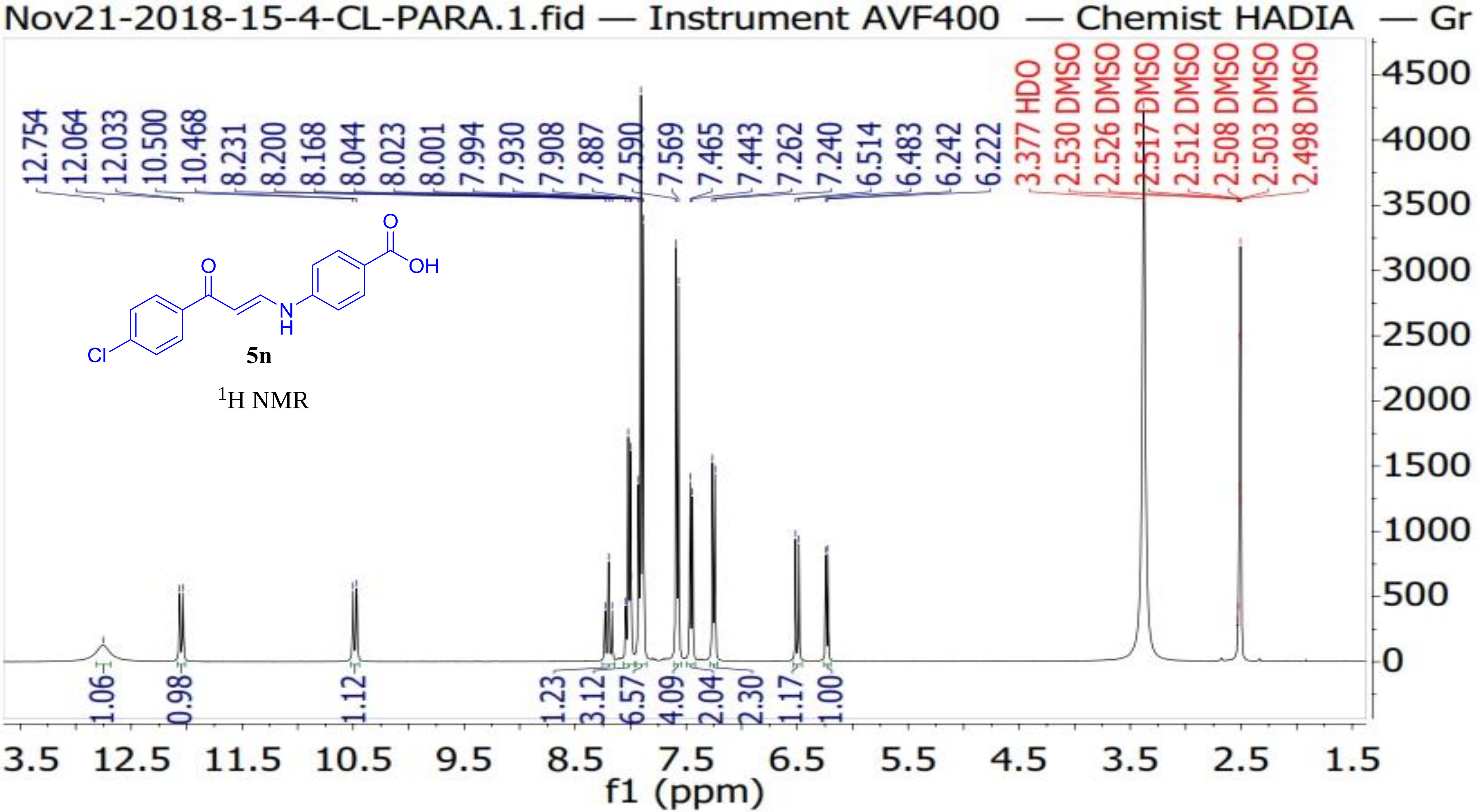
Nov21-2018-15-4-CL-PARA.1.fid — Instrument AVF400 — Chemist HADIA — Gr
12.754
12.064
12.033
10.500
10.468
8.231
8.200
8.168
8.044
8.023
8.001
7.994
7.930
7.908
7.887
7.590
7.569
7.465
7.443
7.262
7.240
6.514
6.483
6.242
6.222
3.377 HDO
2.530 DMSO
2.526 DMSO
2.517 DMSO
2.512 DMSO
2.508 DMSO
2.503 DMSO
2.498 DMSO
O
OH
O
N
H
Cl
5n
1H NMR
1.06
0.98
1.12
1.23
3.12
6.57
4.09
2.04
2.30
1.17
1.00
f1 (ppm)

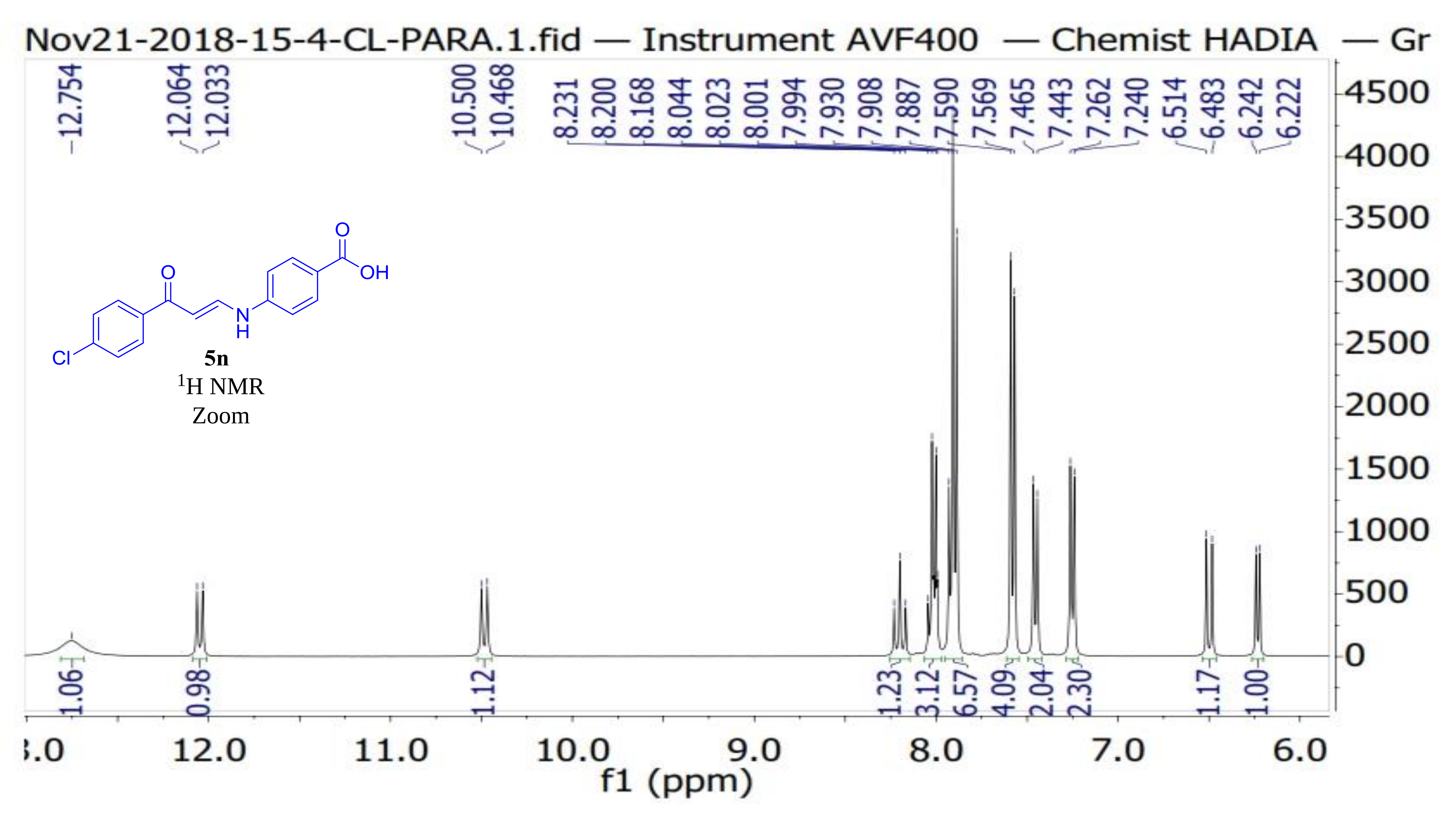

Nov21-2018-15-4-CL-PARA.1.fid — Instrument AVF400 — Chemist HADIA — Gr
12.754
12.064
12.033
10.500
10.468
8.231
8.200
8.168
8.044
8.023
8.001
7.994
7.930
7.908
7.887
7.590
7.569
7.465
7.443
7.262
7.240
6.514
6.483
6.242
6.222
O
OH
O
N
H
Cl
5n
1H NMR
Zoom
1.06
0.98
1.12
1.23
3.12
6.57
4.09
2.04
2.30
1.17
1.00
12.0
11.0
10.0
9.0
8.0
7.0
6.0
f1 (ppm)
4500
4000
3500
3000
2500
2000
1500
1000
500
0

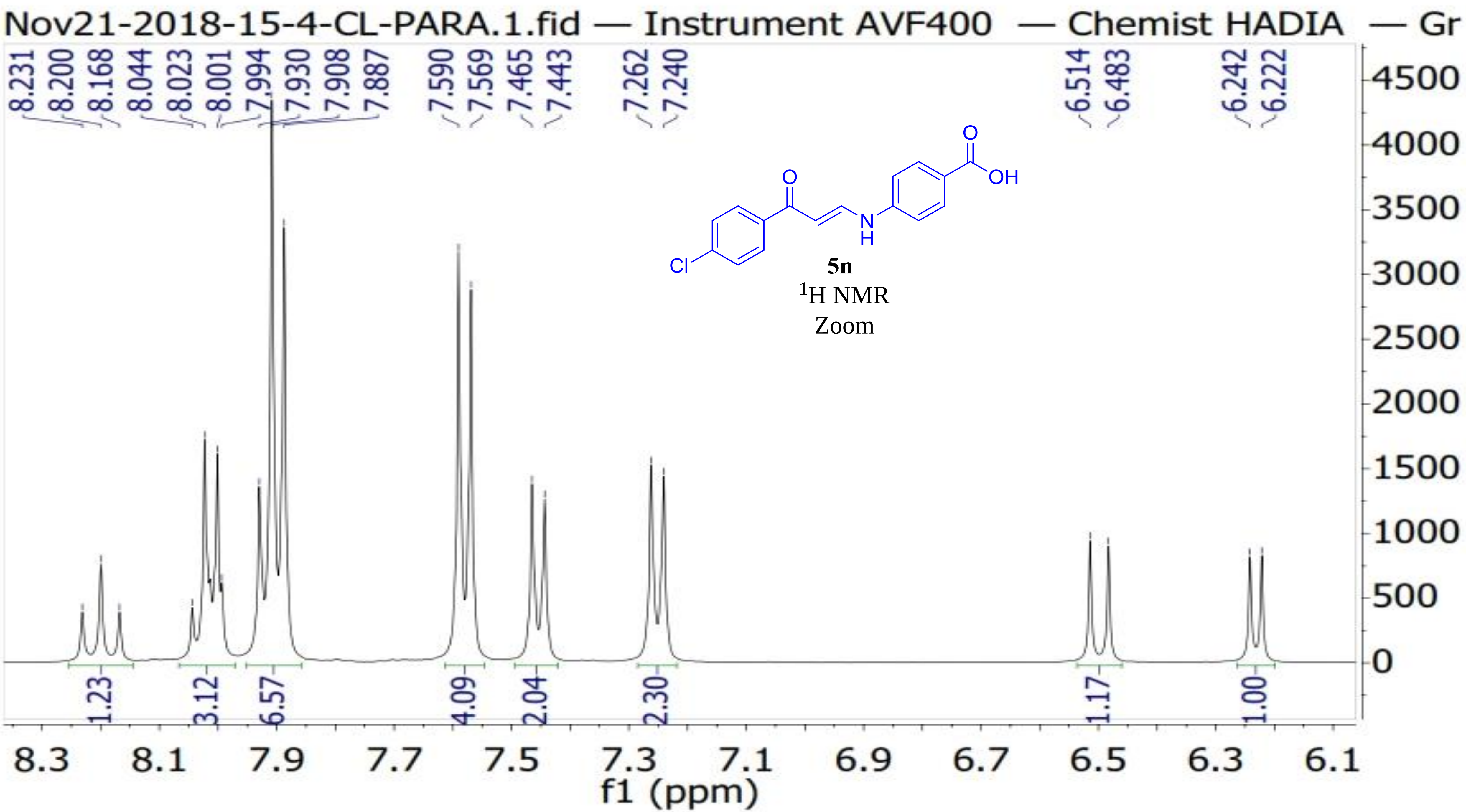

Nov21-2018-15-4-CL-PARA.1.fid — Instrument AVF400 — Chemist HADIA — Gr
8.231
8.200
8.168
8.044
8.023
8.001
7.994
7.930
7.908
7.887
7.590
7.569
7.465
7.443
7.262
7.240
6.514
6.483
6.242
6.222
O
OH
O
N
H
Cl
5n
1H NMR
Zoom
1.23
3.12
6.57
4.09
2.04
2.30
1.17
1.00
8.3
8.1
7.9
7.7
7.5
7.3
7.1
6.9
6.7
6.5
6.3
6.1
f1 (ppm)
4500
4000
3500
3000
2500
2000
1500
1000
500
0

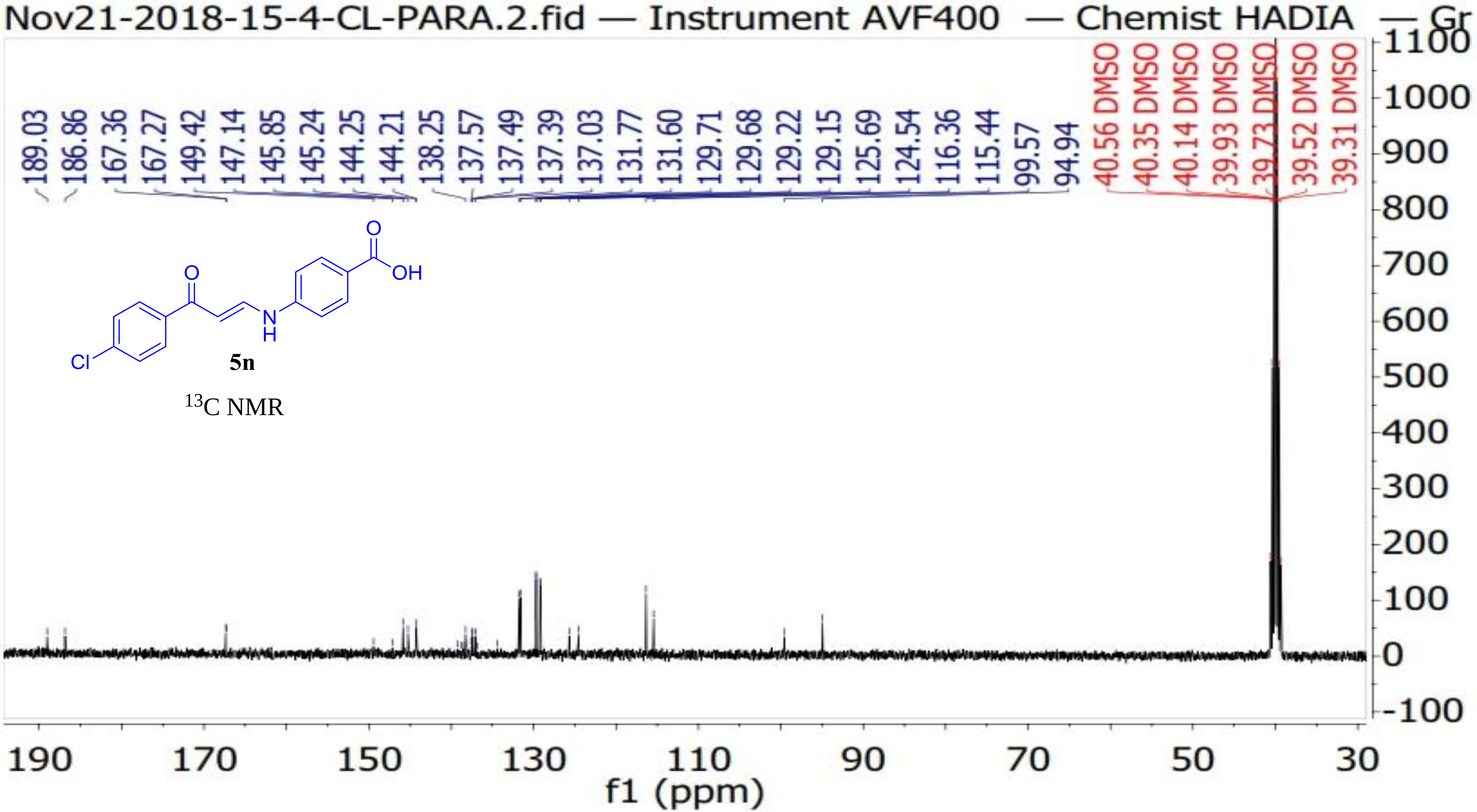

Nov21-2018-15-4-CL-PARA.2.fid — Instrument AVF400 — Chemist HADIA — Gr
189.03
186.86
167.36
167.27
149.42
147.14
145.85
145.24
144.25
144.21
138.25
137.57
137.49
137.39
137.03
131.77
131.60
129.71
129.68
129.22
129.15
125.69
124.54
116.36
115.44
99.57
94.94
40.56 DMSO
40.35 DMSO
40.14 DMSO
39.93 DMSO
39.73 DMSO
39.52 DMSO
39.31 DMSO
O
OH
O
N
H
Cl
5n
13C NMR
f1 (ppm)

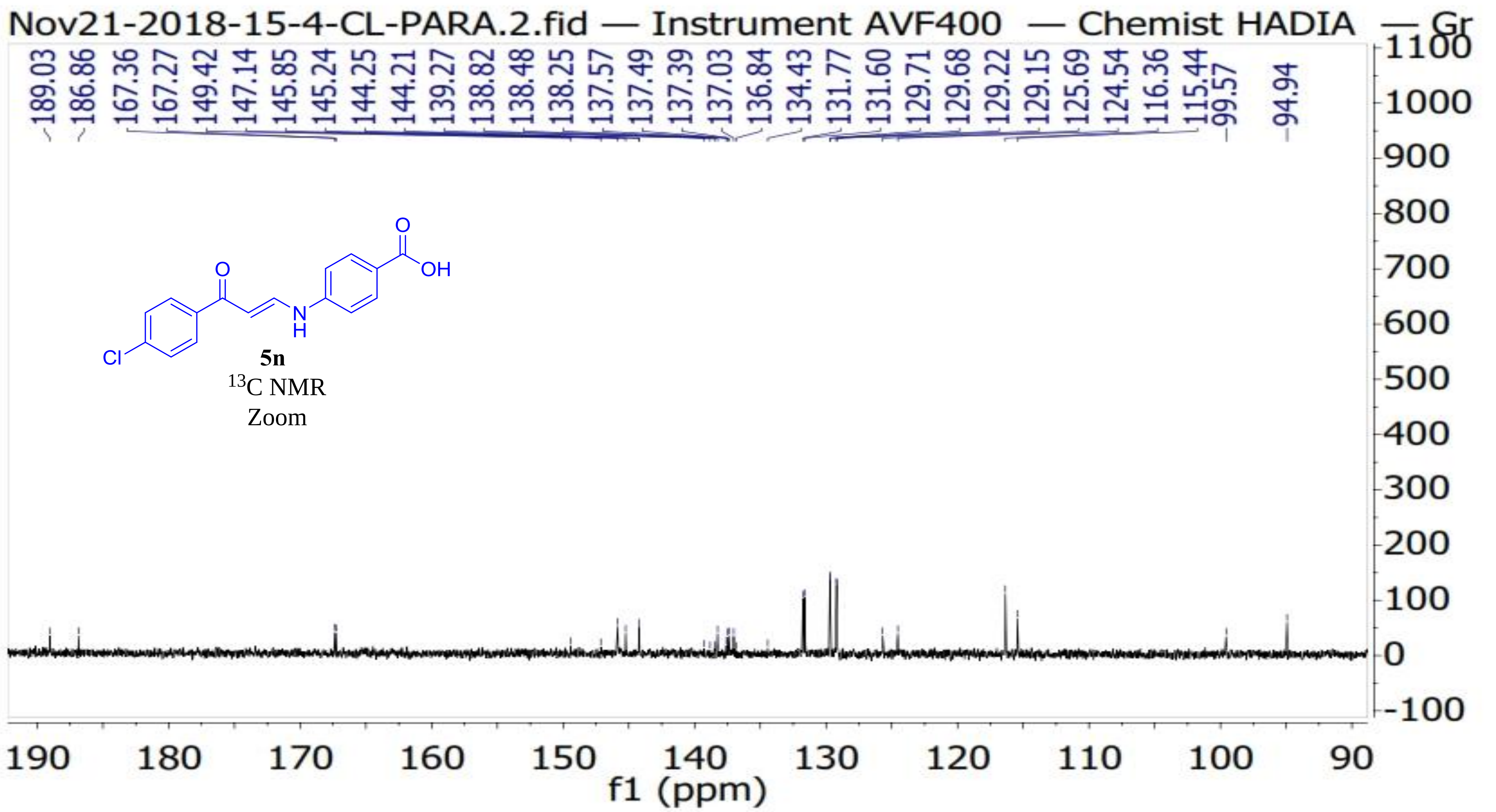
Nov21-2018-15-4-CL-PARA.2.fid — Instrument AVF400 — Chemist HADIA — Gr
189.03
186.86
167.36
167.27
149.42
147.14
145.85
145.24
144.25
144.21
139.27
138.82
138.48
138.25
137.57
137.49
137.39
137.03
136.84
134.43
131.77
131.60
129.71
129.68
129.22
129.15
125.69
124.54
116.36
115.44
99.57
94.94
O
OH
O
N
H
Cl
5n
13C NMR
Zoom
1100
1000
900
800
700
600
500
400
300
200
100
0
-100
190
180
170
160
150
140
130
120
110
100
90
f1 (ppm)

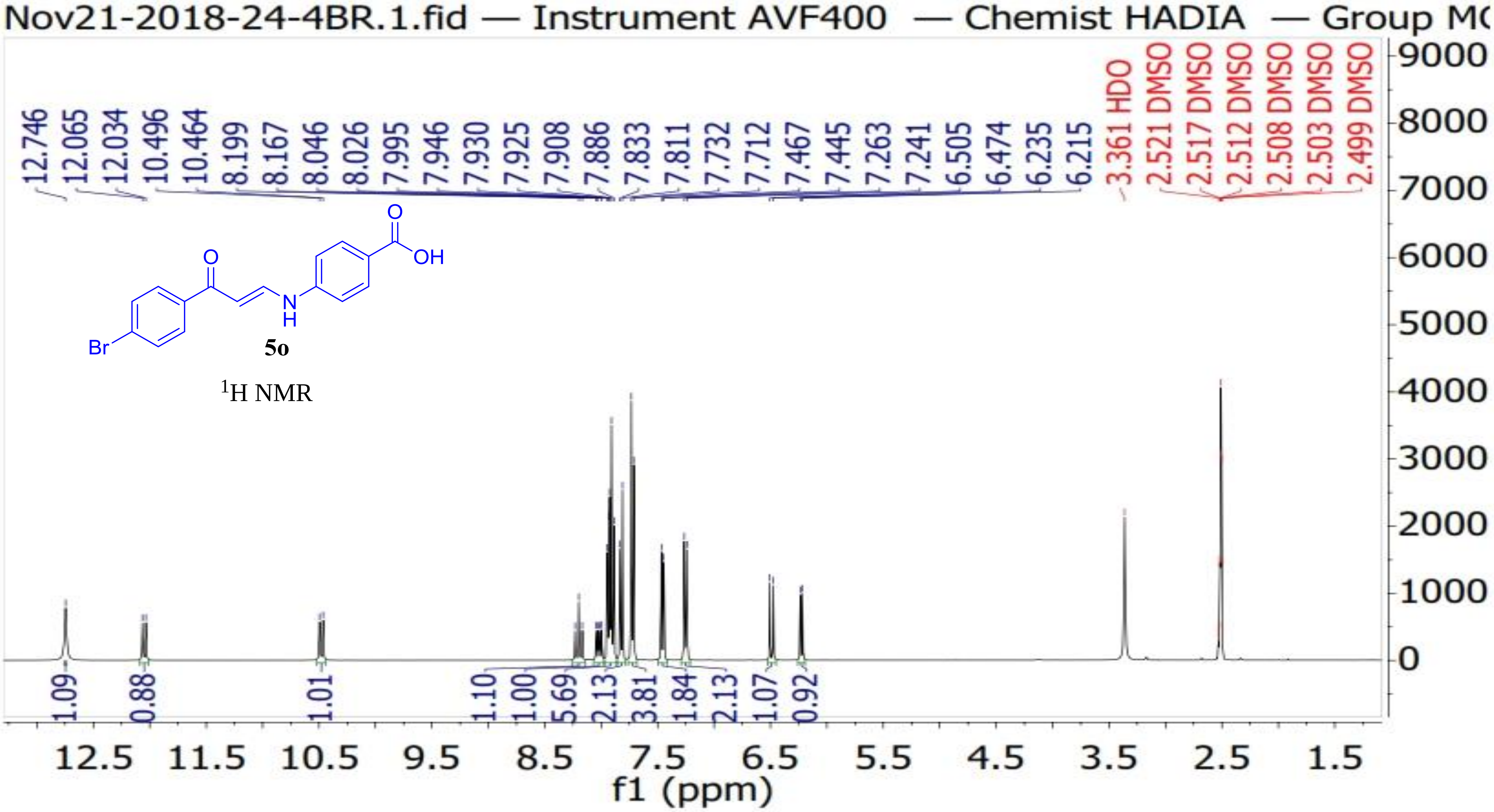
Nov21-2018-24-4BR.1.fid — Instrument AVF400 — Chemist HADIA — Group MC
12.746
12.065
12.034
10.496
10.464
8.199
8.167
8.046
8.026
7.995
7.946
7.930
7.925
7.908
7.886
7.833
7.811
7.732
7.712
7.467
7.445
7.263
7.241
6.505
6.474
6.235
6.215
3.361 HDO
2.521 DMSO
2.517 DMSO
2.512 DMSO
2.508 DMSO
2.503 DMSO
2.499 DMSO
O
OH
O
N
H
Br
5o
1H NMR
1.09
0.88
1.01
1.10
1.00
5.69
2.13
3.81
1.84
2.13
1.07
0.92
9000
8000
7000
6000
5000
4000
3000
2000
1000
0
12.5
11.5
10.5
9.5
8.5
7.5
6.5
5.5
4.5
3.5
2.5
1.5
f1 (ppm)

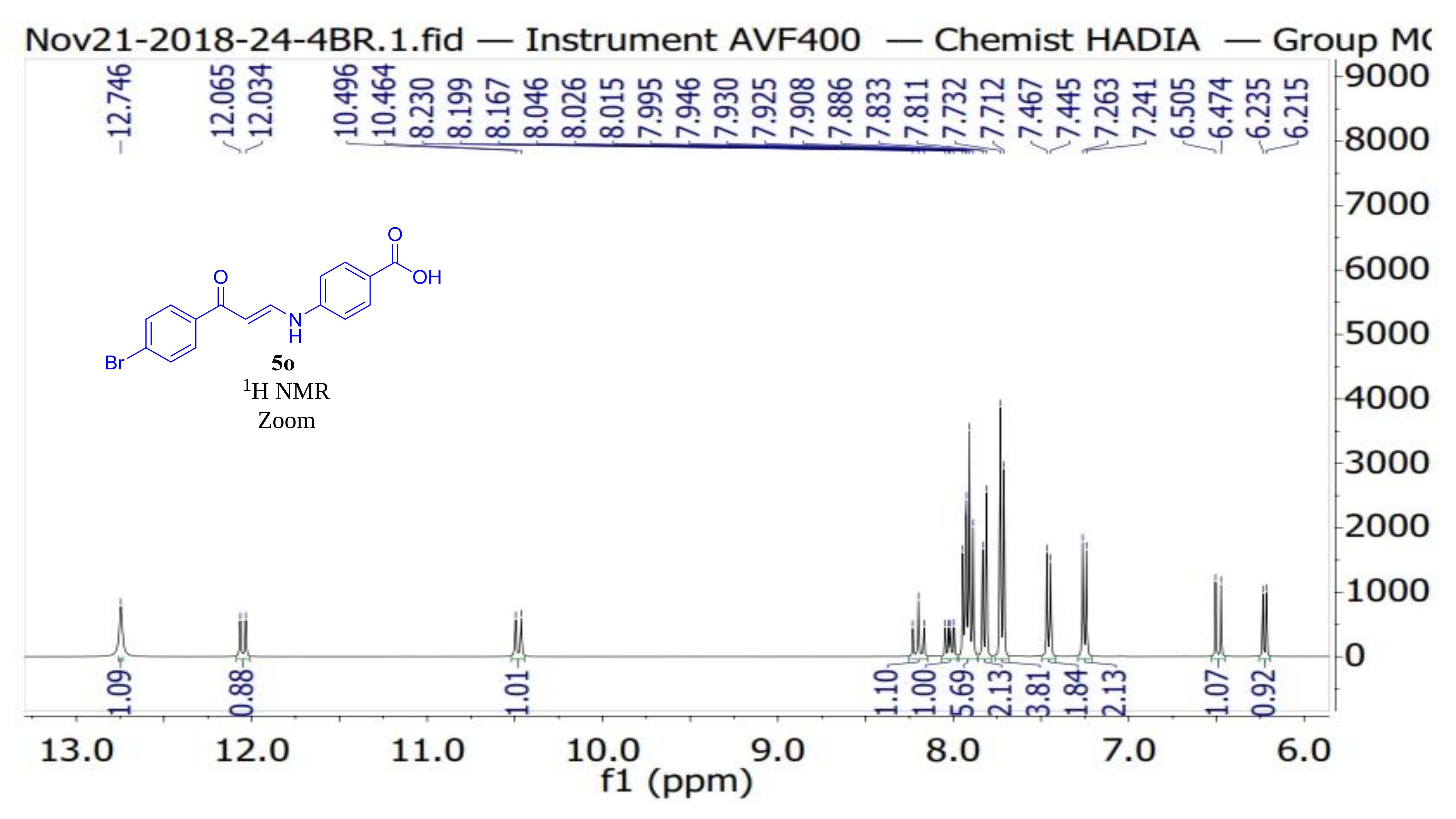

Nov21-2018-24-4BR.1.fid — Instrument AVF400 — Chemist HADIA — Group MC
5o
1H NMR
Zoom
f1 (ppm)

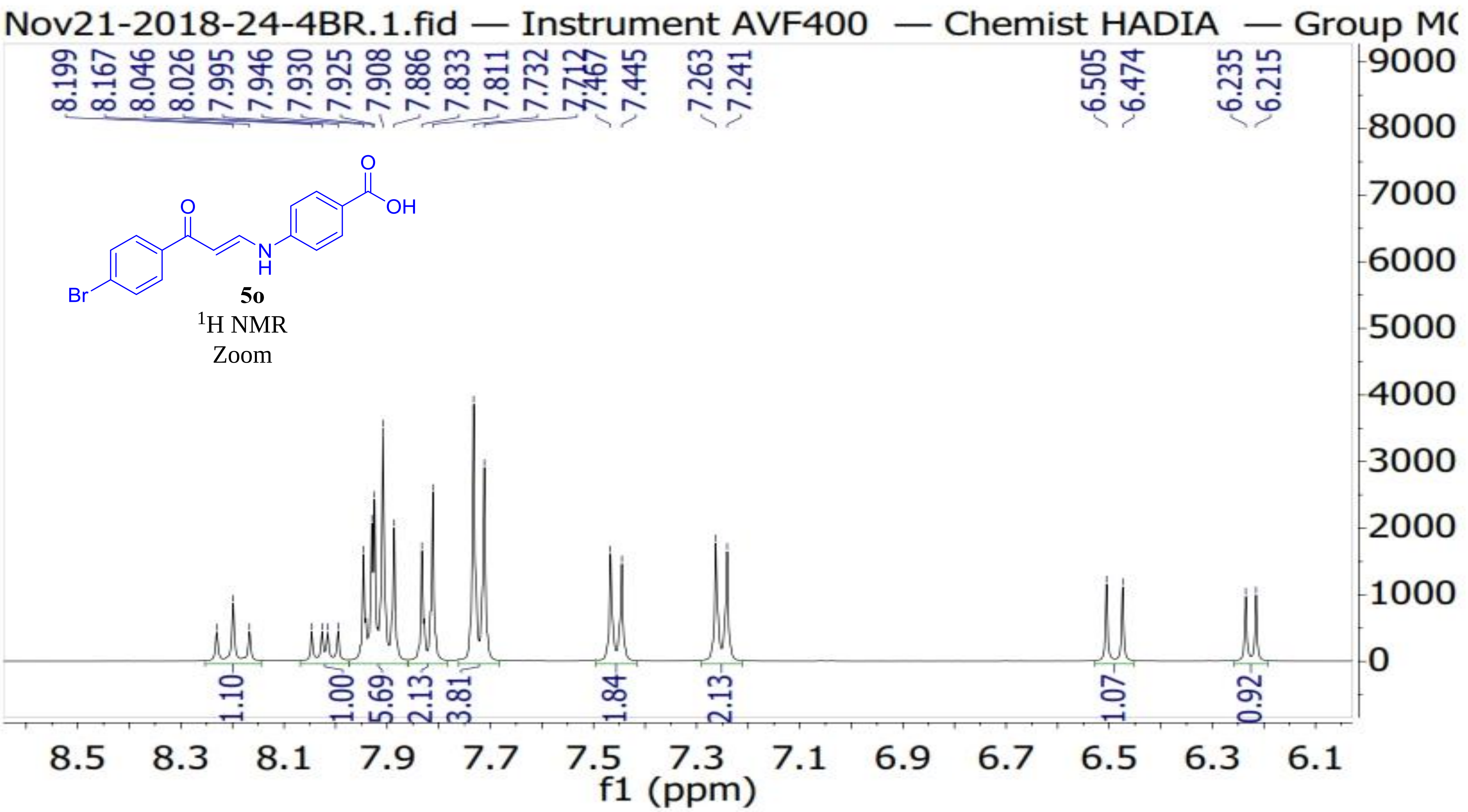

Nov21-2018-24-4BR.1.fid — Instrument AVF400 — Chemist HADIA — Group MC
8.199 8.167 8.046 8.026 7.995 7.946 7.930 7.925 7.908 7.886 7.833 7.811 7.732 7.712 7.467 7.445 7.263 7.241 6.505 6.474 6.235 6.215
Br
O
N
H
OH
5o
1H NMR
Zoom
1.10 1.00 5.69 2.13 3.81 1.84 2.13 1.07 0.92
9000 8000 7000 6000 5000 4000 3000 2000 1000 0
8.5 8.3 8.1 7.9 7.7 7.5 7.3 7.1 6.9 6.7 6.5 6.3 6.1
f1 (ppm)

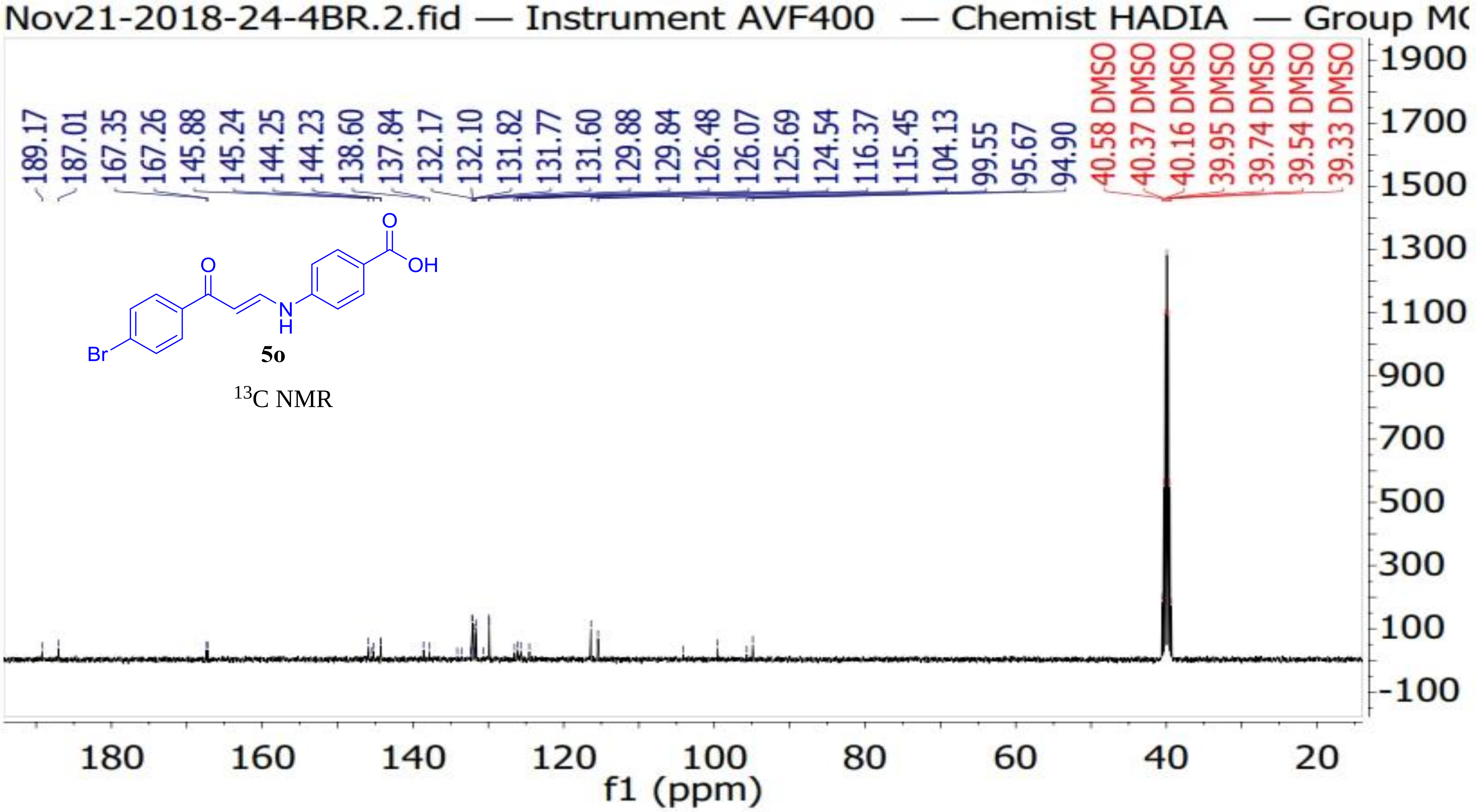
Nov21-2018-24-4BR.2.fid — Instrument AVF400 — Chemist HADIA — Group MC
189.17
187.01
167.35
167.26
145.88
145.24
144.25
144.23
138.60
137.84
132.17
132.10
131.82
131.77
131.60
129.88
129.84
126.48
126.07
125.69
124.54
116.37
115.45
104.13
99.55
95.67
94.90
40.58 DMSO
40.37 DMSO
40.16 DMSO
39.95 DMSO
39.74 DMSO
39.54 DMSO
39.33 DMSO
O
OH
O
N
H
Br
5o
13C NMR
1900
1700
1500
1300
1100
900
700
500
300
100
-100
180
160
140
120
100
80
60
40
20
f1 (ppm)

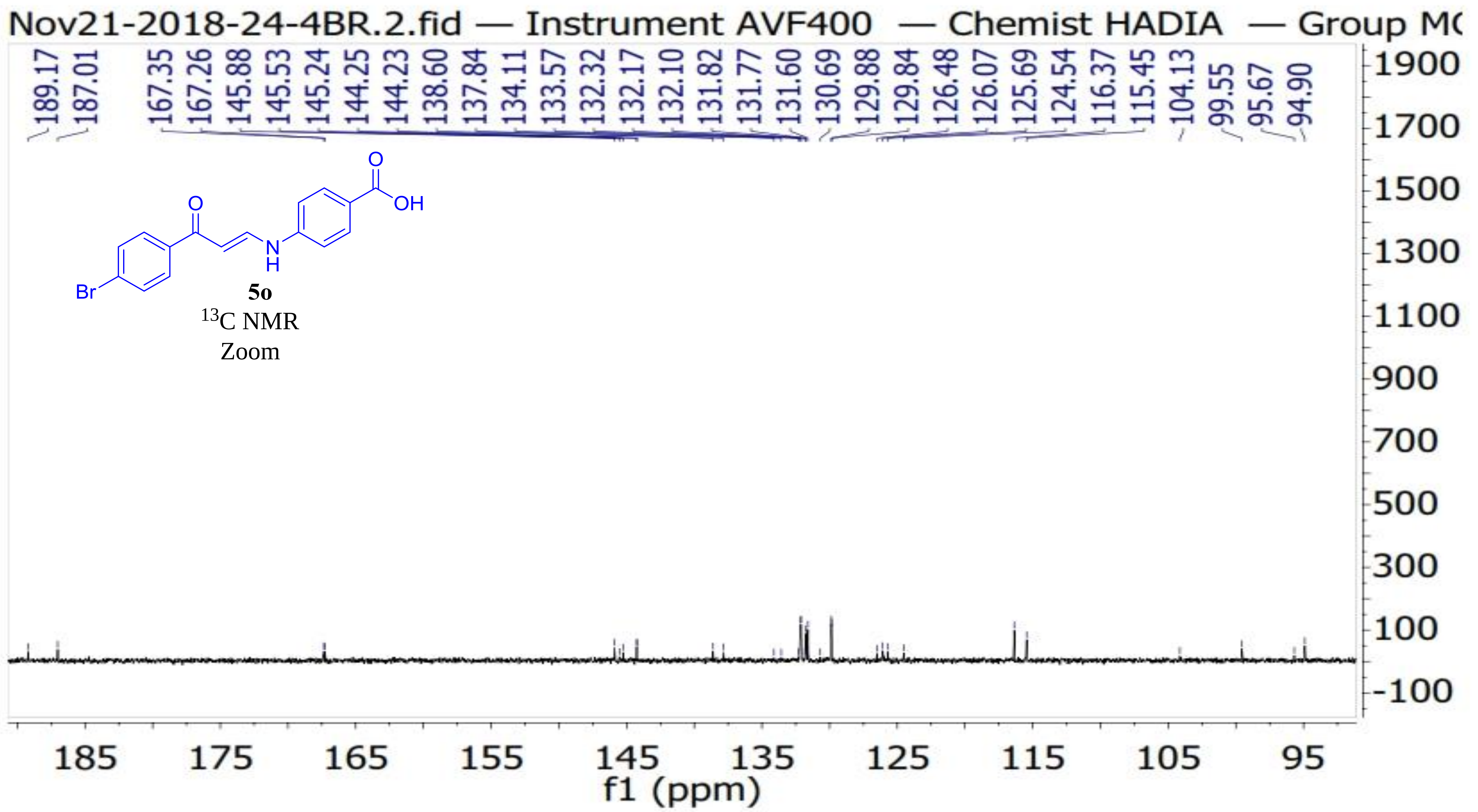
Nov21-2018-24-4BR.2.fid — Instrument AVF400 — Chemist HADIA — Group MC
189.17
187.01
167.35
167.26
145.88
145.53
145.24
144.25
144.23
138.60
137.84
134.11
133.57
132.32
132.17
132.10
131.82
131.77
131.60
130.69
129.88
129.84
126.48
126.07
125.69
124.54
116.37
115.45
104.13
99.55
95.67
94.90
O
OH
O
N
H
Br
5o
13C NMR
Zoom
f1 (ppm)

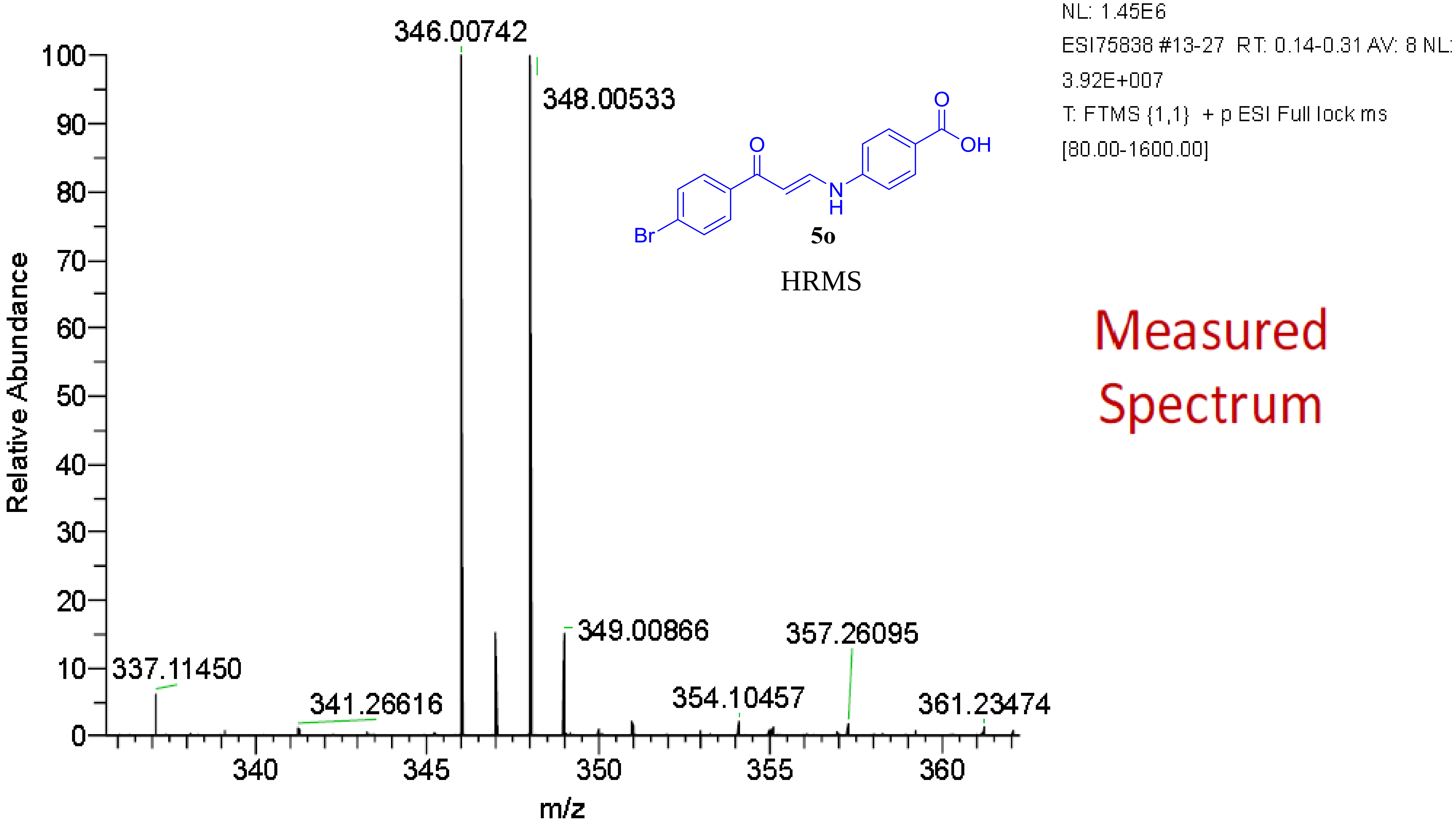
NL: 1.45E6
ESI75838 #13-27 RT: 0.14-0.31 AV: 8 NL: 3.92E+007
T: FTMS {1,1} + p ESI Full lock ms [80.00-1600.00]
346.00742
348.00533
349.00866
357.26095
337.11450
341.26616
354.10457
361.23474
Relative Abundance
m/z
Br
O
N
H
OH
5o
HRMS
Measured Spectrum

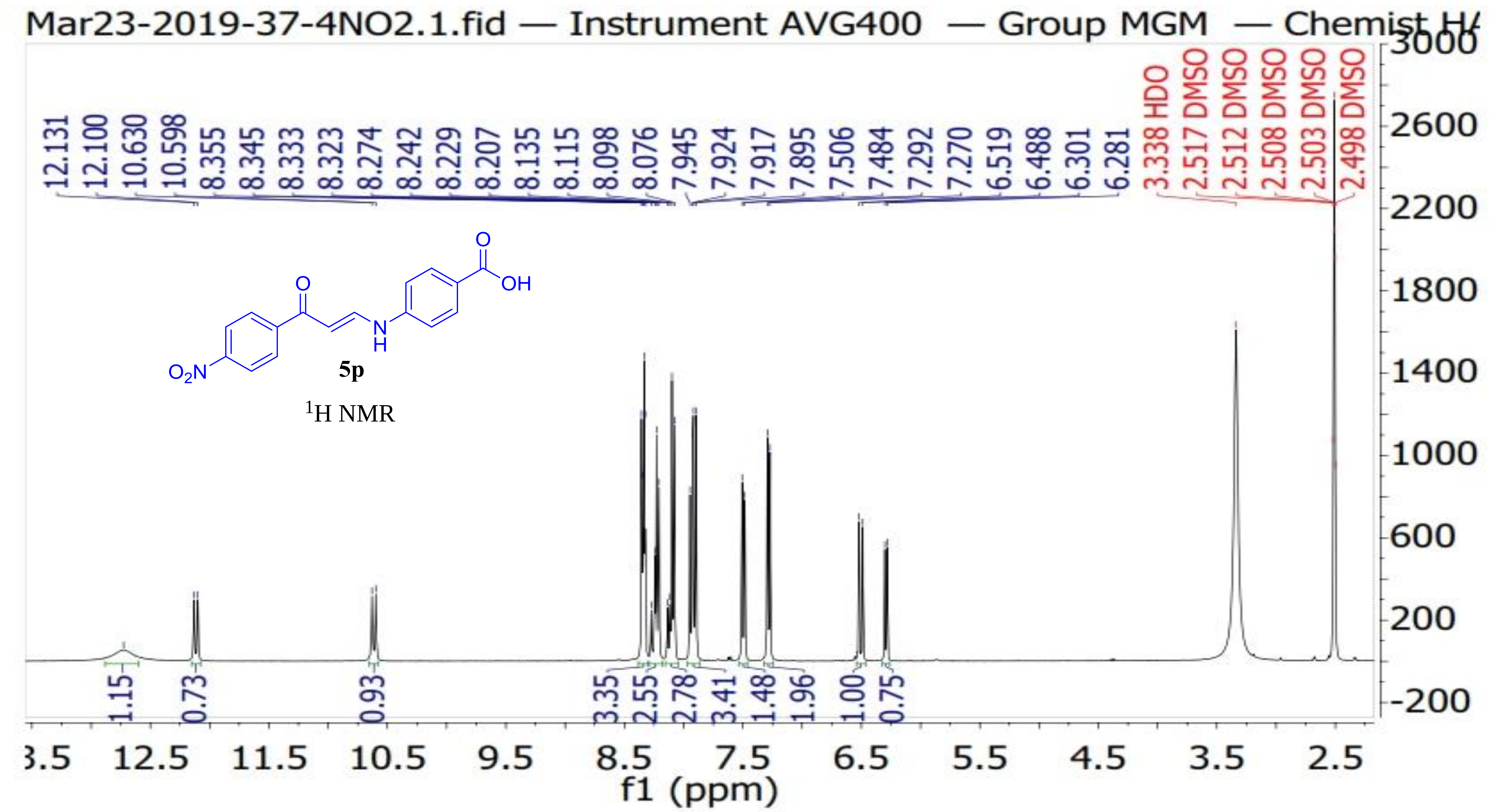
Mar23-2019-37-4NO2.1.fid — Instrument AVG400 — Group MGM — Chemist H/
12.131
12.100
10.630
10.598
8.355
8.345
8.333
8.323
8.274
8.242
8.229
8.207
8.135
8.115
8.098
8.076
7.945
7.924
7.917
7.895
7.506
7.484
7.292
7.270
6.519
6.488
6.301
6.281
3.338 HDO
2.517 DMSO
2.512 DMSO
2.508 DMSO
2.503 DMSO
2.498 DMSO
O
OH
O
N
H
O_2N
5p
^{1}H NMR
1.15
0.73
0.93
3.35
2.55
2.78
3.41
1.48
1.96
1.00
0.75
f1 (ppm)

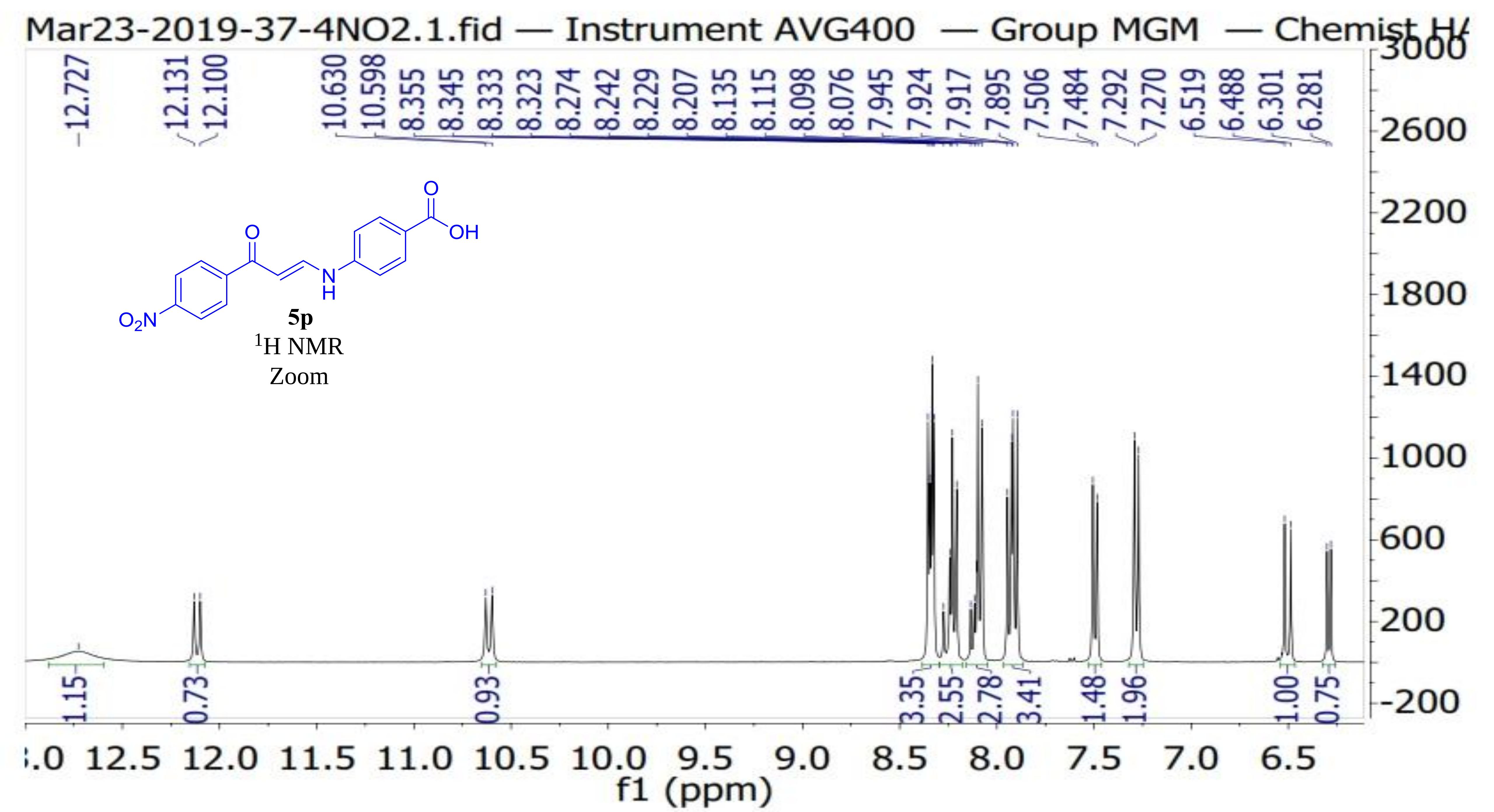
Mar23-2019-37-4NO2.1.fid — Instrument AVG400 — Group MGM — Chemist H/
12.727
12.131
12.100
10.630
10.598
8.355
8.345
8.333
8.323
8.274
8.242
8.229
8.207
8.135
8.115
8.098
8.076
7.945
7.924
7.917
7.895
7.506
7.484
7.292
7.270
6.519
6.488
6.301
6.281
O
OH
O
N
H
O2N
5p
1H NMR
Zoom
1.15
0.73
0.93
3.35
2.55
2.78
3.41
1.48
1.96
1.00
0.75
3000
2600
2200
1800
1400
1000
600
200
-200
12.5
12.0
11.5
11.0
10.5
10.0
9.5
9.0
8.5
8.0
7.5
7.0
6.5
f1 (ppm)

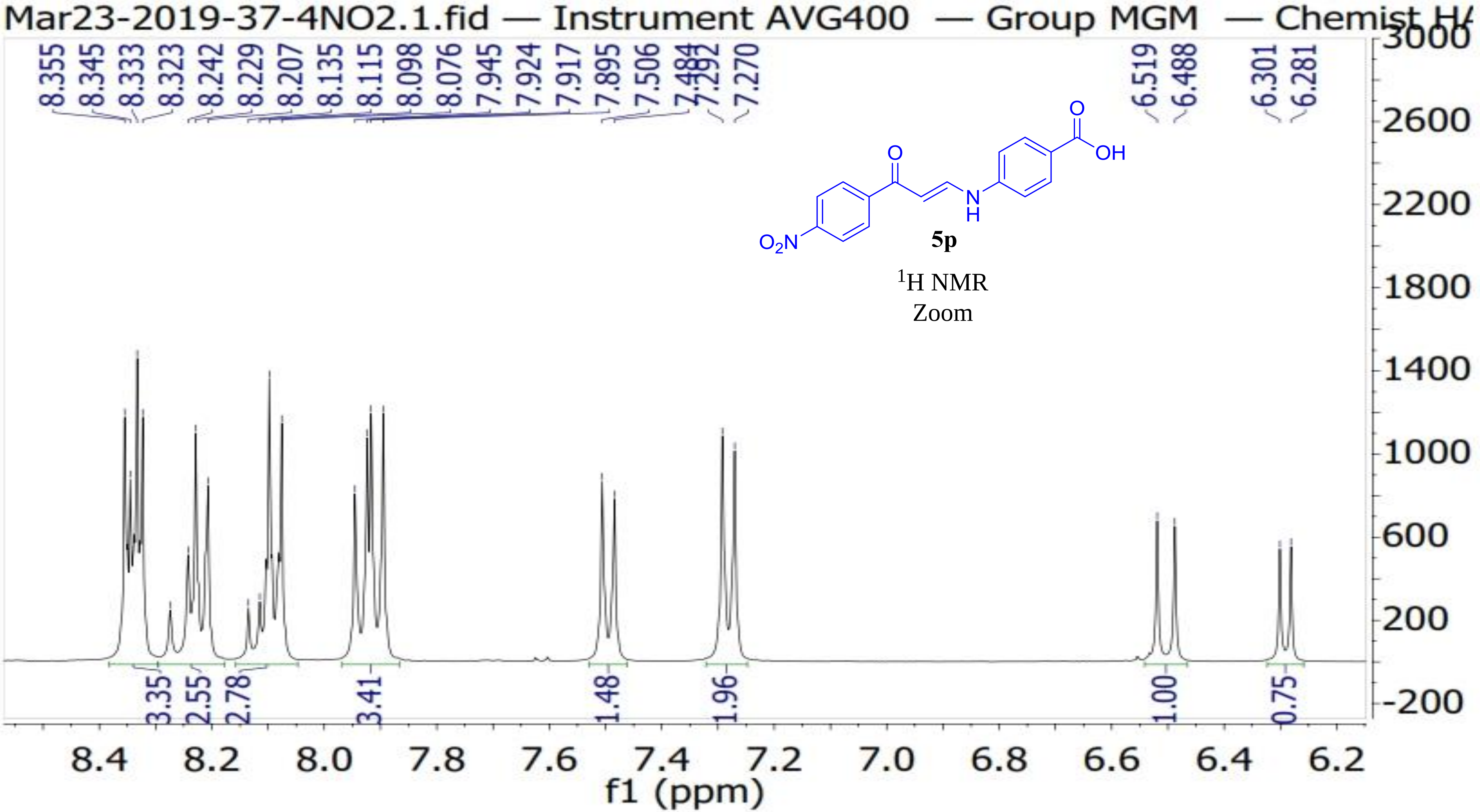

Mar23-2019-37-4NO2.1.fid — Instrument AVG400 — Group MGM — Chemist H/
8.355 8.345 8.333 8.323 8.242 8.229 8.207 8.135 8.115 8.098 8.076 7.945 7.924 7.917 7.895 7.506 7.484 7.292 7.270 6.519 6.488 6.301 6.281
O
OH
O
N
H
O2N
5p
1H NMR
Zoom
3.35 2.55 2.78 3.41 1.48 1.96 1.00 0.75
3000 2600 2200 1800 1400 1000 600 200 -200
8.4 8.2 8.0 7.8 7.6 7.4 7.2 7.0 6.8 6.6 6.4 6.2
f1 (ppm)

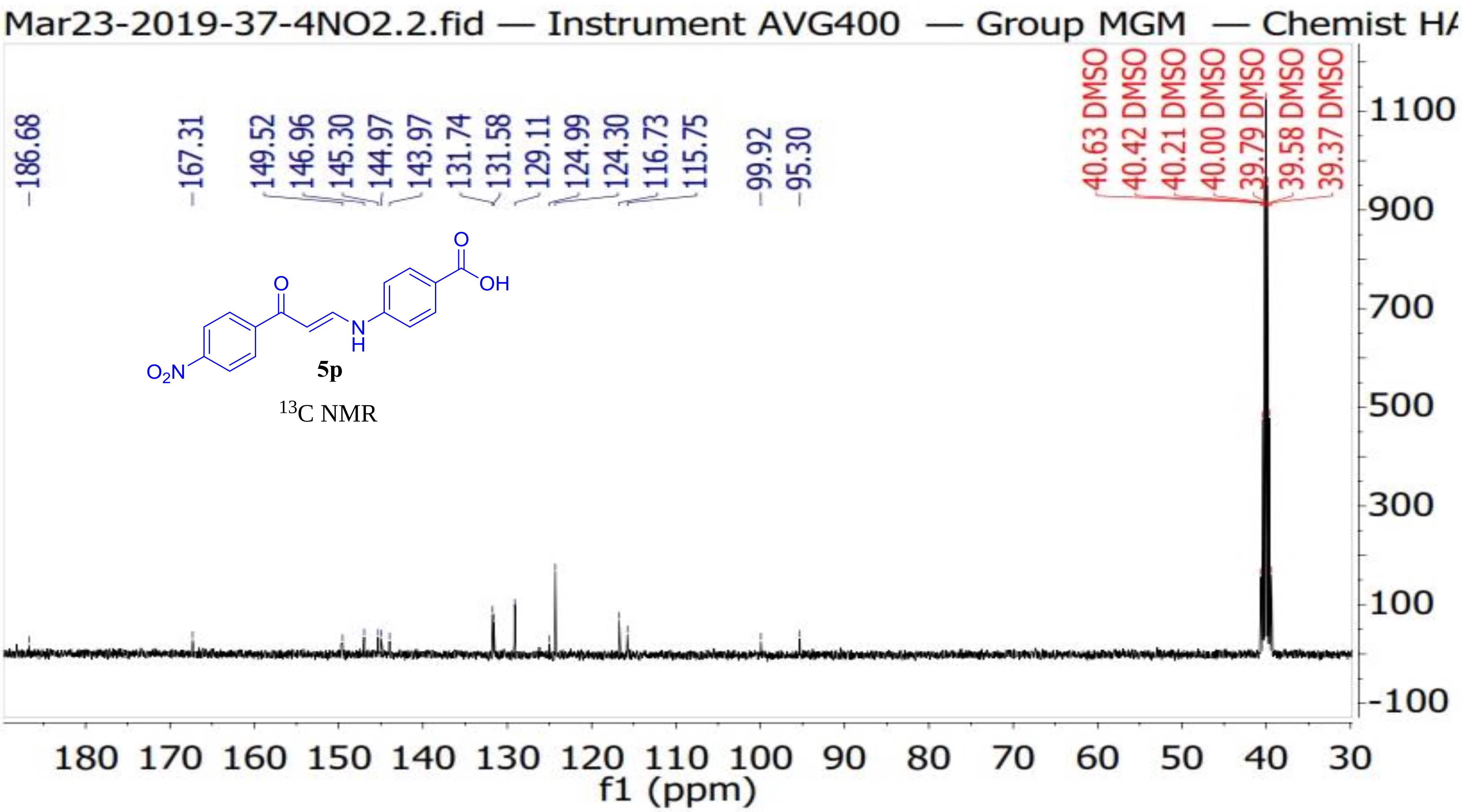

Mar23-2019-37-4NO2.2.fid — Instrument AVG400 — Group MGM — Chemist HA
186.68
167.31
149.52
146.96
145.30
144.97
143.97
131.74
131.58
129.11
124.99
124.30
116.73
115.75
99.92
95.30
40.63 DMSO
40.42 DMSO
40.21 DMSO
40.00 DMSO
39.79 DMSO
39.58 DMSO
39.37 DMSO
O
OH
O
N
H
O_2N
5p
^{13}C NMR
f1 (ppm)

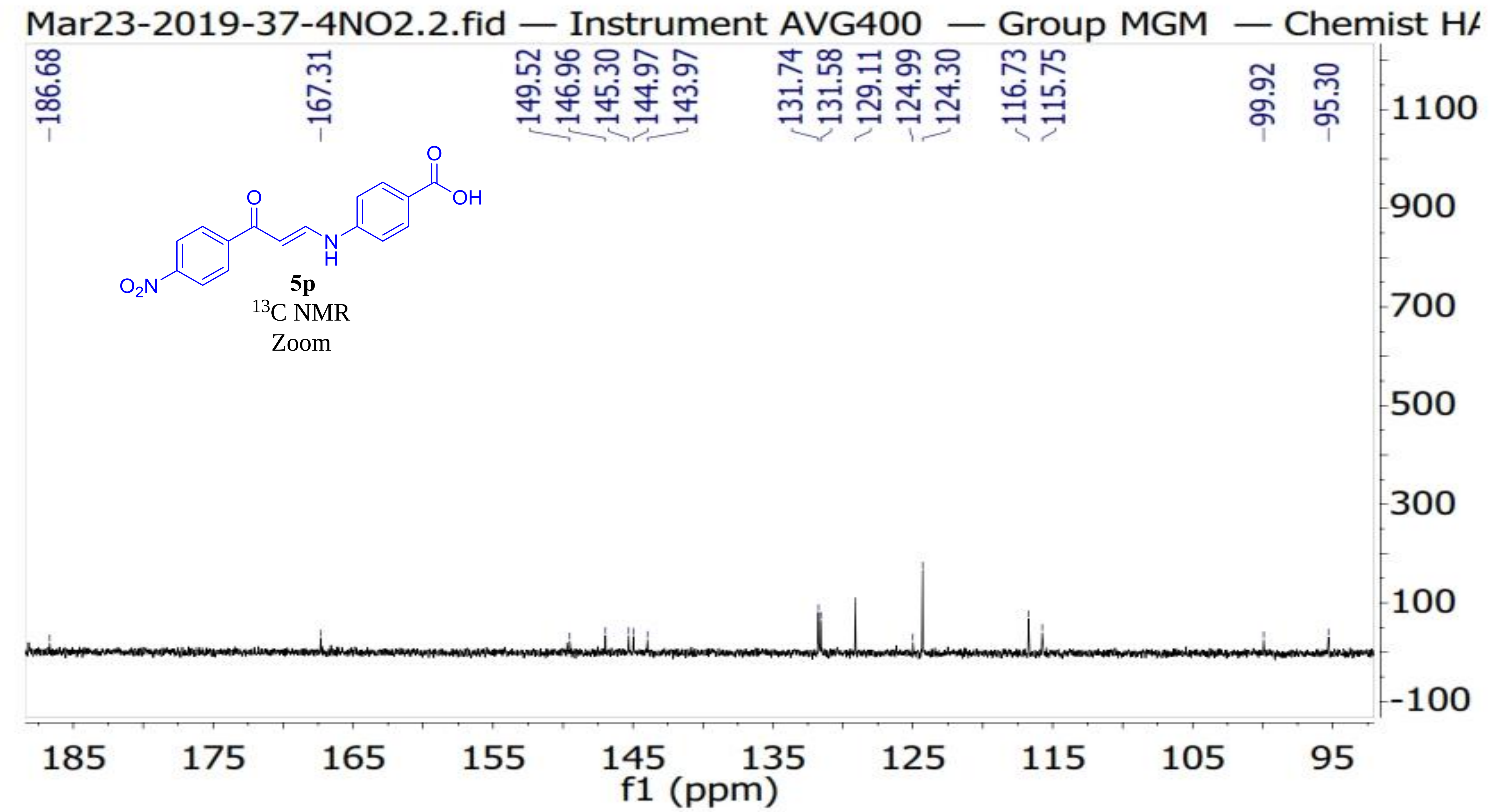

Mar23-2019-37-4NO2.2.fid — Instrument AVG400 — Group MGM — Chemist HA
186.68
167.31
149.52
146.96
145.30
144.97
143.97
131.74
131.58
129.11
124.99
124.30
116.73
115.75
99.92
95.30
O
OH
O
N
H
O2N
5p
13C NMR
Zoom
1100
900
700
500
300
100
-100
185
175
165
155
145
135
125
115
105
95
f1 (ppm)

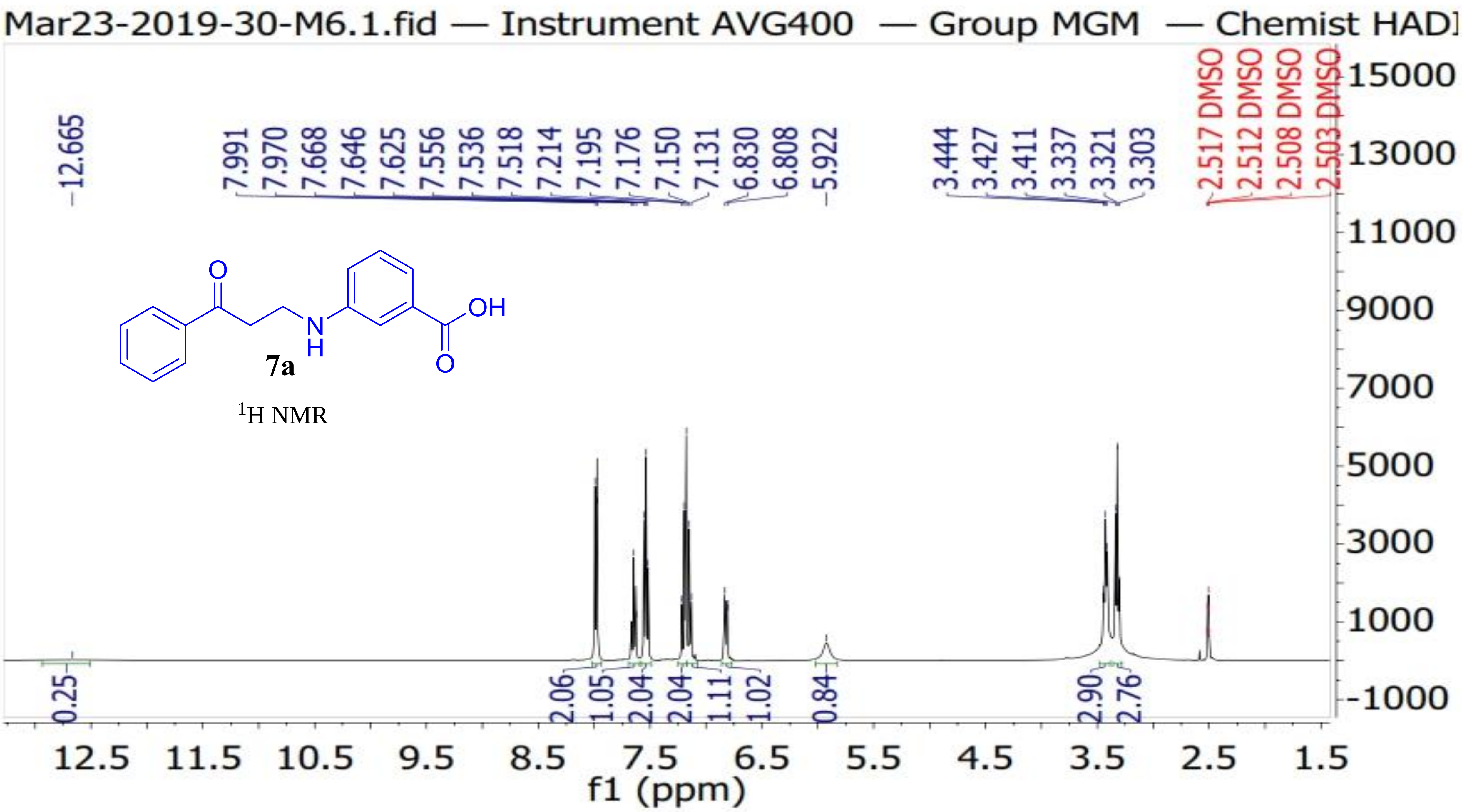
Mar23-2019-30-M6.1.fid — Instrument AVG400 — Group MGM — Chemist HAD]
12.665
7.991 7.970 7.668 7.646 7.625 7.556 7.536 7.518 7.214 7.195 7.176 7.150 7.131 6.830 6.808 5.922
3.444 3.427 3.411 3.337 3.321 3.303
2.517 DMSO 2.512 DMSO 2.508 DMSO 2.503 DMSO
O
OH
N
H
O
7a
1H NMR
0.25 2.06 1.05 2.04 2.04 1.11 1.02 0.84 2.90 2.76
15000 13000 11000 9000 7000 5000 3000 1000 -1000
12.5 11.5 10.5 9.5 8.5 7.5 6.5 5.5 4.5 3.5 2.5 1.5
f1 (ppm)

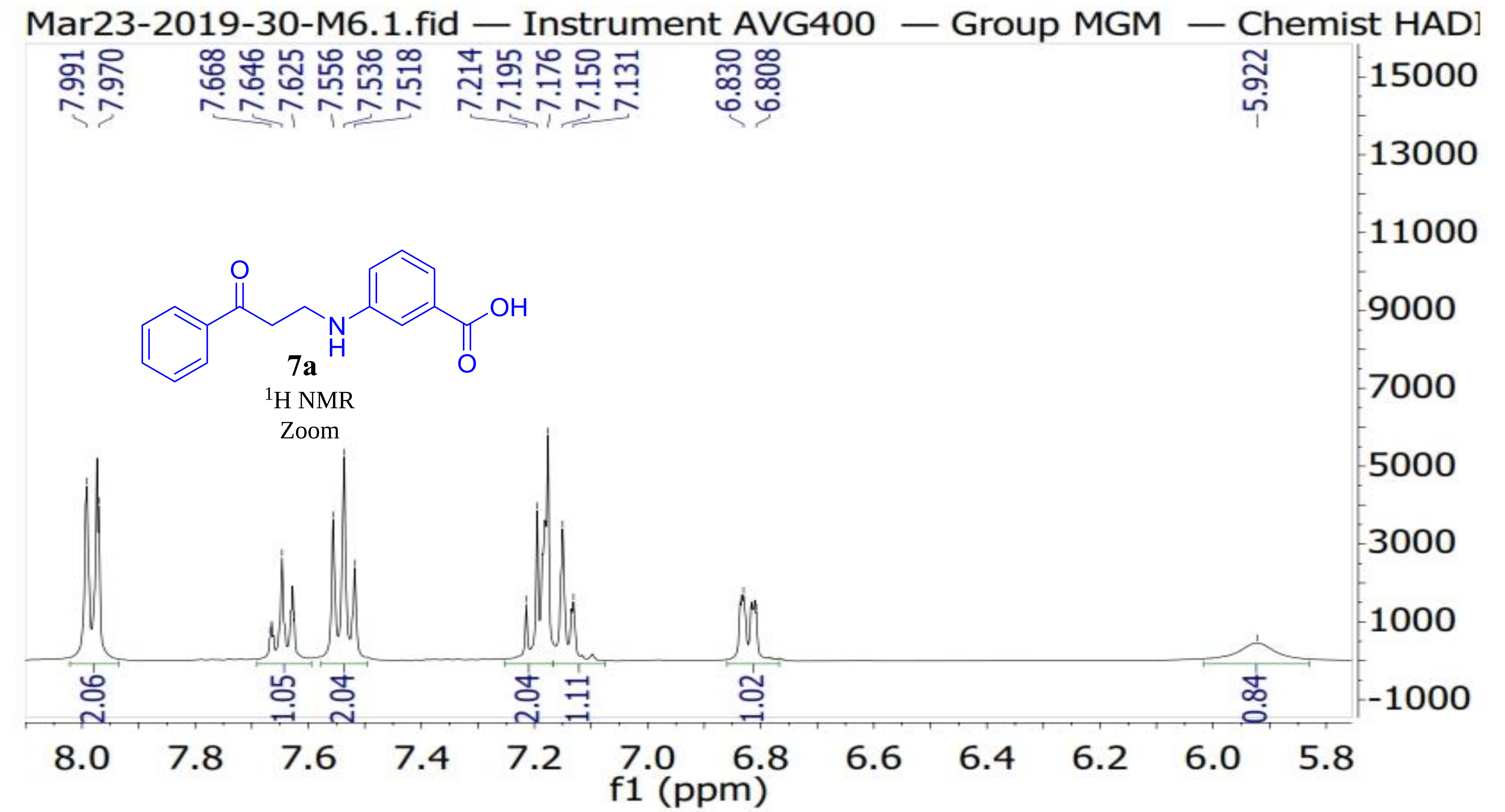
Mar23-2019-30-M6.1.fid — Instrument AVG400 — Group MGM — Chemist HADI
7.991
7.970
7.668
7.646
7.625
7.556
7.536
7.518
7.214
7.195
7.176
7.150
7.131
6.830
6.808
5.922
OH
O
O
N
H
7a
1H NMR
Zoom
2.06
1.05
2.04
2.04
1.11
1.02
0.84
15000
13000
11000
9000
7000
5000
3000
1000
-1000
8.0
7.8
7.6
7.4
7.2
7.0
6.8
6.6
6.4
6.2
6.0
5.8
f1 (ppm)

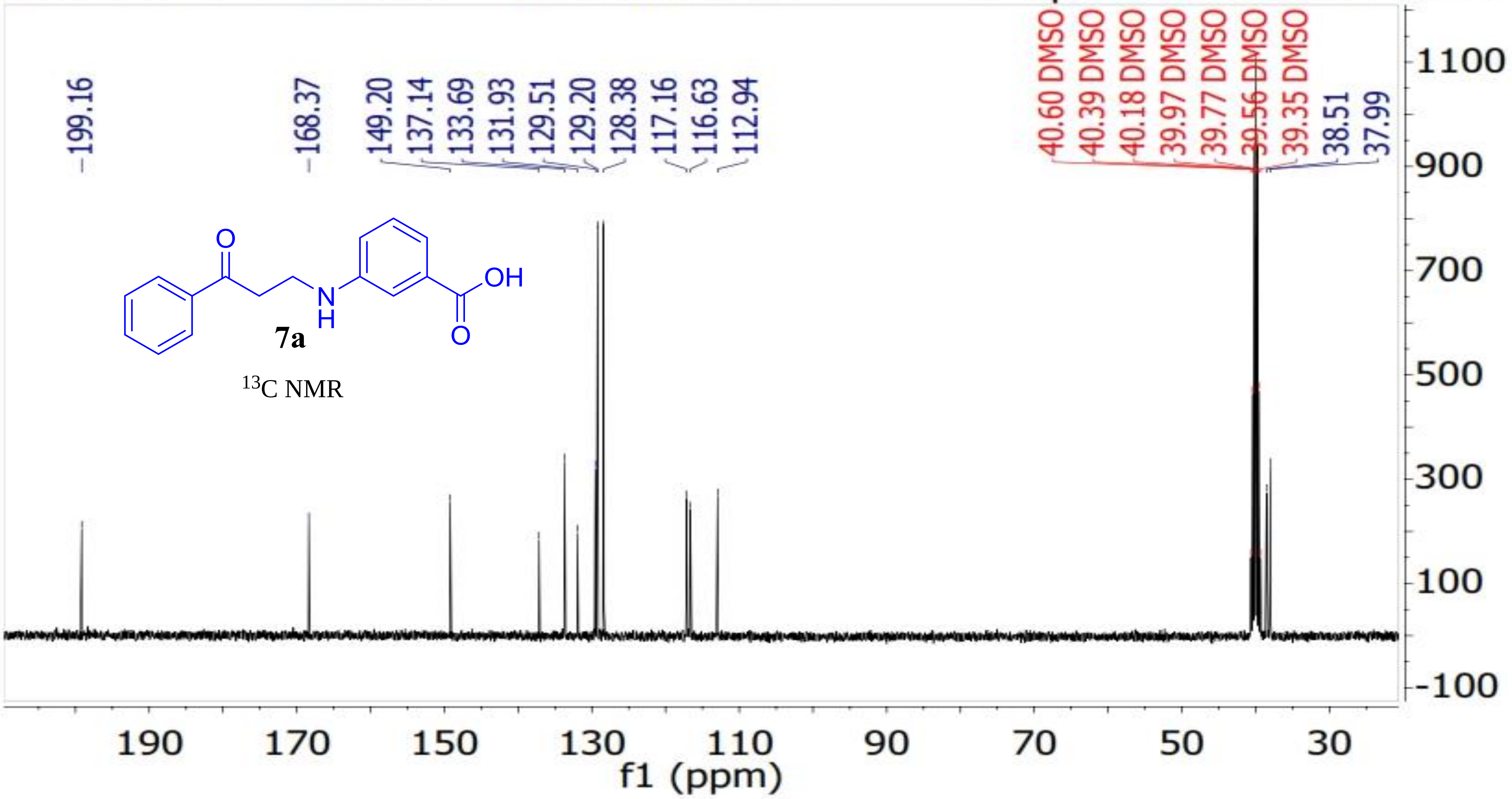
Mar23-2019-30-M6.2.fid — Instrument AVG400 — Group MGM — Chemist HAD]
199.16
168.37
149.20
137.14
133.69
131.93
129.51
129.20
128.38
117.16
116.63
112.94
40.60 DMSO
40.39 DMSO
40.18 DMSO
39.97 DMSO
39.77 DMSO
39.56 DMSO
39.35 DMSO
38.51
37.99
O
OH
N
H
O
7a
13C NMR
1100
900
700
500
300
100
-100
190
170
150
130
110
90
70
50
30
f1 (ppm)

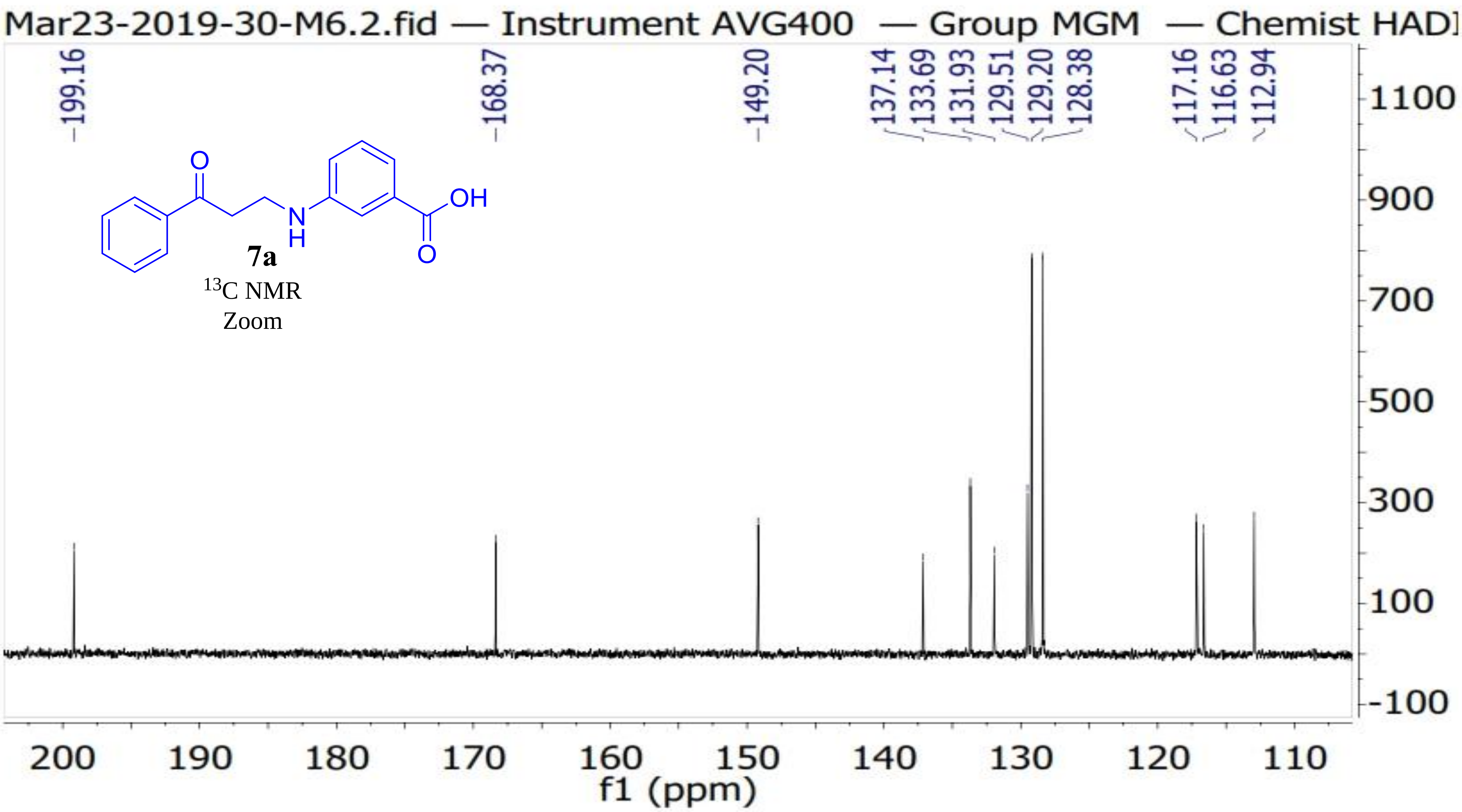

Mar23-2019-30-M6.2.fid — Instrument AVG400 — Group MGM — Chemist HADI
199.16
168.37
149.20
137.14
133.69
131.93
129.51
129.20
128.38
117.16
116.63
112.94
OH
O
O
N
H
7a
13C NMR
Zoom
1100
900
700
500
300
100
-100
200
190
180
170
160
150
140
130
120
110
f1 (ppm)

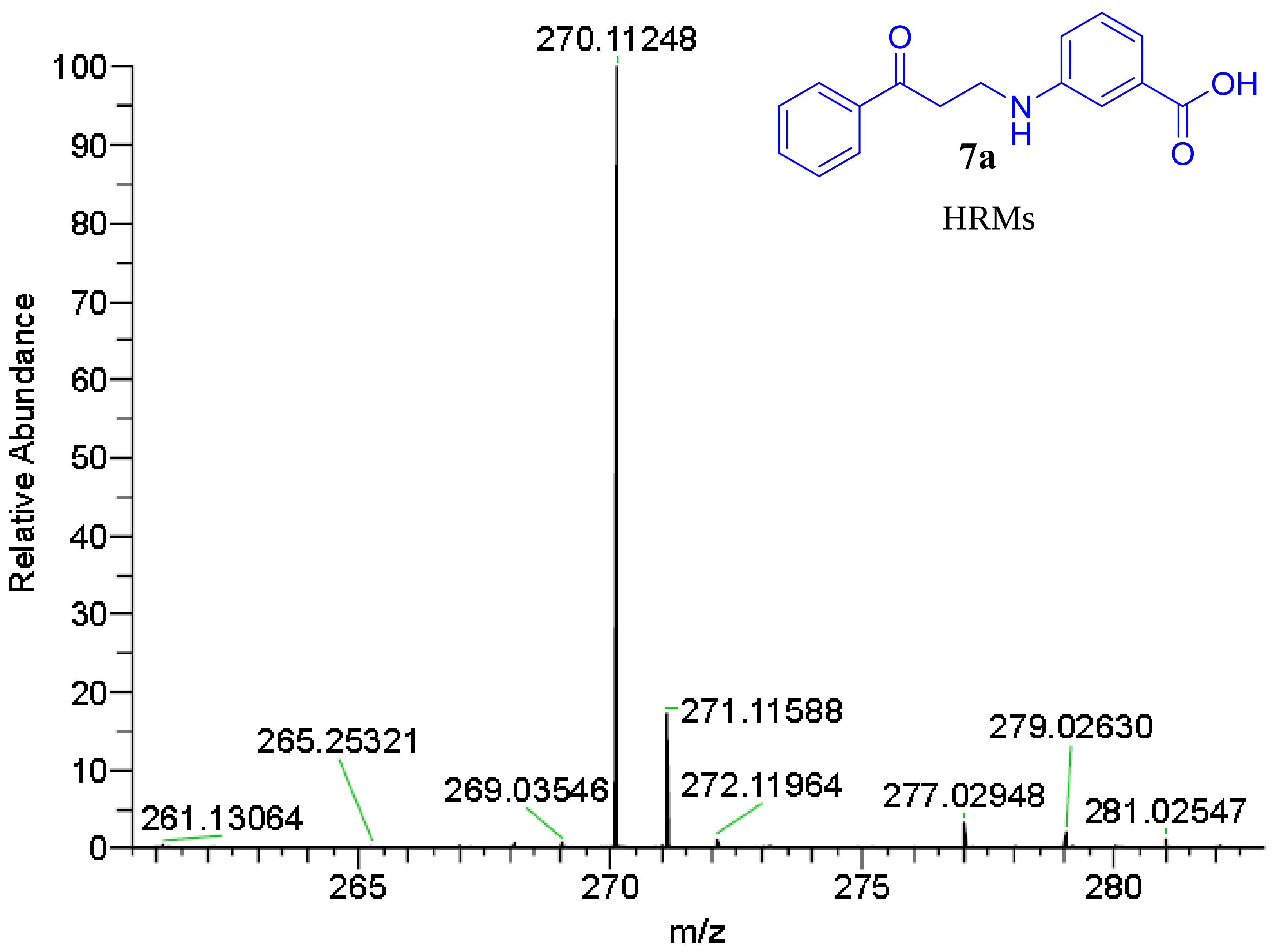

NL: 1.66E7
ESI75822 #13-27 RT: 0.14-0.3 AV: 8 NL: 3.04E+007
T: FTMS {1,1} + p ESI Full lock ms [80.00-1600.00]

Measured Spectrum

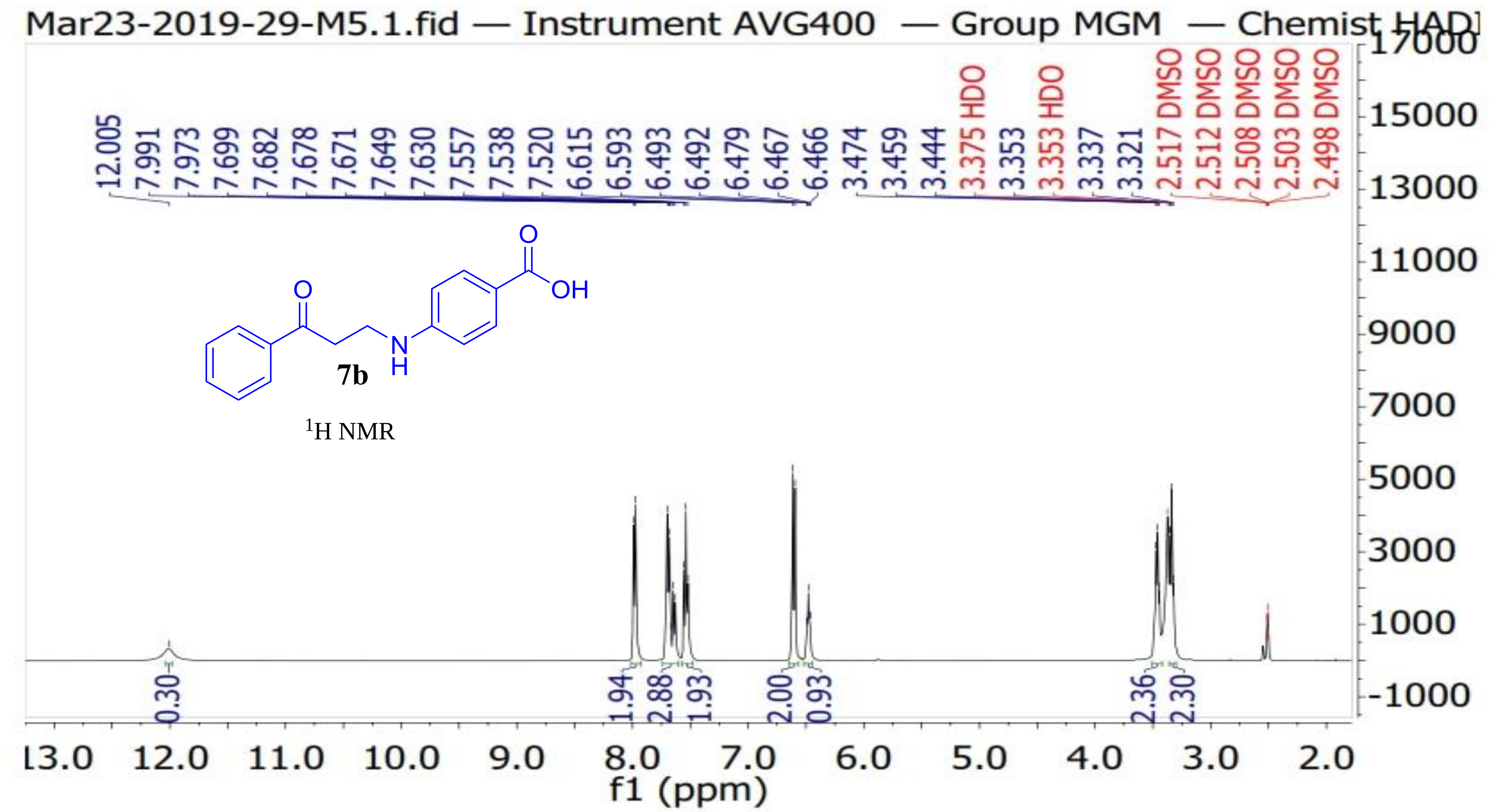

Mar23-2019-29-M5.1.fid — Instrument AVG400 — Group MGM — Chemist HADI
12.005
7.991
7.973
7.699
7.682
7.678
7.671
7.649
7.630
7.557
7.538
7.520
6.615
6.593
6.493
6.492
6.479
6.467
6.466
3.474
3.459
3.444
3.375 HDO
3.353
3.353 HDO
3.337
3.321
2.517 DMSO
2.512 DMSO
2.508 DMSO
2.503 DMSO
2.498 DMSO
7b
¹H NMR
0.30
1.94
2.88
1.93
2.00
0.93
2.36
2.30
f1 (ppm)

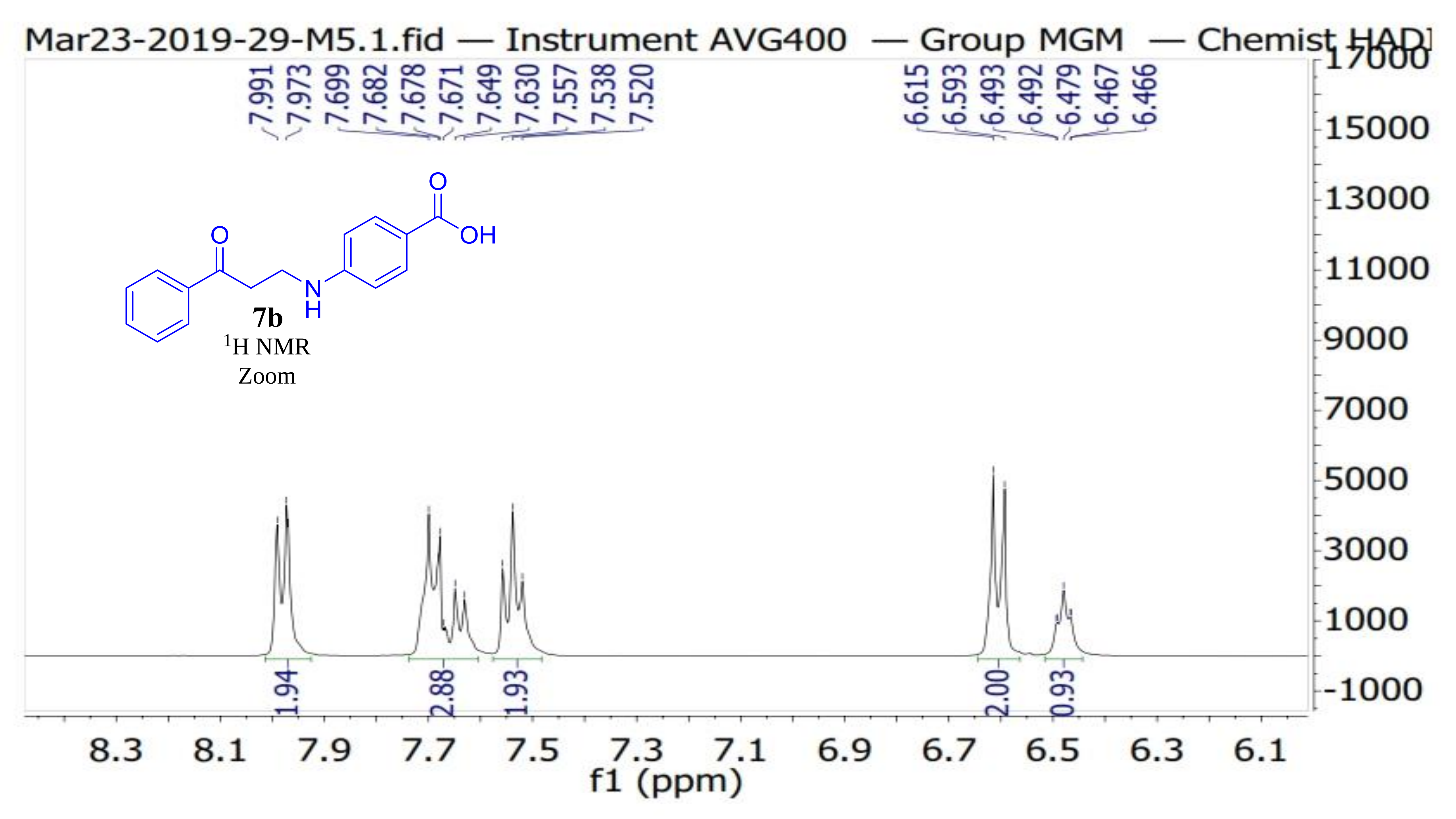

Mar23-2019-29-M5.1.fid — Instrument AVG400 — Group MGM — Chemist HAD
7.991 7.973 7.699 7.682 7.678 7.671 7.649 7.630 7.557 7.538 7.520
6.615 6.593 6.493 6.492 6.479 6.467 6.466
O
OH
O
N
H
7b
1H NMR
Zoom
1.94 2.88 1.93 2.00 0.93
f1 (ppm)

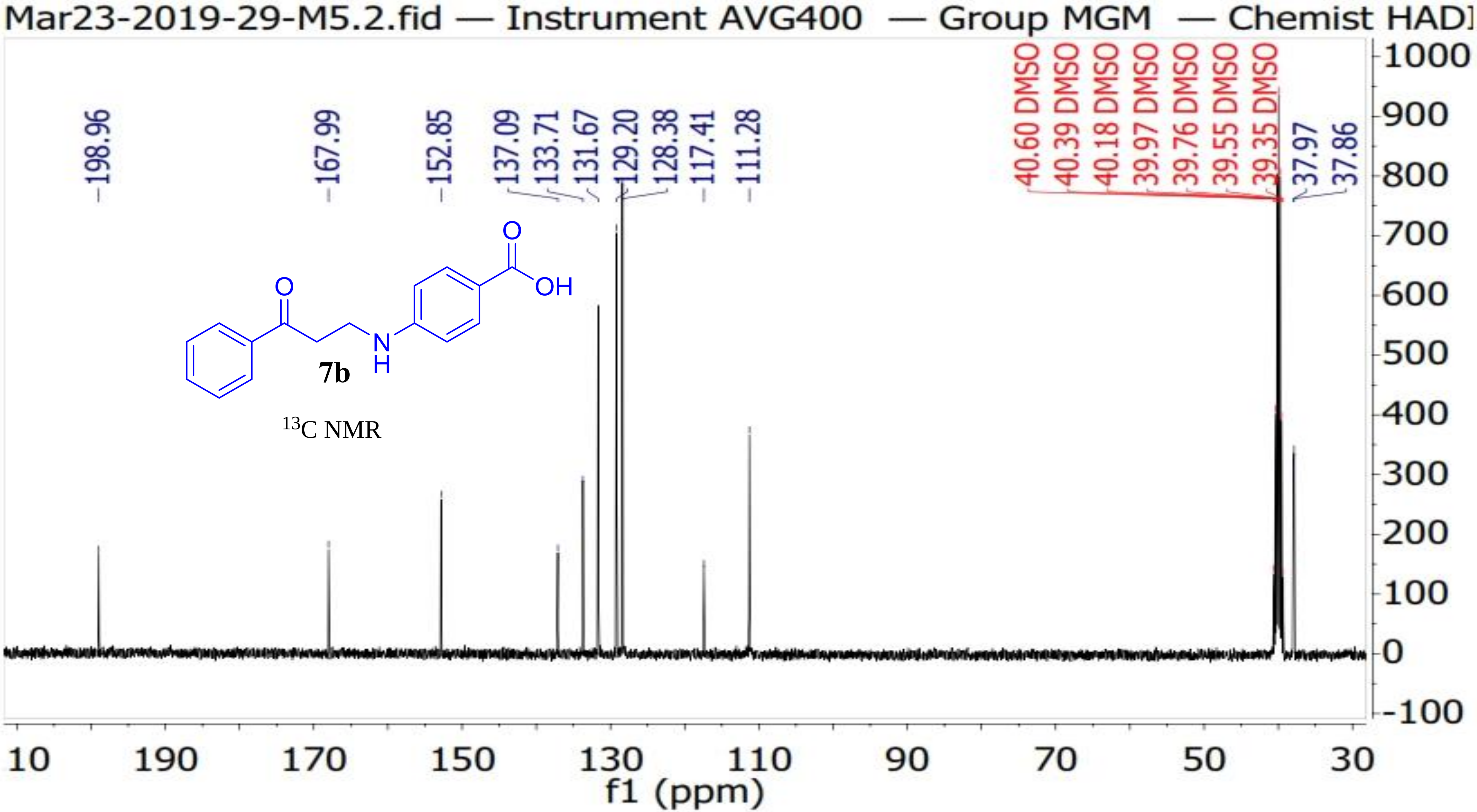
Mar23-2019-29-M5.2.fid — Instrument AVG400 — Group MGM — Chemist HADI
198.96
167.99
152.85
137.09
133.71
131.67
129.20
128.38
117.41
111.28
40.60 DMSO
40.39 DMSO
40.18 DMSO
39.97 DMSO
39.76 DMSO
39.55 DMSO
39.35 DMSO
37.97
37.86
O
OH
O
N
H
7b
13C NMR
1000
900
800
700
600
500
400
300
200
100
0
-100
10
190
170
150
130
110
90
70
50
30
f1 (ppm)

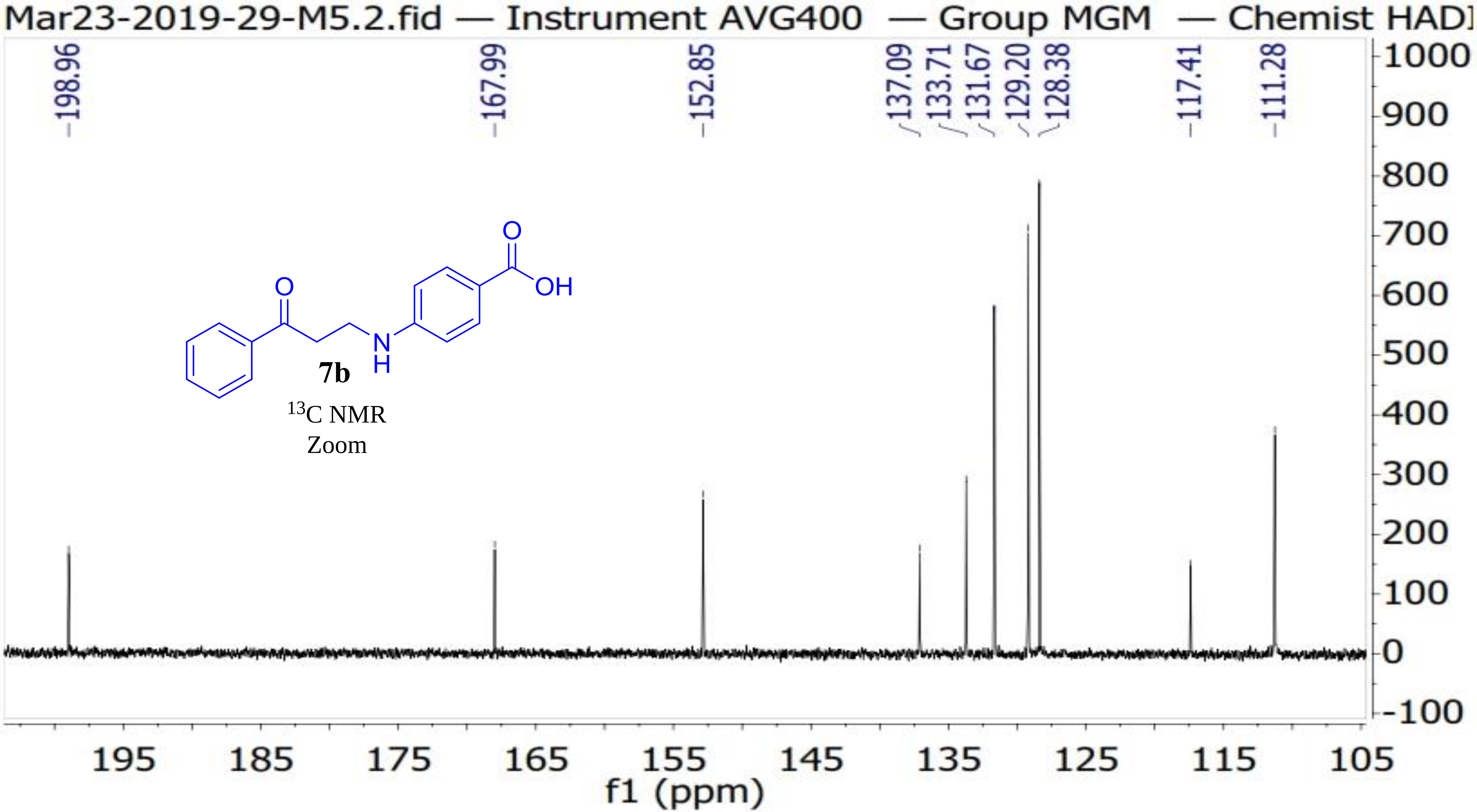

Mar23-2019-29-M5.2.fid — Instrument AVG400 — Group MGM — Chemist HADI
198.96
167.99
152.85
137.09
133.71
131.67
129.20
128.38
117.41
111.28
O
OH
N
H
7b
13C NMR
Zoom
1000
900
800
700
600
500
400
300
200
100
0
-100
195
185
175
165
155
145
135
125
115
105
f1 (ppm)

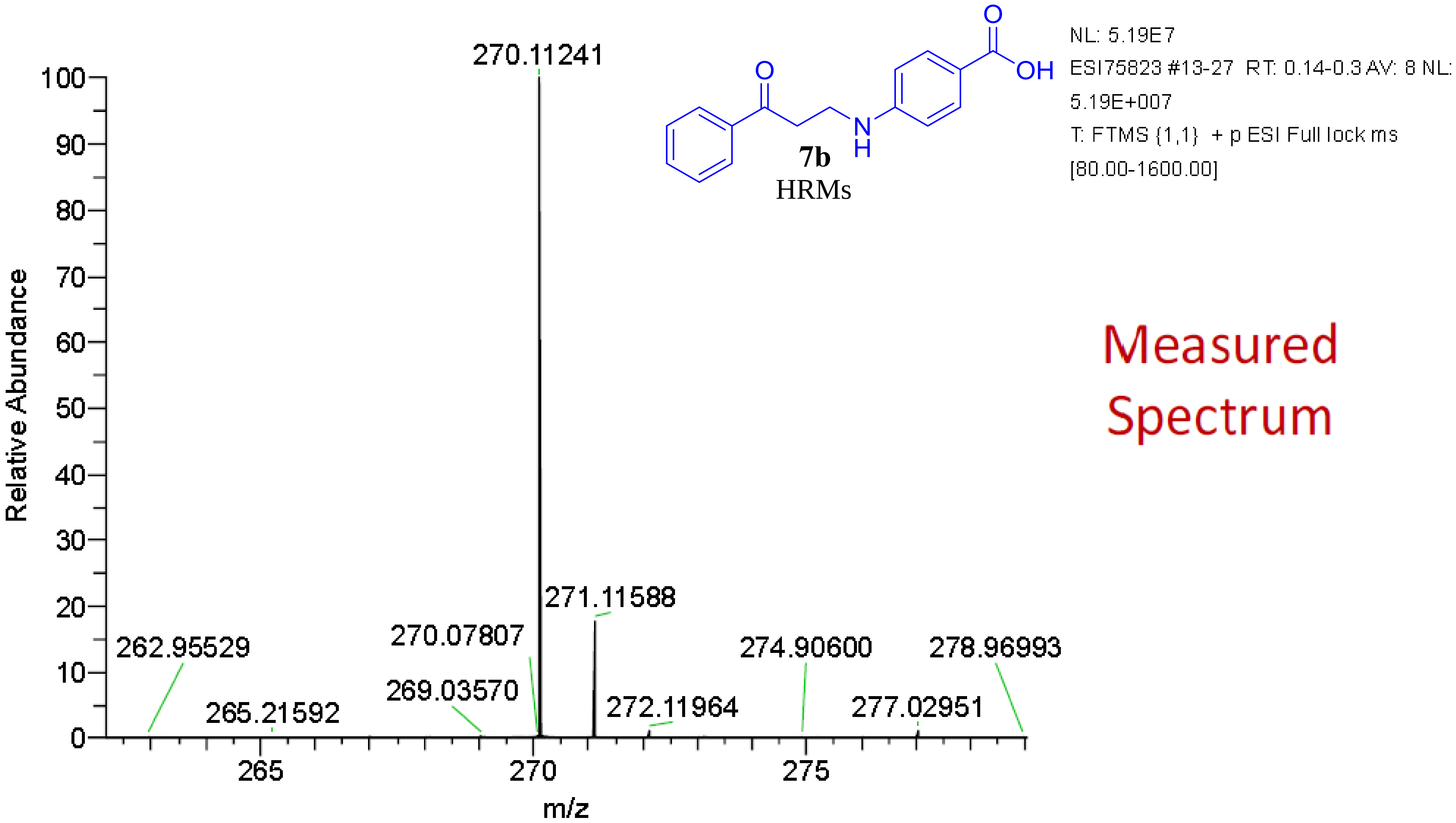
NL: 5.19E7
ESI75823 #13-27 RT: 0.14-0.3 AV: 8 NL: 5.19E+007
T: FTMS {1,1} + p ESI Full lock ms [80.00-1600.00]
O
OH
O
N
H
7b
HRMs
Measured Spectrum
270.11241
271.11588
270.07807
262.95529
269.03570
265.21592
272.11964
274.90600
278.96993
277.02951
Relative Abundance
m/z
0
10
20
30
40
50
60
70
80
90
100
265
270
275

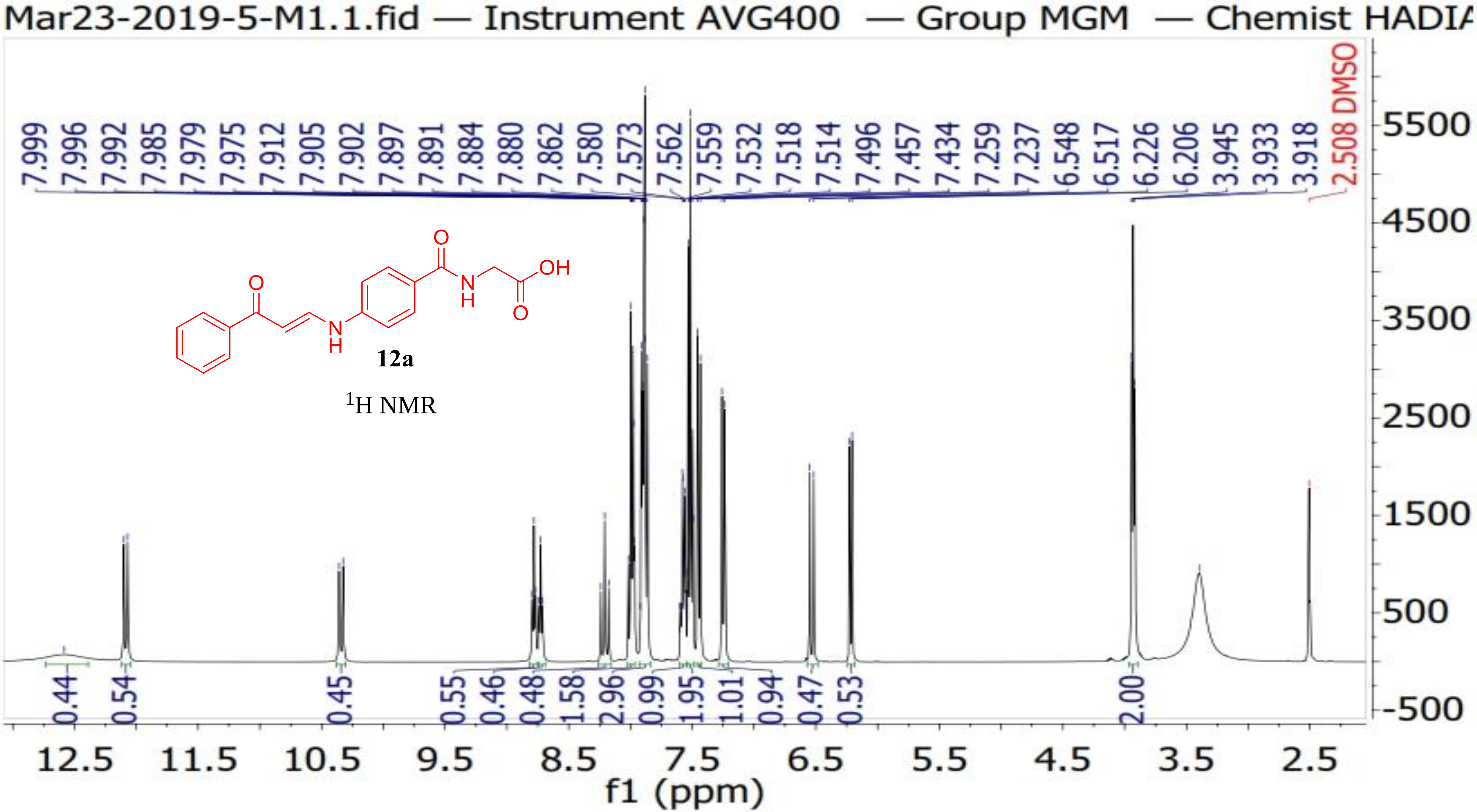
Mar23-2019-5-M1.1.fid — Instrument AVG400 — Group MGM — Chemist HADIA
7.999 7.996 7.992 7.985 7.979 7.975 7.912 7.905 7.902 7.897 7.891 7.884 7.880 7.862 7.580 7.573 7.562 7.559 7.532 7.518 7.514 7.496 7.457 7.434 7.259 7.237 6.548 6.517 6.226 6.206 3.945 3.933 3.918
2.508 DMSO
12a
1H NMR
0.44 0.54 0.45 0.55 0.46 0.48 1.58 2.96 0.99 1.95 1.01 0.94 0.47 0.53 2.00
5500 4500 3500 2500 1500 500 -500
12.5 11.5 10.5 9.5 8.5 7.5 6.5 5.5 4.5 3.5 2.5
f1 (ppm)

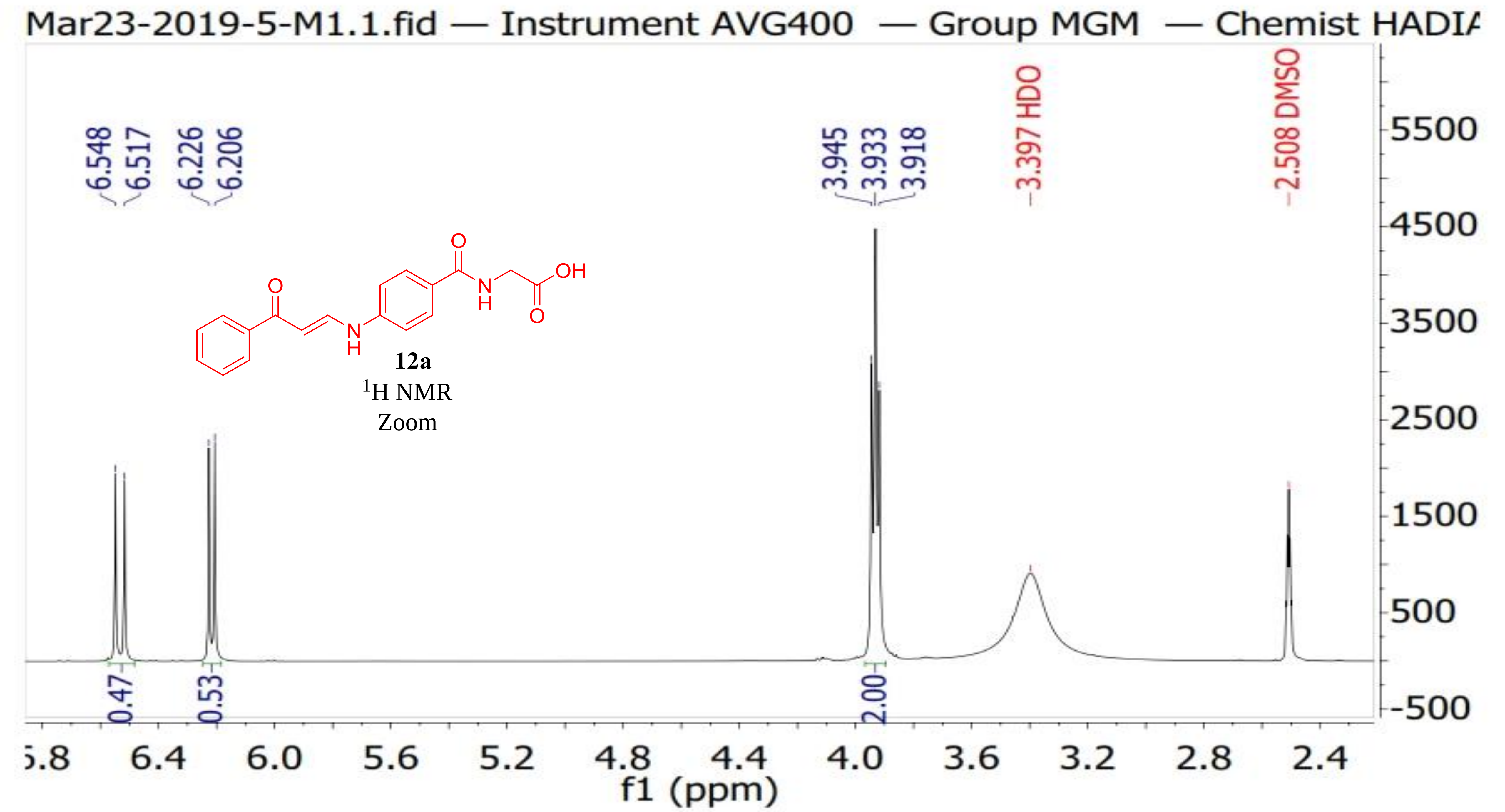

Mar23-2019-5-M1.1.fid — Instrument AVG400 — Group MGM — Chemist HADIA
6.548
6.517
6.226
6.206
3.945
3.933
3.918
3.397 HDO
2.508 DMSO
O
O
N
H
O
N
H
OH
O
12a
1H NMR
Zoom
0.47
0.53
2.00
5500
4500
3500
2500
1500
500
-500
6.4
6.0
5.6
5.2
4.8
4.4
4.0
3.6
3.2
2.8
2.4
f1 (ppm)

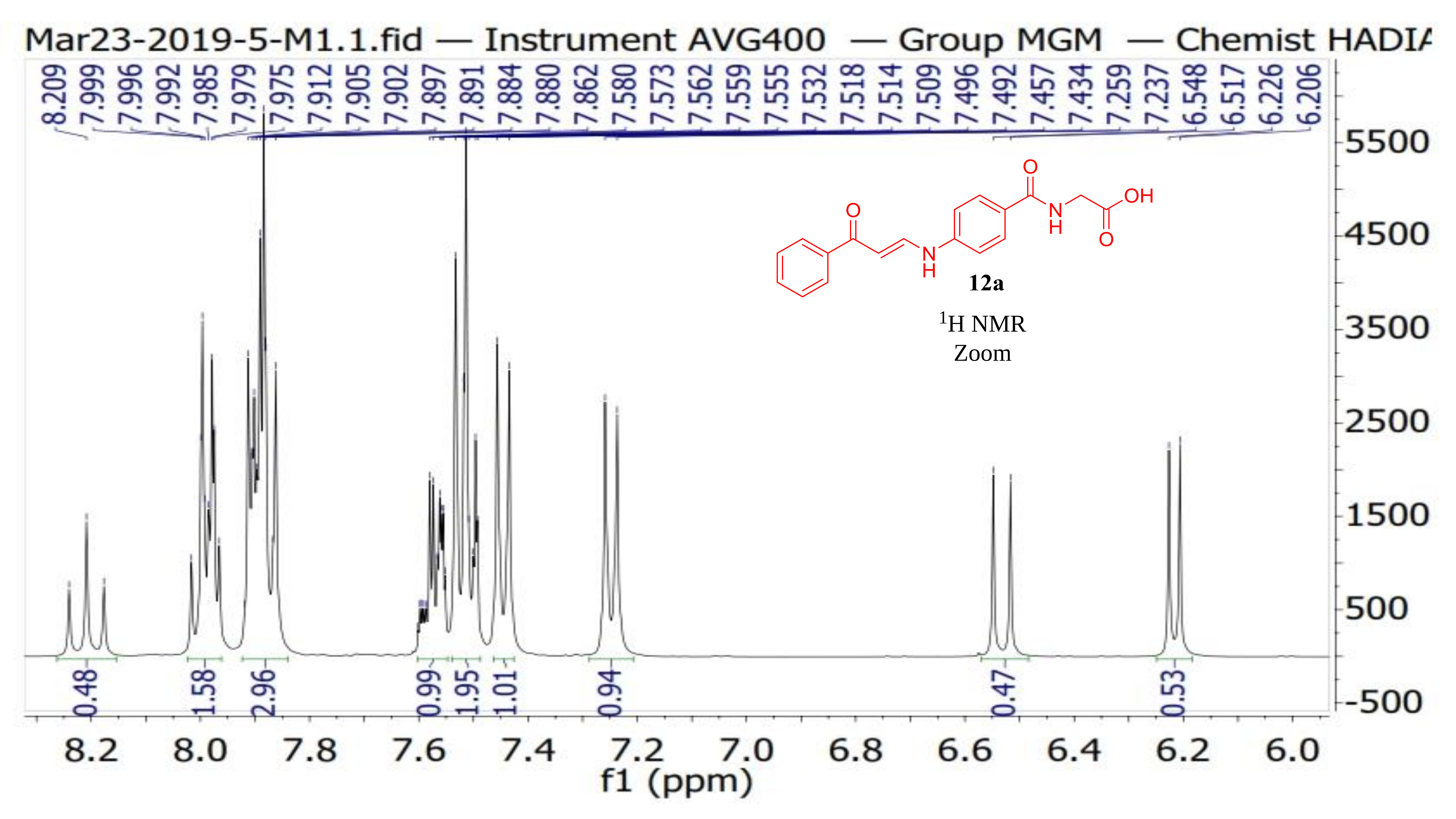
Mar23-2019-5-M1.1.fid — Instrument AVG400 — Group MGM — Chemist HADIA
8.209 7.999 7.996 7.992 7.985 7.979 7.975 7.912 7.905 7.902 7.897 7.891 7.884 7.880 7.862 7.580 7.573 7.562 7.559 7.555 7.532 7.518 7.514 7.509 7.496 7.492 7.457 7.434 7.259 7.237 6.548 6.517 6.226 6.206
O
OH
N
H
O
O
N
H
12a
^{1}H NMR
Zoom
0.48 1.58 2.96 0.99 1.95 1.01 0.94 0.47 0.53
5500
4500
3500
2500
1500
500
-500
8.2 8.0 7.8 7.6 7.4 7.2 7.0 6.8 6.6 6.4 6.2 6.0
f1 (ppm)

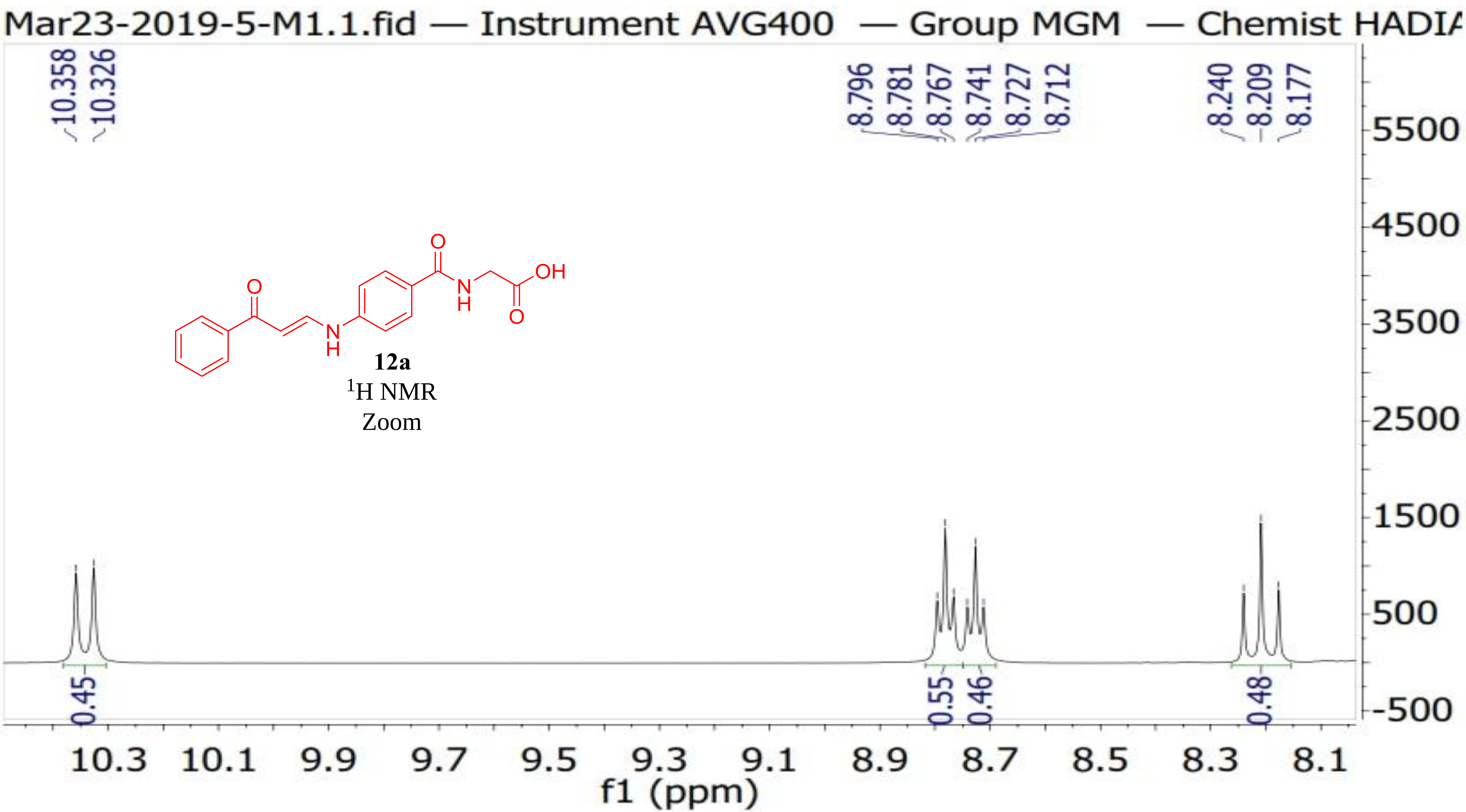
Mar23-2019-5-M1.1.fid — Instrument AVG400 — Group MGM — Chemist HADIA
10.358
10.326
8.796
8.781
8.767
8.741
8.727
8.712
8.240
8.209
8.177
12a
1H NMR
Zoom
O
O
N
H
N
H
OH
O
0.45
0.55
0.46
0.48
5500
4500
3500
2500
1500
500
-500
10.3
10.1
9.9
9.7
9.5
9.3
9.1
8.9
8.7
8.5
8.3
8.1
f1 (ppm)

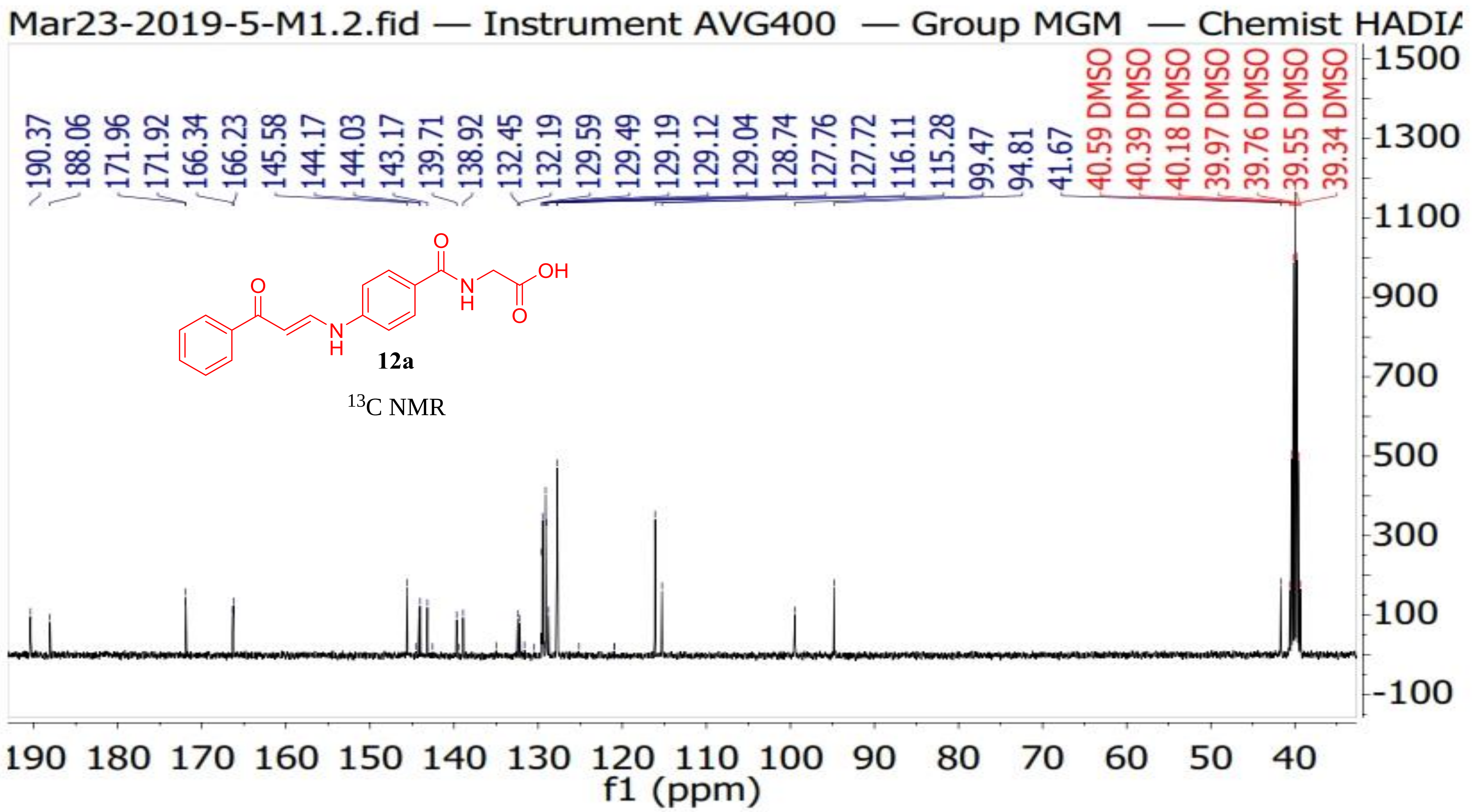
Mar23-2019-5-M1.2.fid — Instrument AVG400 — Group MGM — Chemist HADIA
190.37
188.06
171.96
171.92
166.34
166.23
145.58
144.17
144.03
143.17
139.71
138.92
132.45
132.19
129.59
129.49
129.19
129.12
129.04
128.74
127.76
127.72
116.11
115.28
99.47
94.81
41.67
40.59 DMSO
40.39 DMSO
40.18 DMSO
39.97 DMSO
39.76 DMSO
39.55 DMSO
39.34 DMSO
O
OH
N
H
O
O
N
H
12a
13C NMR
f1 (ppm)

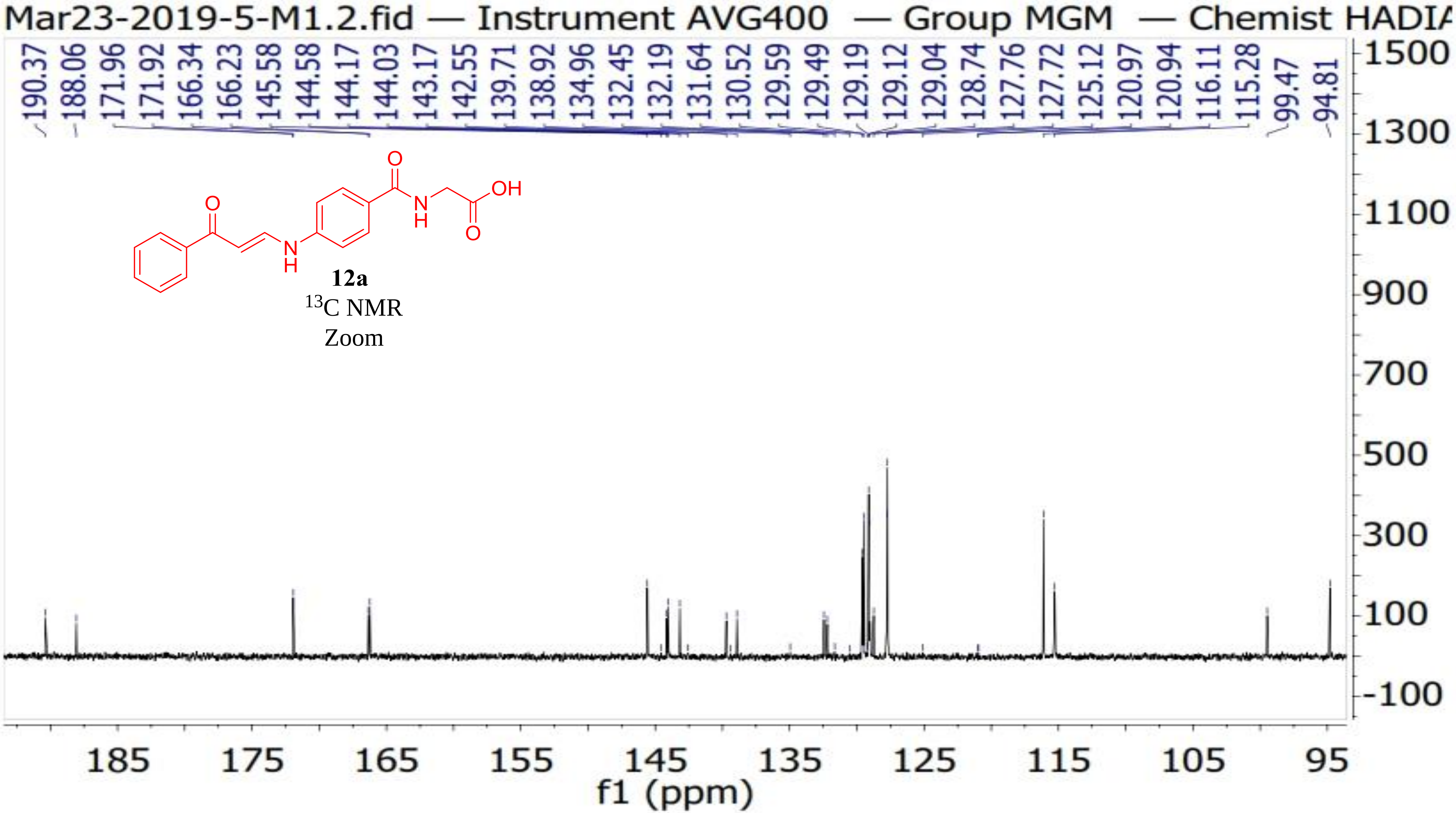
Mar23-2019-5-M1.2.fid — Instrument AVG400 — Group MGM — Chemist HADIA
190.37
188.06
171.96
171.92
166.34
166.23
145.58
144.58
144.17
144.03
143.17
142.55
139.71
138.92
134.96
132.45
132.19
131.64
130.52
129.59
129.49
129.19
129.12
129.04
128.74
127.76
127.72
125.12
120.97
120.94
116.11
115.28
99.47
94.81
O
OH
N
H
O
O
N
H
12a
13C NMR
Zoom
1500
1300
1100
900
700
500
300
100
-100
185
175
165
155
145
135
125
115
105
95
f1 (ppm)

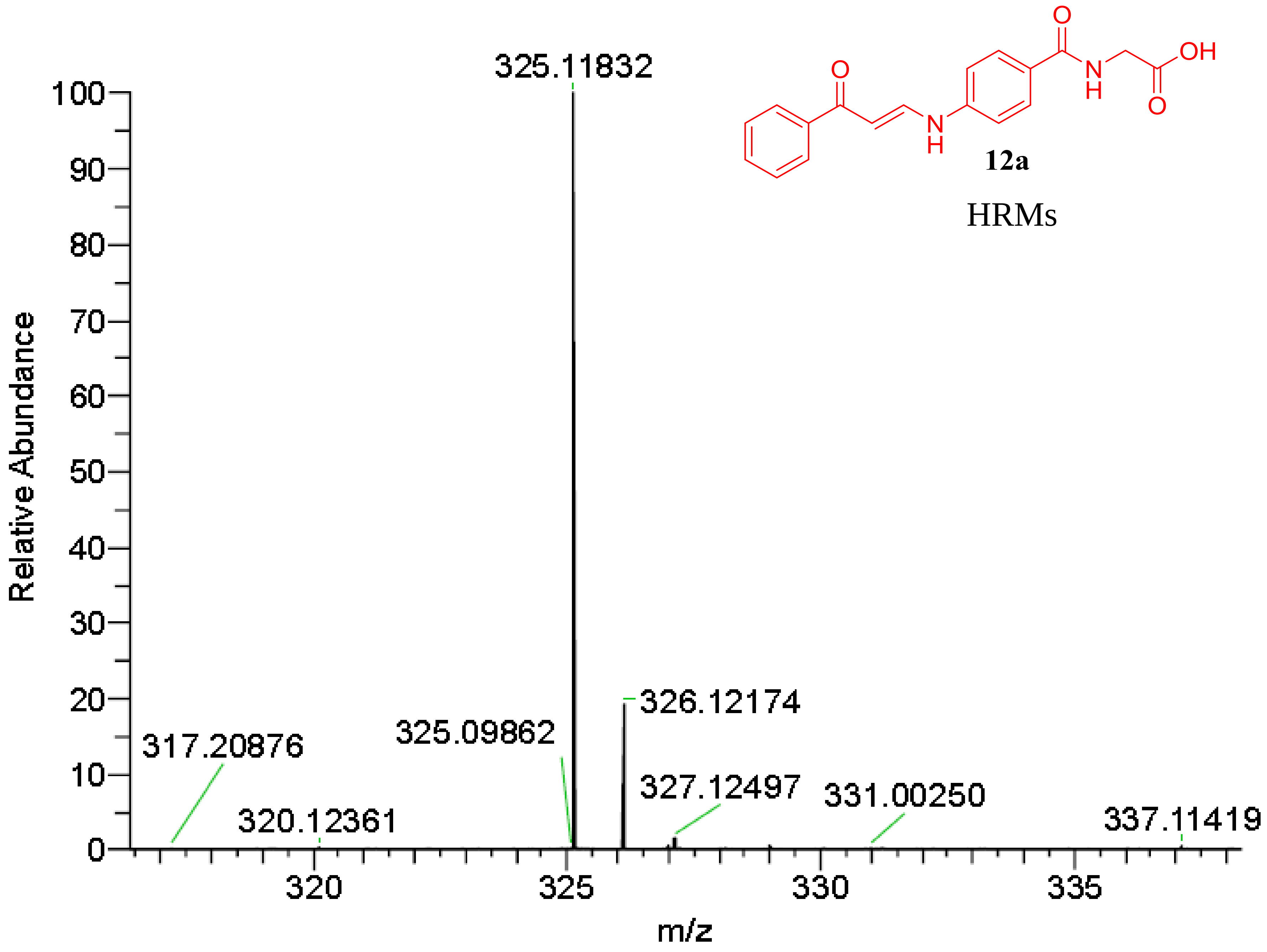

12a

HRMs

NL: 1.36E7
ESI75818 #13-27 RT: 0.15-0.31 AV: 8 NL: 2.54E+007
T: FTMS {1,1} + p ESI Full lock ms [80.00-1600.00]

Measured Spectrum

Mar23-2019-6-M2.1.fid — Instrument AVG400 — Group MGM — Chemist HADIA

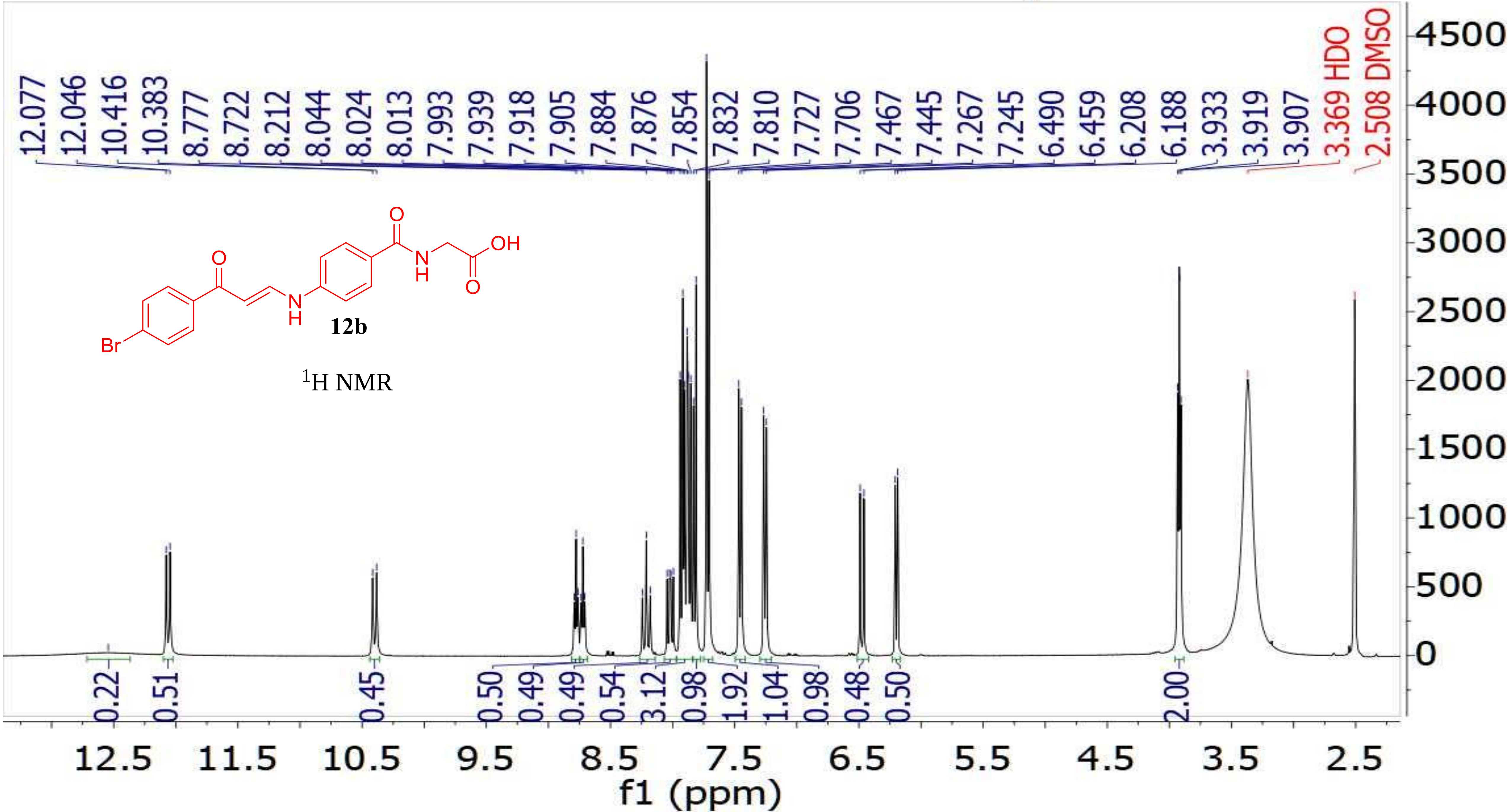

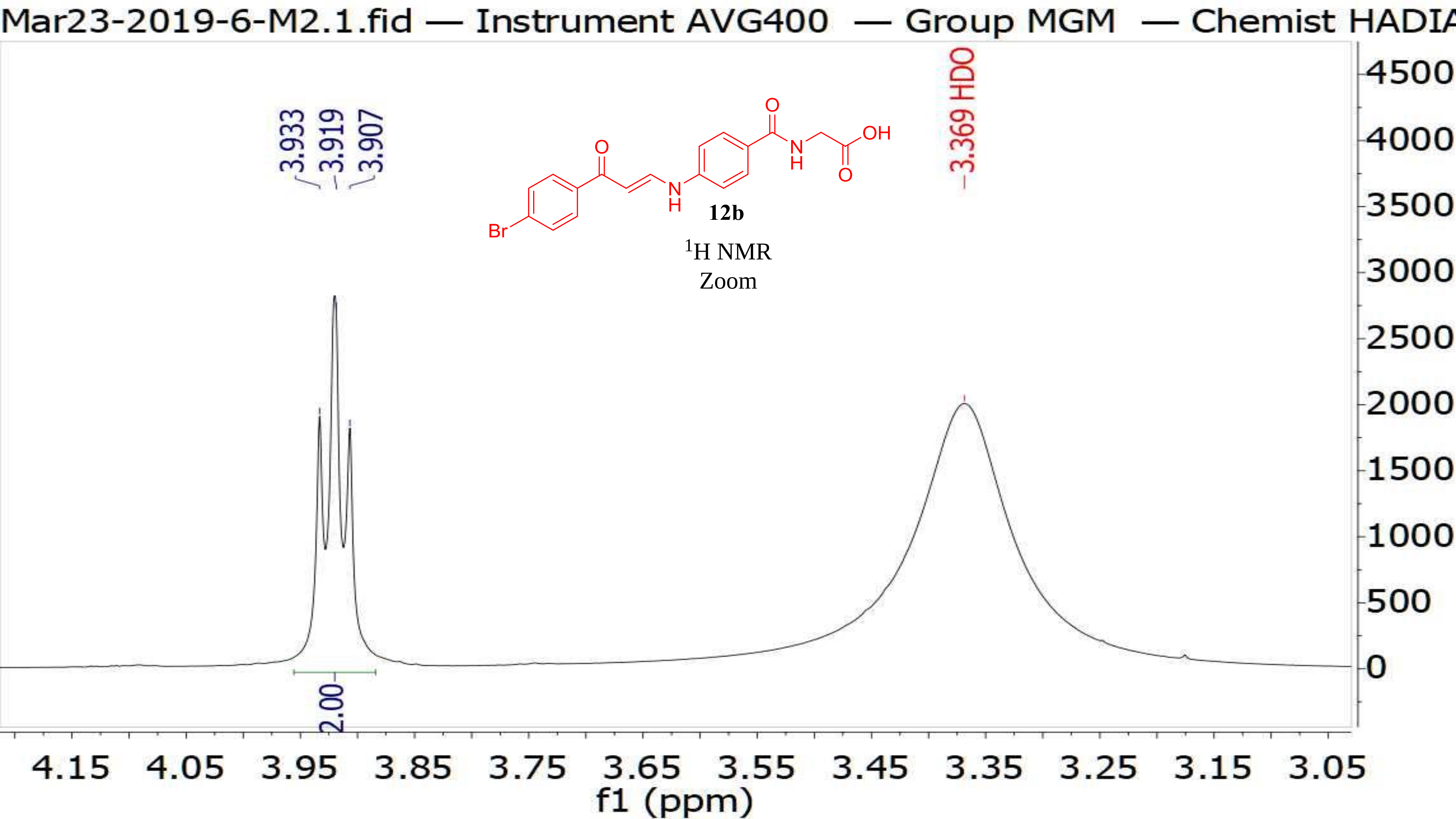
Mar23-2019-6-M2.1.fid — Instrument AVG400 — Group MGM — Chemist HADIA
3.933
3.919
3.907
3.369 HDO
Br
O
N
H
O
N
H
OH
O
12b
1H NMR
Zoom
2.00
4500
4000
3500
3000
2500
2000
1500
1000
500
0
4.15
4.05
3.95
3.85
3.75
3.65
3.55
3.45
3.35
3.25
3.15
3.05
f1 (ppm)

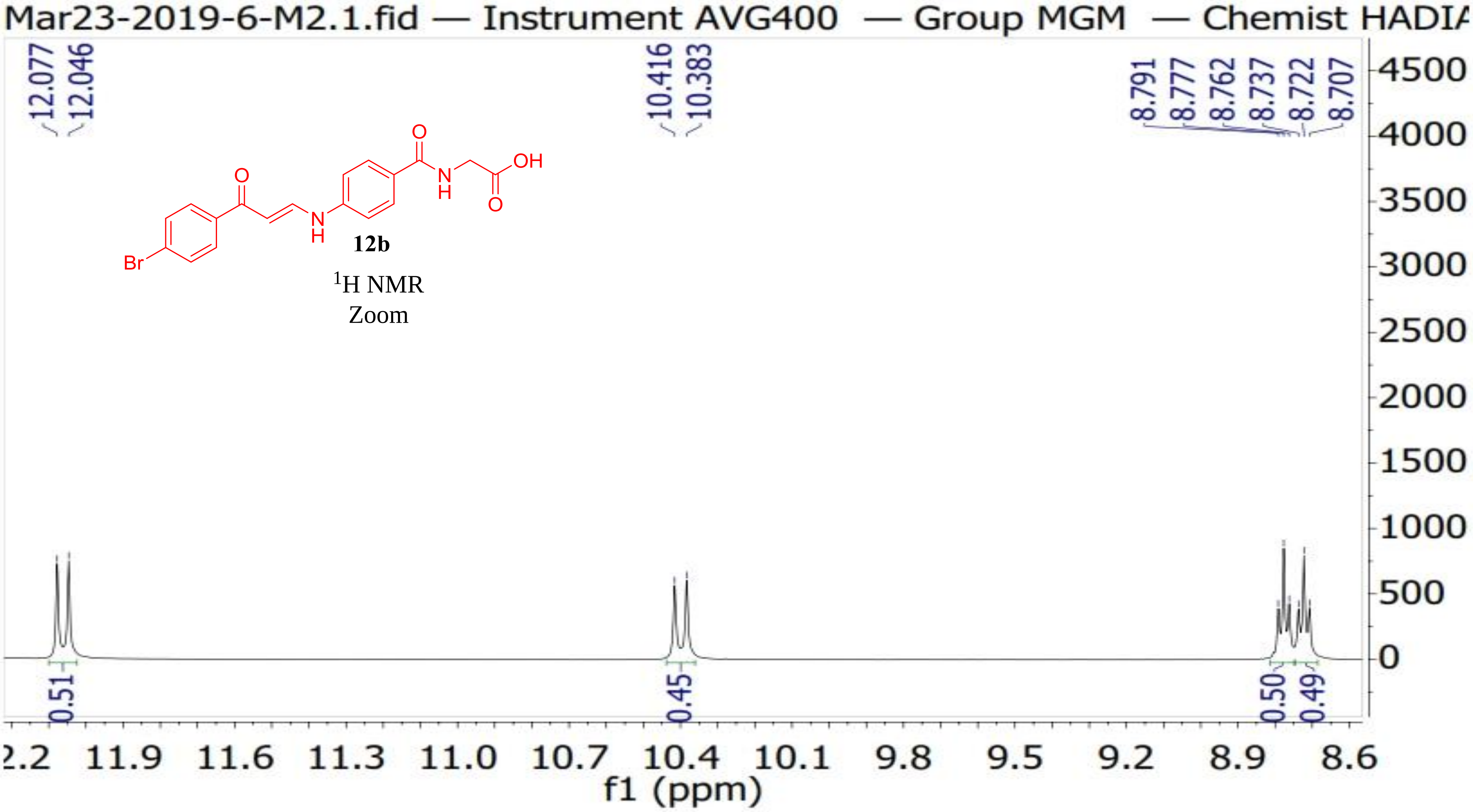
Mar23-2019-6-M2.1.fid — Instrument AVG400 — Group MGM — Chemist HADIA
12.077
12.046
10.416
10.383
8.791
8.777
8.762
8.737
8.722
8.707
12b
^{1}H NMR
Zoom
Br
O
N
H
OH
0.51
0.45
0.50
0.49
f1 (ppm)

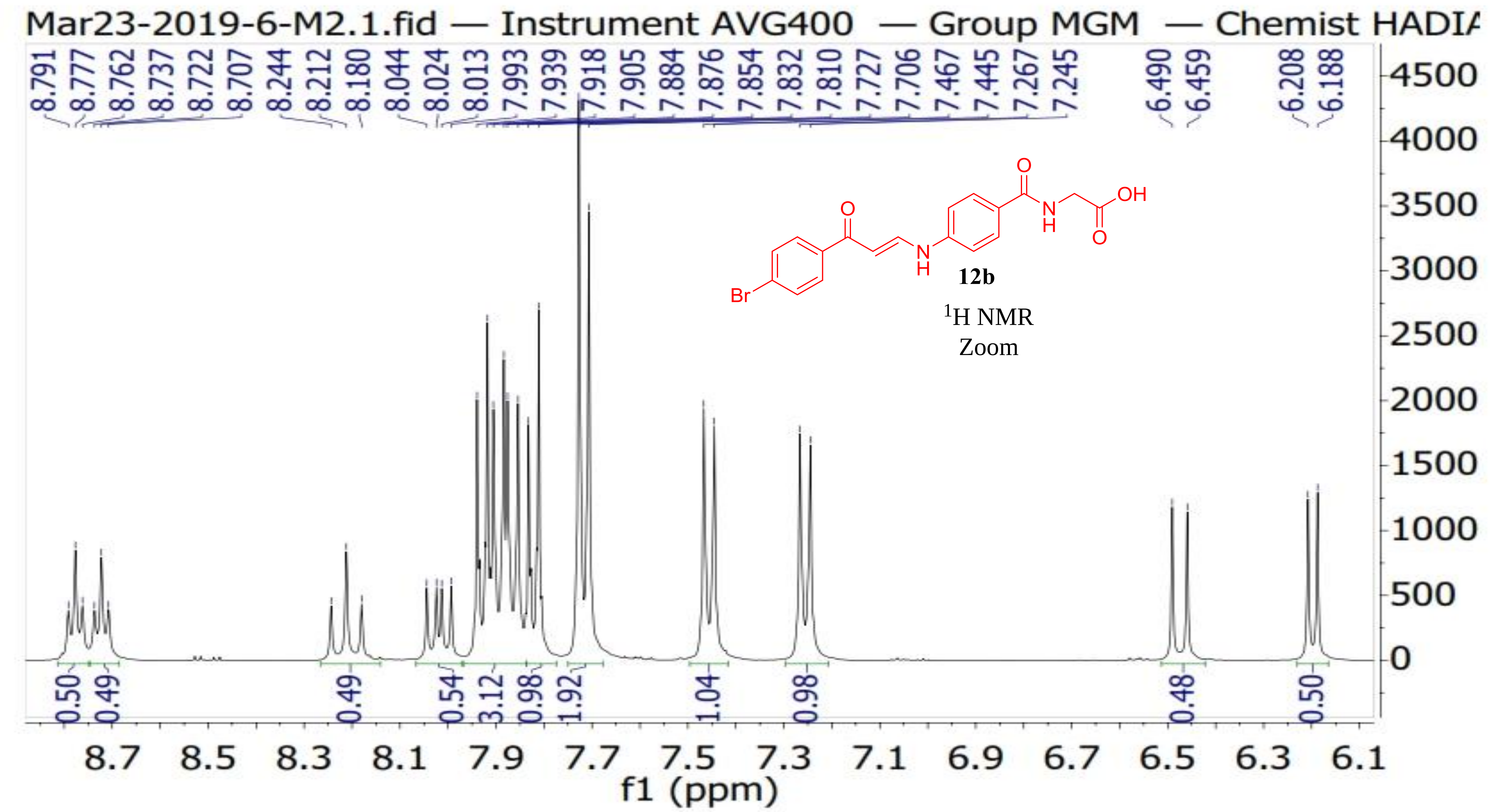
Mar23-2019-6-M2.1.fid — Instrument AVG400 — Group MGM — Chemist HADIA
8.791 8.777 8.762 8.737 8.722 8.707 8.244 8.212 8.180 8.044 8.024 8.013 7.993 7.939 7.918 7.905 7.884 7.876 7.854 7.832 7.810 7.727 7.706 7.467 7.445 7.267 7.245 6.490 6.459 6.208 6.188
Br
O
N
H
O
N
H
OH
O
12b
1H NMR
Zoom
0.50 0.49 0.49 0.54 3.12 0.98 1.92 1.04 0.98 0.48 0.50
f1 (ppm)

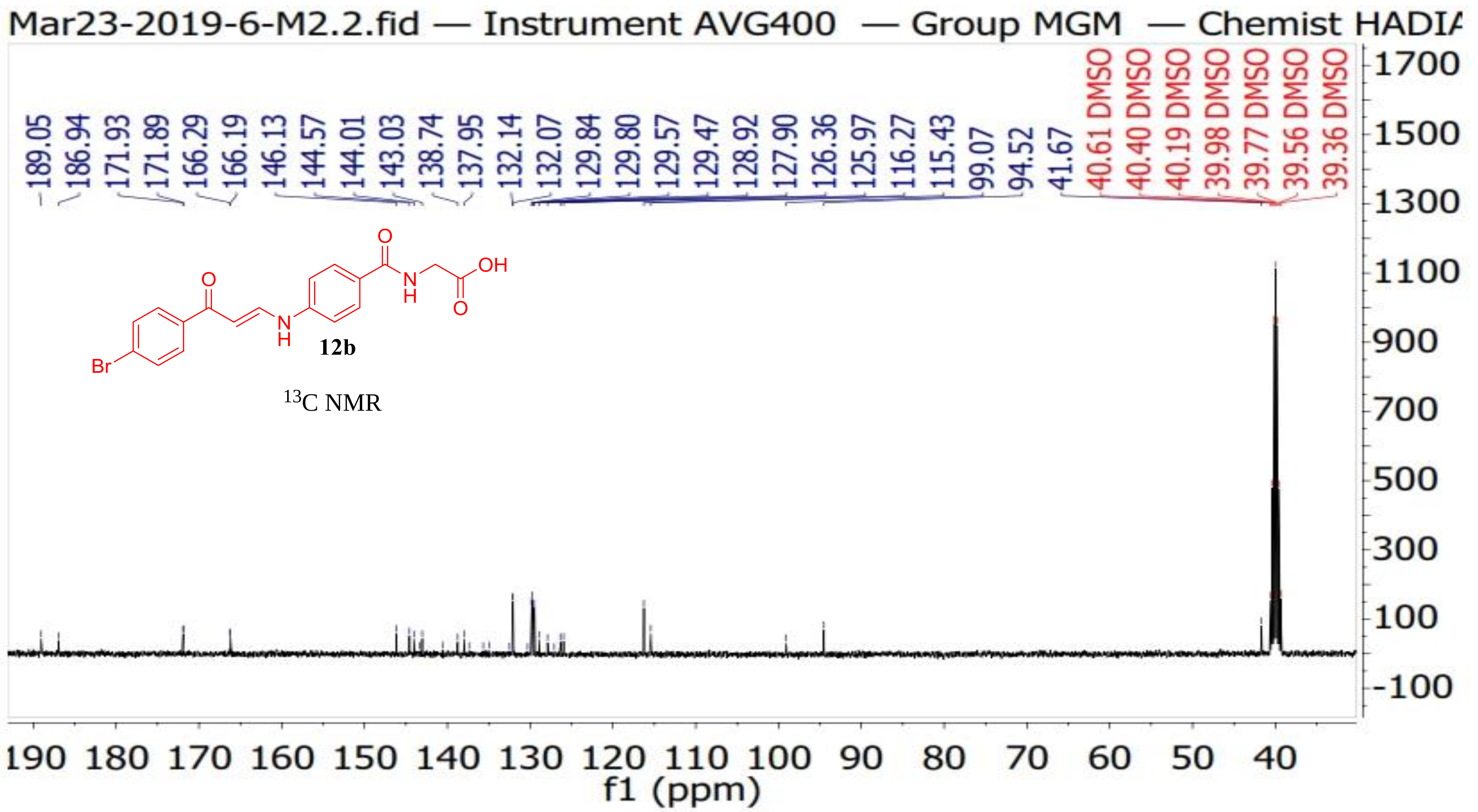

Mar23-2019-6-M2.2.fid — Instrument AVG400 — Group MGM — Chemist HADIA
189.05
186.94
171.93
171.89
166.29
166.19
146.13
144.57
144.01
143.03
138.74
137.95
132.14
132.07
129.84
129.80
129.57
129.47
128.92
127.90
126.36
125.97
116.27
115.43
99.07
94.52
41.67
40.61 DMSO
40.40 DMSO
40.19 DMSO
39.98 DMSO
39.77 DMSO
39.56 DMSO
39.36 DMSO
Br
O
N
H
O
N
H
OH
O
12b
^{13}C NMR
190 180 170 160 150 140 130 120 110 100 90 80 70 60 50 40
f1 (ppm)
1700 1500 1300 1100 900 700 500 300 100 -100

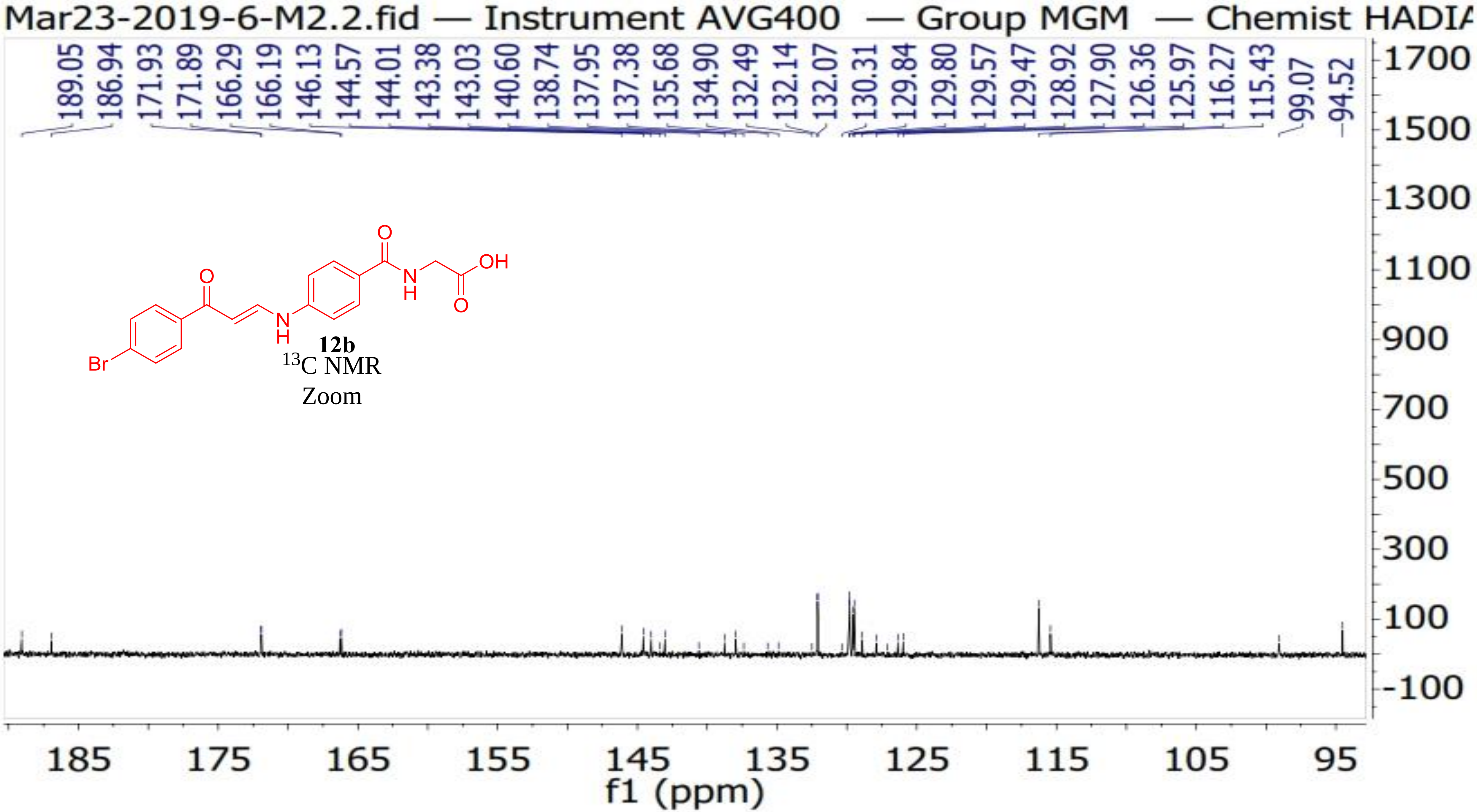
Mar23-2019-6-M2.2.fid — Instrument AVG400 — Group MGM — Chemist HADI
189.05
186.94
171.93
171.89
166.29
166.19
146.13
144.57
144.01
143.38
143.03
140.60
138.74
137.95
137.38
135.68
134.90
132.49
132.14
132.07
130.31
129.84
129.80
129.57
129.47
128.92
127.90
126.36
125.97
116.27
115.43
99.07
94.52
Br
O
N
H
O
N
H
OH
O
12b
^{13}C NMR
Zoom
1700
1500
1300
1100
900
700
500
300
100
-100
185
175
165
155
145
135
125
115
105
95
f1 (ppm)

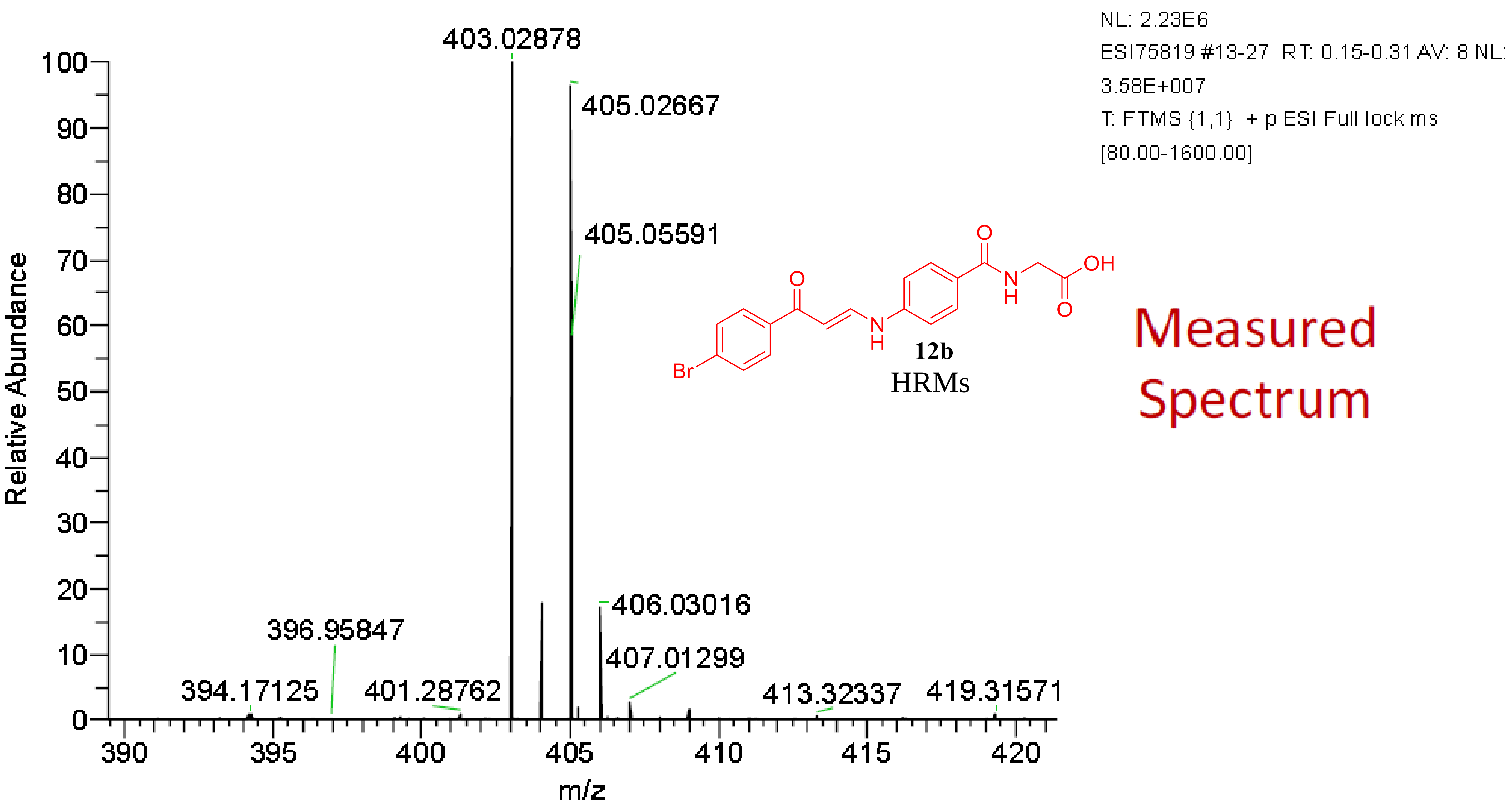
NL: 2.23E6
ESI75819 #13-27 RT: 0.15-0.31 AV: 8 NL: 3.58E+007
T: FTMS {1,1} + p ESI Full lock ms [80.00-1600.00]
Relative Abundance
m/z
403.02878
405.02667
405.05591
406.03016
407.01299
396.95847
394.17125
401.28762
413.32337
419.31571
Br
O
N
H
OH
Measured Spectrum

12b
HRMs
